# Reversible Capture and Release of a Ligand Mediated by a Long-Range Relayed Polarity Switch in a Urea Oligomer

**DOI:** 10.1021/jacs.1c11928

**Published:** 2022-02-10

**Authors:** Steven M. Wales, David T. J. Morris, Jonathan Clayden

**Affiliations:** School of Chemistry, University of Bristol, Bristol BS8 1TS, U.K.

## Abstract

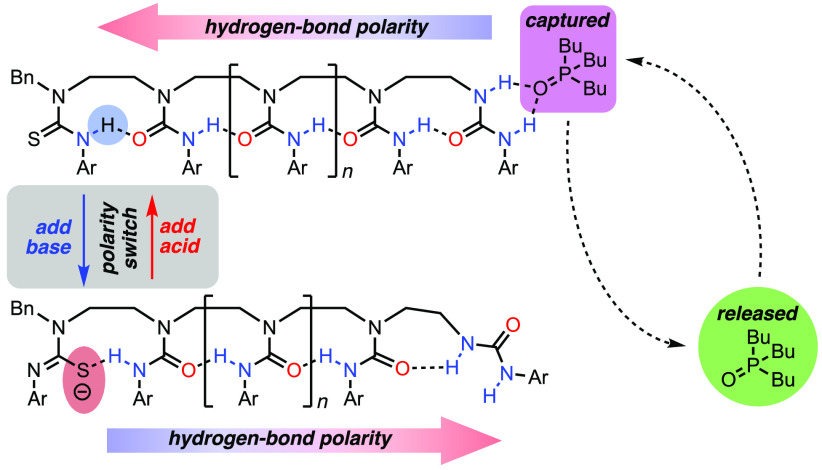

Ethylene-bridged oligoureas characterized by a continuous, switchable chain of hydrogen bonds and carrying a binding site (an N,N′-disubstituted urea) for a hydrogen-bond-accepting ligand (a phosphine oxide) were synthesized. These oligomers show stronger ligand binding when the binding site is located at the hydrogen-bond-donating terminus than when the same binding site is at the hydrogen-bond-accepting terminus. An acidic group at the terminus remote from the binding site allows hydrogen bond polarity, and hence ligand binding ability, to be controlled remotely by a deprotonation/reprotonation cycle. Addition of base induces a remote conformational change that is relayed through up to five urea linkages, reducing the ability of the binding site to retain an intermolecular association to its ligand, which is consequently released into solution. Reprotonation returns the polarity of the oligomer to its original directionality, restoring the function of the remote binding site, which consequently recaptures the ligand. This is the first example of a synthetic molecular structure that relays intermolecular binding information, and these “dynamic foldamer” structures are prototypes of components for chemical systems capable of controlling chemical function from a distance.

The ability to relay, amplify, and process information distinguishes molecular systems in a biological context from those that are purely chemical.^1−4^ Information processing in biology involves intermolecular interactions, either between biological macromolecules^5^ or between a macromolecule and a small ligand,^6,7^ with messages transmitted by way of conformational changes that propagate through those macromolecules.^8,9^

Communication devices^10^ of this type are commonplace in biology,^11−14^ and analogous spatial molecular communication has been achieved in synthetic molecules by induction of conformational changes at the terminus of an oligomeric structure.^15−17^ Examples have involved communication of chirality through contiguous atropisomeric axes^18^ or the screw-sense preference of a helix^19^ or communication of polarity through a flexible chain of hydrogen bonds.^20,21^ Without exception, such synthetic communication devices either exploit *intra*molecular interactions^22,23^ or undergo irreversible change through chemical reaction,^12,13,24^ precluding more general and reversible chemical function. To date, there is no artificial molecular communication device that allows continuous remote control of *inter*molecular interactions commonly seen in biology.

Here we report a molecular communication device that enables the control of noncovalent and reversible intermolecular interactions by a signal that is transmitted through a conformational change. Our general design concept is illustrated in Figure 1. An oligomeric structure in its “native state” (a) carries a terminal binding site that selectively binds a ligand. On input of a signal remote from the binding site (b), a conformational change is communicated to the binding site, disrupting binding and releasing the ligand into solution (c). Ligand release is reversible: removal of the input signal allows the binding site to reassemble and the ligand to return to its bound state.

**Figure 1:**
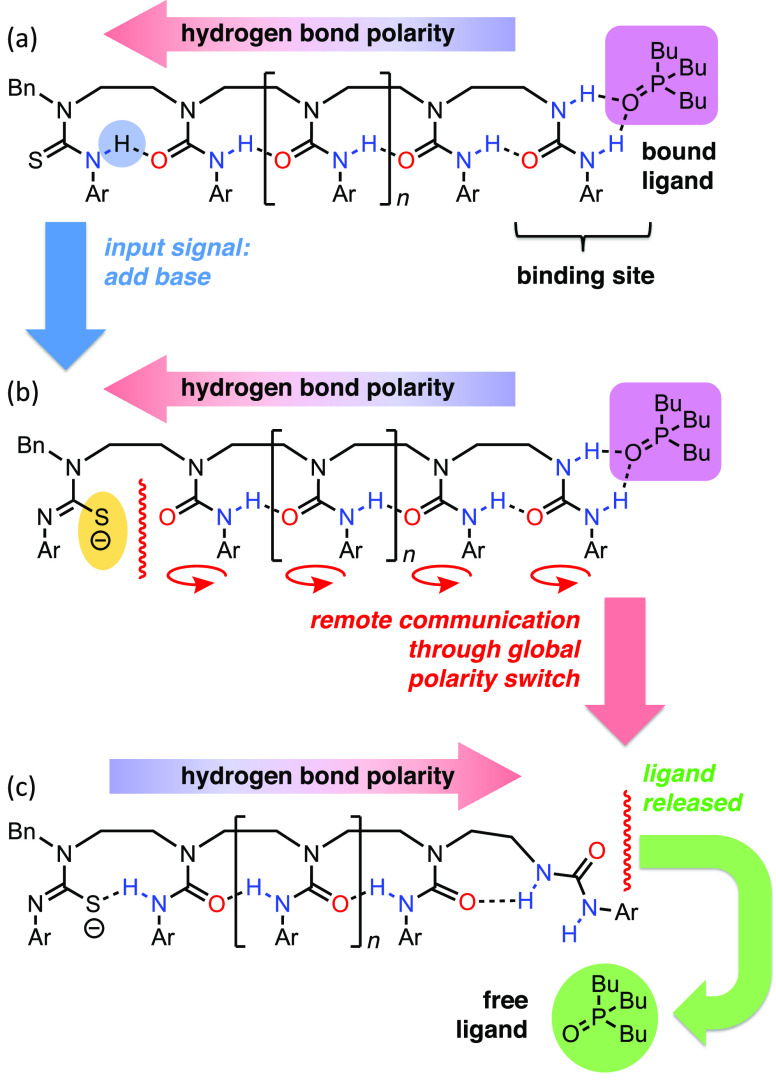
A conceptual framework for modulating ligand binding affinity through remote induction of global conformational changes.

In this instance, the input signal is provided by a change in pH, which leads to the reversible deprotonation and reprotonation of a thiourea functional group that “translates” pH into conformational change by mutating from a hydrogen bond acceptor to a hydrogen bond donor. This switch in polarity reverses the directionality of a chain of hydrogen bonds linking a series of urea functions, which disrupts the intermolecular interaction of a hydrogen bond acceptor (a phosphine oxide) with a terminal binding site.

We chose as this terminal binding site an electron-deficient N,N′-disubstituted urea function (Ar = 3,5-bis(trifluoromethyl)phenyl, abbreviated as “BTMP urea”).^25^ To establish the ability of induced hydrogen bond polarity to govern the local conformation of the BTMP urea—and hence its availability for ligand binding—oligomers **1** and **2** were synthesized (Figure 2a) in which each polarity-controlling group (the thiourea hydrogen bond donor in **1** and the *N*,*N*′-dimethylurea hydrogen bond acceptor in **2**) is separated from the BTMP urea by a hydrogen-bonded chain of three trisubstituted ureas.^20^ We expected these molecules to maximize the stability of their hydrogen-bonded network by adopting hydrogen-bonding patterns of opposite directionality.

**Figure 2:**
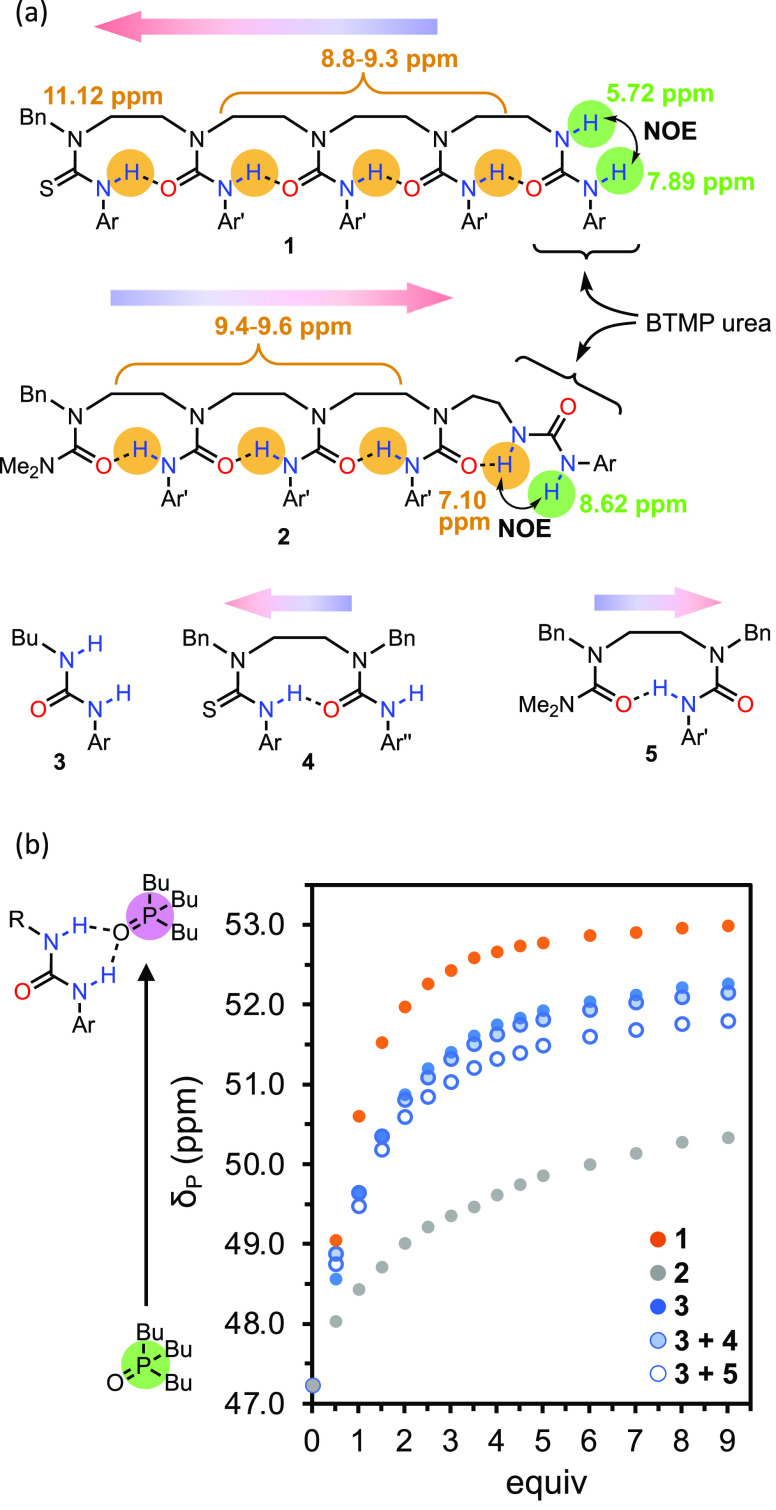
(a) BTMP ureas **1**–**5** (Ar = [3,5-(CF_3_)_2_]C_6_H_3_; Ar′ = *p*-BuOC_6_H_4_; Ar″ = *p*-MeOC_6_H_4_). (b) Titration experiments showing the change in the chemical shift in the ^31^P NMR spectrum of Bu_3_PO (2 mM, CH_2_Cl_2_) when titrated with ureas (0–9 equiv).

Oligomers **1** and **2** each showed a major conformer populated to ≥75% in CD_2_Cl_2_ (25 mM) at −10 °C (Figures S1–S11). In **1**, the more upfield chemical shifts of the alkyl and aryl N–H signals of the BTMP urea (Figure 2a: δ_H_ = 5.72 and 7.89 ppm, respectively) indicate that these (green) N–Hs are not involved in intramolecular hydrogen bonding, while in **2**, the (orange) alkyl N–H signal appears significantly further downfield (δ_H_ = 7.10 ppm) due to hydrogen bonding to the adjacent urea carbonyl group. In both **1** and **2**, a strong NOE correlation between the N–H signals of the BTMP urea (Figures S5 and S11) shows that the binding site adopts a *syn*,*syn* conformation, with the N–H bonds orientated parallel to one another. In **1**, these N–H bonds are available for intermolecular hydrogen bonding, while **2** cannot bind an external ligand without breaking an intramolecular hydrogen bond. The conformational distribution of **1** is largely insensitive to concentration and the number and identity of the internal urea linkages but does vary notably with solvent (Table S1).

Differences in the binding properties of **1** and **2** were explored by ^31^P NMR using the strong hydrogen bond acceptor Bu_3_PO (β = 10.7).^26,27^ Titration of Bu_3_PO with **1** and **2** (0–9 equiv) in CH_2_Cl_2_ (2 mM) resulted in values of Δδ_P_ (from the initial δ_P_ = 47.23 ppm) of +5.76 and +3.11 ppm, respectively (Figure 2b). A 1:1 binding model gave binding constants of 1490 ± 82 M^–1^ (for **1**) and 311 ± 21 M^–1^ (for **2**),^28^ showing that **1** binds the phosphine oxide almost 5 times more strongly than **2**. The BTMP urea is itself a powerful hydrogen bond donor,^20^ but these results demonstrate that the even more strongly hydrogen-bond-donating thiourea in **1** can override the BTMP urea’s hydrogen-bonding preference.

Further information about binding was gained by using model compounds **3**–**5**. N,N*′*-Disubstituted urea **3**, an isolated binding site, has a Bu_3_PO binding constant of 715 ± 16 M^–1^ (Δδ_P_ at 9 equiv = +5.04 ppm), showing that binding is enhanced by the “matched” polarity of **1** and weakened by the “mismatched” polarity of **2**. Under the same conditions, titrations of Bu_3_PO with a 1:1 mixture of **3** and **4** (an isolated thiourea function), as well as a 1:1 mixture of **3** and **5** (an isolated *N*,*N*′-dimethylurea function), gave similar binding curves to **3** alone.^29^ Neither **4** nor **5** alone (5 equiv) had any significant effect on the ^31^P NMR chemical shift of Bu_3_PO (Δδ_P_ < 0.4 ppm, Figures S35 and S37), confirming that the values of Δδ_P_ observed in all titrations (Figure 2b) are solely due to binding of Bu_3_PO to the BTMP urea. Collectively, these results confirm that the opposing polarities of the hydrogen bond chains in **1** and **2**, and the conformational preferences consequently induced in the BTMP urea, are responsible for their differing binding affinities to Bu_3_PO.

Using this information, we designed a communication device in which a BTMP urea is switched remotely between the role of a hydrogen-bond donor and a hydrogen-bond acceptor in response to an external input. Switchable control was enabled by a relatively acidic thiourea that in its neutral form acts as a powerful hydrogen bond donor but on treatment with base is deprotonated to reveal a hydrogen-bond-accepting thiourea anion. Representative thiourea **4** provided a model of this behavior. ^1^H NMR spectroscopy in CD_2_Cl_2_ confirmed that the thiourea N–H (δ_H_ = 10.58 ppm) hydrogen bonds strongly with the adjacent urea carbonyl (Figure 3). Consequently, the N–H of the adjacent urea (δ_H_ = 6.46 ppm) forms no intramolecular hydrogen bond. Upon deprotonation of the thiourea with 1 equiv of phosphazene base *t*-BuN=P(NMe_2_)_3_ (chosen because the conjugate acid [*t*-BuHN–P(NMe_2_)_3_]^+^ is a poor hydrogen bond donor), loss of the thiourea N–H signal (yellow) is accompanied by upfield shifts of the thiourea aryl protons (gray) and an upfield shift of the thiocarbonyl signal in the ^13^C NMR spectrum (Figure S26). Concurrently, the urea N–H signal (green) shifts downfield to δ_H_ = 9.35 ppm (Δδ_H_ = +2.89 ppm), marking the formation of a strong hydrogen bond to the resultant thiourea anion and a switch in hydrogen-bond polarity. Reprotonation of the thiourea anion in the same mixture with 1 equiv of [4-Cl-pyH]^**+**^·[BAr^F^_4_]^−^ returned the NMR signals of **4** to their original positions, demonstrating that the byproducts [*t*-BuHN–P(NMe_2_)_3_]^+^·[BAr^F^_4_]^−^ and 4-chloropyridine do not interfere with the native hydrogen bonding in **4**.

**Figure 3:**
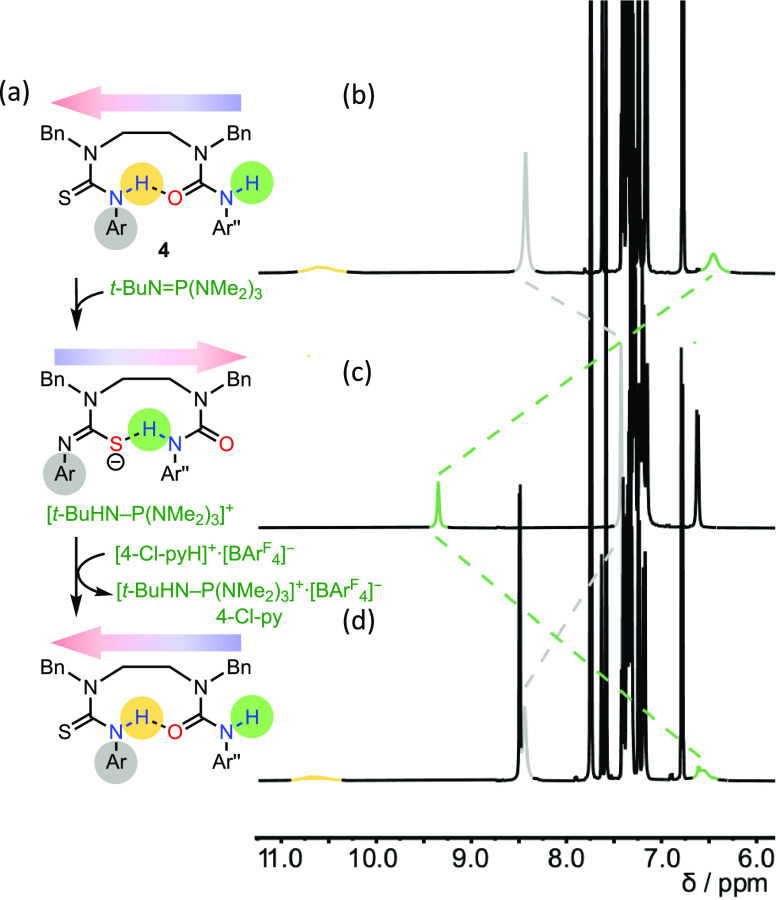
(a) Base-mediated hydrogen bond polarity switching of thiourea transmitter **4**. (b) ^1^H NMR spectrum of **4** in CD_2_Cl_2_ at 42 mM, (c) with the addition of *t*-BuN=P(NMe_2_)_3_ (1 equiv) and (d) on addition of [4-Cl-pyH]^**+**^·[BAr^F^_4_]^−^ (1 equiv). Ar = Ar^F^ = [3,5-(CF_3_)_2_]C_6_H_3_; Ar″ = *p*-MeOC_6_H_4_.

The relayed effect of deprotonating the thiourea function of **1** on the terminal binding of the BTMP urea to Bu_3_PO was investigated by using ^31^P NMR spectroscopy (Figure 4). First, **1** (5 equiv) was added to bind the Bu_3_PO, resulting in a downfield shift from δ_P_ = 47.23 ppm (free Bu_3_PO) to δ_P_ = 52.66 ppm (Δδ_P_ = +5.43 ppm: Bu_3_PO 92% bound, Figure S17). Upon addition of equimolar *t*-BuN=P(NMe_2_)_3_, deprotonation of the thiourea of **1** (Figure S31) was accompanied by a new signal arising from [*t*-BuHN–P(NMe_2_)_3_]^+^·**1**^**–**^ in the ^31^P NMR spectrum (Figure S30).^30^ Simultaneously, the Bu_3_PO signal shifted upfield to δ_P_ = 48.31 ppm (Δδ_P_ = −4.35 ppm) (Figure 4a), consistent with the release of Bu_3_PO from the remote binding site as a result of thiourea deprotonation (Figure S31).

**Figure 4:**
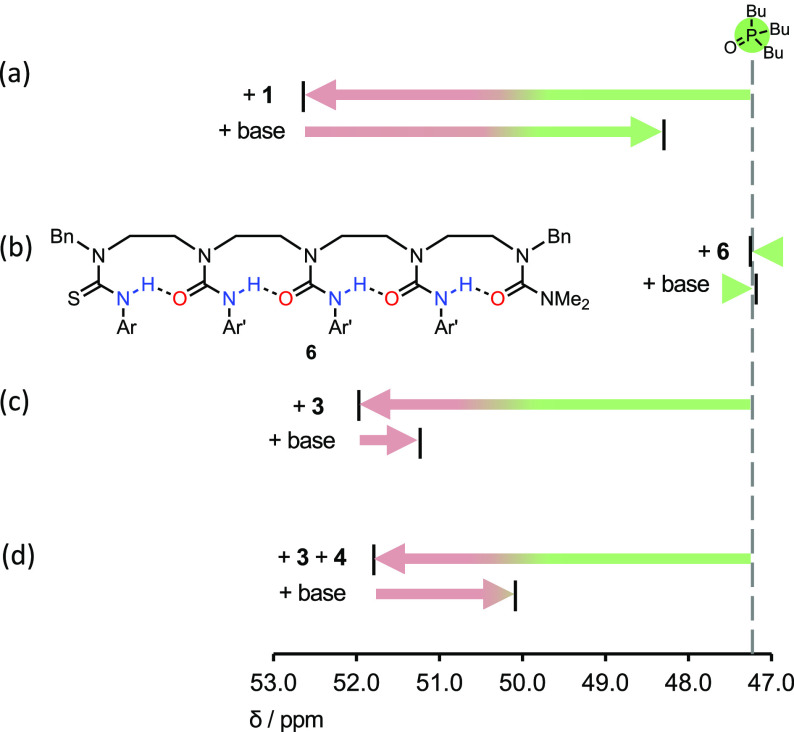
^31^P NMR chemical shift of Bu_3_PO (2 mM, CD_2_Cl_2_) when treated with 5 equiv of a variant urea oligomer (namely (a) **1**, (b) **6**, (c) **3**, and (d) **3** and **4**) followed by 5 equiv of *t*-BuN=P(NMe_2_)_3_. Ar = [3,5-(CF_3_)_2_]C_6_H_3_; Ar′ = *p*-BuOC_6_H_4_.

Additional control experiments (Figure 4b–d) confirmed that induced release of Bu_3_PO results from a relayed polarity switch. Bu_3_PO was treated with a series of modified urea oligomers (5 equiv), each lacking one or more components of the integrated communication system, followed by *t*-BuN=P(NMe_2_)_3_ (5 equiv). Oligomer **6**, whose binding site is blocked by alkylation, was unable to bind Bu_3_PO, and minimal Δδ_P_ resulted with either **6** or **6** + *t*-BuN=P(NMe_2_)_3_ (Figure 4b, Figures S38 and S39). This result confirms that the conjugate acid [BuHN–P(NMe_2_)_3_]^+^ is itself unable to hydrogen bond to Bu_3_PO. The isolated urea **3** binds Bu_3_PO (Δδ_P_ = +4.76 ppm, Figure S32) but is resistant to deprotonation by *t*-BuN=P(NMe_2_)_3_ (Figure 4c and Figure S32). When Bu_3_PO was complexed to **3** in the presence of **4** (Δδ_P_ = +4.58 ppm)—representing a “broken” device with a disconnected binding site—addition of base (Figure 4d, Figures S33 and S34) was accompanied by a modest upfield shift of Bu_3_PO (Δδ_P_ = −1.71 ppm), indicating weakly competitive intermolecular binding of **3** to the thiourea anion of **4**^**–**^, which partially liberates the phosphine oxide.^31^

A fully functioning device capable of reversible induced capture and release of a ligand over multiple cycles was demonstrated with the homologous oligourea **7**, which communicates information through a hydrogen-bonded chain of five internal ureas (Figure 5). The protonation state of the transmitting thiourea, which remotely controls the receiver’s binding affinity for the ligand, was monitored by ^1^H NMR (Figure S41), while the state of the ligand—bound or free—was simultaneously monitored by ^31^P NMR.

**Figure 5:**
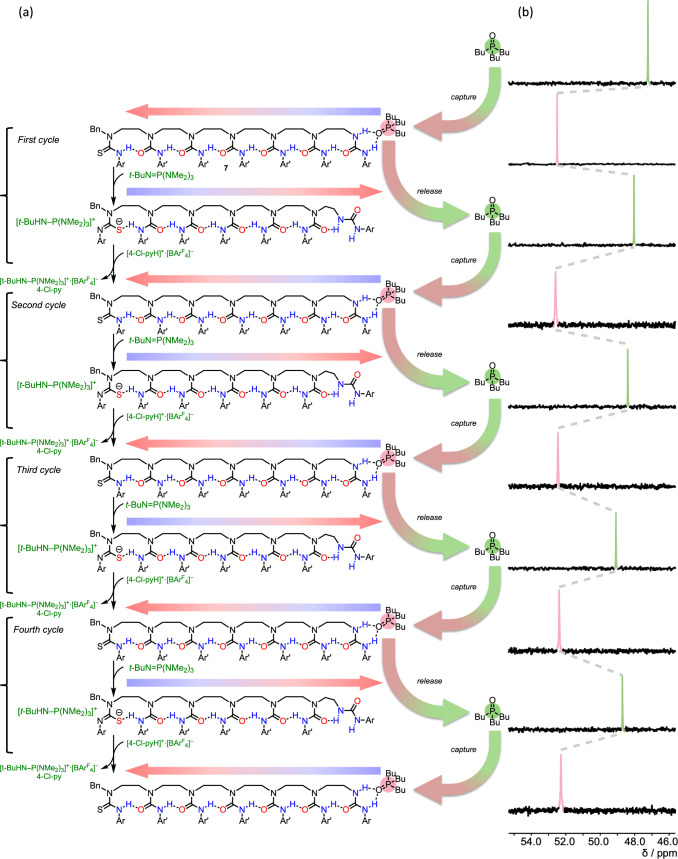
(a) Base-sensitive thiourea-capped oligomer **7** functioning as a ligand-capturing device. **7** captures Bu_3_PO by hydrogen bonding at its terminal disubstituted urea binding site. Deprotonation of the remote thiourea with *t*-BuN=P(NMe_2_)_3_ transmits a global polarity change to the disubstituted urea which releases the phosphine oxide; reprotonation with [4-Cl-pyH]^**+**^·[BAr^F^_4_]^−^ recaptures the phosphine oxide. (b) Characteristic changes in the ^31^P NMR chemical shift of Bu_3_PO (2 mM, CD_2_Cl_2_) on adding **7** (5 equiv), followed by repeated sequential additions of *t*-BuN=P(NMe_2_)_3_ (5 equiv) and [4-Cl-pyH]^**+**^·[BAr^F^_4_]^−^ (5 equiv). Ar = Ar^F^ = [3,5-(CF_3_)_2_]C_6_H_3_; Ar′ = *p*-BuOC_6_H_4_.

The switching cycle started with the addition of **7** (5 equiv) to bind Bu_3_PO (Figure 5), which induced a downfield shift (δ_P_ = 52.51 ppm, Δδ_P_ = +5.28 ppm) in the ^31^P NMR spectrum. Thiourea deprotonation with *t*-BuN=P(NMe_2_)_3_ released this Bu_3_PO back into solution (Δδ_P_ = −4.47 ppm). The ligand was then repeatedly recaptured and released by three sequential cycles of reprotonation with [4-Cl-pyH]^**+**^·[BAr^F^_4_]^−^ and deprotonation with *t*-BuN=P(NMe_2_)_3_ in one pot. Finally, the addition of further [4-Cl-pyH]^**+**^·[BAr^F^_4_]^−^ recaptured the Bu_3_PO (δ_P_ = 52.28 ppm). Even after 4.5 capture and release cycles, communication device **7** maintains its binding function with minimal loss in efficiency.

In summary, a molecular communication device that can reversibly and remotely trigger a chemical response—namely the release and recapture of a ligand—has been realized. Information about the pH of an acidic thiourea’s surroundings is converted to communicable hydrogen-bond polarity, which is relayed through a chain of hydrogen bonds to control the binding properties of a remote N,N′-disubstituted urea. Capture and release of the ligand can then be switched upon sequential treatment with acid and base several times in one pot. The remote modulation of an intermolecular interaction is reminiscent of actin treadmilling, suggesting future use of polarity reversal in the design of actin mimetics.^32^ The ability to bind strong hydrogen bond acceptors, including Bu_3_PO,^25^ correlates strongly with catalytic activity in hydrogen bond donors,^33,34^ suggesting that structures related to **1** and **7** might furthermore function as remotely switchable catalysts.

## Supporting Information Available

Synthetic schemes, experimental procedures and compound characterization data, details of conformational analysis, titration data and binding constant determination, ^31^P and ^1^H NMR spectra for capture and release experiments, and NMR spectra of novel compounds (PDF)
