# Supplementary material for: Reversible Capture and Release of a Ligand Mediated by a Long-Range Relayed Polarity Switch in a Urea Oligomer
Source: J Am Chem Soc. 2022 Feb 10;144(7):2841–6. doi: 10.1021/jacs.1c11928 (PMC9097480; doi:10.1021/jacs.1c11928)
Supplement: Supplementary file 1 — ja1c11928_si_001.pdf [file ja1c11928_si_001.pdf]

Reversible capture and release of a ligand mediated by a long-range relayed polarity switch in a urea oligomer

Steven M. Wales, David T. J. Morris and Jonathan Clayden*

Email: j.clayden@bristol.ac.uk

Contents

Supplementary Methods 2

General Information 2

Synthetic Schemes 3

Experimental Procedures 5

Conformational Analysis 23

Titrations, Binding Constants and Related Experiments 49

^{31}P NMR Capture-And-Release Experiments and Supporting ^{1}H NMR Spectra 61

NMR Spectra of Novel Compounds 80

References 99

Supplementary Methods

General Information

Where specified, procedures were performed under an atmosphere of nitrogen. Air and moisture-sensitive liquids/solutions were transferred to reaction vessels by syringe under an atmosphere of nitrogen. Solvents and reagents were purchased from commercial suppliers and were used without further purification unless otherwise specified. Agitation was achieved using Teflon coated stirrer bars by magnetic induction. All thin layer chromatography (TLC) experiments were conducted on pre-coated plastic plates (Macherey-Nagel polygram SIL G/UV_{254}) and visualized using ultraviolet light (254 nm) or staining. Flash chromatography was performed on an automated Biotage Isolera™ Spektra Four using gradient elution on pre-packed silica gel Sfär Duo columns. Solvent systems for TLC and flash chromatography are reported in solvent:solvent volume ratios. All variable-temperature NMR experiments were conducted using a Bruker AVANCE III HD 500 MHz NMR Spectrometer with 5 mm DCH ^{13}C–^{1}H/D Cryo Probe (500 MHz). All room temperature NMR experiments were conducted using a Bruker Nano 400 Spectrometer (400 MHz) or a Bruker AVANCE III HD 500 MHz NMR Spectrometer with 5 mm DCH ^{13}C–^{1}H/D Cryo Probe (500 MHz), with chemical shifts reported (δ in ppm) relative to the specified deuterated solvent. All ^{31}P NMR spectra are referenced relative to an external standard (Ph_3PO) as detailed within. All NMR characterization experiments were performed at 25 °C and 1 atm unless otherwise specified. Multiplicity is reported as follows – s = singlet, d = doublet, t = triplet, q = quartet, m = multiplet. All spin-spin coupling constants (J) are reported in hertz (Hz) to the nearest 0.1 Hz. High-resolution mass spectrometry experiments (HR-MS) were performed on a Bruker micrOTOF Spectrometer using electrospray ionization, positive ion mode or a Bruker Ultraflex using MALDI with only molecular ion ($[M+H]^+$ or $[M+Na]^+$) peaks being reported.

Synthetic Schemes

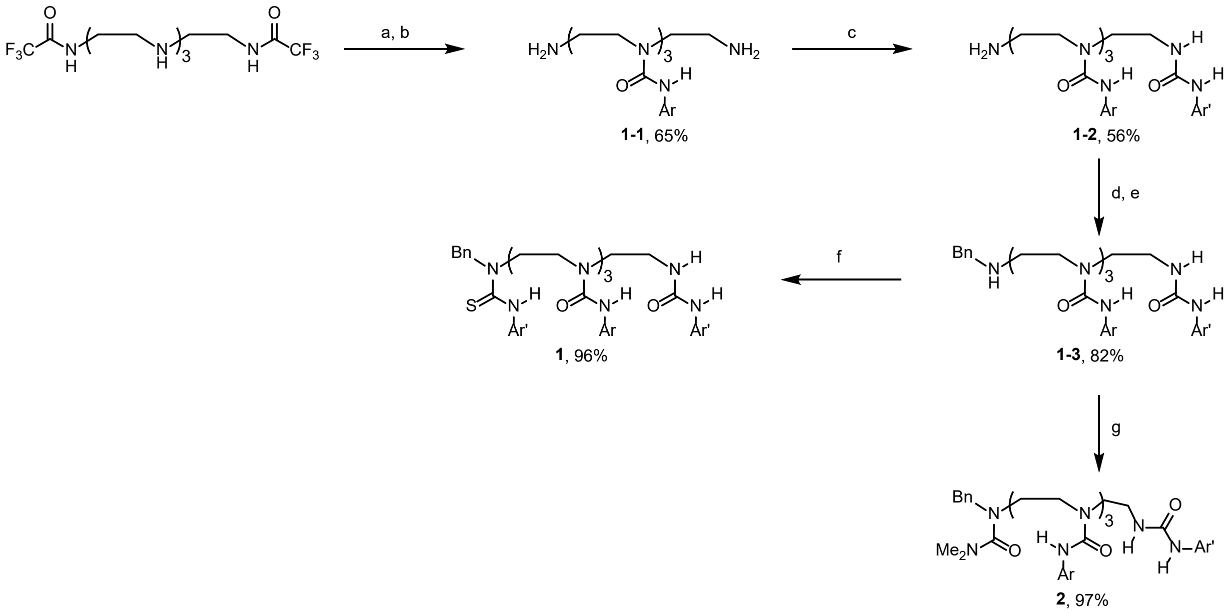

Scheme S1. Synthesis of capture-and-release oligourea **1** and control oligourea **2**. Reagents and conditions: (a) 4-*n*-butyloxyphenyl isocyanate (3.3 equiv), DCM, 0 °C to RT, 30 mins; (b) NaOH (8.0 equiv), EtOH/THF/H_2O, RT, 2 h; (c) 3,5-bis(trifluoromethyl)phenyl isocyanate (0.5 equiv, added over 80 mins, −10 °C to 0 °C), 0 °C to RT, 2.5 h; (d) benzaldehyde (1.0 equiv), MeOH/THF, RT, 20.5 h; (e) $NaBH_4$, MeOH/THF, 0 °C to RT, 3.5 h; (f) 3,5-bis(trifluoromethyl)phenyl isothiocyanate (1.0 equiv), DCM, 0 °C to RT, 1.5 h; (g) dimethylcarbamoyl chloride (1.2 equiv), TEA (1.5 equiv), DCE, 45 °C, 22 h. For all compounds, Ar = 4-*n*-BuO-Ph and Ar' = 3,5-bis(CF_3)-Ph.

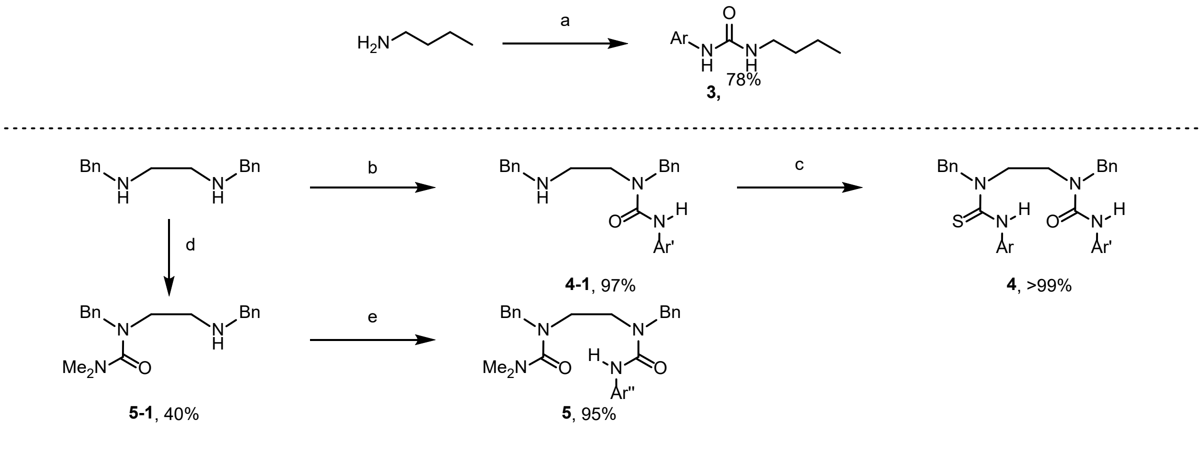

Scheme S2. Synthesis of control compounds **3**-**5**. Reagents and conditions: (a) 3,5-bis(trifluoromethyl)phenyl isocyanate (1.0 equiv), 0 °C to RT, 1 h; (b) 4-methoxyphenyl isocyanate (0.5 equiv), DCM, RT, 3 h; (c) 3,5-bis(trifluoromethyl)phenyl isothiocyanate (1.0 equiv), DCM, 0 °C to RT, 0.5 h; (d) dimethylcarbamoyl chloride (0.5 equiv), TEA (1.3 equiv), DCE, 45 °C, 30 h; (e) 4-*n*-butyloxyphenyl isocyanate (1.0 equiv), DCM, RT, 50 mins. For all compounds, Ar = 3,5-bis(CF_3)-Ph, Ar' = 4-MeO-Ph and Ar'' = 4-*n*-BuO-Ph.

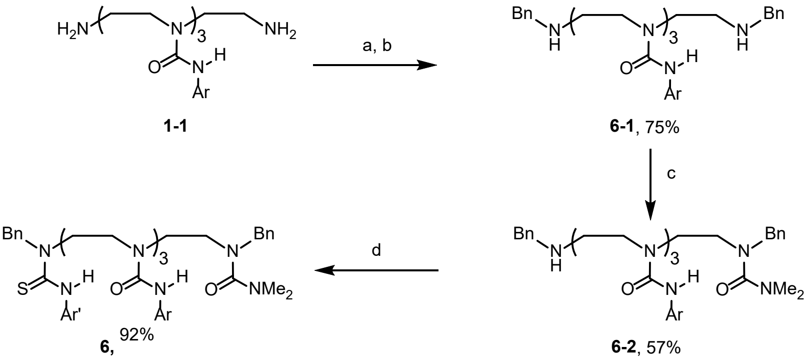

Scheme S3. Synthesis of control thiourea **6**. Reagents and conditions: (a) benzaldehyde (2.0 equiv), MeOH, RT, 17 h; (b) $NaBH_4$, MeOH, 0 °C to RT, 3 h; (c) dimethylcarbamoyl chloride (0.5 equiv), TEA (1.3 equiv), DCE, 45 °C, 24 h; (d) 3,5-bis(trifluoromethyl)phenyl isothiocyanate (1.0 equiv), DCM, 0 °C to RT, 2.5 h. For all compounds, Ar = 4-*n*-BuO-Ph and Ar' = 3,5-bis(CF_3)-Ph.

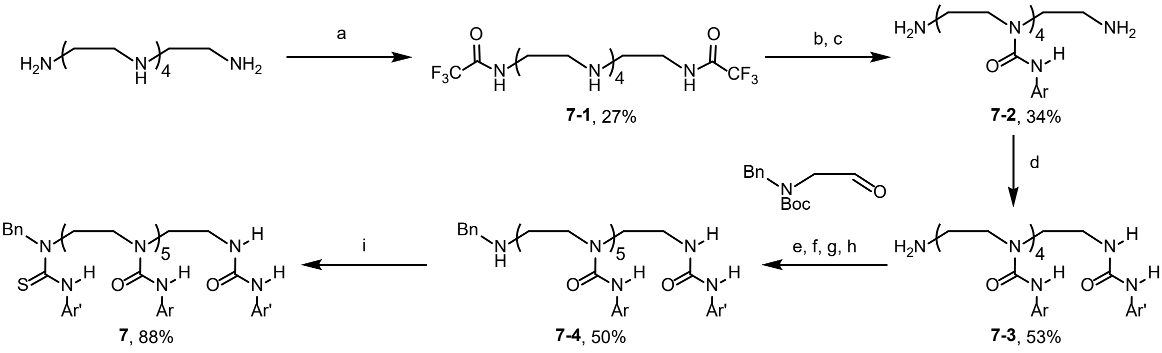

Scheme S4. Synthesis of capture-and-release oligourea **7**. Reagents and conditions: (a) Ethyl trifluoroacetate (2.0 equiv), MeOH, −78 °C to RT, 19 h; (b) 4-*n*-butyloxyphenyl isocyanate (4.4 equiv), DCM, 0 °C to RT, 20 mins; (c) NaOH (8.0 equiv), EtOH/THF/H_2O/DMF, RT, 2 h; (d) 3,5-bis(trifluoromethyl)phenyl isocyanate (1.0 equiv, added over 70 mins, −10 °C to 0 °C), 0 °C to RT, 1 h; (e) *N*-benzyl-*N*-Boc-2-aminoacetaldehyde (1.0 equiv), MeOH/THF, RT, 19 h; (f) $NaBH_4$, MeOH/THF, 0 °C to RT, 3 h; (g) 4-*n*-butyloxyphenyl isocyanate (1.0 equiv), DCM, RT, 1.5 h; (h) TFA, DCM, RT, 27.5 h (i) 3,5-bis(trifluoromethyl)phenyl isothiocyanate (1.0 equiv), DCM, RT, 2 h. For all compounds, Ar = 4-*n*-BuO-Ph and Ar' = 3,5-bis(CF_3)-Ph.

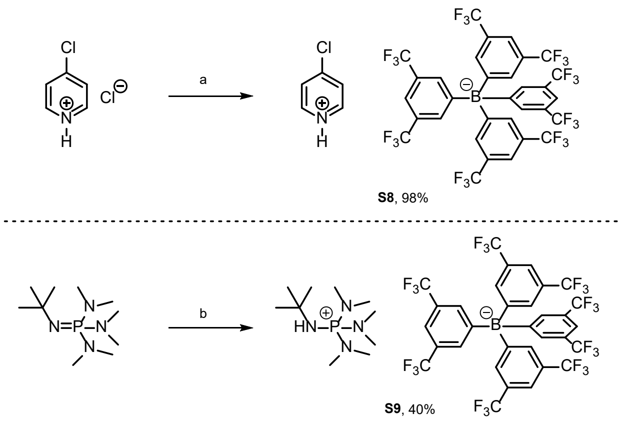

Scheme S5. Synthesis of pyridinium borate salt **S8** and phosphazenium borate salt **S9**. Reagents and conditions: (a) $NaBAr^F_4$ (1.0 equiv), MeCN, RT, 2 h; (b) **S8** (1.0 equiv), DCM, RT, 1 min.

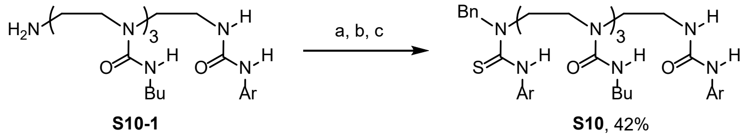

Scheme S6. Synthesis of oligourea **S10** containing alkyl ureas as the communication channel. Reagents and conditions: (a) benzaldehyde (1.0 equiv), MeOH, RT, 21 h; (b) $NaBH_4$, MeOH, 0 °C to RT, 96 h; (c) 3,5-bis(trifluoromethyl)phenyl isothiocyanate (1.0 equiv), DCM, RT, 1 h; Ar = 3,5-bis(CF_3)-Ph.

Experimental Procedures

4,7,10-Tris(4-*n*-butyloxyanilinylcarbonyl)-1,4,7,10,13-pentaazatridecane, 1-1

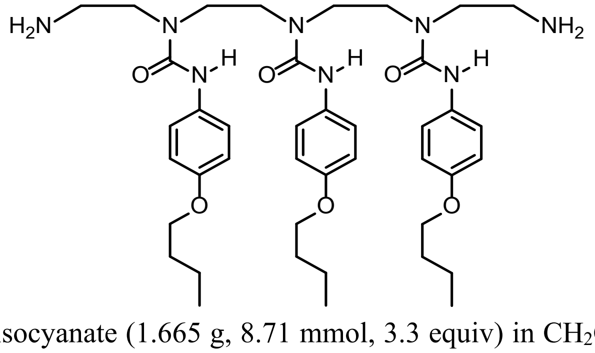

***Step 1** (tris-urea formation):* To a solution of 1,13-(bis(trifluoroacetyl))tetraethylenepentamine[1] (1.006 g, 2.64 mmol, 1.0 equiv) in lab grade CH_2Cl_2 (21.4 mL) at 0 °C under air was added over 5 min a solution of 4-butoxyphenyl isocyanate (1.665 g, 8.71 mmol, 3.3 equiv) in CH_2Cl_2 (5.0 mL) [note that the isocyanate solution was delivered into the reaction flask by filtration through a cotton pipette plug to remove a small amount of an insoluble urea impurity]. After complete addition, the ice bath was removed and the mixture was stirred at room temperature for 30 min. MeOH (5 mL) was added and the mixture was stirred for 5 min to quench any unreacted isocyanate. The mixture was concentrated *in vacuo*. To the residue was added petroleum ether/Et_2O (1:1, 20 mL) and the suspension was sonicated for ~5 min until the gum turned to a fine white powder, then the solid was collected by filtration, washed with petroleum ether/Et_2O (1:1, 20 mL) and dried to give the intermediate tris-urea (2.122 g, 84%) as an off-white solid. **TLC** –

R_f = 0.26 (SiO_2, 5:95 MeOH:CH_2Cl_2). ***Step 2** (trifluoroacetamide hydrolysis):* The product from *Step 1* (2.122 g, 2.22 mmol, 1.0 equiv) was dissolved in a mixture of lab grade THF (11.1 mL) and EtOH (22.2 mL). A solution of NaOH (711.0 mg, 17.78 mmol, 8.0 equiv) in water (11.1 mL) was added and the mixture was stirred at room temperature under air for 2 h. Most of the solvents were removed *in vacuo*, then water (15 mL) was added. The product was extracted with CH_2Cl_2 (50 mL + 30 mL) then the combined organic extracts were dried (Na_2SO_4) and concentrated. Flash chromatography (Biotage, 50 g Sfär Duo column, MeOH/[35% aqueous NH_3]/CH_2Cl_2 gradient from 0:0:100 to 10:2:88) gave the title compound (1.315 g, 78%, or 65% over two steps) as a white solid. **TLC** – R_f = 0.05 (SiO_2, 10:2:88 MeOH:[35% aqueous NH_3]:CH_2Cl_2). **^{1}H NMR** (500 MHz, $CDCl_3$) δ_H 0.95 (t, J = 7.4, 6H, 2 x C*H*$_3$), 0.96 (t, J = 7.4, 3H, C*H*$_3$), 1.46 (dq, J = 7.4, 4H, 2 x C*H*$_2$CH$_3$), 1.47 (dq, J = 7.4, 2H, C*H*$_2$CH$_3$), 1.69-1.75 (m, 6H, 3 x C*H*$_2$CH$_2$CH$_3$), 1.79 (s, 4H, 2 x N*H*$_2$), 2.86 (t, J = 4.8, 4H, 2 x C*H*$_2$NH$_2$), 3.31-3.41 (m, 8H, 4 x NC*H*$_2$), 3.45-3.50 (m, 4H, 2 x NC*H*$_2$), 3.89 (t, J = 6.6, 4H, 2 x OC*H*$_2$), 3.90 (t, J = 6.5, 2H, OC*H*$_2$), 6.79 (d, J = 9.0, 4H, 4 x Ar*H*), 6.80 (d, J = 8.9, 2H, 2 x Ar*H*), 7.32 (d, J = 7.5, 4H, 4 x Ar*H*), 7.56 (d, J = 8.9, 2H, 2 x Ar*H*), 9.02 (s, 1H, N*H*), 9.87 (s, 2H, 2 x N*H*). **^{13}C NMR** (126 MHz, $CDCl_3$) δ_C 13.9 (3 x *C*H$_3$), 19.3 (3 x *C*H$_2$CH$_3$), 31.4 (3 x *C*H$_2$CH$_2$CH$_3$), 41.7 (2 x N*C*H$_2$), 47.0 (2 x N*C*H$_2$), 47.4 (2 x N*C*H$_2$), 52.3 (2 x N*C*H$_2$), 68.1 (3 x O*C*H$_2$), 114.6 (2 x Ar*C*), 114.7 (4 x Ar*C*), 120.8 (2 x Ar*C*), 121.1 (4 x Ar*C*), 133.3 (Ar*C*), 133.4 (2 x Ar*C*), 154.6 (2 x Ar*C*), 154.6 (Ar*C*), 156.3 (*C*O), 158.0 (2 x *C*O). **HR-MS** (ESI, positive ion mode) – *m/z* for for $[C_{41}H_{62}N_8O_6+H]^+$ = 763.4865. Found 763.4830.

1-(3,5-Bis(trifluoromethyl)anilinylcarbonyl)-4,7,10-tris(4-*n*-butyloxyanilinylcarbonyl)-1,4,7,10,13-pentaazatridecane, 1-2

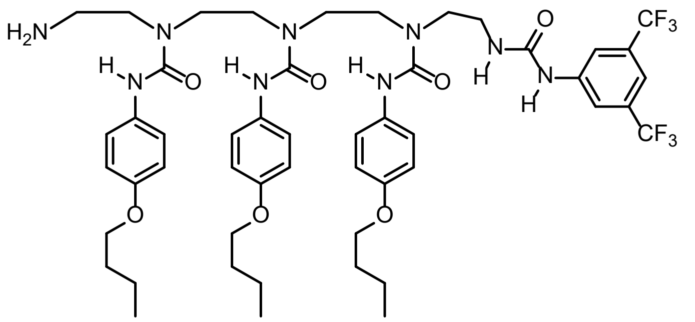

To a solution of **1-1** (695.0 mg, 0.91 mmol, 2.0 equiv) in lab grade CH_2Cl_2 (6.6 mL) at −10 °C (ice/salt bath) under N_2 was added dropwise over 80 min a solution of 3,5-bis(trifluoromethyl)phenyl isocyanate (116.2 mg, 0.46 mmol, 1.0 equiv) in CH_2Cl_2 (2.5 mL) while maintaining the cold bath temperature between −10 °C and 0 °C. After complete addition, the cold bath was allowed to warm to room temperature and the mixture was stirred for a further 2.5 h, before being concentrated *in vacuo*. Flash chromatography (Biotage, 25 g Sfär Duo column, MeOH/[35% aqueous NH_3]/CH_2Cl_2 gradient from 0:0:100 to 10:2:88) gave the title compound (261.6 mg, 56% based on the isocyanate) as a white solid. Further elution from the chromatography column returned unreacted **1-1** (424.1 mg, 61% based on total diamine used). *Data for **1-2**:* **TLC** – R_f = 0.36 (SiO_2, 10:2:88 MeOH:[35% aqueous NH_3]:CH_2Cl_2). **^{1}H NMR** (500 MHz, $CDCl_3$) δ_H 0.93 (t, J = 7.4, 3H, C*H*$_3$), 0.96 (t, J = 7.4, 3H, C*H*$_3$),

0.96 (t, J = 7.4, 3H, CH_3), 1.38-1.51 (m, 6H, 3 x CH_2CH_3), 1.63-1.76 (m, 6H, 3 x CH_2CH_3), 1.84 (s, 2H, NH_2), 2.91 (t, J = 4.5, 2H, NCH_2), 3.31-3.57 (m, 14H, 7 x NCH_2), 3.76 (t, J = 6.4, 2H, OCH_2), 3.85-3.92 (m, 4H, 2 x OCH_2), 6.72 (d, J = 8.9, 2H, 2 x Ar*H*), 6.77 (d, J = 9.0, 2H, 2 x Ar*H*), 6.80 (d, J = 9.6, 2H, 2 x Ar*H*), 6.87 (s, 1H, N*H*), 7.27 (d, J = 8.5, 2H, 2 x Ar*H*), 7.36 (s, 1H, Ar*H*), 7.44 (d, J = 8.9, 2H, 2 x Ar*H*), 7.53 (d, J = 8.9, 2H, 2 x Ar*H*), 7.62 (s, 2H, 2 x Ar*H*), 8.42 (s, 1H, N*H*), 9.01 (s, 1H, N*H*), 9.16 (s, 1H, N*H*), 10.20 (s, 1H, N*H*). **^{13}C NMR** (126 MHz, $CDCl_3$) δ_C 13.9 (CH_3), 14.0 (2 x CH_3), 19.3 (CH_2CH_3), 19.3 (2 x CH_2CH_3), 31.4 ($CH_2CH_2CH_3$), 31.5 (2 x $CH_2CH_2CH_3$), 39.1 (NCH_2), 41.9 (NCH_2), 47.0 (2 x NCH_2), 48.1 (NCH_2), 48.2 (NCH_2), 48.3 (NCH_2), 53.0 (NCH_2), 67.9 (OCH_2), 68.1 (OCH_2), 68.1 (OCH_2), 114.8 (2 x Ar*C*), 114.8 (2 x Ar*C*), 114.9 (2 x Ar*C*), 118.0 (2 x Ar*C*), 121.1 (2 x Ar*C*), 121.6 (2 x Ar*C*), 122.1 (2 x Ar*C*), 123.4 (q, J = 273.4, 2 x CF_3), 131.7 (q, J = 33.1, 2 x Ar*C*), 132.3 (Ar*C*), 132.8 (3 x Ar*C*), 141.2 (Ar*C*), 155.0 (Ar*C*), 155.1 (Ar*C*), 155.4 (Ar*C*), 156.4 (*C*O), 157.0 (*C*O), 157.0 (*C*O), 158.9 (*C*O). **^{19}F NMR** (377 MHz, $CDCl_3$) δ_F −62.7 (2 x CF_3). **HR-MS** (ESI, positive ion mode) – *m/z* for $[C_{50}H_{65}F_6N_9O_7+H]^+$ = 1018.4984. Found 1018.4950.

1-(3,5-Bis(trifluoromethyl)anilinylcarbonyl)-13-Benzyl-4,7,10-tris(4-*n*-butyloxyanilinylcarbonyl)-1,4,7,10,13-pentaazatridecane, 1-3

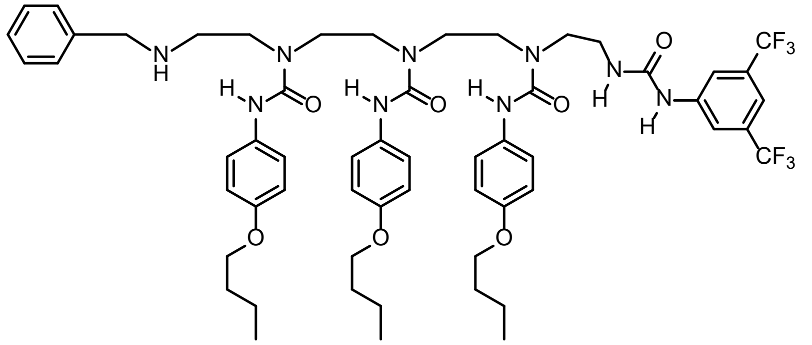

To a solution of **1-2** (642.8 mg, 0.63 mmol, 1.0 equiv) in lab grade THF (3.2 mL) was added a solution of benzaldehyde (67.0 mg, 0.63 mmol, 1.0 equiv) in lab grade MeOH (3.2 mL) and the mixture was stirred under air at room temperature for 20.5 h. The mixture was cooled to 0 °C and solid $NaBH_4$ (47.8 mg, 1.26 mmol, 2.0 equiv) was added, then the mixture was allowed to warm to room temperature in the cold bath with stirring over 3.5 h. 1 M K_2CO_3 (10 mL) was added and most of the organic solvents were removed *in vacuo*. Water (5 mL) was added and the product was extracted with CH_2Cl_2 (40 mL + 25 mL) then the combined organic extracts were dried (Na_2SO_4) and concentrated. Flash chromatography (Biotage, 25 g Sfär Duo column, MeOH/CH_2Cl_2 gradient from 0:100 to 10:90) gave the title compound (573.7 mg, 82%) as a white solid. **TLC** – R_f = 0.43 (SiO_2, 10:90 MeOH:CH_2Cl_2). **^{1}H NMR** (500 MHz, $CDCl_3$) δ_H 0.94 (t, J = 7.4, 3H, CH_3), 0.97 (t, J = 7.4, 3H, CH_3), 0.98 (t, J = 7.4, 3H, CH_3), 1.38-1.52 (m, 6H, 3 x CH_2CH_3), 1.64-1.77 (m, 6H, 3 x $CH_2CH_2CH_3$), 1.90 (s, 1H, N*H*), 2.89 (t, J = 4.0, 2H, NCH_2), 3.34-3.45 (m, 6H, 3 x NCH_2), 3.46-3.57 (m, 8H, 4 x NCH_2), 3.75 (t, J = 6.2, 2H, NCH_2), 3.83 (s, 2H, CH_2Ar), 3.86-3.92 (m, 4H, 2 x OCH_2), 6.73 (d, J = 8.9, 2H, 2 x Ar*H*), 6.76 (d, J = 9.0, 2H, 2 x Ar*H*), 6.78 (d, J = 9.0, 2H, 2 x Ar*H*), 6.93 (s, 1H, N*H*), 7.13 (d, J = 8.9, 2H, 2 x Ar*H*), 7.26 (d, J = 9.0, 2H, 2 x Ar*H*),

7.29-7.34 (m, 3H, 3 x Ar*H*), 7.37 (s, 1H, Ar*H*), 7.45 (d, J = 9.0, 2H, 2 x Ar*H*), 7.53 (d, J = 9.0, 2H, 2 x Ar*H*), 7.61 (s, 2H, 2 x Ar*H*), 8.39 (s, 1H, N*H*), 9.04 (s, 1H, N*H*), 9.13 (s, 1H, N*H*), 10.12 (s, 1H, N*H*). **^{13}C NMR** (126 MHz, $CDCl_3$) δ_C 13.9 (CH_3), 14.0 (2 x CH_3), 19.3 (CH_2CH_3), 19.4 (2 x CH_2CH_3), 31.4 ($CH_2CH_2CH_3$), 31.5 (2 x $CH_2CH_2CH_3$), 39.1 (NCH_2), 47.0 (2 x NCH_2), 48.4 (3 x NCH_2), 49.7 (NCH_2), 51.2 (NCH_2), 54.4 (CH_2Ar), 67.9 (OCH_3), 68.1 (OCH_3), 68.1 (OCH_3), 114.8 (6 x Ar*C*), 118.1 (2 x Ar*C*), 121.3 (2 x Ar*C*), 121.6 (2 x Ar*C*), 122.2 (2 x Ar*C*), 123.5 (q, J = 274.0, 2 x CF_3), 127.8 (Ar*C*), 128.5 (2 x Ar*C*), 128.9 (2 x Ar*C*), 131.7 (q, J = 33.0, 2 x Ar*C*), 132.2 (2 x Ar*C*), 132.6 (Ar*C*), 132.8 (Ar*C*), 138.7 (Ar*C*), 141.2 (Ar*C*), 155.0 (Ar*C*), 155.1 (Ar*C*), 155.4 (Ar*C*), 156.4 (*C*O), 157.0 (*C*O), 157.1 (*C*O), 158.8 (*C*O). **^{19}F NMR** (377 MHz, $CDCl_3$) δ_F −63.2 (2 x CF_3). **HR-MS** (ESI, positive ion mode) – *m/z* for $[C_{57}H_{71}F_6N_9O_7+H]^+$ = 1108.5453. Found 1108.5441.

1-Benzyl-1-(3,5-bis(trifluoromethyl)anilinylthiocarbonyl)-4,7,10-tris(4-*n*-butyloxyanilinylcarbonyl)-13-(3,5-bis(trifluoromethyl)anilinylcarbonyl)-1,4,7,10,13-pentaazatridecane, 1

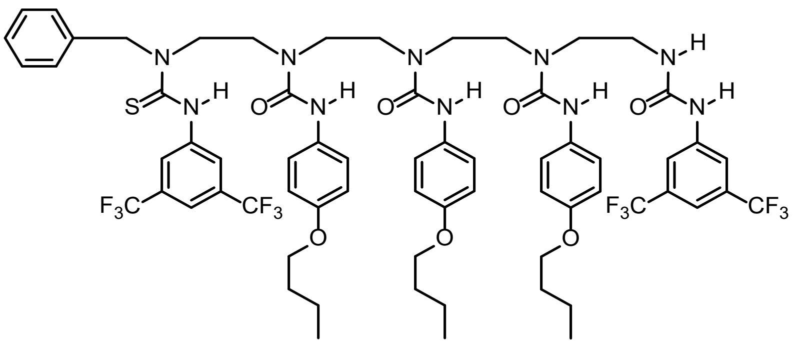

To a suspension of **1-3** (110.8 mg, 0.10 mmol, 1.0 equiv) in lab grade CH_2Cl_2 (2.0 mL) at 0 °C under air was added a solution of 3,5-bis(trifluoromethyl)phenyl isothiocyanate (27.1 mg, 0.10 mmol, 1.0 equiv) in CH_2Cl_2 (1.0 mL) and the mixture was allowed to warm to room temperature in the cold bath with stirring over 1.5 h. MeOH (1 mL) was added and the mixture was stirred for a further 2 min before being concentrated *in vacuo*. Flash chromatography (Biotage, 5 g Sfär Duo column, MeOH/CH_2Cl_2 gradient from 0:100 to 6:94) gave the title compound (132.9 mg, 96%) as a white solid. **TLC** – R_f = 0.46 (SiO_2, 5:95 MeOH:CH_2Cl_2). **^{1}H NMR** (500 MHz, CD_2Cl_2) δ_H 0.93-0.98 (m, 9H, 3 x CH_3), 1.41-1.49 (m, 6H, 3 x CH_2CH_3), 1.68-1.75 (m, 6H, 3 x $CH_2CH_2CH_3$), 3.23-3.29 (m, 2H, NCH_2), 3.39-3.53 (m, 12H, 6 x NCH_2), 3.72-3.83 (m, 2H, NCH_2), 3.83-3.91 (m, 6H, 3 x OCH_2), 5.33 (s, 2H, CH_2Ar), 5.62 (s, 1H, N*H*), 6.74-6.80 (m, 6H, 6 x Ar*H*), 7.31-7.37 (m, 3H, 3 x Ar*H*), 7.38-7.43 (m, 2H, 2 x Ar*H*), 7.45-7.52 (m, 7H, 7 x Ar*H*), 7.65 (s, 1H, Ar*H*), 7.83 (s, 2H, 2 x Ar*H*), 8.45 (s, 2H, 2 x Ar*H*), 8.55 (s, 1H, N*H*), 8.94 (s, 1H, N*H*), 9.17 (s, 1H, N*H*), 10.83 (s, 1H, N*H*). **^{13}C NMR** (126 MHz, CD_2Cl_2) δ_C 14.0 (3 x CH_3), 19.6 (3 x CH_2CH_3), 31.7 ($CH_2CH_2CH_3$), 31.8 (2 x $CH_2CH_2CH_3$), 40.2 (NCH_2), 47.6-49.0 (7 x NCH_2), 56.4 (CH_2Ar), 68.0 (OCH_3), 68.1 (OCH_3), 68.1 (OCH_3), 114.9 (6 x Ar*C*), 116.1 (Ar*C*), 118.6 (2 x Ar*C*), 121.6 (4 x Ar*C*), 122.1 (2 x Ar*C*), 123.8 (q, J = 271.6, 4 x CF_3), 124.7 (2 x Ar*C*) 124.8 (Ar*C*), 127.0 (Ar*C*), 127.5 (2 x Ar*C*), 128.2 (Ar*C*), 129.3 (2 x Ar*C*), 132.2 (q, J = 33.1, 4 x Ar*C*), 132.8 (3 x Ar*C*),

137.2 (Ar*C*), 141.0 (Ar*C*), 142.9 (Ar*C*), 155.6 (3 x Ar*C*), 156.5 (*C*O), 157.3 (*C*O), 157.4 (2 x *C*O), 182.0 (*C*S). **^{19}F NMR** (377 MHz, CD_2Cl_2) δ_F −63.3 (2 x CF_3), −63.1 (2 x CF_3). **HR-MS** (ESI, positive ion mode) – *m/z* for $[C_{66}H_{74}F_{12}N_{10}O_7S+Na]^+$ = 1401.5163. Found 1401.5156.

1-Benzyl-1-(dimethylaminocarbonyl)-4,7,10-tris(4-*n*-butyloxyanilinylcarbonyl)-13-(3,5-bis(trifluoromethyl)anilinylthiocarbonyl)-1,4,7,10,13-pentaazatridecane, **2**

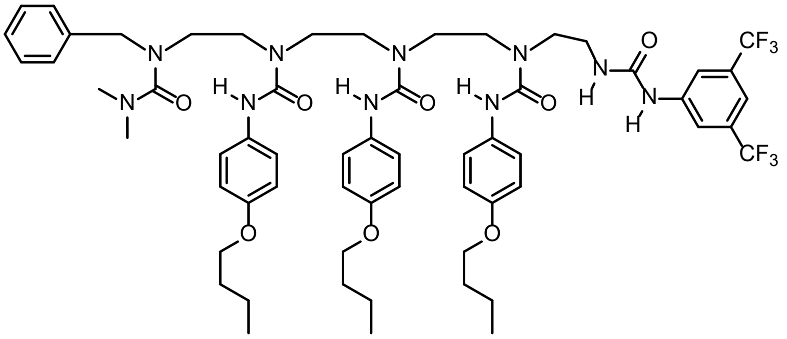

To neat **1-3** (76.1 mg, 0.069 mmol, 1.0 equiv) was added a solution of dimethylcarbamoyl chloride (8.9 mg, 0.082 mmol, 1.2 equiv) and Et_3N (10.4 mg, 0.10 mmol, 1.5 equiv) in lab grade 1,2-DCE (0.7 mL). The vial (sealed with cap) was placed in a sand bath at 45 °C and stirred at this temperature under air for 22 h, before being concentrated *in vacuo*. Flash chromatography (Biotage, 5 g Sfär Duo column, MeOH/CH_2Cl_2 gradient from 0:100 to 7:93) gave the title compound (78.8 mg, 97%) as a white solid. **TLC** – R_f = 0.43 (SiO_2, 5:95 MeOH:CH_2Cl_2). **^{1}H NMR** (500 MHz, CD_2Cl_2) δ_H 0.94 (t, J = 7.6, 3H, C*H*3), 0.97 (t, J = 7.4, 3H, C*H*3), 0.98 (t, J = 7.3, 3H, C*H*3), 1.38-1.53 (m, 6H, 3 x C*H*2CH3), 1.63-1.78 (m, 6H, 3 x OCH2C*H*2), 2.87 (s, 6H, 2 x NC*H*3), 3.26 (t, J = 7.0, 2H, NC*H*2), 3.33-3.53 (m, 14H, 7 x NC*H*2), 3.77 (t, J = 6.2, 2H, OC*H*2), 3.90 (t, J = 6.6, 2H, OC*H*2), 3.93 (t, J = 6.6, 2H, OC*H*2), 4.42 (s, 2H, C*H*2Ar), 6.73 (d, J = 8.6, 2H, 2 x Ar*H*), 6.79 (d, J = 8.8, 2H, 2 x Ar*H*), 6.84 (d, J = 8.9, 2H, 2 x Ar*H*), 7.25-7.32 (m, 2H, 2 x Ar*H*), 7.37 (s, 1H, Ar*H*), 7.37-7.41 (m, 3H, 3 x Ar*H*), 7.46 (d, J = 7.7, 2H, 2 x Ar*H*), 7.53 (d, J = 8.9, 2H, 2 x Ar*H*), 7.59 (d, J = 8.7, 2H, 2 x Ar*H*), 7.68 (s, 2H, 2 x Ar*H*), 8.42 (s, 1H, N*H*), 9.10 (s, 1H, N*H*), 9.17 (s, 1H, N*H*), 9.24 (s, 1H, N*H*). **^{13}C NMR** (126 MHz, CD_2Cl_2) δ_C 14.0 (*C*H3), 14.1 (2 x *C*H3), 19.6 (*C*H2CH3), 19.6 (*C*H2CH3), 19.7 (*C*H2CH3), 31.7 (OCH2*C*H2), 31.8 (2 x OCH2*C*H2), 38.9 (2 x N*C*H3), 39.6 (N*C*H2), 47.1 (N*C*H2), 47.3 (N*C*H2), 47.5 (N*C*H2), 47.6 (N*C*H2), 48.5 (N*C*H2), 48.6 (N*C*H2), 49.2 (N*C*H2), 54.5 (*C*H2Ar), 68.2 (O*C*H2), 68.3 (O*C*H2), 68.4 (O*C*H2), 114.8 (2 x Ar*C*), 114.9 (2 x Ar*C*), 114.9 (2 x Ar*C*), 118.2 (Ar*C*), 120.5 (2 x Ar*C*), 121.8 (2 x Ar*C*), 122.3 (2 x Ar*C*), 123.9 (q, J = 272.3, 2 x *C*F3), 127.0 (2 x Ar*C*), 127.9 (Ar*C*), 129.3 (2 x Ar*C*), 131.8 (q, J = 32.4, 2 x Ar*C*), 132.9 (Ar*C*), 133.4 (2 x Ar*C*), 133.5 (2 x Ar*C*), 137.6 (Ar*C*), 142.1 (Ar*C*), 155.3 (Ar*C*), 155.3 (Ar*C*), 155.6 (Ar*C*), 156.2 (*C*O), 157.3 (*C*O), 157.3 (*C*O), 157.4 (*C*O), 165.9 (*C*O). **^{19}F NMR** (377 MHz, CD_2Cl_2) δ_F −63.2 (2 x C*F*3). **HR-MS** (ESI, positive ion mode) – *m/z* for $[C_{60}H_{76}F_6N_{10}O_8+Na]^+$ = 1201.5644. Found 1201.5666.

N-(3,5-Bis(trifluoromethyl)phenyl)-N'-butyl urea, 3

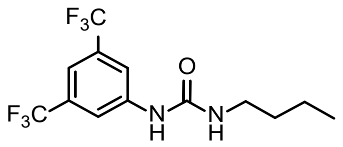

To a solution of $BuNH_2$ (36.6 mg, 0.50 mmol, 1.0 equiv) in lab grade CH_2Cl_2 (3.0 mL) at 0 °C under air was added a solution of 3,5-bis(trifluoromethyl)phenyl isocyanate (127.6 mg, 0.50 mmol, 1.0 equiv) in CH_2Cl_2 (2.0 mL) and the resulting suspension was allowed to warm in the cold bath with stirring over 1 h. MeOH (~1 mL) was added and the mixture was stirred for a further 20 min before being concentrated *in vacuo*. The resulting solid was re-dissolved in CH_2Cl_2 (5 mL) by briefly warming at 40 °C on a rotary evaporator (atmospheric pressure to avoid significant loss of solvent). The resulting solution was placed in a freezer overnight. Petroleum ether (~5 mL) was added to promote further precipitation and the mixture was placed in the freezer again overnight. The resulting precipitate was collected by gravity filtration, washed with petroleum ether (~10 mL) and dried to give the title compound (127.6 mg, 78%) as a white solid. **TLC** – R_f = 0.52 (SiO_2, 2.5:97.5 MeOH:CH_2Cl_2). Spectroscopic data matched that previously reported.[2]

1,4-Dibenzyl-1-(4-methoxyanilinylcarbonyl)-1,4-diazabutane, 4-1

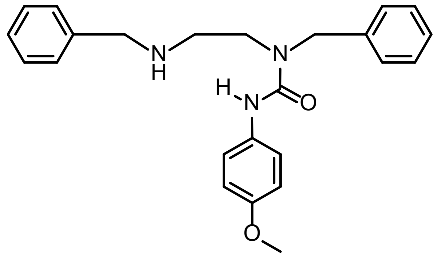

To a solution of *N*,*N'*-dibenzylethylenediamine (480.7 mg, 2.00 mmol, 2.0 equiv) in lab grade CH_2Cl_2 (5 mL) at room temperature under air was added a solution of 4-methoxyphenyl isocyanate (149.1 mg, 1.00 mmol, 1.0 equiv) in CH_2Cl_2 (5 mL) and the mixture was stirred for 3 h, before being concentrated *in vacuo*. Flash chromatography (Biotage, 10 g Sfär Duo column, MeOH/CH_2Cl_2 gradient from 0:100 to 10:90), gave the desired product containing a minor higher-R_f impurity (presumably the corresponding bis-urea). To this was added ~0.3 M HCl (50 mL) and the mixture was briefly sonicated to promote protonation of the immiscible product. EtOAc (40 mL) was added and the phases were separated. The organic phase was further extracted with ~0.3 M HCl (15 mL) and the combined aqueous extracts were brought to pH >10 by the addition of solid NaOH. The product was extracted with CH_2Cl_2 (40 mL + 20 mL), dried (Na_2SO_4) and concentrated to give an initial portion of the pure title compound (179.9 mg). TLC analysis showed significant product (freebase) still present in the original EtOAc organic phase. Therefore, 1 M HCl (30 mL) was added and the biphasic solution was stirred vigorously at room temperature under air for 22 h, resulting in a homogenous solution (presumably due to hydrolysis of EtOAc). The solvents were removed *in vacuo*. The residue was taken up in water (75 mL) and washed with EtOAc (50 mL), then the aqueous layer was brought to pH >10 by the addition of solid NaOH. The product was extracted with CH_2Cl_2 (40 mL + 20 mL), dried (Na_2SO_4) and concentrated to give a second portion of the pure title compound (198.4 mg; total yield = 378.3 mg, 97%) as a colourless gum that solidified upon storage in a freezer. **TLC** –

R_f = 0.55 (SiO_2, 7.5:92.5 MeOH:CH_2Cl_2). **^{1}H NMR** (400 MHz, $CDCl_3$) δ_H 2.74-2.77 (m, 2H, NC*H*$_2$), 3.38-3.41 (m, 2H, NC*H*$_2$), 3.80 (s, 3H, OC*H*$_3$), 3.83 (s, 2H, C*H*$_2$Ar), 4.59 (s, 2H, C*H*$_2$Ar), 6.81 (d, J = 9.0, 2H, 2 x Ar*H*), 7.22 (d, J = 9.0, 2H, 2 x Ar*H*), 7.26-7.38 (m, 10H, 10 x Ar*H*), 9.63 (s, 1H, N*H*). **^{13}C NMR** (101 MHz, $CDCl_3$) δ_C 48.8 (N*C*H$_2$), 48.9 (N*C*H$_2$), 51.2 (*C*H$_2$Ar), 54.3 (*C*H$_2$Ar), 55.7 (O*C*H$_3$), 114.1 (2 x Ar*C*), 121.1 (2 x Ar*C*), 127.4 (Ar*C*), 127.6 (Ar*C*), 128.1 (2 x Ar*C*), 128.4 (2 x Ar*C*), 128.7 (2 x Ar*C*), 128.8 (2 x Ar*C*), 133.6 (Ar*C*), 138.7 (Ar*C*), 139.0 (Ar*C*), 155.1 (Ar*C*), 158.0 (*C*O). **HR-MS** (ESI, positive ion mode) – *m/z* for $[C_{24}H_{27}N_3O_2+H]^+$ = 390.2176. Found 390.2180.

1,4-Dibenzyl-1-(3,5-bis(trifluoromethyl)anilinylthiocarbonyl)-4-(4-methoxyanilinylcarbonyl)-1,4-diazabutane, 4

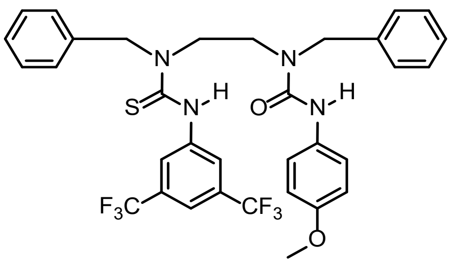

To a solution of **4-1** (58.4 mg, 0.15 mmol, 1.0 equiv) in lab grade CH_2Cl_2 (0.5 mL) at 0 °C under air was added a solution of 3,5-bis(trifluoromethyl)phenyl isothiocyanate (40.7 mg, 0.15 mmol, 1.0 equiv) in CH_2Cl_2 (1.0 mL) and the mixture was allowed to warm in the cold bath with stirring over 30 min. The solution was concentrated *in vacuo* to give the title compound (101.0 mg, >99%) as a white solid (*note:* residual CH_2Cl_2 accounted for the 2% extra mass beyond the theoretical yield of 99.1 mg). **TLC** – R_f = 0.83 (SiO_2, 2.5:97.5 MeOH:CH_2Cl_2). **^{1}H NMR** (500 MHz, CD_2Cl_2) δ_H 3.42-3.48 (m, 2H, NC*H*$_2$), 3.71 (t, J = 7.5, 2H, NC*H*$_2$), 3.74 (s, 3H, OC*H*$_3$), 4.46 (s, 2H, C*H*$_2$Ar), 5.20 (s, 2H, C*H*$_2$Ar), 6.45 (s, 1H, N*H*), 6.78 (d, J = 8.9, 2H, 2 x Ar*H*), 7.16 (d, J = 8.9, 2H, 2 x Ar*H*), 7.24 (d, J = 7.4, 2H, 2 x Ar*H*), 7.29-7.43 (m, 8H, 8 x Ar*H*), 7.63 (s, 1H, Ar*H*), 8.43 (s, 2H, 2 x Ar*H*), 10.65 (s, 1H, N*H*). **^{13}C NMR** (126 MHz, CD_2Cl_2) δ_C 48.1 (2 x N*C*H$_2$), 55.9 (O*C*H$_3$), 56.5 (*C*H$_2$Ar), 114.5 (2 x Ar*C*), 117.8 (Ar*C*), 122.6 (2 x Ar*C*), 124.1 (q, J = 271.4, 2 x *C*F$_3$), 124.8 (2 x Ar*C*), 127.3 (2 x Ar*C*), 128.3 (2 x Ar*C*), 128.4 (Ar*C*), 128.9 (Ar*C*), 129.3 (2 x Ar*C*), 129.9 (2 x Ar*C*), 131.3 (q, J = 33.2, 2 x Ar*C*), 131.5 (Ar*C*), 136.6 (Ar*C*), 137.4 (Ar*C*), 143.2 (Ar*C*), 156.8 (Ar*C*), 157.3 (*C*O), 181.9 (*C*S). **^{19}F NMR** (377 MHz, CD_2Cl_2) δ_F −63.0 (2 x C*F*$_3$). **HR-MS** (ESI, positive ion mode) – *m/z* for $[C_{33}H_{30}F_6N_4O_2S+Na]^+$ = 683.1886. Found 683.1868.

1,4-Dibenzyl-1-(dimethylaminocarbonyl)-1,4-diazabutane, 5-1

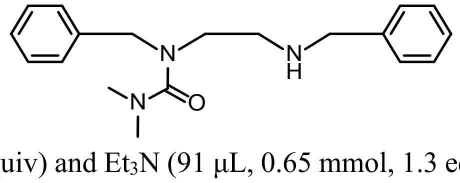

To neat *N,N'*-dibenzylethylenediamine (240.4 mg, 1.00 mmol, 2.0 equiv) was added a solution of dimethylcarbamoyl chloride (53.8 mg, 0.50 mmol, 1.0 equiv) and Et_3N (91 μL, 0.65 mmol, 1.3 equiv) in lab grade 1,2-DCE (2.0 mL). The vial (with cap) was placed in a sand bath at 45 °C and stirred at this temperature under air for 30 h. 1 M K_2CO_3 (20 mL)

was added and the product was extracted with CH_2Cl_2 (30 mL + 20 mL) then the combined organic extracts were dried (Na_2SO_4) and concentrated. To remove the di-urea side product, the residue was dissolved in Et_2O (10 mL) and 1 M aqueous HCl (5 mL) and water (10 mL) were added. The biphase was stirred at room temperature for 20 min, then diluted with Et_2O (25 mL) and 1 M aqueous HCl (3 mL) and water (20 mL). The organic phase was separated. The aqueous phase was basified (to pH > 12) with NaOH pellets, then the product was extracted with CH_2Cl_2 (30 mL + 20 mL). The combined organic extracts were dried (Na_2SO_4) and concentrated. Flash chromatography (Biotage, 10 g Sfär Duo column, MeOH/CH_2Cl_2 gradient from 0:100 to 6.5:93.5) gave the title compound (62.7 mg, 40% based on carbamoyl chloride) as a pale-yellow oil. **TLC** – R_f = 0.30 (SiO_2, 7.5:92.5 MeOH:CH_2Cl_2). **^{1}H NMR** (500 MHz, $CDCl_3$) δ_H 2.79 (t, J = 6.4, 2H, NCH_2), 2.87 (s, 6H, 2 x NCH_3), 3.26 (t, J = 6.4, 2H, NCH_2), 3.77 (s, 2H, CH_2Ar), 4.40 (s, 2H, CH_2Ar), 7.22-7.35 (m, 10H, 10 x Ar*H*). **^{13}C NMR** (126 MHz, $CDCl_3$) δ_C 38.8 (2 x NCH_3), 46.7 (NCH_2), 47.5 (NCH_2), 52.1 (CH_2Ar), 53.8 (CH_2Ar), 127.0 (Ar*C*), 127.2 (Ar*C*), 127.5 (2 x Ar*C*), 128.1 (2 x Ar*C*), 128.4 (2 x Ar*C*), 128.6 (2 x Ar*C*), 138.2 (Ar*C*), 140.3 (Ar*C*), 165.6 (*C*O). **HR-MS** (ESI, positive ion mode) – *m/z* for $[C_{19}H_{25}N_3O+H]^+$ = 312.2070. Found 312.2085.

1,4-Dibenzyl-1-(dimethylaminocarbonyl)-4-(4-*n*-butyloxyanilinylcarbonyl)-1,4-diazabutane, **5**

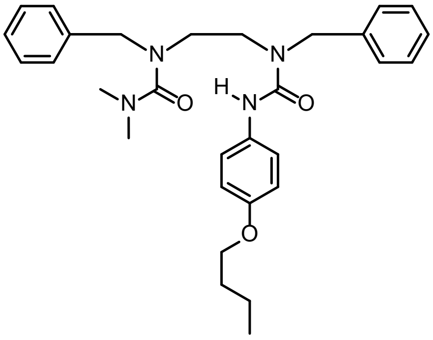

To a solution of **5-1** (42.4 mg, 0.14 mmol, 1.0 equiv) in lab grade CH_2Cl_2 (0.4 mL) at room temperature under air was added a solution of 4-butoxyphenyl isocyanate (26.0 mg, 0.14 mmol, 1.0 equiv) in CH_2Cl_2 (1.0 mL) [note that the isocyanate solution was delivered into the reaction vial by filtration through a cotton pipette plug to remove a trace amount of an insoluble urea impurity]. After stirring for 50 min, MeOH (~1 mL) was added and the mixture was stirred for 5 min to quench any unreacted isocyanate, before being concentrated *in vacuo*. Flash chromatography (Biotage, 5 g Sfär Duo column, MeOH/CH_2Cl_2 gradient from 0:100 to 2.5:97.5) gave the title compound (65.0 mg, 95%) as a white solid. **TLC** – R_f = 0.46 (SiO_2, 2.5:97.5 MeOH:CH_2Cl_2). **^{1}H NMR** (500 MHz, CD_2Cl_2) δ_H 0.98 (t, J = 7.4, 3H, CH_3), 1.49 (dq, J = 7.6, 7.6, 2H, CH_2CH$_3$), 1.71-1.77 (m, 2H, OCH$_2$CH_2), 2.84 (s, 6H, 2 x NCH_3), 3.00 (t, J = 7.4, 2H, NCH_2), 3.26 (t, J = 7.4, 2H, NCH_2), 3.94 (t, J = 6.5, 2H, OCH_2), 4.28 (s, 2H, CH_2Ar), 4.44 (s, 2H, CH_2Ar), 6.82 (d, J = 8.9, 2H, 2 x Ar*H*), 7.15-7.35 (m, 10H, 10 x Ar*H*), 7.56 (d, J = 8.9, 2H, 2 x Ar*H*), 8.78 (s, 1H, N*H*). **^{13}C NMR** (126 MHz, CD_2Cl_2) δ_C 14.2 (CH_3), 19.8 (CH_2CH_3), 32.0 (OCH$_2$$CH_2$), 39.1 (2 x N$CH_3$), 45.0 (N$CH_2$), 47.1 (N$CH_2$), 51.2 ($CH_2$Ar), 54.7 ($CH_2$Ar), 68.5 (O$CH_2$), 114.8 (2 x Ar*C*), 121.4 (2 x Ar*C*), 127.3 (2 x Ar*C*), 127.5 (Ar*C*), 127.9 (Ar*C*), 128.2 (2 x Ar*C*), 128.8 (2 x Ar*C*), 129.2 (2 x Ar*C*), 134.3 (Ar*C*), 137.7 (Ar*C*), 139.5 (Ar*C*), 154.9 (Ar*C*), 156.2 (*C*O), 165.8 (*C*O). **HR-MS** (ESI, positive ion mode) – *m/z* for $[C_{30}H_{38}N_4O_3+H]^+$ = 503.3017. Found 503.3019.

1,13-Dibenzyl-4,7,10-tris(4-*n*-butyloxyanilinylcarbonyl)-1,4,7,10,13-pentaazatridecane, 6-1

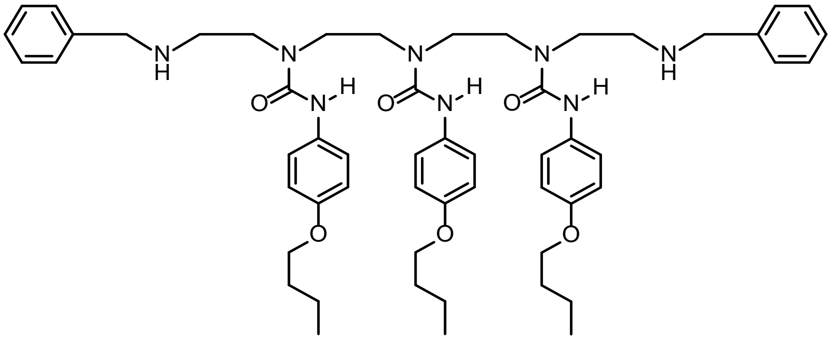

To a solution of **1-1** (201.3 mg, 0.26 mmol, 1.0 equiv) in lab grade MeOH (1.3 mL) was added a solution of benzaldehyde (56.0 mg, 0.53 mmol, 2.0 equiv) in MeOH (1.3 mL) and the mixture was stirred under air at room temperature for 17 h. To the cloudy mixture was added 10 drops of THF via a pipette, then the mixture was cooled to 0 °C and solid $NaBH_4$ (39.9 mg, 1.06 mmol, 4.0 equiv) was added. The mixture was allowed to warm to room temperature in the cold bath with stirring over 3 h. 1 M K_2CO_3 (10 mL) was added and the product was extracted with CH_2Cl_2 (25 mL + 15 mL) then the combined organic extracts were dried (Na_2SO_4) and concentrated. Flash chromatography (Biotage, 5 g Sfär Duo column, MeOH/CH_2Cl_2 gradient from 0:100 to 10:90) gave the title compound (185.6 mg, 75%) as a white foam. **TLC** – R_f = 0.38 (SiO_2, 10:90 MeOH:CH_2Cl_2). **^{1}H NMR** (500 MHz, $CDCl_3$) δ_H 1.03 (t, J = 7.4, 9H, 3 x C*H*$_3$), 1.50-1.58 (m, 6H, 3 x C*H*$_2$CH$_3$), 1.76-1.82 (m, 6H, 3 x C*H*$_2$CH$_2$CH$_3$), 2.00 (s, 2H, 2 x N*H*), 2.86-2.90 (m, 4H, 2 x NC*H*$_2$), 3.39-3.47 (m, 8H, 4 x NC*H*$_2$), 3.48-3.55 (m, 4H, 2 x NC*H*$_2$), 3.83 (s, 4H, 2 x C*H*$_2$Ar), 3.96 (t, J = 6.5, 6H, 3 x OC*H*$_2$), 6.81 (d, J = 8.9, 4H, 4 x Ar*H*), 6.86 (d, J = 9.0, 2H, 2 x Ar*H*), 7.22-7.38 (m, 14H, 14 x Ar*H*), 7.64 (d, J = 8.9, 2H, 2 x Ar*H*), 9.03 (s, 1H, N*H*), 9.91 (s, 2H, 2 x N*H*). **^{13}C NMR** (126 MHz, $CDCl_3$) δ_C 13.9 (3 x *C*H$_3$), 19.2 (3 x *C*H$_2$CH$_3$), 31.4 (3 x *C*H$_2$CH$_2$CH$_3$), 46.8 (2 x N*C*H$_2$), 47.6 (2 x N*C*H$_2$), 49.4 (2 x N*C*H$_2$), 50.4 (2 x N*C*H$_2$), 54.1 (2 x *C*H$_2$Ar), 67.9 (O*C*H$_2$), 68.0 (2 x O*C*H$_2$), 114.6 (2 x Ar*C*), 114.7 (4 x Ar*C*), 120.9 (4 x Ar*C*), 121.0 (2 x Ar*C*), 127.4 (2 x Ar*C*), 128.3 (4 x Ar*C*), 128.6 (4 x Ar*C*), 133.1 (2 x Ar*C*), 133.4 (Ar*C*), 139.0 (2 x Ar*C*), 154.5 (3 x Ar*C*), 156.2 (*C*O), 157.9 (2 x *C*O). **HR-MS** (ESI, positive ion mode) – *m/z* for $[C_{55}H_{74}N_8O_6+H]^+$ = 943.5804. Found 943.5782.

1,13-Dibenzyl-1-(dimethylaminocarbonyl)-4,7,10-tris(4-*n*-butyloxyanilinylcarbonyl)-1,4,7,10,13-pentaazatridecane, **6-2**

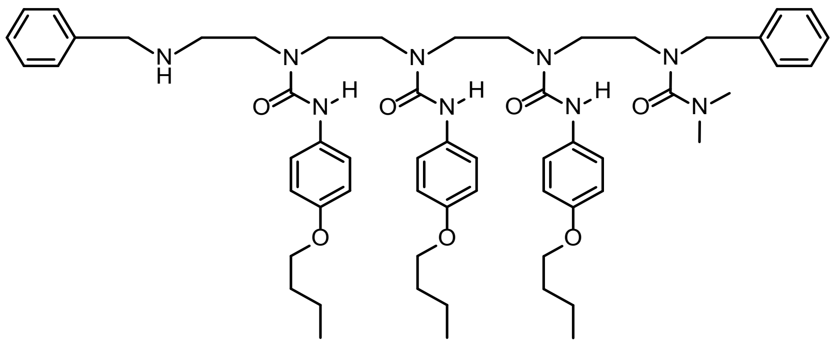

To neat **6-1** (219.8 mg, 0.23 mmol, 2.0 equiv) was added a solution of dimethylcarbamoyl chloride (12.5 mg, 0.12 mmol, 1.0 equiv) in lab grade 1,2-DCE (1.2 mL) followed by 3 drops of Et_3N from a 21-gauge needle (~15 mg, 0.15 mmol, 1.3 equiv). The vial (with cap) was placed in a pre-heated sand bath at 45 °C and stirred at this temperature under air for 24 h. 1 M K_2CO_3 (20 mL) was added and the product was extracted with CH_2Cl_2 (30 mL + 20 mL) then the combined organic extracts were dried (Na_2SO_4) and concentrated. Flash chromatography (Biotage, 10 g Sfär Duo column, MeOH/CH_2Cl_2 gradient from 0:100 to 10:90) gave the title compound (67.8 mg, 57% based on carbamoyl chloride) as a white foam. Further elution from the chromatography column returned unreacted **6-1** (95.2 mg, 43% based on total diamine used). *Data for **6-2**:* **TLC** – R_f = 0.36 (SiO_2, 7.5:92.5 MeOH:CH_2Cl_2). **^{1}H NMR** (500 MHz, $CDCl_3$) δ_H 0.98 (t, J = 7.3, 9H, 3 x C*H*$_3$), 1.46-1.55 (m, 6H, 3 x C*H*$_2$CH$_3$), 1.73-1.80 (m, 6H, 3 x C*H*$_2$CH$_2$CH$_3$), 2.04 (s, 1H, N*H*), 2.87 (s, 6H, 2 x NC*H*$_3$), 2.89 (t, J = 4.6, 2H, NC*H*$_2$), 3.23 (t, J = 7.4, 2H, NC*H*$_2$), 3.35-3.49 (m, 12H, 6 x NC*H*$_2$), 3.83 (s, 2H, C*H*$_2$Ar), 3.92-3.97 (m, 6H, 3 x OC*H*$_2$), 4.41 (s, 2H, C*H*$_2$Ar), 6.78 (d, J = 8.8, 2H, 2 x Ar*H*), 6.81 (d, J = 8.9, 2H, 2 x Ar*H*), 6.85 (d, J = 9.0, 2H, 2 x Ar*H*), 7.23 (d, J = 8.8, 2H, 2 x Ar*H*), 7.26-7.38 (m, 10H, 10 x Ar*H*), 7.56 (d, J = 8.9, 2H, 2 x Ar*H*), 7.60 (d, J = 9.0, 2H, 2 x Ar*H*), 8.91 (s, 1H, N*H*), 8.99 (s, 1H, N*H*), 9.77 (s, 1H, N*H*). **^{13}C NMR** (126 MHz, $CDCl_3$) δ_C 14.0 (3 x *C*H$_3$), 19.4 (3 x *C*H$_2$CH$_3$), 31.5 (2 x *C*H$_2$CH$_2$CH$_3$), 31.6 (*C*H$_2$CH$_2$CH$_3$), 38.9 (2 x N*C*H$_3$), 46.5 (N*C*H$_2$), 46.9 (N*C*H$_2$), 47.0 (N*C*H$_2$), 47.4 (N*C*H$_2$), 47.8 (N*C*H$_2$), 49.5 (2 x N*C*H$_2$), 50.4 (N*C*H$_2$), 54.1 (*C*H$_2$Ar), 54.3 (*C*H$_2$Ar), 68.1 (3 x O*C*H$_2$), 114.7 (2 x Ar*C*), 114.8 (4 x Ar*C*), 121.0 (2 x Ar*C*), 121.1 (4 x Ar*C*), 126.9 (2 x Ar*C*), 127.6 (2 x Ar*C*), 127.7 (Ar*C*), 128.4 (2 x Ar*C*), 128.8 (2 x Ar*C*), 129.1 (Ar*C*), 133.2 (Ar*C*), 133.5 (2 x Ar*C*), 137.1 (Ar*C*), 138.9 (Ar*C*), 154.7 (3 x Ar*C*), 156.3 (*C*O), 156.4 (*C*O), 158.0 (*C*O), 165.5 (*C*O). **HR-MS** (ESI, positive ion mode) – *m/z* for $[C_{58}H_{79}N_9O_7+H]^+$ = 1014.6175. Found 1014.6159.

1,13-Dibenzyl-1-(dimethylaminocarbonyl)-13-(3,5-bis(trifluoromethyl)anilinylthiocarbonyl)-4,7,10-tris(4-*n*-butyloxyanilinylcarbonyl)-1,4,7,10,13-pentaazatridecane, **6**

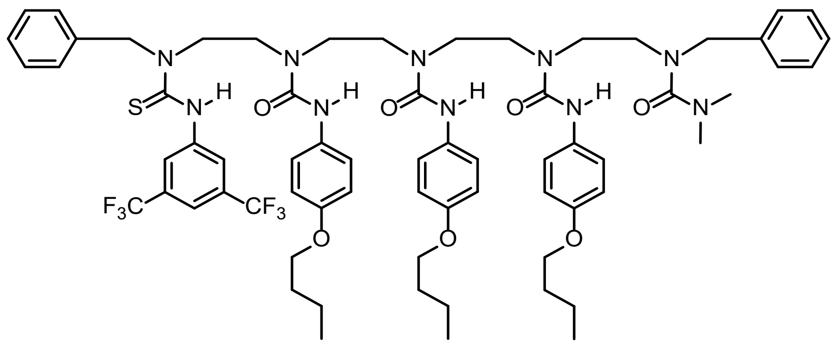

To a solution of **6-2** (32.6 mg, 0.032 mmol, 1.0 equiv) in lab grade CH_2Cl_2 (0.5 mL) at 0 °C under air was added a solution of 3,5-bis(trifluoromethyl)phenyl isothiocyanate (8.7 mg, 0.032 mmol, 1.0 equiv) in CH_2Cl_2 (0.5 mL) and the mixture was allowed to warm to room temperature in the cold bath with stirring over 2.5 h. MeOH (0.5 mL) was added and the mixture was stirred for a further 10 min before being concentrated *in vacuo*. Flash chromatography (Biotage, 5 g Sfär Duo column, MeOH/CH_2Cl_2 gradient from 0:100 to 4:96) gave the title compound (38.2 mg, 92%) as a white solid. **TLC** – R_f = 0.35 (SiO_2, 2.5:97.5 MeOH:CH_2Cl_2). **^{1}H NMR** (500 MHz, CD_2Cl_2) δ_H 0.96-1.01 (m, 9H, 3 x C*H*$_3$), 1.46-1.54 (m, 6H, 3 x C*H*$_2$CH$_3$), 1.72-1.80 (m, 6H, 3 x C*H*$_2$CH$_2$CH$_3$), 2.88 (s, 6H, 2 x NC*H*$_3$), 3.22-3.45 (m, 14H, 7 x NC*H*$_2$), 3.70 (t, J = 7.3, 2H, NC*H*$_2$), 3.91-3.98 (m, 6H, 3 x OC*H*$_2$), 4.41 (s, 2H, C*H*$_2$Ar), 5.33 (s, 2H, C*H*$_2$Ar), 6.81 (d, J = 8.9, 2H, 2 x Ar*H*), 6.82 (d, J = 9.0, 2H, 2 x Ar*H*), 6.87 (d, J = 9.0, 2H, 2 x Ar*H*), 7.28-7.44 (m, 10H, 10 x Ar*H*), 7.57 (d, J = 8.9, 2H, 2 x Ar*H*), 7.61 (d, J = 9.0, 2H, 2 x Ar*H*), 7.63 (d, J = 9.0, 2H, 2 x Ar*H*), 7.65 (s, 1H, Ar*H*), 8.55 (s, 2H, 2 x Ar*H*), 9.13 (s, 1H, N*H*), 9.28 (s, 1H, N*H*), 9.35 (s, 1H, N*H*), 10.93 (s, 1H, N*H*). **^{13}C NMR** (126 MHz, CD_2Cl_2) δ_C 14.2 (3 x *C*H$_3$), 19.8 (3 x *C*H$_2$CH$_3$), 31.9 (*C*H$_2$CH$_2$CH$_3$), 32.0 (2 x *C*H$_2$CH$_2$CH$_3$), 39.0 (2 x N*C*H$_3$), 47.6 (N*C*H$_2$), 47.7 (N*C*H$_2$), 47.9 (2 x N*C*H$_2$), 48.3 (N*C*H$_2$), 48.5 (N*C*H$_2$), 49.1 (N*C*H$_2$), 49.7 (N*C*H$_2$), 54.6 (*C*H$_2$Ar), 56.6 (*C*H$_2$Ar), 68.0 (3 x O*C*H$_2$), 115.0 (6 x Ar*C*), 117.6 (Ar*C*), 121.4 (2 x Ar*C*), 121.6 (2 x Ar*C*), 121.7 (2 x Ar*C*), 124.1 (q, J = 273.1, 2 x *C*F$_3$), 124.7 (2 x Ar*C*), 127.0 (2 x Ar*C*), 127.9 (2 x Ar*C*), 128.0 (Ar*C*), 128.1 (Ar*C*), 129.2 (2 x Ar*C*), 129.5 (2 x Ar*C*), 131.3 (q, J = 33.2, 2 x Ar*C*), 133.4 (Ar*C*), 133.5 (Ar*C*), 133.6 (Ar*C*), 137.7 (Ar*C*), 137.8 (Ar*C*), 143.5 (Ar*C*), 155.4 (Ar*C*), 155.5 (2 x Ar*C*), 157.2 (*C*O), 157.3 (*C*O), 157.4 (*C*O), 166.0 (*C*O), 182.1 (*C*S). **^{19}F NMR** (377 MHz, CD_2Cl_2) δ_F −63.0 (2 x C*F*$_3$). **HR-MS** (ESI, positive ion mode) – *m/z* for $[C_{67}H_{82}F_6N_{10}O_7S+Na]^+$ = 1307.5885. Found 1307.5868.

1,16-Bis(trifluoroacetyl)-1,4,7,10,13,16-hexaazahexadecane, **7-1**

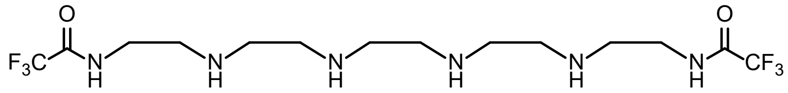

To a solution of pentaethylenehexamine (2.324 g, 10.00 mmol, 1.0 equiv) in lab grade MeOH (200 mL) under N_2 at −78 °C (liquid N_2/EtOAc cold bath) was added a solution of ethyl trifluoroacetate (2.842 g, 20.00 mmol, 2.0 equiv) in MeOH (5 mL) and the mixture was allowed to warm to room temperature in the cold bath with stirring over 19 h, before being concentrated *in vacuo*. Flash chromatography (Biotage, 25 g Sfär Duo column, MeOH/[35% aqueous NH_3]/CH_2Cl_2 gradient from 0:0:100 to 10:2:88) gave the title compound (1.135 g, 27%) as a pale yellow oil containing several minor impurities. The ^1H NMR spectrum of the major product was consistent with that reported previously.[3] This material was used in the next step without further purification. **TLC** – R_f = 0.08 (SiO_2, 15:3:82 MeOH/[35% aqueous NH_3]/CH_2Cl_2, ninhydrin stain).

4,7,10,13-Tetrakis(4-*n*-butyloxyanilinylcarbonyl)-1,4,7,10,13,16-hexaazahexadecane, **7-2**

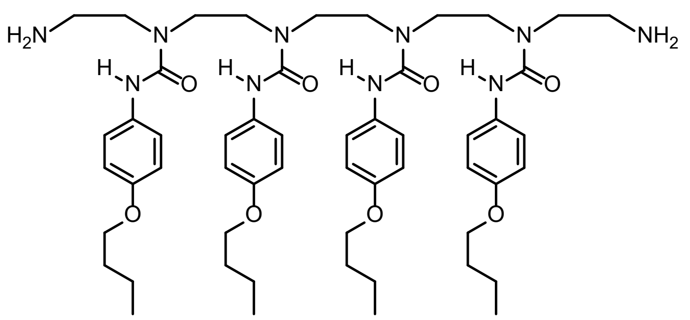

Step 1 *(tetrakis-urea formation):* To a solution of **7-1** (1.135 g, 2.67 mmol, 1.0 equiv) in lab grade CH_2Cl_2 (21.7 mL) at 0 °C under air was added over 5 min a solution of 4-butoxyphenyl isocyanate (2.13 mL, 11.77 mmol, 4.4 equiv) in CH_2Cl_2 (5.0 mL) [note that the isocyanate solution was delivered into the reaction flask by filtration through a cotton pipette plug to remove a small amount of an insoluble urea impurity]. After complete addition, the ice bath was removed and the mixture was stirred at room temperature for 20 min. MeOH (~3 mL) was added and the mixture was stirred for 15 min to quench any unreacted isocyanate. After standing at room temperature for 1.5 h, the suspension was gravity filtered to remove an unknown precipitate, and the filter cake was rinsed with further CH_2Cl_2 (~10 mL). The filtrate was concentrated *in vacuo*. To the solid residue was added 1:1 Et_2O/petroleum ether (100 mL) and the suspension was placed inside a sonicator for 10 min, before being gravity filtered to collect the undissolved product. The flask and filter cake were rinsed/washed with further 1:1 Et_2O/petroleum ether (70 mL) and the cake was air dried to give the crude intermediate tetrakis-urea (2.481 g, 78%) as a pale yellow solid. TLC – Rf = 0.39 (SiO_2, 5:95 MeOH/CH_2Cl_2). ***Step 2*** *(trifluoroacetamide hydrolysis):* The product from *Step 1* (2.481 g, 2.09 mmol, 1.0 equiv) was suspended in a mixture of lab grade THF (10.4 mL), EtOH (20.9 mL) and DMF (20 drops from a Pasteur pipette). A solution of NaOH (667.5 mg, 16.69 mmol, 8.0 equiv) in water (10.4 mL) was added and the solution was stirred at room temperature under air for 2 h. Most of the solvents were removed *in vacuo*, then water (20 mL) was added. The product was extracted with CH_2Cl_2 (50 mL + 30 mL) then the

combined organic extracts were dried (Na_2SO_4) and concentrated. Two iterative rounds of flash chromatography (Biotage, 25 g Sfär Duo column (first purification), 50 g Sfär Duo column (second purification), MeOH/[35% aqueous NH_3]/CH_2Cl_2 gradient from 0:0:100 to 10:2:88 for both purifications) gave the title compound (895.7 mg, 43% or 34% over two steps) as a white foamy solid. **TLC** – R_f = 0.13 (SiO_2, 10:2:88 MeOH/[35% aqueous NH_3]/CH_2Cl_2). **^{1}H NMR** (500 MHz, $CDCl_3$) δ_H 0.96 (t, J = 7.4, 6H, 2 x C*H*$_3$), 0.97 (t, J = 7.3, 6H, 2 x C*H*$_3$), 1.41-1.52 (m, 8H, 4 x C*H*$_2$CH$_3$), 1.67-1.78 (m, 8H, 4 x OCH$_2$C*H*$_2$), 2.87 (t, J = 4.5, 4H, 2 x NC*H*$_2$), 3.30-3.51 (m, 16H, 8 x NC*H*$_2$), 3.91 (t, J = 6.4, 4H, 2 x OC*H*$_2$), 3.92 (t, J = 6.4, 4H, 2 x OC*H*$_2$), 6.80 (d, J = 8.9, 4H, 4 x Ar*H*), 6.81 (d, J = 9.0, 4H, 4 x Ar*H*), 7.30 (d, J = 8.0, 4H, 4 x Ar*H*), 7.60 (d, J = 8.9, 4H, 4 x Ar*H*), 9.09 (s, 2H, 2 x N*H*), 10.06 (s, 2H, 2 x N*H*). **^{13}C NMR** (126 MHz, $CDCl_3$) δ_C 13.9 (2 x *C*H$_3$), 13.9 (2 x *C*H$_3$), 19.3 (4 x *C*H$_2$CH$_3$), 31.4 (2 x OCH$_2$*C*H$_2$), 31.5 (2 x OCH$_2$*C*H$_2$), 41.8 (2 x N*C*H$_2$), 47.1 (2 x N*C*H$_2$), 47.9 (4 x N*C*H$_2$), 52.7 (2 x N*C*H$_2$), 68.0 (2 x O*C*H$_2$), 68.1 (2 x O*C*H$_2$), 114.7 (4 x Ar*C*), 114.8 (4 x Ar*C*), 120.7 (4 x Ar*C*), 121.1 (4 x Ar*C*), 133.2 (2 x Ar*C*), 133.4 (2 x Ar*C*), 154.6 (2 x Ar*C*), 154.7 (2 x Ar*C*), 156.4 (2 x *C*O), 158.4 (2 x *C*O). **HR-MS** (ESI, positive ion mode) – *m/z* for $[C_{54}H_{80}N_{10}O_8+H]^+$ = 997.6233. Found 997.6218.

1-(3,5-Bis(trifluoromethyl)anilinylcarbonyl)-4,7,10,13-tetrakis(4-*n*-butyloxyanilinylcarbonyl)-1,4,7,10,13,16-hexaazahexadecane, **7-3**

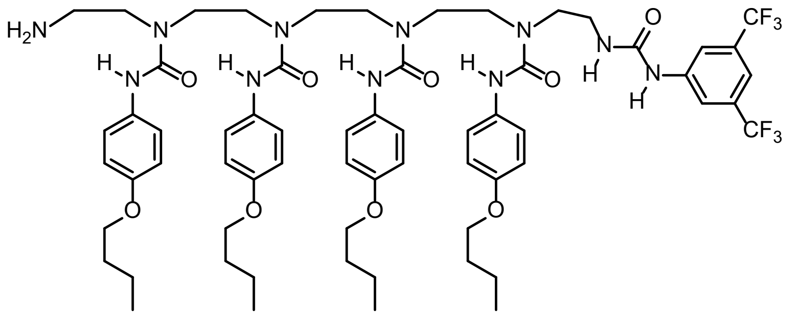

To a solution of **7-2** (434.2 mg, 0.44 mmol, 1.0 equiv) in lab grade CH_2Cl_2 (6.2 mL) at −10 °C (ice/salt bath) under N_2 was added dropwise over 70 min a solution of 3,5-bis(trifluoromethyl)phenyl isocyanate (111.1 mg, 0.44 mmol, 1.0 equiv) in CH_2Cl_2 (2.5 mL) while maintaining the cold bath temperature between −10 °C and 0 °C. After complete addition, the cold bath was allowed to warm to room temperature and the mixture was stirred for a further 1 h, before being concentrated *in vacuo*. Flash chromatography (Biotage, 25 g Sfär Duo column, MeOH/[35% aqueous NH_3]/CH_2Cl_2 gradient from 0:0:100 to 10:2:88) gave the title compound (286.3 mg, 53%) as a white solid. [Further elution from the chromatography column returned unreacted **7-2** (71.3 mg, 16% recovered)]. *Data for 7-3:* **TLC** – R_f = 0.38 (SiO_2, 10:2:88 MeOH/[35% aqueous NH_3]/CH_2Cl_2). **^{1}H NMR** (500 MHz, $CDCl_3$) δ_H 0.92-0.99 (m, 12H, 4 x C*H*$_3$), 1.38-1.51 (m, 8H, 4 x C*H*$_2$CH$_3$), 1.62-1.77 (m, 8H, 4 x OCH$_2$C*H*$_2$), 2.91-2.96 (m, 2H, NC*H*$_2$), 3.31-3.59 (m, 18H, 9 x NC*H*$_2$), 3.72-3.79 (m, 2H, OC*H*$_2$), 3.87-3.94 (m, 6H, 3 x OC*H*$_2$), 6.73 (d, J = 8.6, 2H, 2 x Ar*H*), 6.78-6.84 (m, 6H, 6 x Ar*H*), 6.97 (s, 1H, N*H*), 7.29 (d, J = 8.8, 2H, 2 x Ar*H*), 7.37 (s, 1H, Ar*H*), 7.47 (d, J = 8.6, 2H, 2 x Ar*H*), 7.54-7.65 (m, 6H, 6 x Ar*H*), 8.35 (s, 1H, N*H*), 9.21 (s, 1H, N*H*), 9.28 (s, 2H, 2 x

N*H*), 10.25 (s, 1H, N*H*). **^{13}C NMR** (126 MHz, $CDCl_3$) δ_C 13.9 (CH_3), 14.0 (3 x CH_3), 19.3 (CH_2CH_3), 19.4 (3 x CH_2CH_3), 31.4 (OCH_2CH_2), 31.5 (3 x OCH_2CH_2), 39.2 (NCH_2), 42.0 (2 x NCH_2), 47.2 (2 x NCH_2), 47.7 (NCH_2), 48.3 (NCH_2), 48.5 (NCH_2), 48.8 (NCH_2), 53.1 (NCH_2), 67.9 (OCH_2), 68.1 (OCH_2), 68.1 (OCH_2), 68.2 (OCH_2), 114.8 (2 x Ar*C*), 114.8 (2 x Ar*C*), 114.9 (2 x Ar*C*), 115.0 (2 x Ar*C*), 118.2 (Ar*C*), 120.9 (2 x Ar*C*), 121.1 (2 x Ar*C*), 121.6 (2 x Ar*C*), 122.0 (2 x Ar*C*), 123.4 (q, J = 274.2, 2 x CF_3), 131.8 (q, J = 32.9, 2 x Ar*C*), 132.3 (2 x Ar*C*), 132.9 (Ar*C*), 132.9 (2 x Ar*C*), 133.0 (Ar*C*), 141.2 (Ar*C*), 155.0 (Ar*C*), 155.0 (Ar*C*), 155.1 (Ar*C*), 155.4 (Ar*C*), 156.5 (*C*O), 156.9 (*C*O), 157.0 (2 x *C*O), 158.9 (*C*O). **^{19}F NMR** (377 MHz, $CDCl_3$) δ_F −62.8 (2 x CF_3). **HR-MS** (ESI, positive ion mode) – *m/z* for $[C_{63}H_{83}F_6N_{11}O_9+H]^+$ = 1252.6352. Found 1252.6357.

1-Benzyl-4,7,10,13,16-pentakis(4-*n*-butyloxyanilinylcarbonyl)-19-(3,5-bis(trifluoromethyl)anilinylcarbonyl)-1,4,7,10,13,16,19-heptaazanonadecane, **7-4**

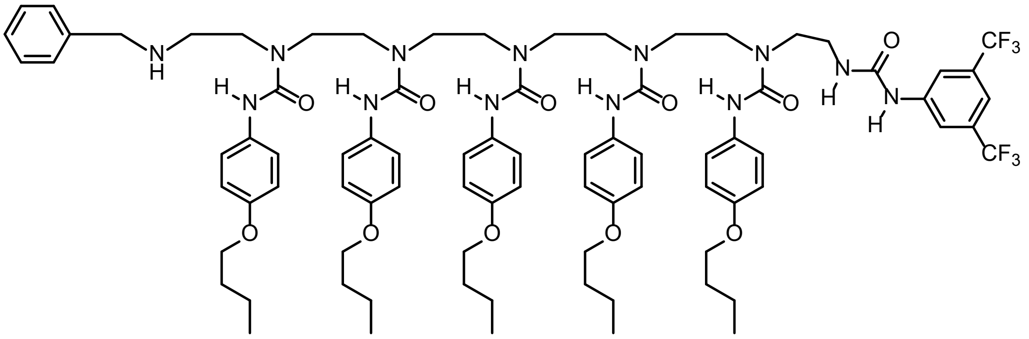

Step 1 (*reductive amination*): To a solution of **7-3** (494.4 mg, 0.39 mmol, 1.0 equiv) in lab grade THF (2.0 mL) was added a solution of *N*-benzyl-*N*-Boc-2-aminoacetaldehyde[1] (98.4 mg, 0.39 mmol, 1.0 equiv) in lab grade MeOH (2.0 mL) and the mixture was stirred under air at room temperature for 19 h. The mixture was cooled to 0 °C and solid $NaBH_4$ (30.0 mg, 0.79 mmol, 2.0 equiv) was added and the cold bath was allowed to warm to room temperature with stirring over 3 h. 1 M K_2CO_3 (10 mL) was added and most organic solvent was removed *in vacuo*. The mixture was diluted with water (10 mL) and the product was extracted with CH_2Cl_2 (2 × 20 mL) then the combined organic extracts were dried (Na_2SO_4) and concentrated. Flash chromatography (Biotage, 25 g Sfär Duo column, MeOH/CH_2Cl_2 gradient from 0:100 to 8:92) gave the product (358.7 mg, 61%) as a white solid. TLC – R_f = 0.34 (7.5:92.5 MeOH/CH_2Cl_2). **Step 2** (*urea formation*): The product from *Step 1* (358.7 mg, 0.24 mmol, 1.0 equiv) was dissolved in lab grade CH_2Cl_2 (1.0 mL) and a solution of 4-butoxyphenyl isocyanate (46.2 mg, 0.24 mmol, 1.0 equiv) in CH_2Cl_2 (1.4 mL) was added [note that the isocyanate solution was delivered into the reaction flask by filtration through a cotton pipette plug to remove a small amount of an insoluble urea impurity]. The mixture was stirred at room temperature under air for 1.5 h. MeOH (~0.5 mL) was added and the mixture was stirred for 5 min to quench any unreacted isocyanate, before being concentrated *in vacuo*. Flash chromatography (Biotage, 25 g Sfär Duo column, MeOH/CH_2Cl_2 gradient from 0:100 to 3.5:96.5) gave the product (360.4 mg, 89% or 54% over two steps) as a white

solid. **Step 3** (*Boc deprotection*): The product from *Step 2* (360.4 mg, 0.21 mmol, 1.0 equiv) was dissolved in lab grade CH_2Cl_2 (2.1 mL) and TFA (0.25 mL, 3.22 mmol, 15.0 equiv) was added. The mixture was stirred under air at room temperature for 27.5 h. After cooling to 0 °C, saturated $NaHCO_3$ (20 mL) was slowly added to the open flask with stirring. After complete addition, the cold bath was removed and the mixture was stirred for a further 5 min. After further dilution with saturated $NaHCO_3$ (10 mL), the product was extracted with CH_2Cl_2 (30 mL + 20 mL) and the combined organic extracts were dried (Na_2SO_4) and concentrated. Flash chromatography (Biotage, 25 g Sfär Duo column, MeOH/CH_2Cl_2 gradient from 0:100 to 7.5:92.5) gave the title compound (313.2 mg, 92% or 50% over three steps) as a white solid. **TLC** – R_f = 0.46 (SiO_2, 7.5:92.5 MeOH:CH_2Cl_2). **^{1}H NMR** (500 MHz, CD_2Cl_2) δ_H 0.93-1.00 (m, 15H, 5 x C*H*$_3$), 1.42-1.52 (m, 10H, 5 x C*H*$_2$CH$_3$), 1.66-1.77 (m, 10H, 5 x OCH$_2$C*H*$_2$), 2.84-2.88 (m, 2H, NC*H*$_2$), 3.31-3.58 (m, 22H, 11 x NC*H*$_2$), 3.79-3.86 (m, 4H, OC*H*$_2$, C*H*$_2$Ar), 3.87-3.95 (m, 6H, 3 x OC*H*$_2$), 5.33 (s, 1H, N*H*), 6.64 (s, 1H, N*H*), 6.73-6.85 (m, 10H, 10 x Ar*H*), 7.14 (d, J = 6.5, 2H, 2 x Ar*H*), 7.25-7.34 (m, 5H, 5 x Ar*H*), 7.41 (s, 1H, Ar*H*), 7.51 (d, J = 7.9, 2H, 2 x Ar*H*), 7.54-7.62 (m, 6H, 6 x Ar*H*), 7.75 (s, 2H, 2 x Ar*H*), 8.47 (s, 1H, N*H*), 9.00-9.35 (m, 4H, 4 x N*H*), 10.18 (s, 1H, N*H*). **^{13}C NMR** (126 MHz, CD_2Cl_2) δ_C 14.0 (*C*H$_3$), 14.0 (4 x *C*H$_3$), 19.6 (*C*H$_2$CH$_3$), 19.6 (4 x *C*H$_2$CH$_3$), 31.8 (OCH$_2$*C*H$_2$), 31.8 (4 x OCH$_2$*C*H$_2$), 39.8 (N*C*H$_2$), 47.5 (2 x N*C*H$_2$), 48.0 (2 x N*C*H$_2$), 48.7 (3 x N*C*H$_2$), 49.9 (N*C*H$_2$), 51.4 (N*C*H$_2$), 54.2 (N*C*H$_2$), 54.5 (*C*H$_2$Ar), 68.3 (O*C*H$_2$), 68.3 (O*C*H$_2$), 68.4 (3 x O*C*H$_2$), 114.9 (10 x Ar*C*), 118.2 (Ar*C*), 121.6 (2 x Ar*C*), 121.6 (6 x Ar*C*), 122.1 (2 x Ar*C*), 123.8 (q, J = 272.7, 2 x *C*F$_3$), 127.9 (Ar*C*), 128.7 (2 x Ar*C*), 129.0 (2 x Ar*C*), 131.9 (q, J = 32.6, 2 x Ar*C*), 133.0 (2 x Ar*C*), 133.1 (2 x Ar*C*), 133.3 (Ar*C*), 133.4 (Ar*C*), 133.4 (Ar*C*), 139.4 (Ar*C*), 141.9 (2 x Ar*C*), 155.3 (Ar*C*), 155.4 (2 x Ar*C*), 155.4 (Ar*C*), 155.6 (Ar*C*), 156.6 (*C*O), 157.2 (3 x *C*O), 157.3 (*C*O), 159.1 (*C*O). **^{19}F NMR** (377 MHz, CD_2Cl_2) δ_F −63.0 (2 x C*F*$_3$). **HR-MS** (Nanospray, positive ion mode) – *m/z* for $[C_{83}H_{107}F_6N_{13}O_{11}+H]^+$ = 1576.8195. Found 1576.8208.

1-Benzyl-1-(3,5-bis(trifluoromethyl)anilinylthiocarbonyl)-4,7,10,13,16-pentakis(4-*n*-butyloxyanilinylcarbonyl)-19-(3,5-bis(trifluoromethyl)anilinylcarbonyl)-1,4,7,10,13,16,19-heptaazanonadecane, 7

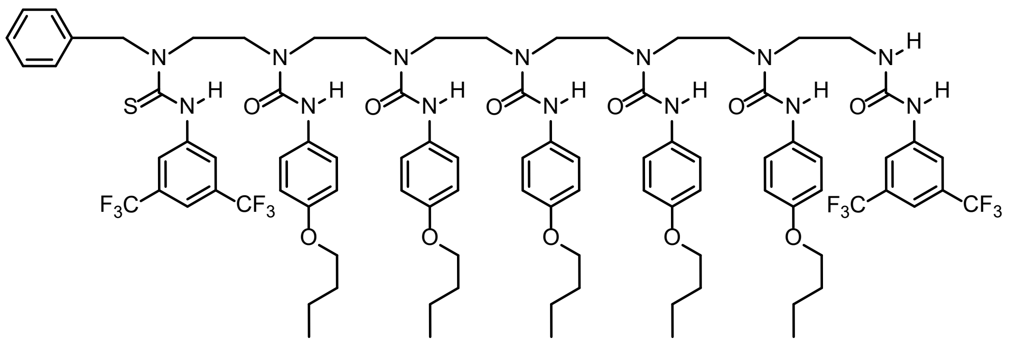

To neat **7-4** (63.1 mg, 0.040 mmol, 1.0 equiv) was added a solution of 3,5-bis(trifluoromethyl)phenyl isothiocyanate (10.8 mg, 0.040 mmol, 1.0 equiv) in lab grade CH_2Cl_2 (0.8 mL) and the mixture was stirred at room temperature under air for 2 h, before being concentrated *in vacuo*. Flash chromatography (Biotage, 5 g Sfär Duo column, MeOH/CH_2Cl_2 gradient from 0:100 to 5:95) gave the title compound (65.4 mg, 88%) as a white solid. **TLC** – R_f = 0.53 (SiO_2, 7.5:92.5 MeOH:CH_2Cl_2). **^{1}H NMR** (500 MHz, CD_2Cl_2) δ_H 0.93-0.99 (m, 15H, 5 x C*H*$_3$), 1.41-1.51 (m, 10H, 5 x C*H*$_2$CH$_3$), 1.67-1.76 (m, 10H, 5 x OCH$_2$C*H*$_2$), 3.09-3.17 (m, 2H, NC*H*$_2$), 3.28-3.33 (m, 2H, NC*H*$_2$), 3.35-3.57 (m, 18H, 9 x NC*H*$_2$), 3.70-3.79 (m, 2H, NC*H*$_2$), 3.84-3.94 (m, 10H, 5 x OC*H*$_2$), 5.23 (s, 2H, C*H*$_2$Ar), 5.60 (s, 1H, N*H*), 6.74-6.84 (m, 10H, 10 x Ar*H*), 7.27-7.42 (m, 5H, 5 x Ar*H*), 7.46-7.56 (m, 11H, 11 x Ar*H*), 7.64 (s, 1H, Ar*H*), 7.69 (s, 1H, N*H*), 7.82 (s, 2H, 2 x Ar*H*), 8.44 (s, 2H, 2 x Ar*H*), 8.59 (s, 1H, N*H*), 8.92 (s, 2H, 2 x N*H*), 9.06 (s, 1H, N*H*), 9.21 (s, 1H, N*H*), 10.84 (s, 1H, N*H*). **^{13}C NMR** (126 MHz, CD_2Cl_2) δ_C 14.0 (*C*H$_3$), 14.0 (4 x *C*H$_3$), 19.6 (*C*H$_2$CH$_3$), 19.6 (4 x *C*H$_2$CH$_3$), 31.7 (OCH$_2$*C*H$_2$), 31.8 (4 x OCH$_2$*C*H$_2$), 40.2 (N*C*H$_2$), 47.9 (3 x N*C*H$_2$), 48.2 (2 x N*C*H$_2$), 48.6 (4 x N*C*H$_2$), 49.0 (2 x N*C*H$_2$), 56.2 (*C*H$_2$Ar), 68.3 (3 x O*C*H$_2$), 68.4 (O*C*H$_2$), 68.5 (O*C*H$_2$), 114.8 (2 x Ar*C*), 114.9 (2 x Ar*C*), 114.9 (2 x Ar*C*), 115.0 (2 x Ar*C*), 115.0 (2 x Ar*C*), 115.9 (Ar*C*) 118.5 (Ar*C*), 121.4 (2 x Ar*C*), 122.0 (8 x Ar*C*), 123.8 (q, J = 272.8, 4 x *C*F$_3$), 127.6 (2 x Ar*C*), 128.2 (Ar*C*), 129.3 (2 x Ar*C*), 132.1 (q, J = 32.9, 4 x Ar*C*), 132.9 (4 x Ar*C*), 133.0 (3 x Ar*C*), 133.0 (2 x Ar*C*), 137.0 (Ar*C*), 141.0 (Ar*C*), 143.0 (Ar*C*), 155.6 (5 x Ar*C*), 156.5 (*C*O), 157.2 (*C*O), 157.3 (2 x *C*O), 157.4 (2 x *C*O), 181.8 (*C*S). **^{19}F NMR** (377 MHz, CD_2Cl_2) δ_F −63.1 (2 x C*F*$_3$), −63.3 (2 x C*F*$_3$). **HR-MS** (Nanospray, positive ion mode) – *m/z* for $[C_{92}H_{110}F_{12}N_{14}O_{11}S+H]^+$ = 1847.8086. Found 1847.8060.

4-Chloropyridinium tetrakis(3,5-bis(trifluoromethyl)phenyl)borate, **S8**

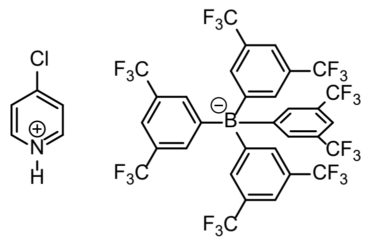

To a glass vial (not pre-dried) was added 4-chloropyridinium chloride (15.0 mg, 0.10 mmol) and sodium tetrakis-(3,5-bis(trifluoromethyl)phenyl)borate (88.6 mg, 0.10 mmol). Commercially obtained dry MeCN (1.5 mL) was added and the suspension was stirred at room temperature under air (capped vial) for 2 h. The suspension was diluted with CH_2Cl_2 (~2 mL) and stirred for a further 15 min before being filtered through a pipette cotton plug to remove the suspended solids. The reaction vial and cotton plug were further rinsed with CH_2Cl_2 (~3 mL), then the filtrate was concentrated, re-evaporated twice from CH_2Cl_2 (to fully remove MeCN) and dried under high vacuum to give the title compound (95.6 mg, 98%) as an easily handled off-white powder. **^{1}H NMR** (500 MHz, CD_2Cl_2) δ_H 7.56 (s, 4H, 4 x Ar*H*), 7.72 (pentet, J = 2.2, 8H, 8 x Ar*H*), 8.05 (d, J = 7.0, 2H, 2 x Ar*H*), 8.52 (d, J = 6.9, 2H, 2 x Ar*H*). **^{13}C NMR** (126 MHz, CD_2Cl_2) δ_C 118.0 (pentet, J = 4.0, 4 x Ar*C*), 125.1 (q, J = 272.3, 8 x *C*F_3), 129.4 (qdd, J = 31.5, 5.7, 2.9, 8 x Ar*C*), 129.7 (2 x Ar*C*), 135.3 (8 x Ar*C*), 142.0 129.7 (2 x Ar*C*), 158.8 (Ar*C*), 162.3 (q, J = 49.8, 4 x Ar*C*B).

***tert*-Butylamino-tris(dimethylamino)phosphonium tetrakis(3,5-bis(trifluoromethyl)phenyl)borate**, **S9**

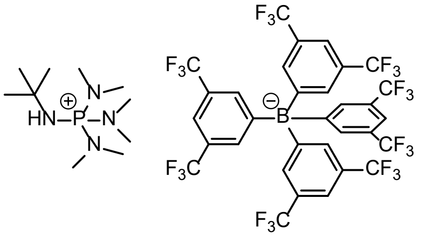

(No stirring was performed in this reaction.) To a solution of **S8** (50.0 mg, 0.051 mmol, 1.0 equiv) in reagent grade CH_2Cl_2 (1 mL) under air at room temperature was added a solution of phosphazene base P_1-*t*-Bu (12.6 mg, 0.054 mmol, 1.05 equiv) in CH_2Cl_2 (1 mL). The reaction flask was gently swirled by hand for 1 min, then the solvent was blown off using a stream of N_2. The residue was further dried *in vacuo* to give a pale yellow solid. A cold mixture of water/MeOH (1:1, 8 mL, pre-cooled in an ice bath) was added and the suspension was briefly sonicated (~30 seconds), then gravity filtered. The collected solid was washed with further cold water/MeOH (1:1, 2 mL) and dried *in vacuo* to give the title compound (22.6 mg, 40%) as a white powder. **^{1}H NMR** (500 MHz, CD_2Cl_2) δ_H 1.31 (s, 9H, C(C*H*$_3$)$_3$), 2.71 (d, 18H, J = 10.0, 6 x NC*H*$_3$), 7.57 (s, 4H, 4 x Ar*H*), 7.72 (s, 8H, 8 x Ar*H*). **^{13}C NMR** (126 MHz, CD_2Cl_2) δ_C 31.5 (d, J = 4.4, C(*C*H$_3$)$_3$), 37.9 (d, J = 4.6, 6 x N*C*H$_3$), 53.7 (m, *C*(CH$_3$)$_3$), 117.9 (m, 4 x Ar*C*), 125.0 (q, J = 270.9, 8 x *C*F$_3$), 129.3 (qq, J = 31.5, 2.9, 8 x Ar*C*), 135.2 (8 x Ar*C*), 162.1 (q, J = 50.6, 4 x Ar*C*B). **^{19}F NMR** (377 MHz, CD_2Cl_2) δ_F −62.9 (8 x C*F*$_3$). **^{31}P NMR** (162 MHz, CD_2Cl_2) δ_P 34.7 ([NR$_2$]$_4$*P*$^+$).

1-Benzyl-1-(3,5-bis(trifluoromethyl)anilinylthiocarbonyl)-4,7,10-tris(*n*-butylaminocarbonyl)-13-(3,5-bis(trifluoromethyl)anilinylcarbonyl)-1,4,7,10,13-pentaazatridecane, S10

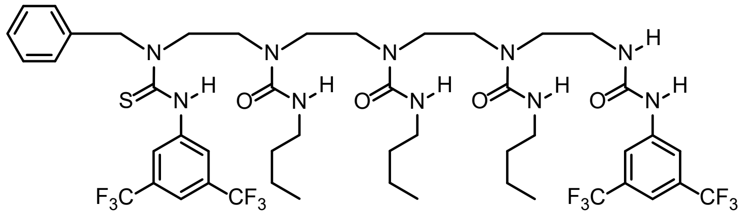

Step 1 (*reductive amination*): To neat **S10-1**[1] (78.2 mg, 0.11 mmol, 1.0 equiv) was added a solution of benzaldehyde (11.2 mg, 0.11 mmol, 1.0 equiv) in lab grade MeOH (1.1 mL) and the mixture was stirred under air at room temperature for 21 h. The mixture was cooled to 0 °C and solid $NaBH_4$ (8.0 mg, 0.21 mmol, 2.0 equiv) was added, then the mixture was allowed to warm to room temperature in the cold bath with stirring arbitrarily over 96 h. 1 M K_2CO_3 (6 mL) was added and the product was extracted with CH_2Cl_2 (25 mL + 15 mL) then the combined organic extracts were dried (Na_2SO_4) and concentrated. Flash chromatography (Biotage, 10 g Sfär Duo column, MeOH/CH_2Cl_2 gradient from 0:100 to 10:90) gave the product (38.6 mg, 44%) as a white solid. **TLC** – R_f = 0.15 (SiO_2, 10:90 MeOH:CH_2Cl_2). **Step 2** (*thiourea formation*): The product from *Step 1* (38.6 mg, 0.046 mmol, 1.0 equiv) was taken up in lab grade CH_2Cl_2 (0.4 mL) and a solution of 3,5-bis(trifluoromethyl)phenyl isothiocyanate (12.6 mg, 0.046 mmol, 1.0 equiv) in CH_2Cl_2 (1.0 mL) was added. The mixture was stirred at room temperature under air for 1 h, before being concentrated *in vacuo*. Flash chromatography (Biotage, 5 g Sfär Duo column, MeOH/CH_2Cl_2 gradient from 0:100 to 6:94) gave the product (48.4 mg, 95% or 42% over two steps) as a white solid. **TLC** – R_f = 0.29 (SiO_2, 5:95 MeOH:CH_2Cl_2). **^{1}H NMR** (500 MHz, CD_2Cl_2) δ_H 0.82-0.91 (m, 9H, 3 x C*H*$_3$), 1.24-1.37 (m, 6H, 3 x *CH$_2$*CH$_3$), 1.42-1.53 (m, 6H, 3 x NCH$_2$*CH$_2$*), 3.05-3.55 (m, 20H, 10 x NC*H*$_2$), 3.55-4.07 (m, 2H, NC*H*$_2$), 5.18 (s, 2H, C*H*$_2$Ar), 6.01 (s, 2H, 2 x N*H*), 6.56 (s, 2H, 2 x N*H*), 7.29-7.44 (m, 5H, 5 x Ar*H*), 7.47 (s, 1H, Ar*H*), 7.63 (s, 1H, Ar*H*), 7.93 (s, 2H, 2 x Ar*H*), 8.29 (s, 2H, 2 x Ar*H*), 10.93 (s, 1H, N*H*). **^{13}C NMR** (126 MHz, CD_2Cl_2) δ_C 14.0 (*C*H$_3$), 14.0 (*C*H$_3$), 14.1 (*C*H$_3$), 20.6 (*C*H$_2$CH$_3$), 20.6 (*C*H$_2$CH$_3$), 20.7 (*C*H$_2$CH$_3$), 32.5 (NCH$_2$*C*H$_2$), 32.6 (NCH$_2$*C*H$_2$), 32.7 (NCH$_2$*C*H$_2$), 40.0 (2 x N*C*H$_2$), 41.3 (2 x overlapping N*C*H$_2$), 41.3 (2 x overlapping N*C*H$_2$), 41.4 (overlapping N*C*H$_2$), 48.0 (2 x N*C*H$_2$), 48.1 (overlapping N*C*H$_2$), 48.8 (N*C*H$_2$), 56.2 (*C*H$_2$Ar), 115.7 (2 x Ar*C*), 118.3 (3 x Ar*C*), 124.0 (q, J = 272.6, 4 x *C*F$_3$), 125.3 (Ar*C*), 127.6 (2 x Ar*C*), 128.5 (Ar*C*), 129.5 (2 x Ar*C*), 131.5 (q, J = 33.6, 2 x Ar*C*), 132.4 (q, J = 33.1, 2 x Ar*C*), 137.1 (broad Ar*C*), 141.8 (Ar*C*), 142.9 (broad Ar*C*), 156.3 (*C*O), 159.4 (*C*O), 159.5 (2 x *C*O), 182.3 (*C*S). **^{19}F NMR** (377 MHz, CD_2Cl_2) δ_F −63.4 (2 x C*F*$_3$), −63.2 (2 x C*F*$_3$). **HR-MS** (ESI, positive ion mode) – *m/z* for $[C_{48}H_{62}F_{12}N_{10}O_4S+Na]^+$ = 1125.4377. Found 1125.4367.

Conformational Analysis

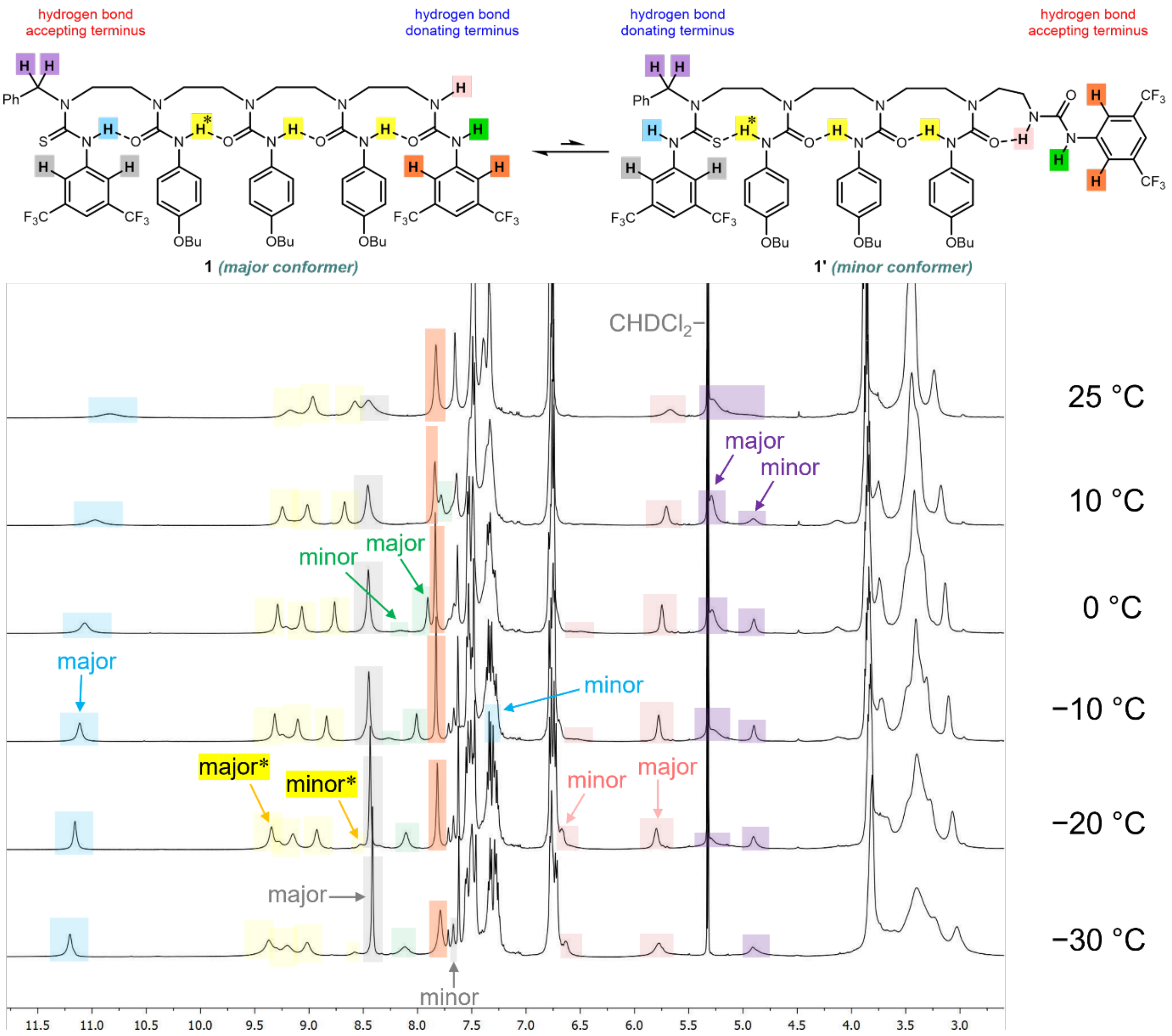

Figure S1 – Variable temperature ^{1}H NMR spectra of compound 1 (500 MHz, 27 mM, CD_2Cl_2). Two conformers are present, which differ in the directionality of the hydrogen bond chain. The major conformer **1** (75% at −10 ºC) has the thiourea at the hydrogen bond-accepting terminus, while the minor conformer **1'** (25% at −10 ºC) has the thiourea at the hydrogen bond-donating terminus. Selected pairs of rotationally exchanging protons are highlighted in different colours (except for the internal urea N–Hs, which are all coloured yellow), and where the protons in rotational exchange resonate at distinct chemical shifts, the relevant signals are labelled on the spectra as belonging to the major or minor conformer. These assignments were supported by NOESY (EXSY) and ROESY experiments (*vide infra*). The disappearance of the benzylic methylene signal at ~5.3 ppm (coloured purple) for the major conformer below −20 °C is attributed to the proximal sulfur atom slowing down rotation about the N−CH_2Ph bond at lower temperatures, causing signal broadening.

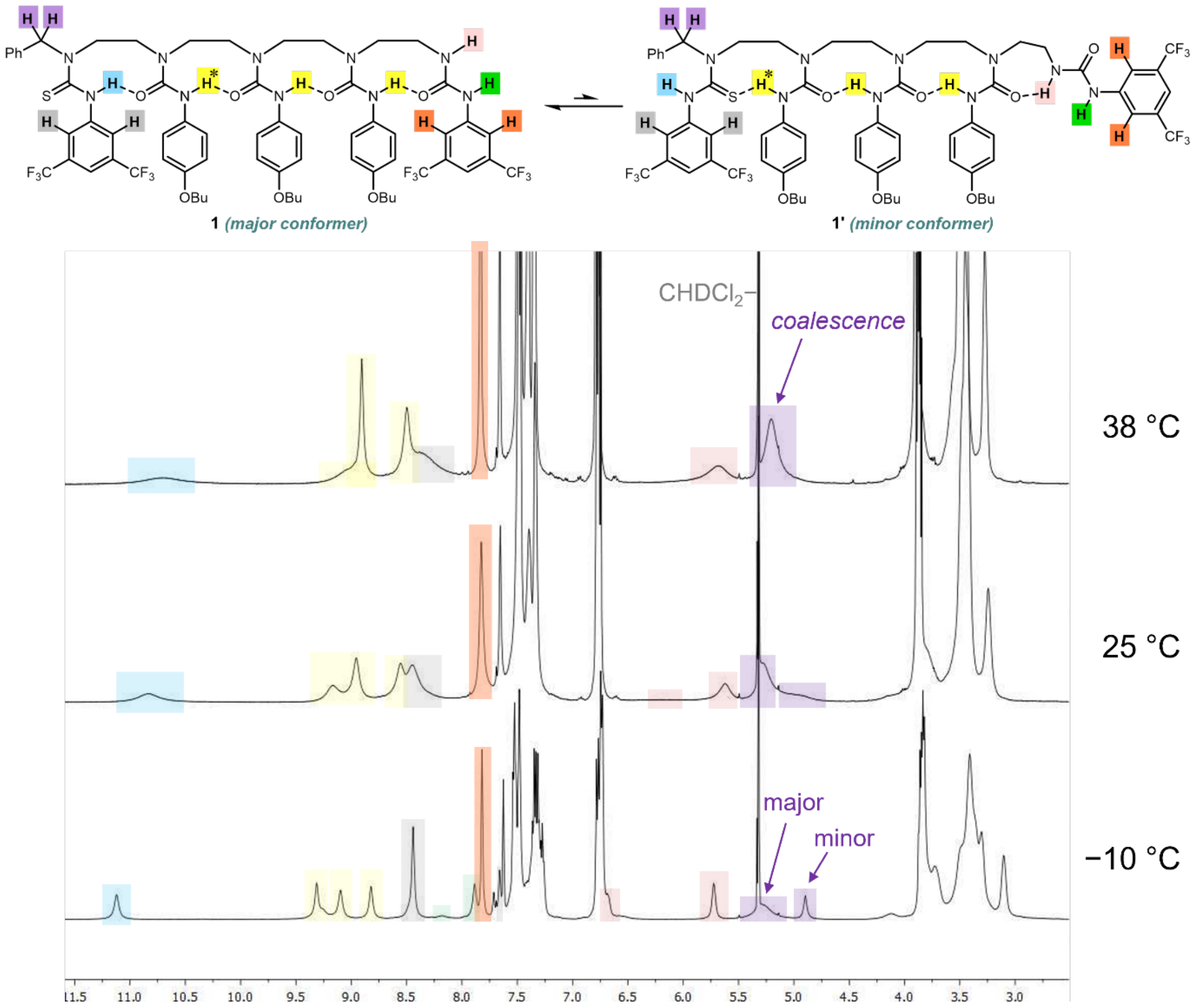

Figure S2 – Additional variable temperature ¹H NMR spectra of compound 1 (500 MHz, 25 mM, CD_2Cl_2). Coalescence of the signals from the benzylic methylene protons (coloured purple) of the two conformers is observed at 38 °C.

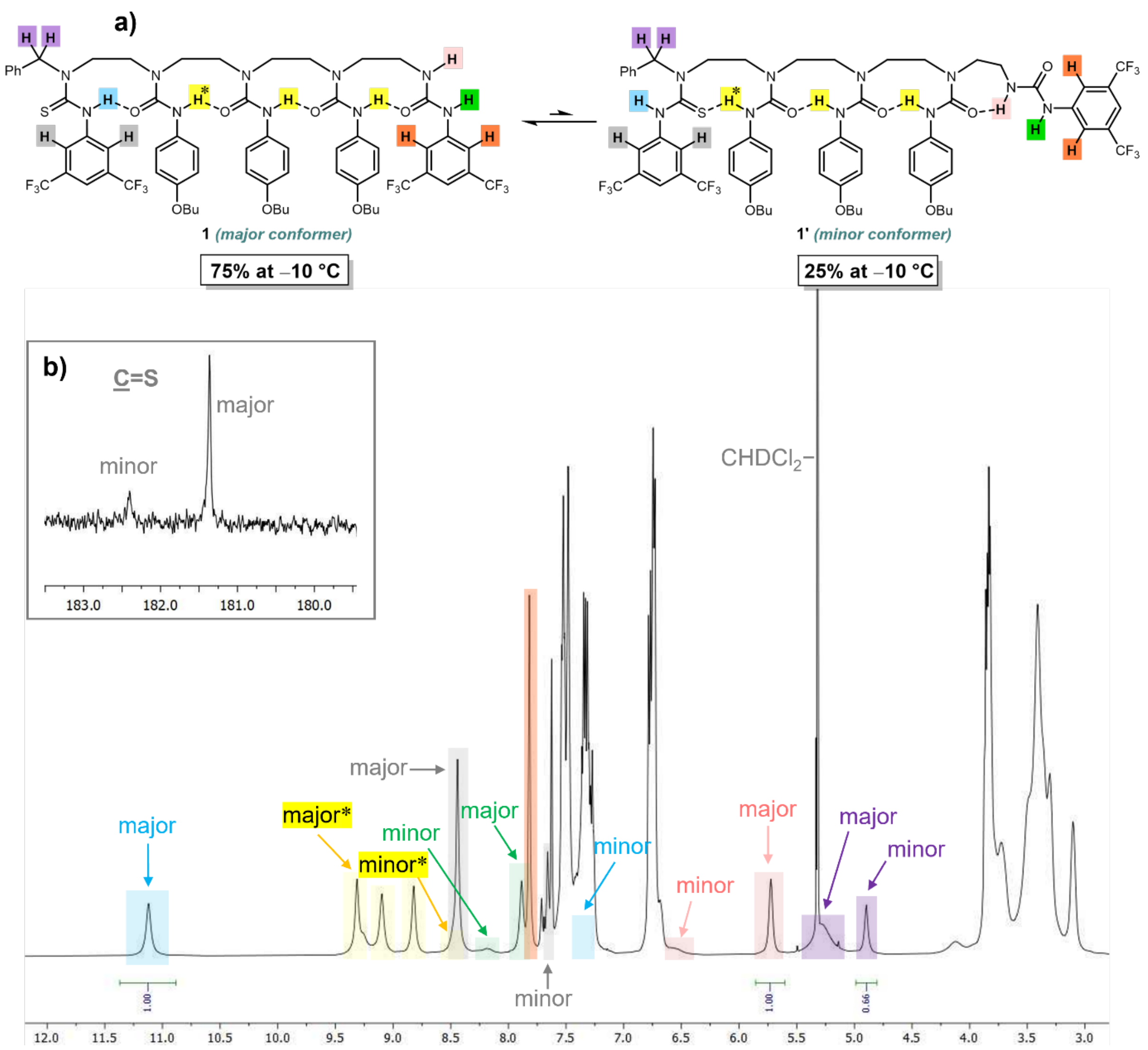

Figure S3 – (a) ^{1}**H NMR spectrum of compound 1 at −10 °C (500 MHz, 25 mM, CD_2Cl_2).** At this temperature, distinct signals are observed for both conformers because rotation about the urea N−CO bonds is slow on the NMR timescale. Representative signals are labelled on the spectra as belonging to the major or minor conformer, as supported by NOESY (EXSY) and ROESY experiments (*vide infra*). Comparison of the integration of the resolved thiourea proton of the major conformer (δ_H = 11.12 ppm, coloured blue) with the integration of the resolved benzylic methylene protons of the minor conformer (δ_H = 4.90 ppm, coloured purple) allows the conformer distribution to be quantified as 75:25; **(b) A portion of the** ^{13}**C NMR spectrum of compound 1 at −10 °C (126 MHz, 27 mM, CD_2Cl_2)**. The two signals observed in this region are assigned to the thiocarbonyl (C=S) resonances of the major and minor conformers.

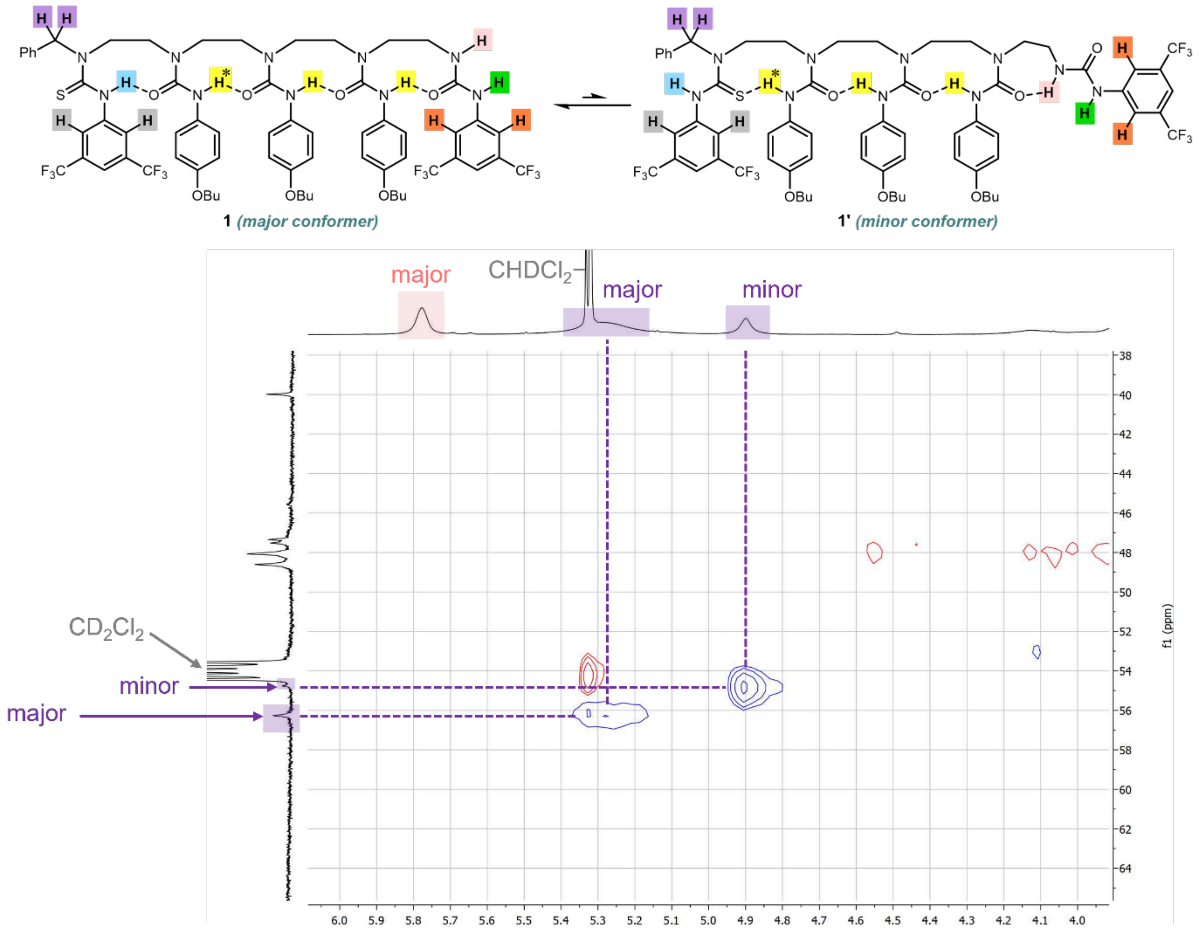

Figure S4 – A portion of the HSQC NMR spectrum of compound 1 at −10 ºC (27 mM, CD_2Cl_2). The spectrum shows correlations for the benzylic methylene signals of the major and minor conformers.

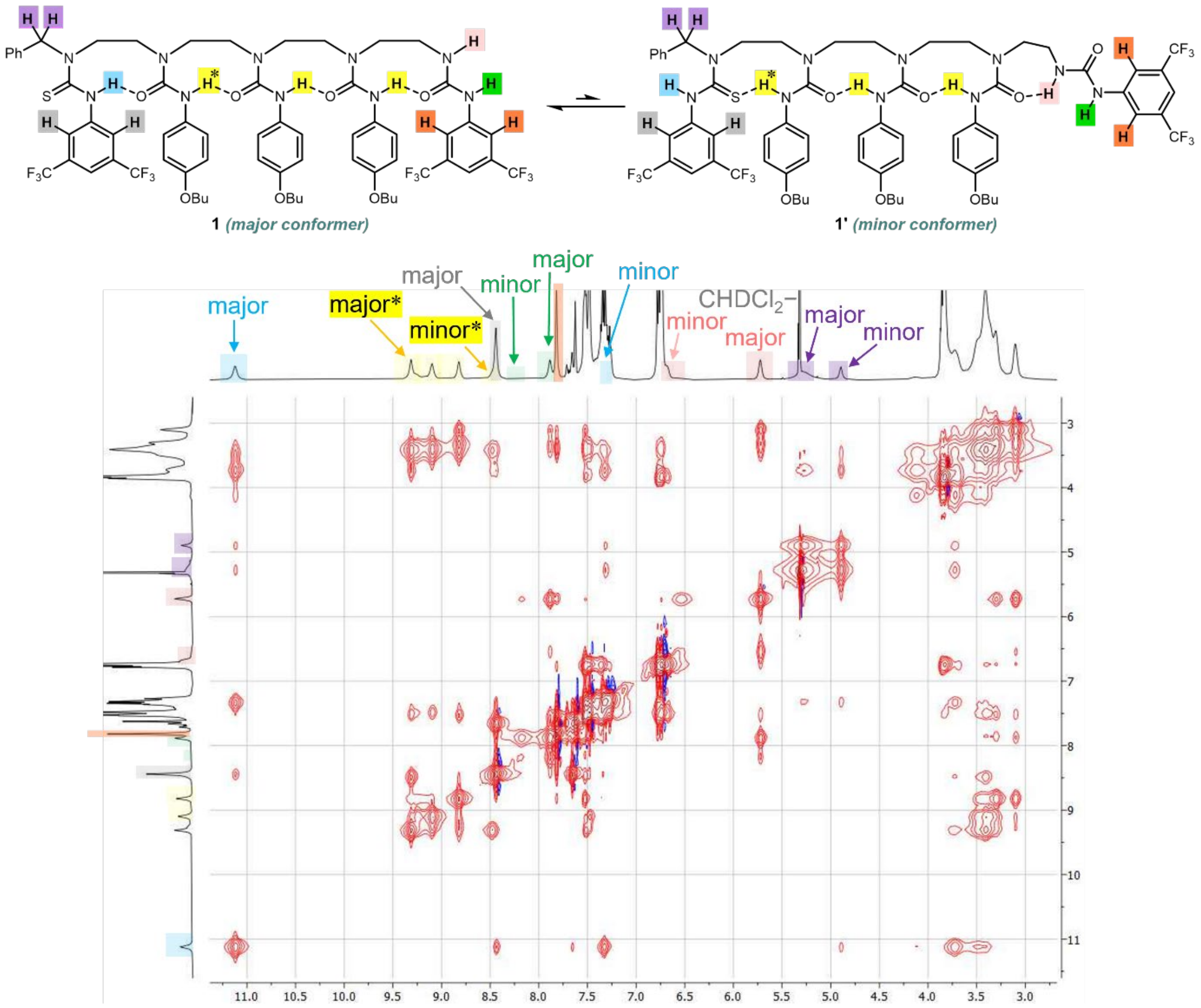

Figure S5 – NOESY / EXSY spectrum of compound 1 at −10 ºC (500 MHz, 25 mM, CD_2Cl_2). Cross-peaks arising from through space correlations (nOe) appear in the same phase (red) as the cross-peaks from rotational exchange (EXSY correlations), indicating that the nOes are negative.

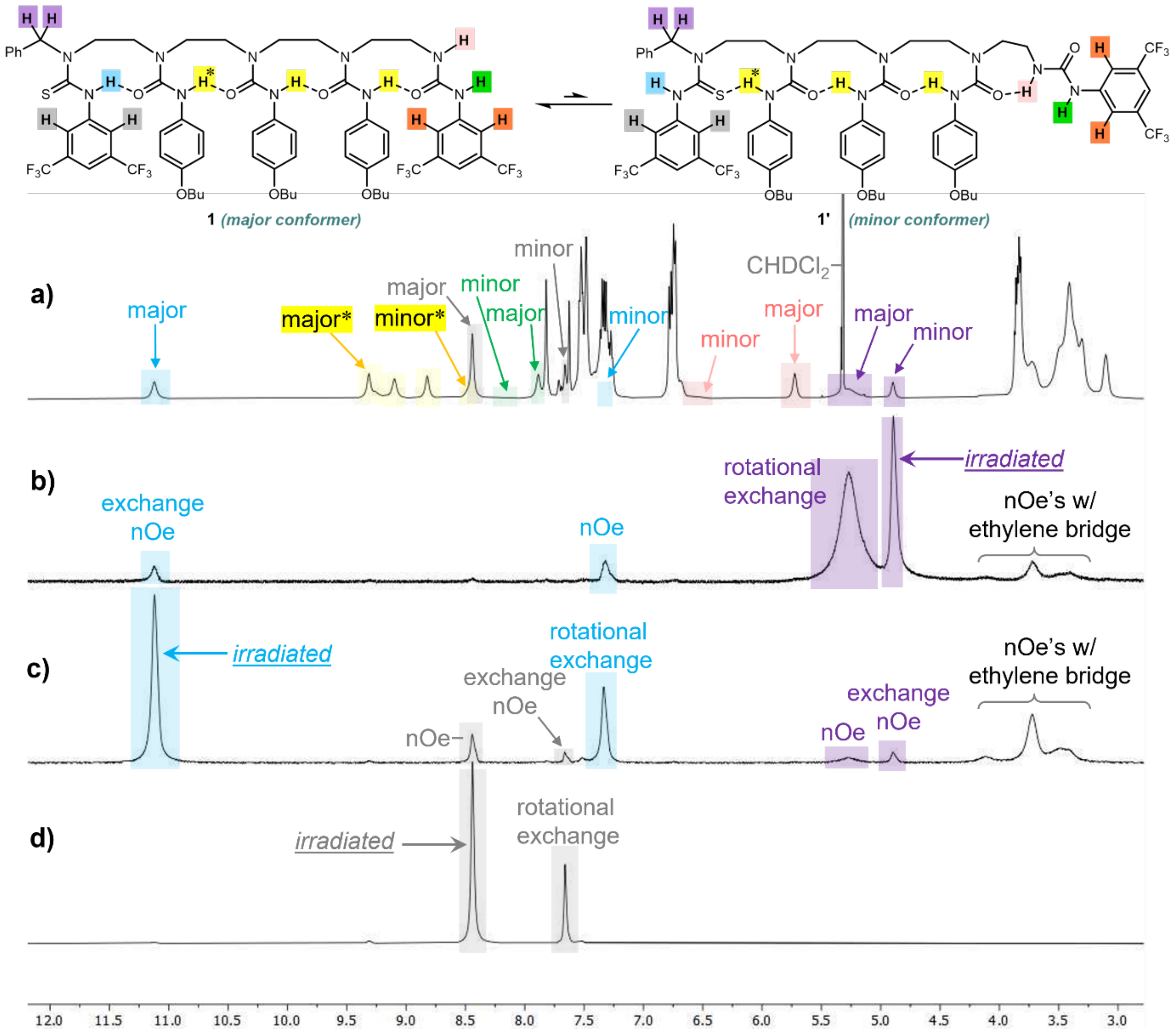

Figure S6 – Selective one-dimensional NOESY experiments for compound 1 at −10 ºC (500 MHz, 25 mM, CD_2Cl_2). (a) ^1H NMR spectrum at −10 ºC (for reference); (b) Irradiation of the benzylic methylene of the minor conformer (δ_H = 4.90 ppm, coloured purple); (c) Irradiation of the thiourea proton of the major conformer (δ_H = 11.12 ppm, coloured blue); and (d) Irradiation of the thiourea *ortho*-aryl protons of the major conformer (δ_H = 8.44 ppm, coloured grey). Signals arising from rotational exchange and through space nOe's appear in the same phase, indicating that the nOe's are negative. 'Exchange nOe's' are also observed as a result of excitation transfer between the two conformers due to rotational exchange occurring on the timescale of the nOe build-up.

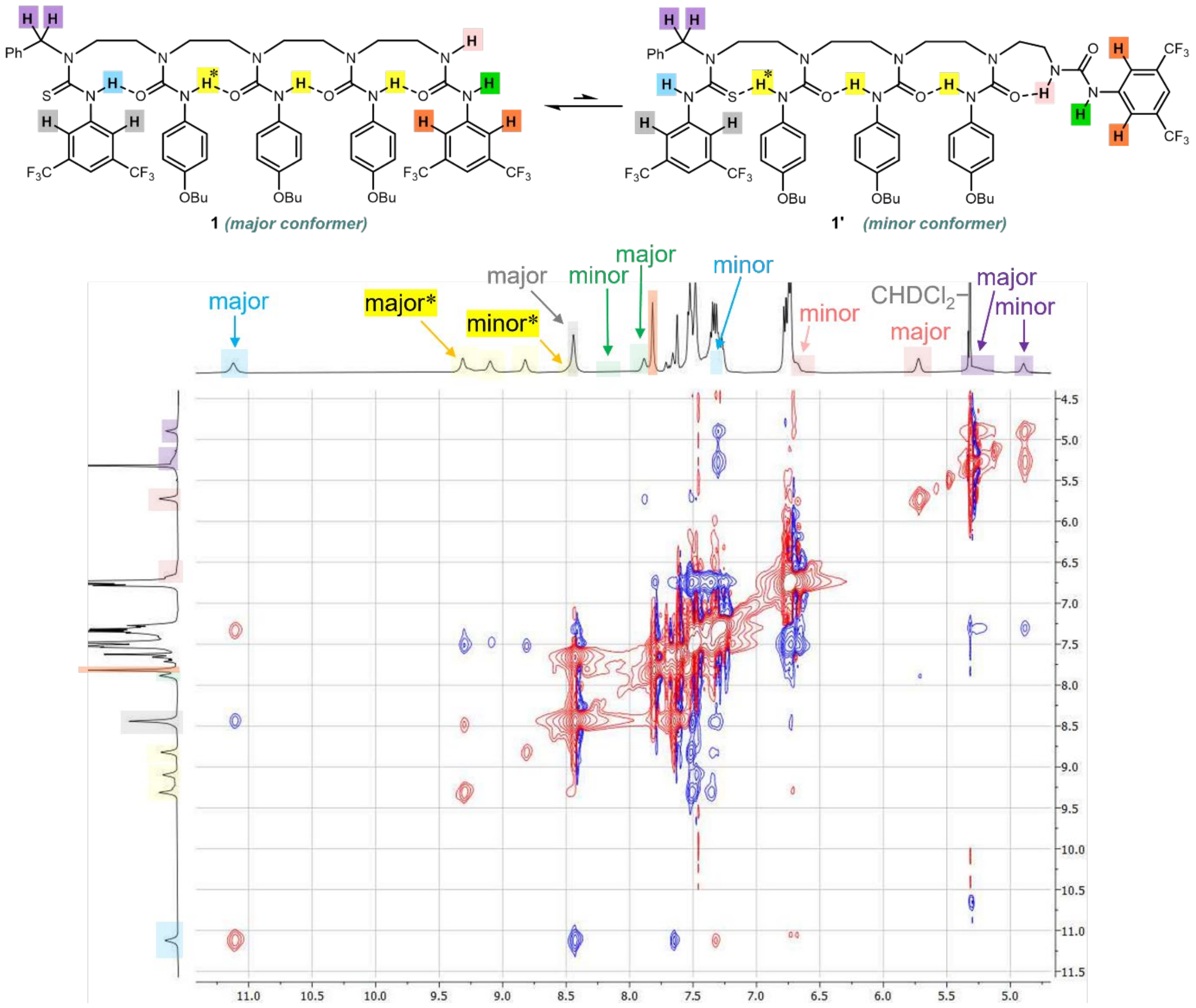

Figure S7a – ROESY spectrum of compound 1 at −10 ºC (500 MHz, 25 mM, CD_2Cl_2). Cross-peaks arising from through space correlations appear in blue, while cross peaks arising from rotational exchange appear in red (same phase as the diagonal).

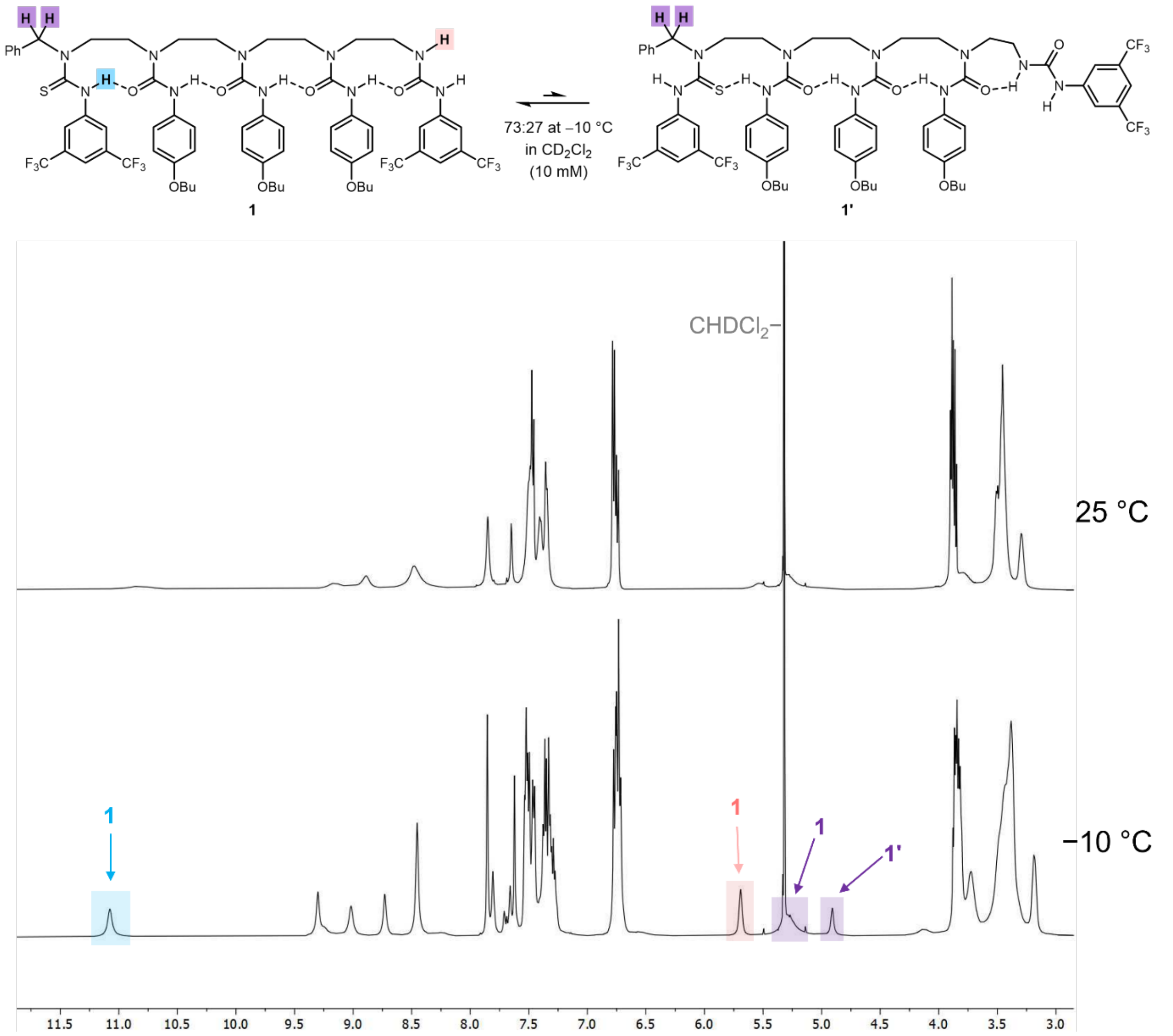

Figure S7b – Variable temperature ^{1}H NMR spectra of compound 1 (500 MHz, 10 mM, CD_2Cl_2). The ratio of **1**:**1'** at −10 °C is 73:27. See also Table S1.

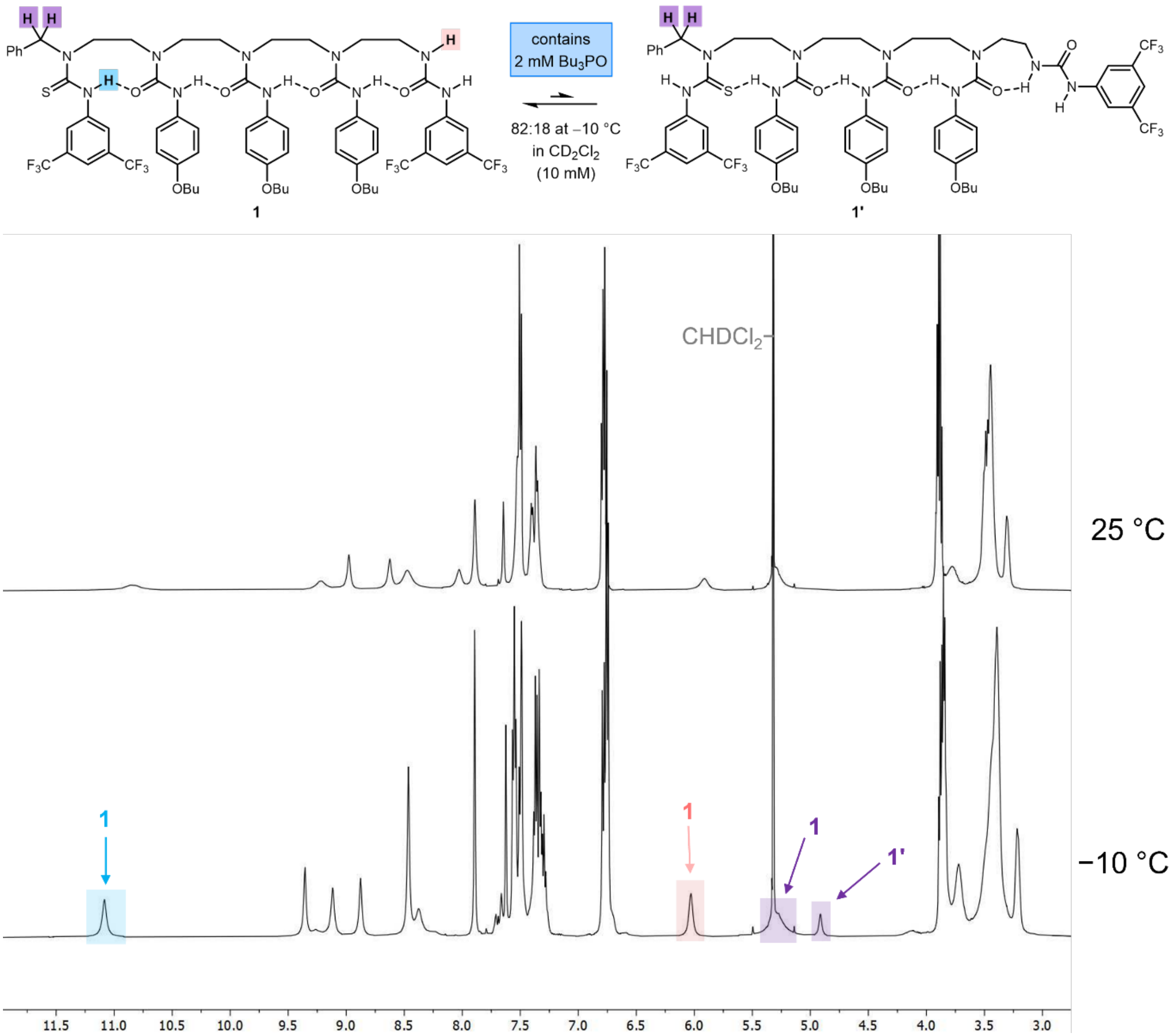

Figure S7c – Variable temperature ^{1}H NMR spectra of a mixture of compound 1 (10 mM) and Bu$_3$PO (2 mM) (500 MHz, CD$_2$Cl$_2$). The ratio of **1**:**1'** at −10 °C is 82:18. See also Table S1.

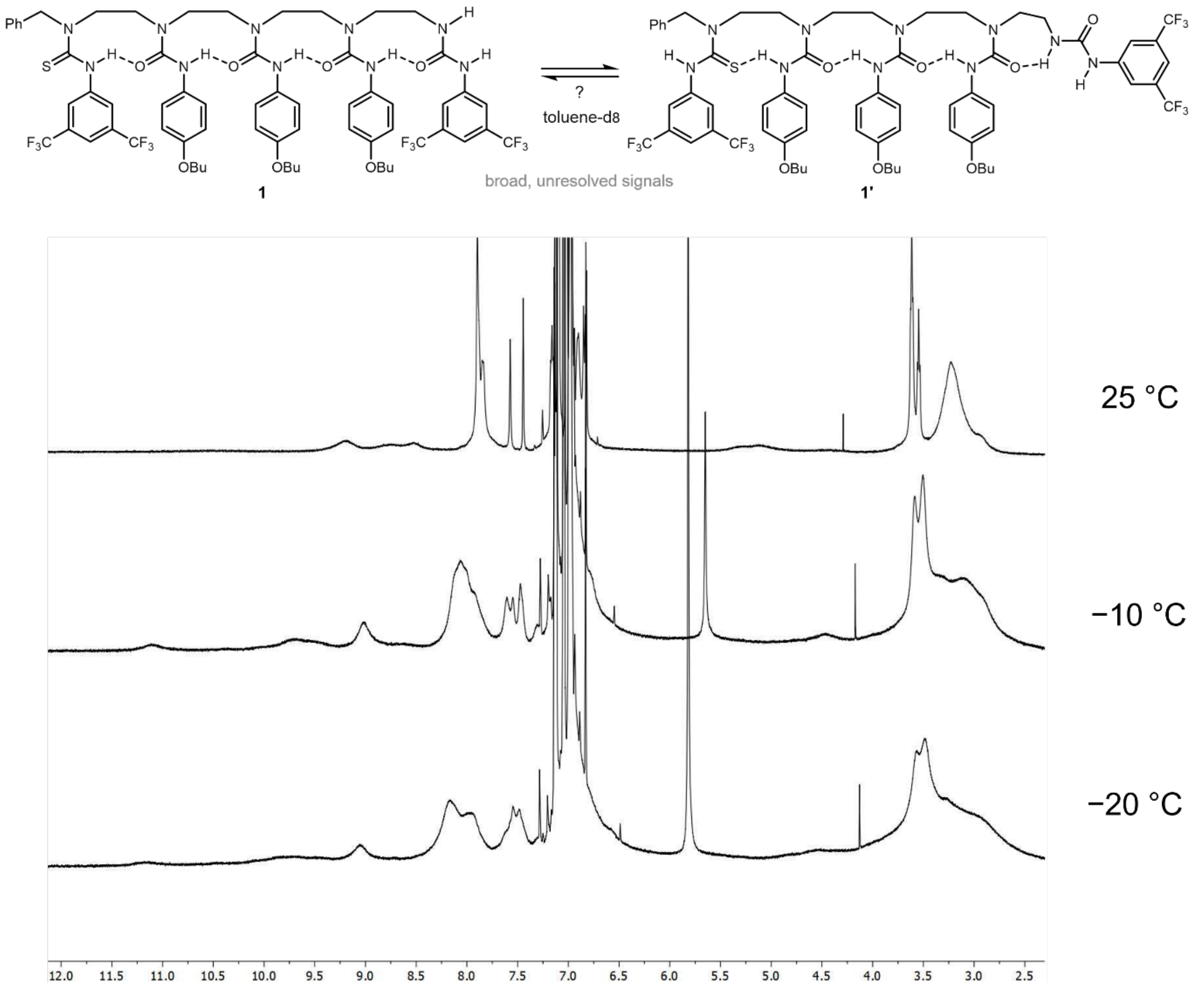

Figure S7d – Variable temperature ^{1}H NMR spectra of compound 1 (500 MHz, 2.5 mM, toluene-d8). The signals are broad and unresolved in toluene-d8, precluding conformational analysis.

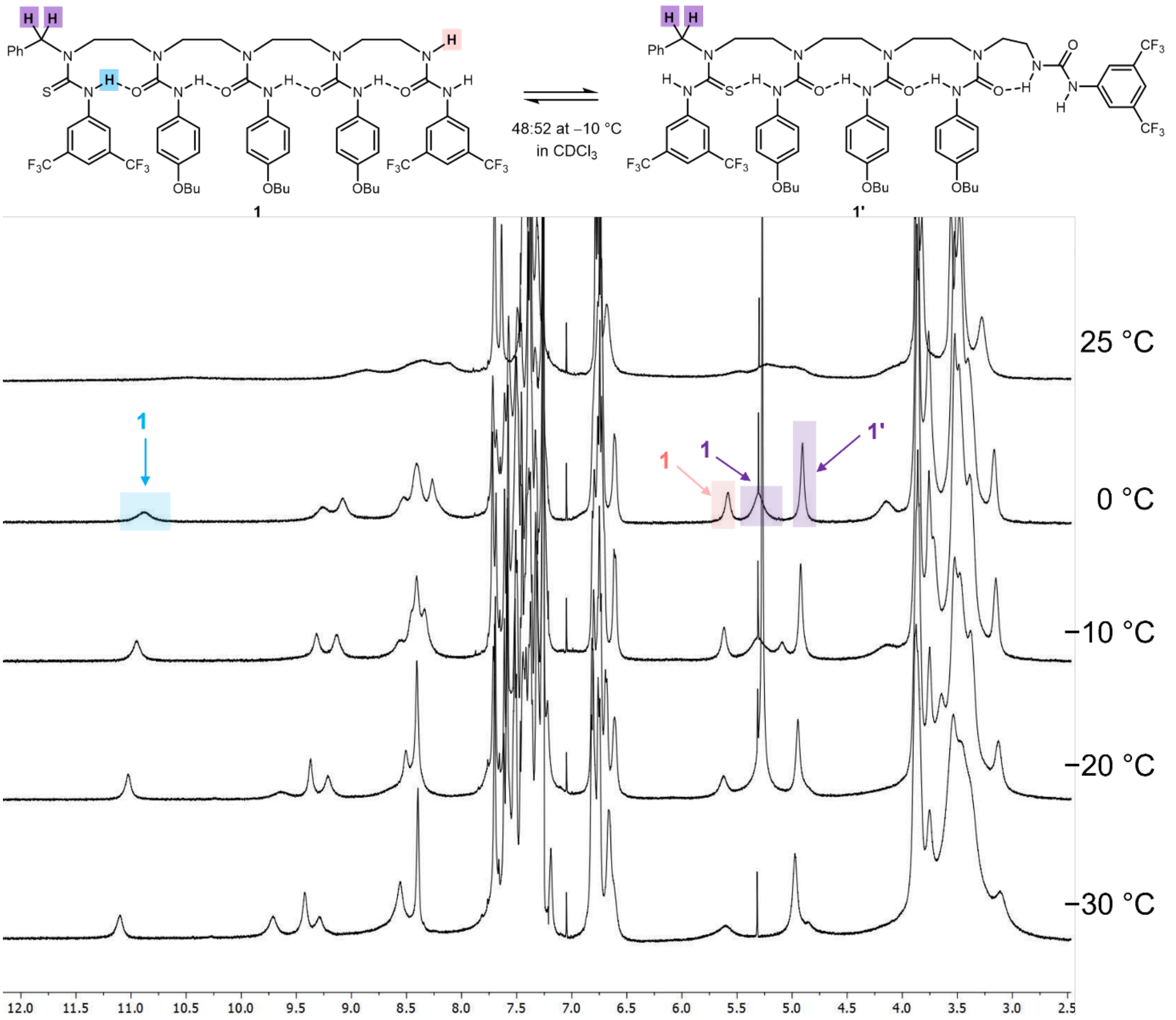

Figure S7e – Variable temperature ^{1}H NMR spectra of compound 1 (500 MHz, 2.5 mM, $CDCl_3$). The ratio of **1**:**1'** at −10 °C is 48:52. Thus, in $CDCl_3$, the relative populations of conformers **1** and **1'** are 'reversed' with respect to their populations in CD_2Cl_2 and all other solvents studied. See also Table S1.

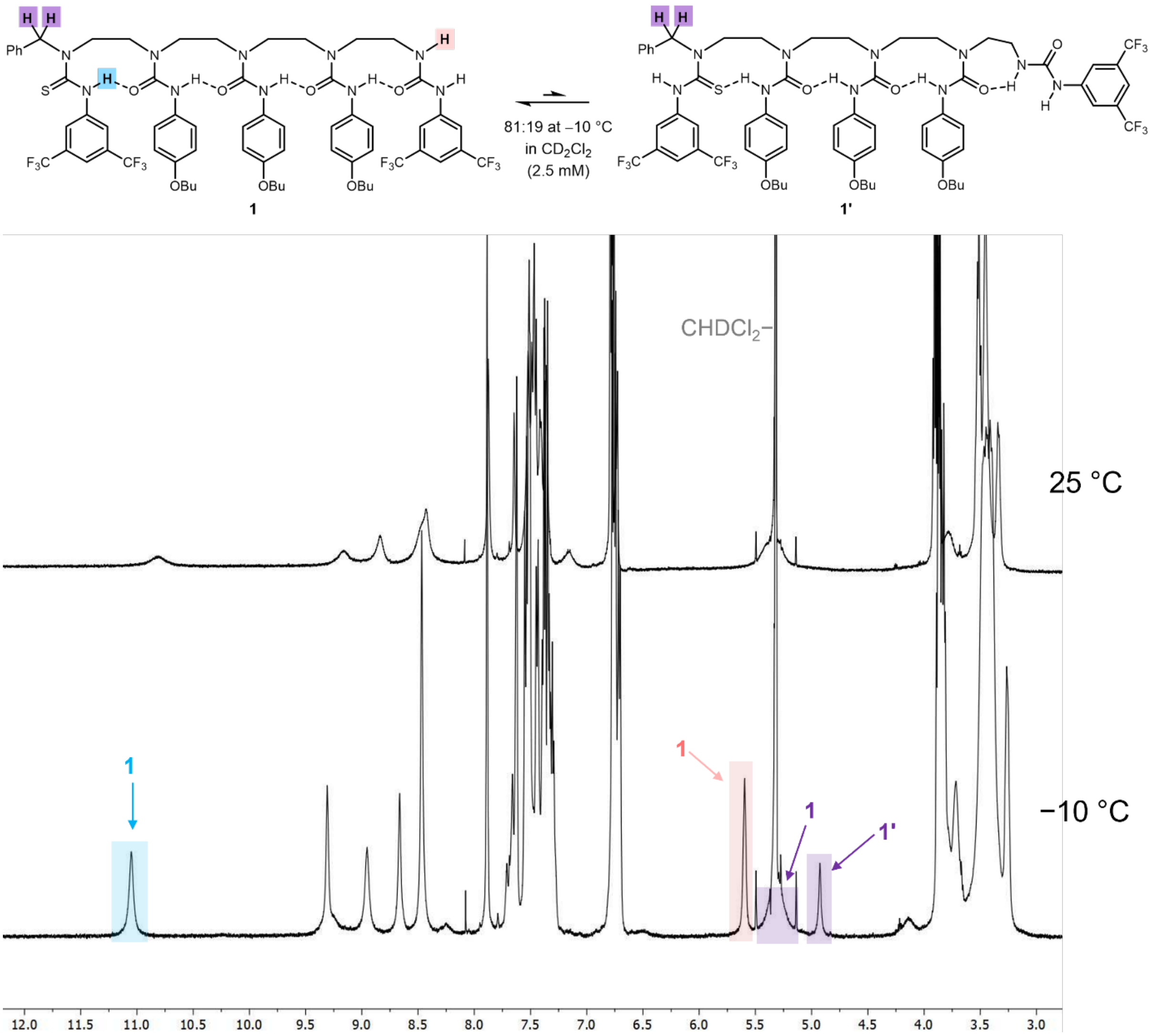

Figure S7f – Variable temperature ^{1}H NMR spectra of compound 1 (500 MHz, 2.5 mM, CD_2Cl_2). The ratio of **1**:**1'** at −10 °C is 81:19. See also Table S1.

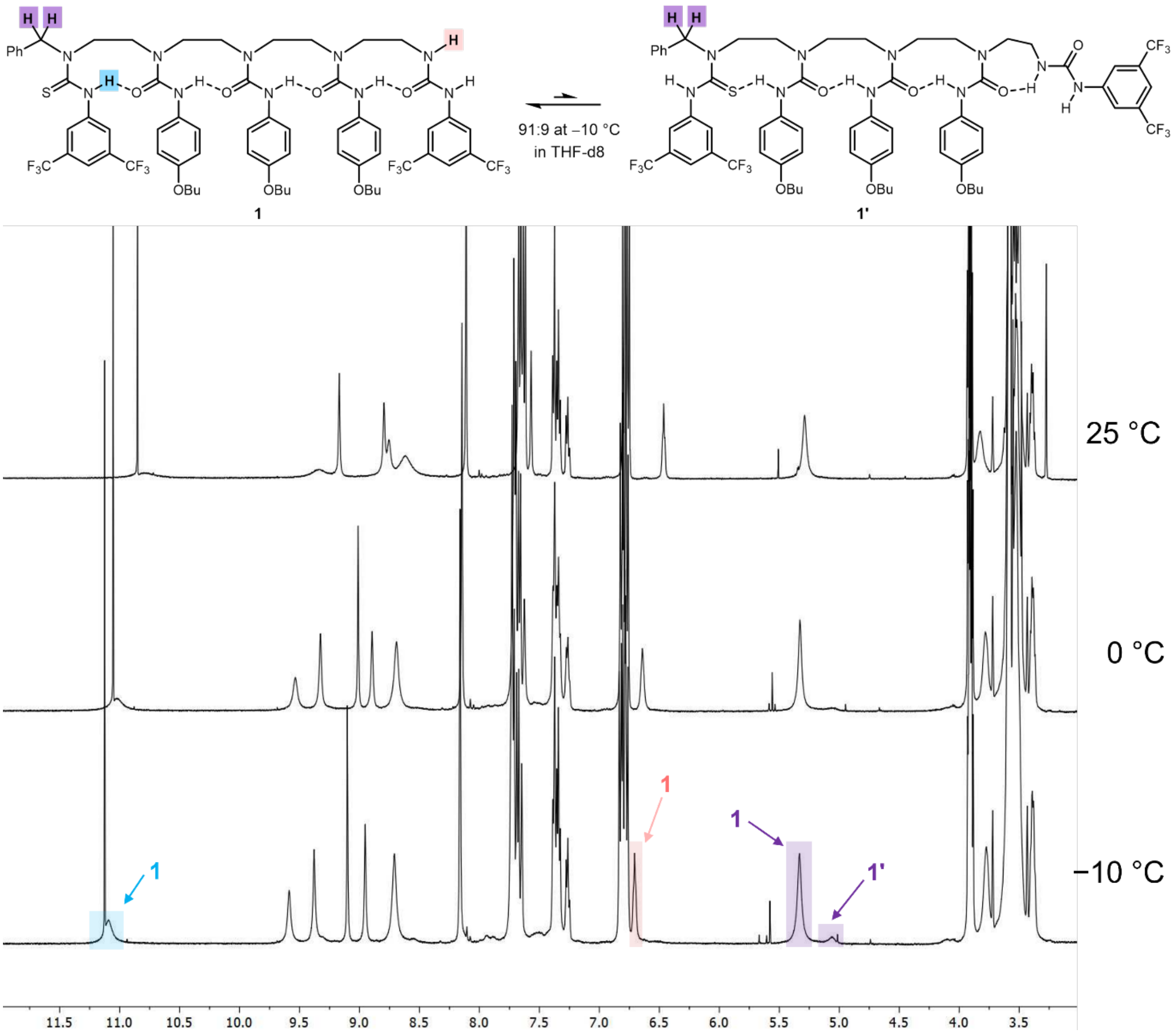

Figure S7g – Variable temperature ^{1}H NMR spectra of compound 1 (500 MHz, 2.5 mM, THF-d8). The ratio of **1**:**1'** at −10 °C is 91:9. See also Table S1.

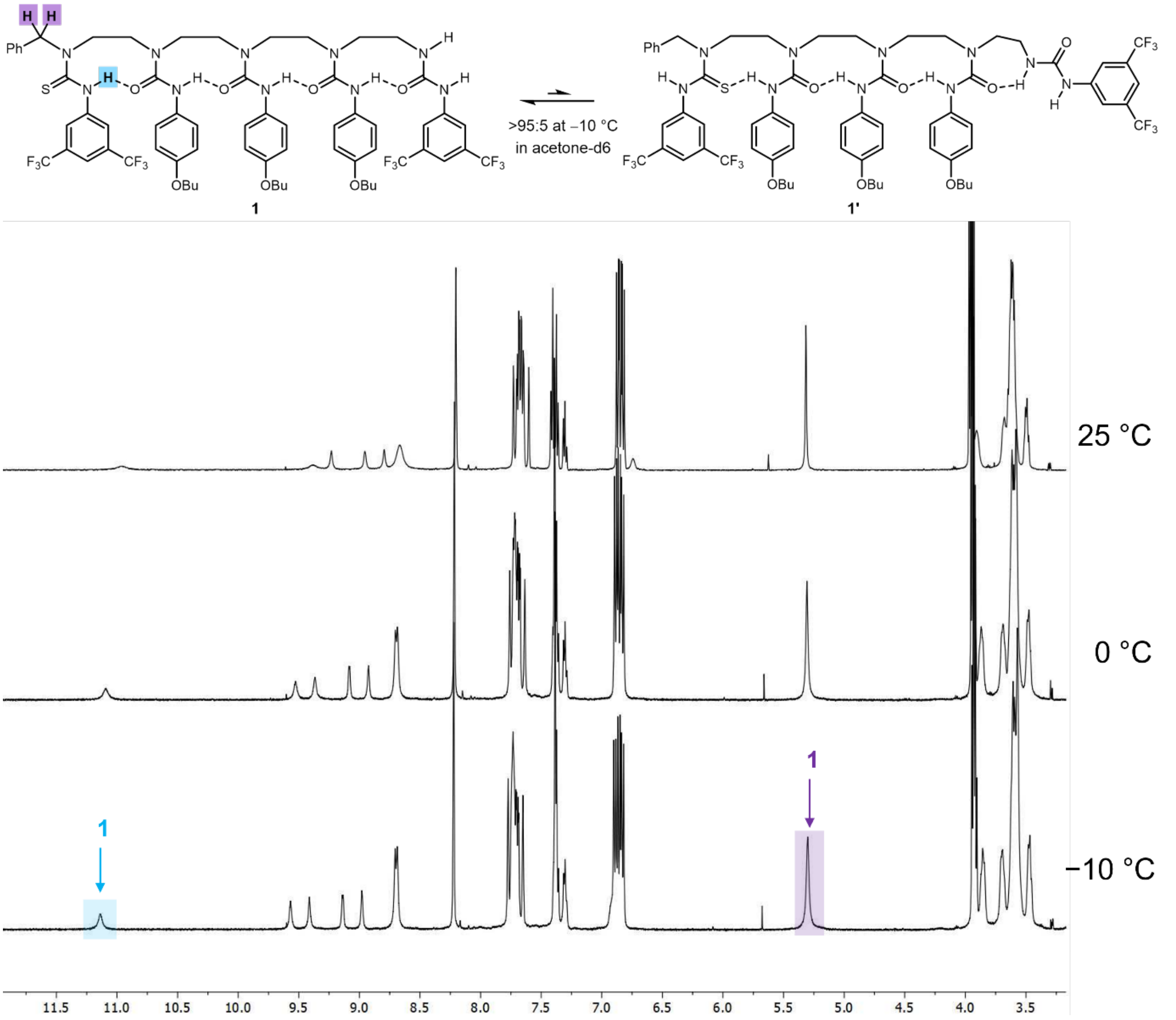

Figure S7h – Variable temperature ^{1}H NMR spectra of compound 1 (500 MHz, 2.5 mM, acetone-d6). The ratio of **1**:**1'** at −10 °C is >95:5. See also Table S1.

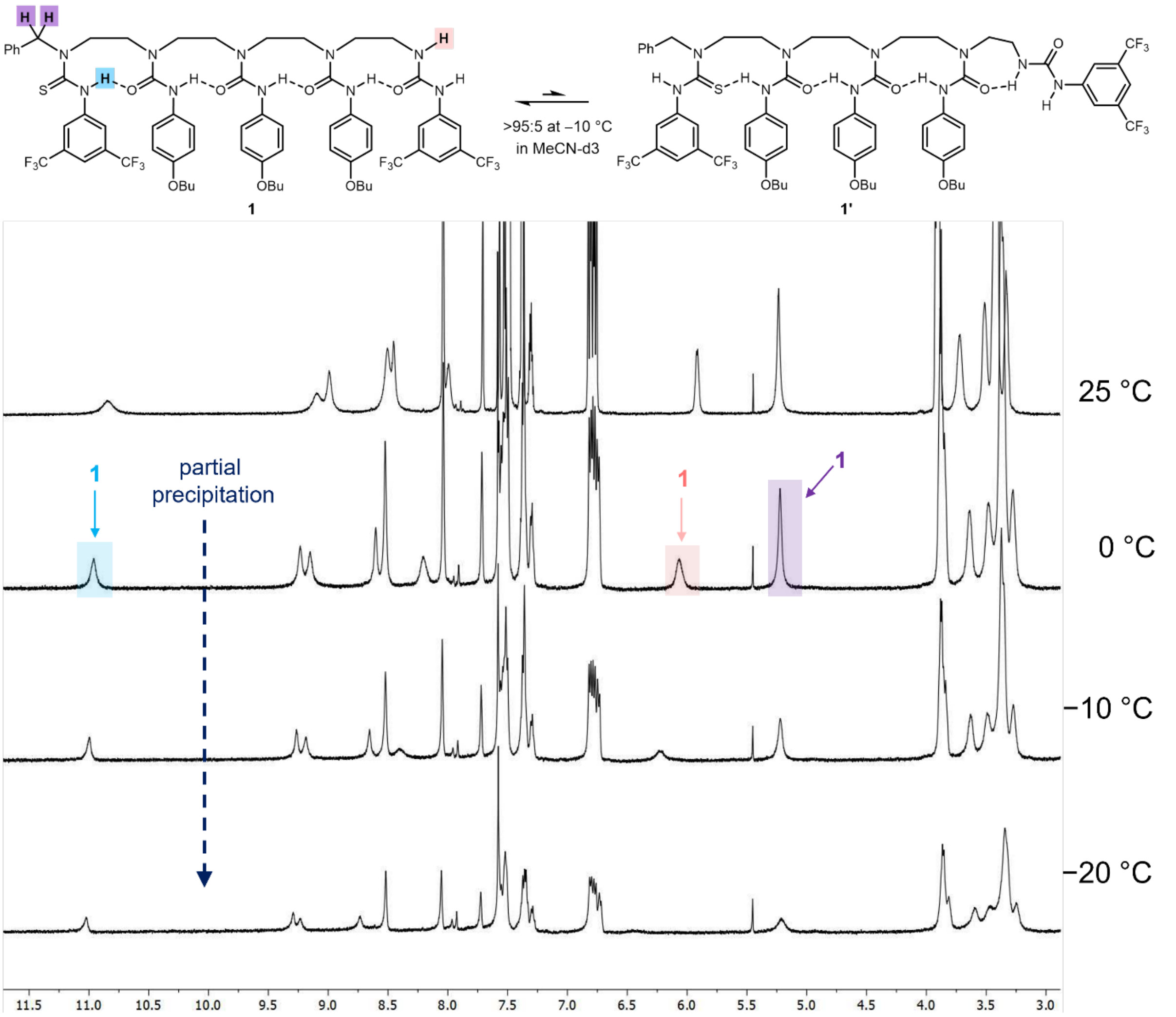

Figure S7i – Variable temperature ^{1}H NMR spectra of compound 1 (500 MHz, 2.5 mM, MeCN-d3). The ratio of **1**:**1'** at −10 °C is >95:5. Note that partial precipitation occurred upon cooling in the NMR spectrometer. See also Table S1.

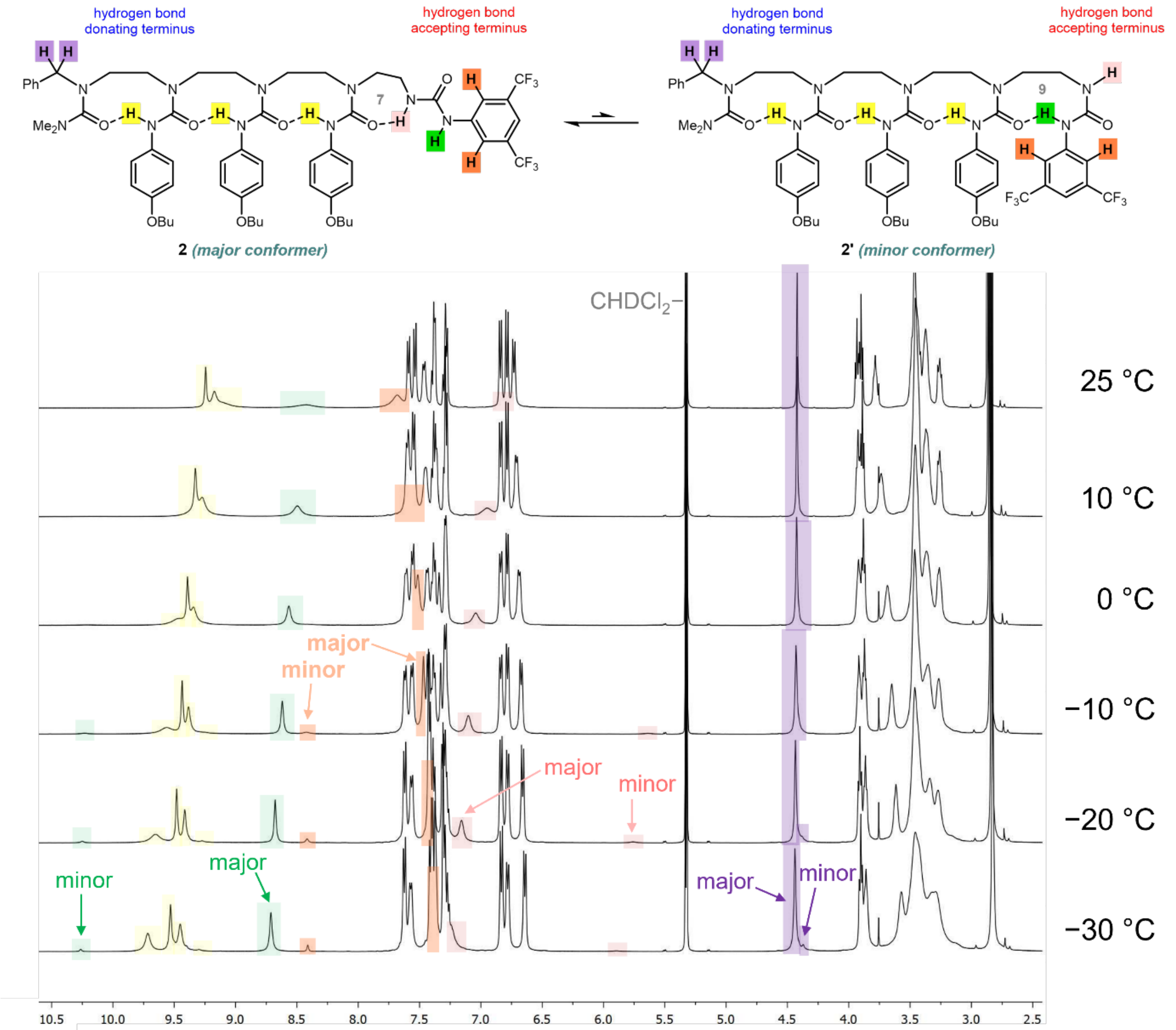

Figure S8 – Variable temperature ^{1}H NMR spectra of compound 2 (500 MHz, 25 mM, CD_2Cl_2). Two conformers are present, which differ only in the local conformation of the disubstituted urea − the global directionality of the hydrogen bond chain is the same in both conformers, with the disubstituted urea occupying the hydrogen bond-accepting terminus. The major conformer **2** (95% at −10 ºC) has the disubstituted urea in the *syn*,*syn*-conformation and its alkyl N–H participates in a seven-membered hydrogen-bonding ring with the adjacent urea. In the minor conformer **2'** (5% at −10 ºC), the disubstituted urea is in an *anti*,*syn*-conformation and only the aryl N–H participates in intramolecular hydrogen bonding; in this case in a nine-membered ring. Selected pairs of rotationally exchanging protons are highlighted in different colours (except for the internal urea N–Hs, which are all coloured yellow), and where the protons in rotational exchange resonate at distinct chemical shifts, the relevant signals are labelled on the spectra as belonging to the major or minor conformer. These assignments were supported by a NOESY (EXSY) experiments (*vide infra*).

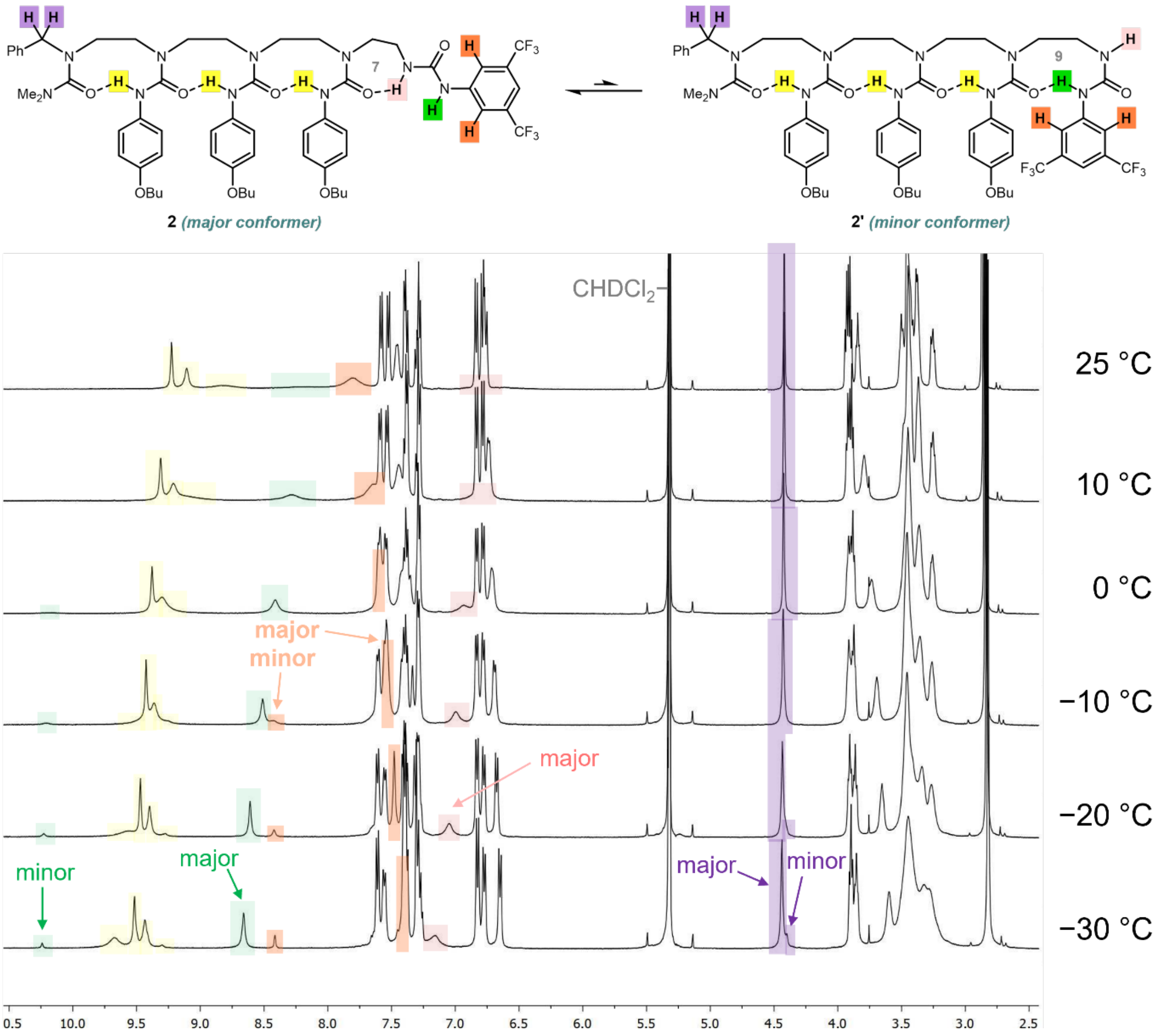

Figure S9 – Variable temperature ^{1}H NMR spectra of compound 2 at 10-fold dilution (500 MHz, 2.5 mM, CD_2Cl_2). The chemical shifts of all protons and the ratios of the major and minor conformer at each temperature are identical or similar to those observed at 25 mM, confirming that intermolecular interactions are insignificant at the concentrations used for analysis.

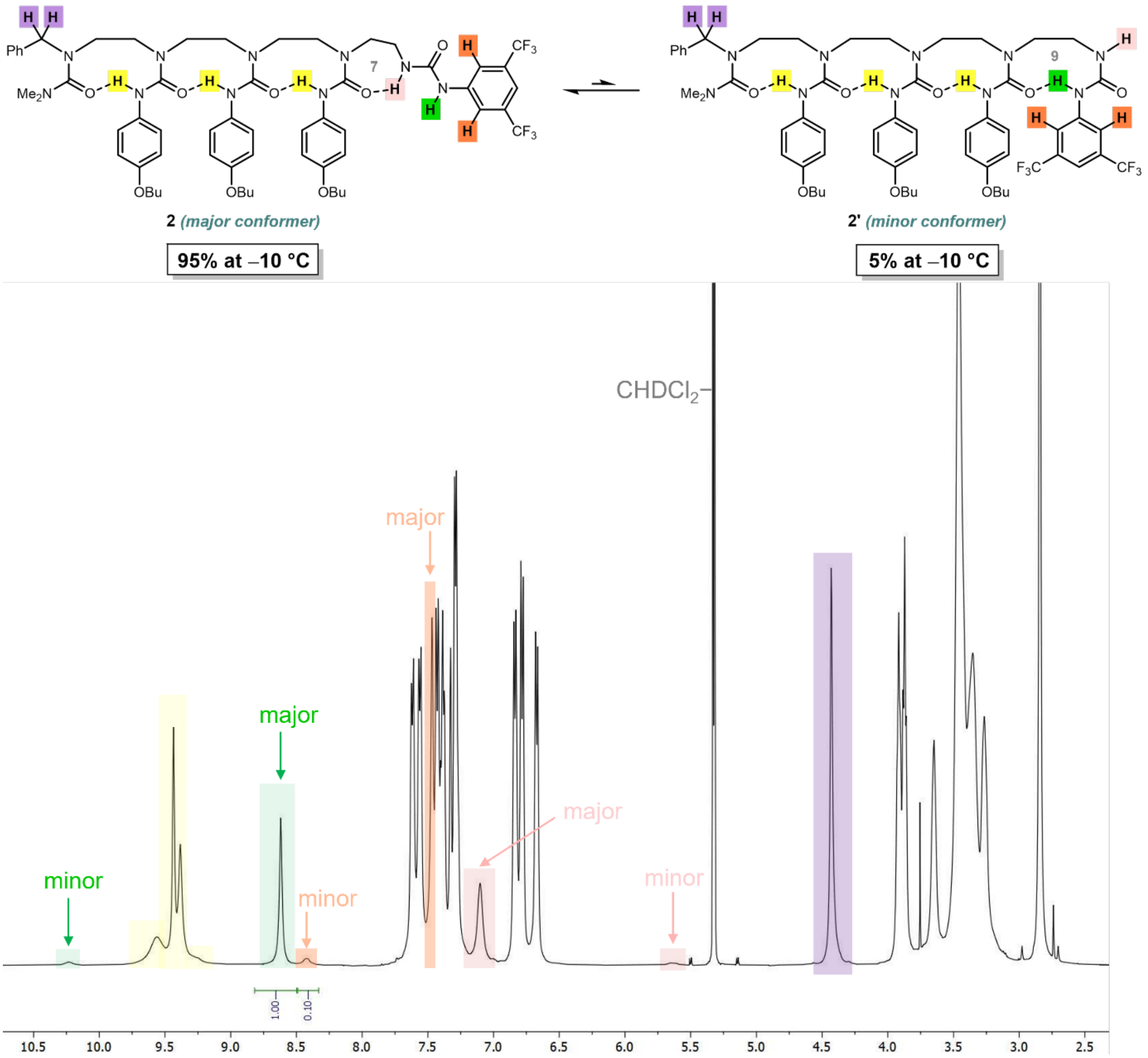

Figure S10 – ^{1}H NMR spectrum of compound 2 at −10 ºC (500 MHz, 25 mM, CD_2Cl_2). At this temperature, distinct signals are observed for both conformers because rotation about the urea N−CO bonds is slow on the NMR timescale. Resolved signals are labelled on the spectra as belonging to the major or minor conformer, as supported by a NOESY (EXSY) experiment (*vide infra*). Comparison of the integration of the resolved aryl N–H signal of the disubstituted urea for the major conformer (δ_H = 8.62 ppm, coloured green) with the integration of the resolved aryl ortho proton signal of the disubstituted urea of the minor conformer (δ_H = 8.42 ppm, coloured orange) allows the conformer distribution to be quantified as 95:5.

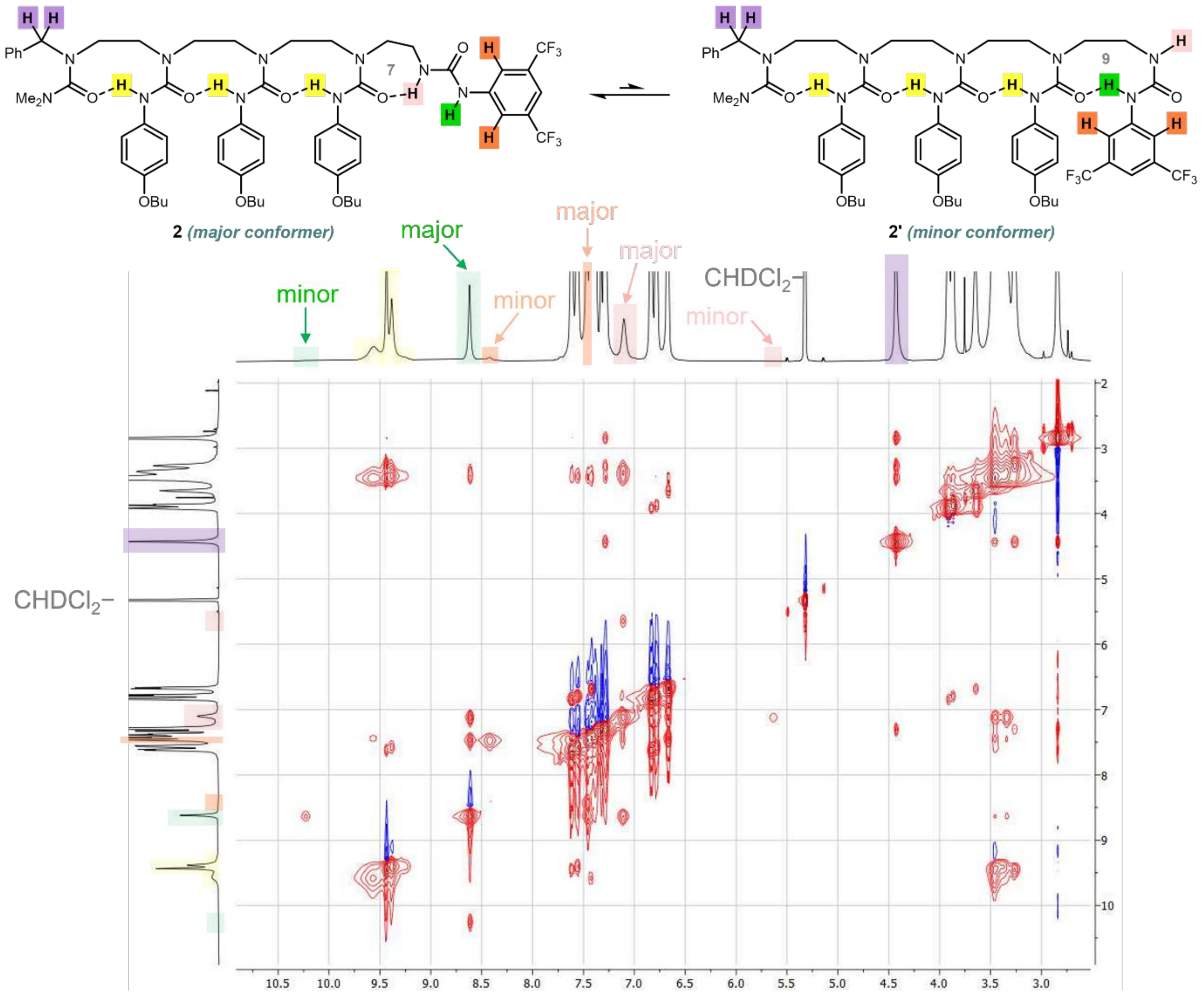

Figure S11 – NOESY / EXSY spectrum of compound 2 at −10 ºC (500 MHz, 25 mM, CD_2Cl_2). Cross-peaks arising from through space correlations (nOe) appear in the same phase (red) as the cross peaks from rotational exchange (EXSY correlations), indicating that the nOes are negative.

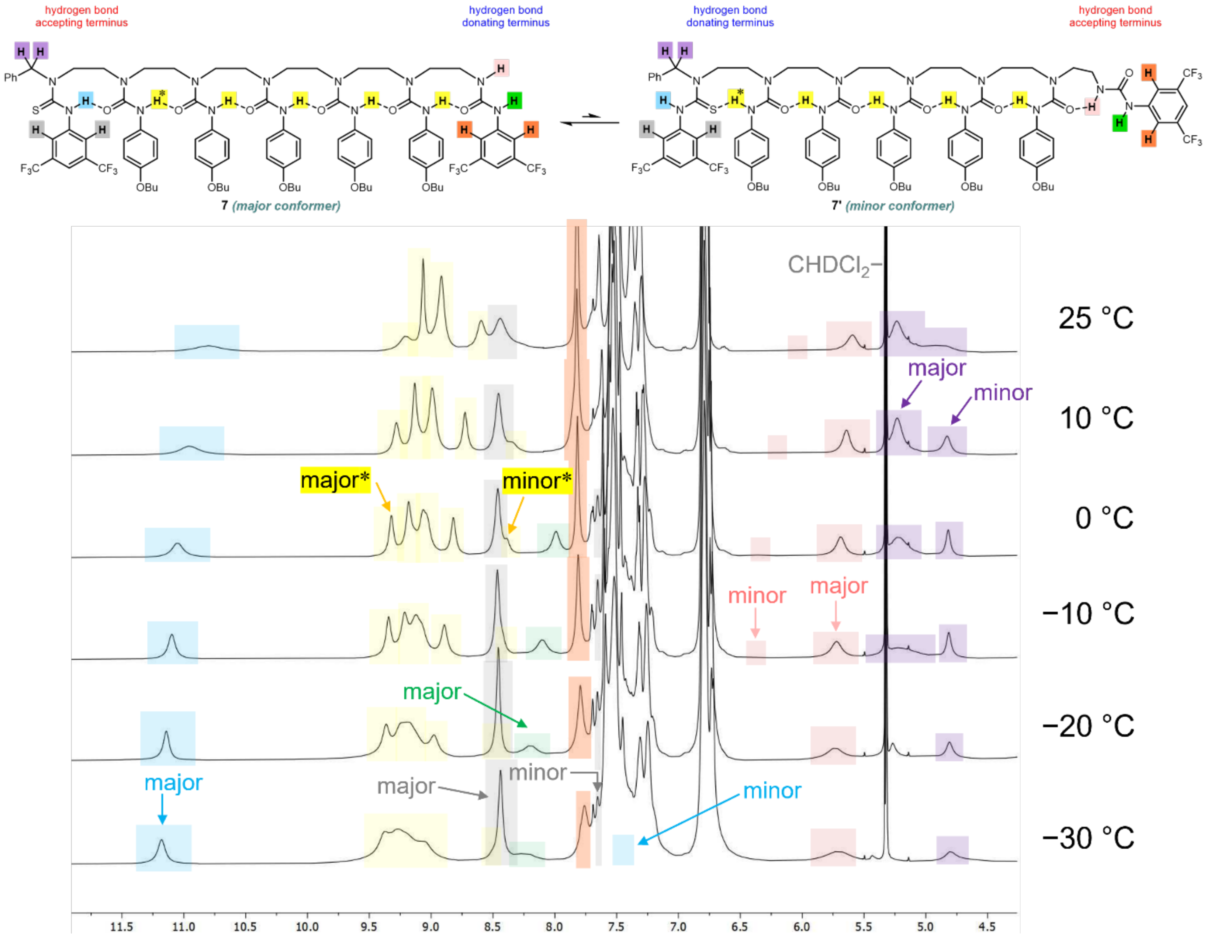

Figure S12 – Variable temperature ^{1}H NMR spectra of compound 7 (500 MHz, 25 mM, CD_2Cl_2). Like its shorter homologue **1**, two conformers are populated for **7**, which differ in the directionality of the hydrogen bond chain. The major conformer **7** (74% at −10 ºC) has the thiourea at the hydrogen bond-accepting terminus, while the minor conformer **7'** (26% at −10 ºC) has the thiourea at the hydrogen bond-donating terminus. Selected pairs of rotationally exchanging protons are highlighted in different colours (except for the internal urea N–Hs, which are all coloured yellow), and where the protons in rotational exchange resonate at distinct chemical shifts, the relevant signals are labelled on the spectra as belonging to the major or minor conformer. These assignments were supported by NOESY (EXSY) experiments (*vide infra*). The disappearance of the benzylic methylene signal at ~5.2 ppm (coloured purple) for the major conformer below −10 °C is attributed to the proximal sulfur atom slowing down rotation about the N−CH_2Ph bond at lower temperatures, causing signal broadening.

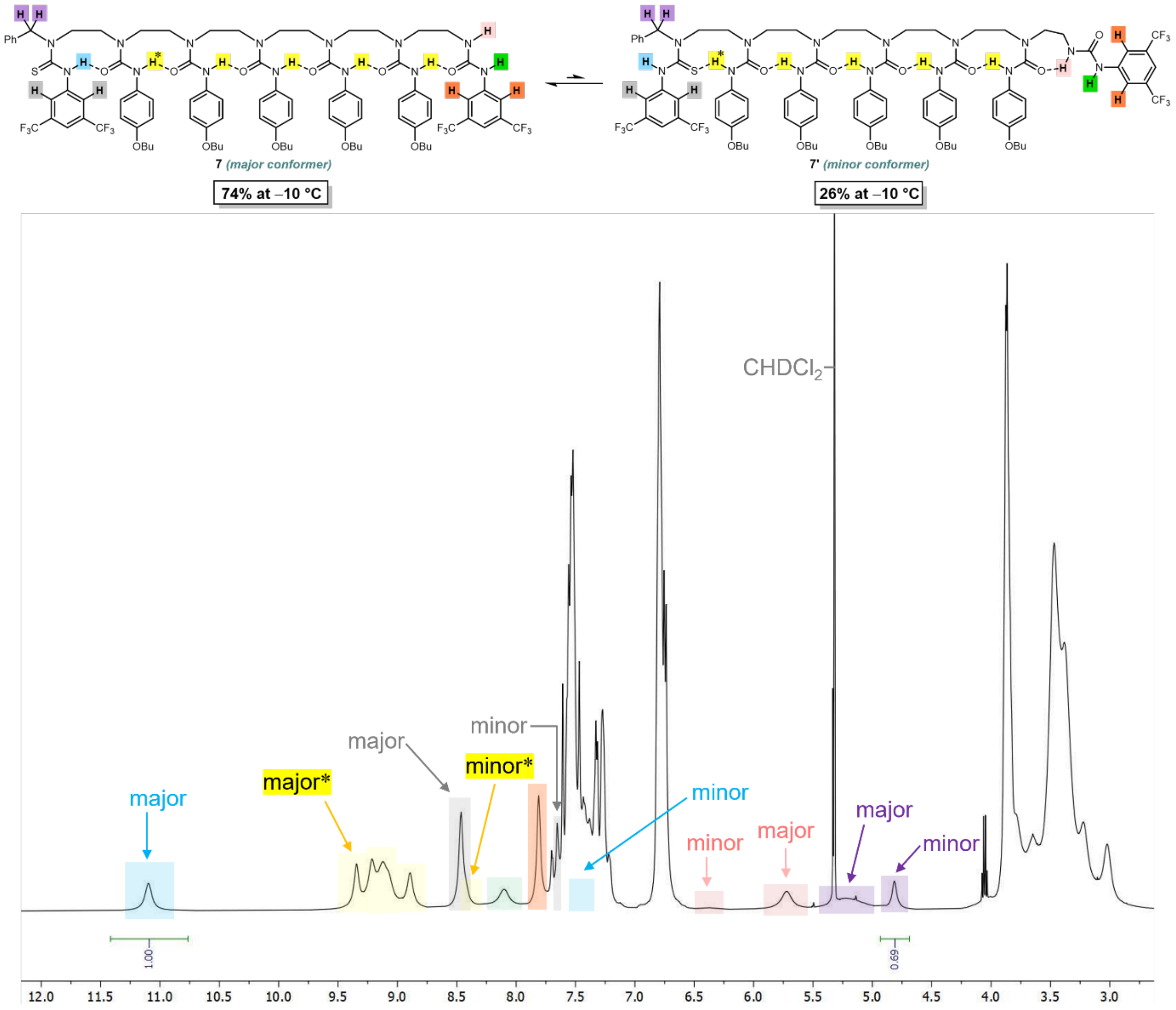

Figure S13 – ^{1}H NMR spectrum of compound 7 at −10 ºC (500 MHz, 25 mM, CD_2Cl_2). At this temperature, distinct signals are observed for both conformers because rotation about the urea N−CO bonds is slow on the NMR timescale. Representative signals are labelled on the spectra as belonging to the major or minor conformer, as supported by NOESY (EXSY) experiments (*vide infra*). Comparison of the integration of the resolved thiourea proton of the major conformer (δ_H = 11.10 ppm, coloured blue) with the integration of the resolved benzylic methylene protons of the minor conformer (δ_H = 4.81 ppm, coloured purple) allows the conformer distribution to be quantified as 74:26.

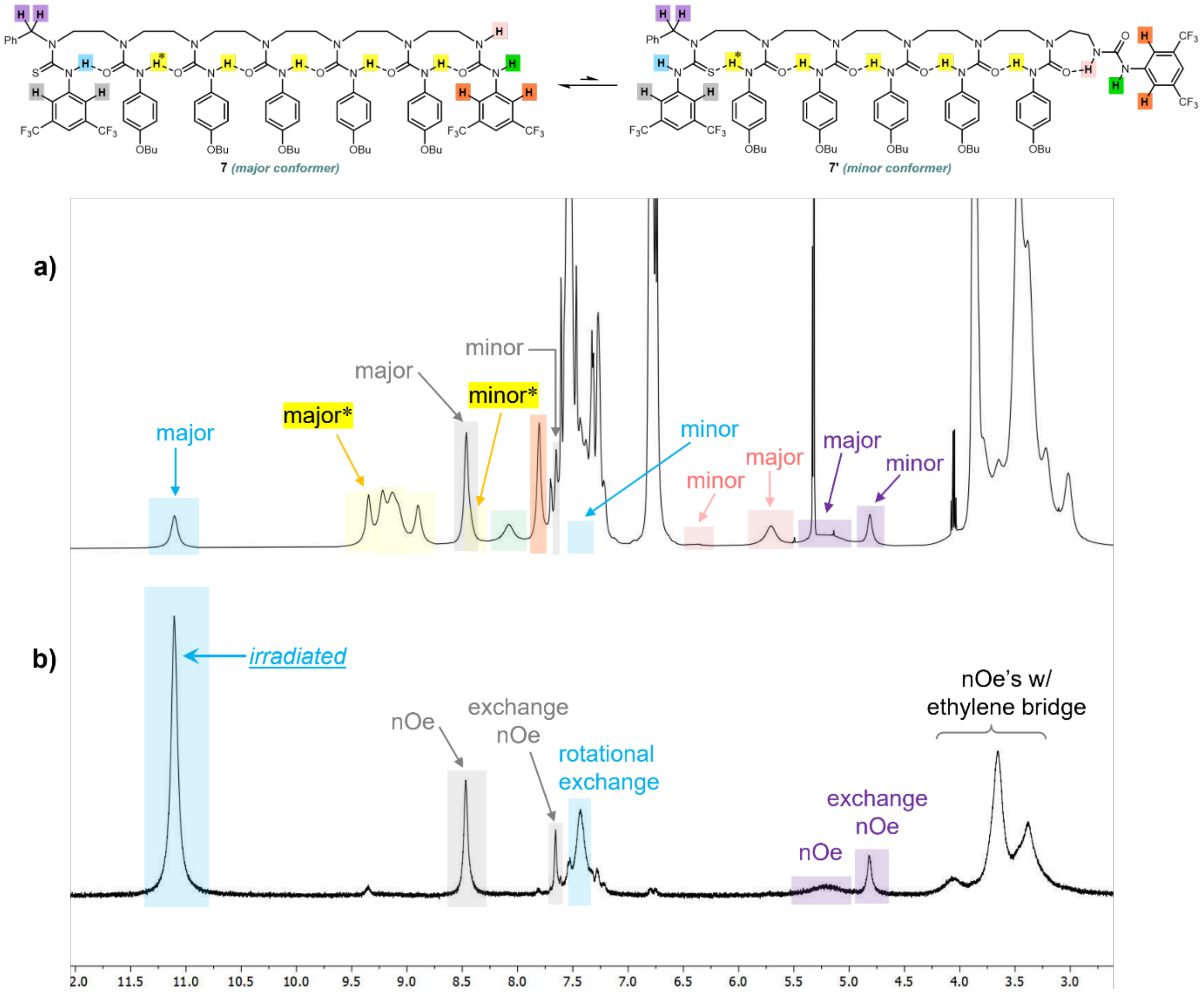

Figure S14 – Selective one-dimensional NOESY experiment for compound 7 at −10 ºC (500 MHz, 25 mM, CD_2Cl_2). (a) ^{1}H NMR spectrum at −10 ºC (for reference); (b) Irradiation of the thiourea proton of the major conformer (δ_H = 11.10 ppm, coloured blue). Signals arising from rotational exchange and through space nOes appear in the same phase, indicating that the nOes are negative. 'Exchange nOes' are also observed as a result of excitation transfer between the two conformers due to rotational exchange occurring on the timescale of the nOe build-up.

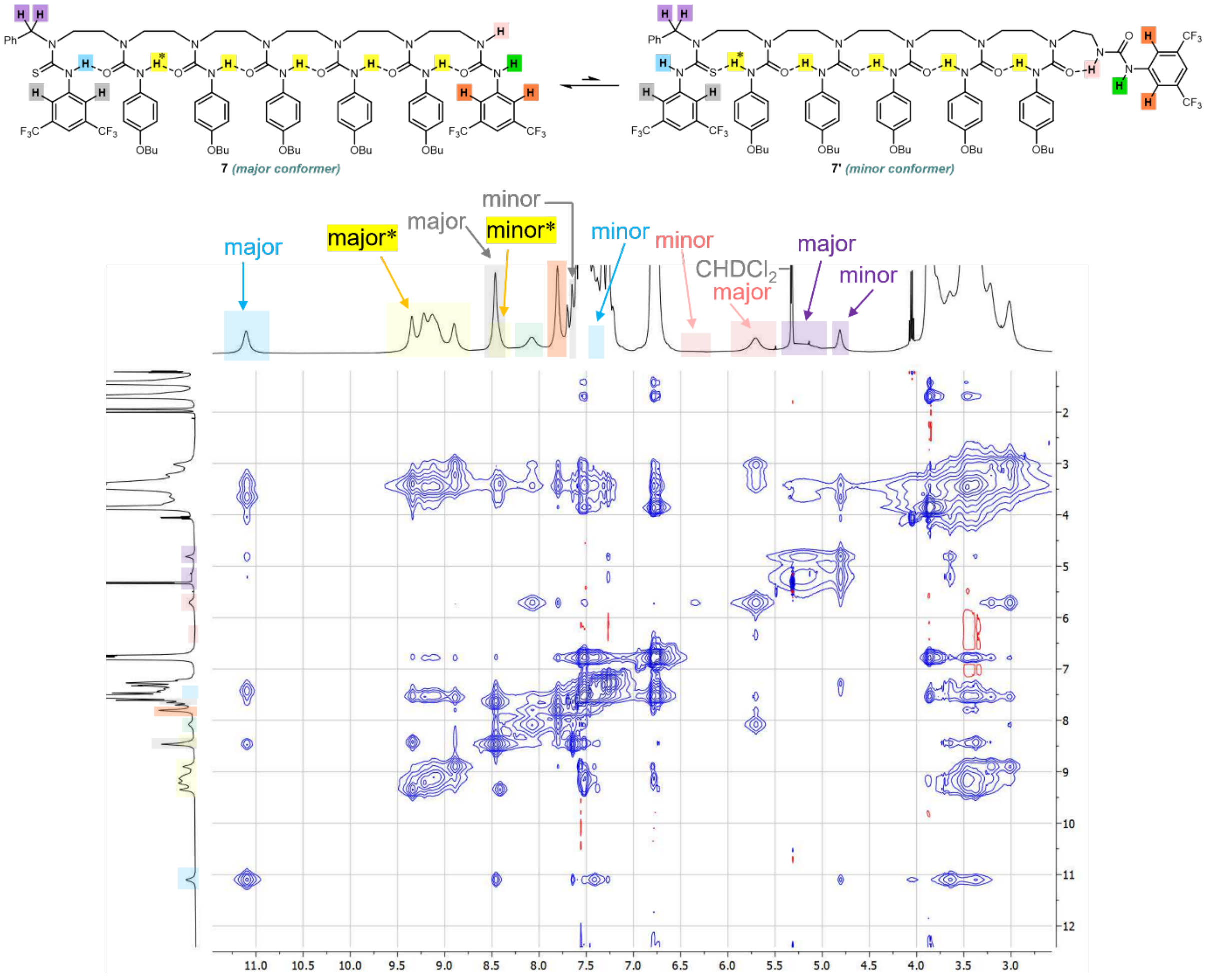

Figure S15 – NOESY / EXSY spectrum of compound 7 at −10 ºC (500 MHz, 25 mM, CD_2Cl_2). Cross-peaks arising from through space correlations (nOe) appear in the same phase as the cross peaks from rotational exchange (EXSY correlations), indicating that the nOes are negative.

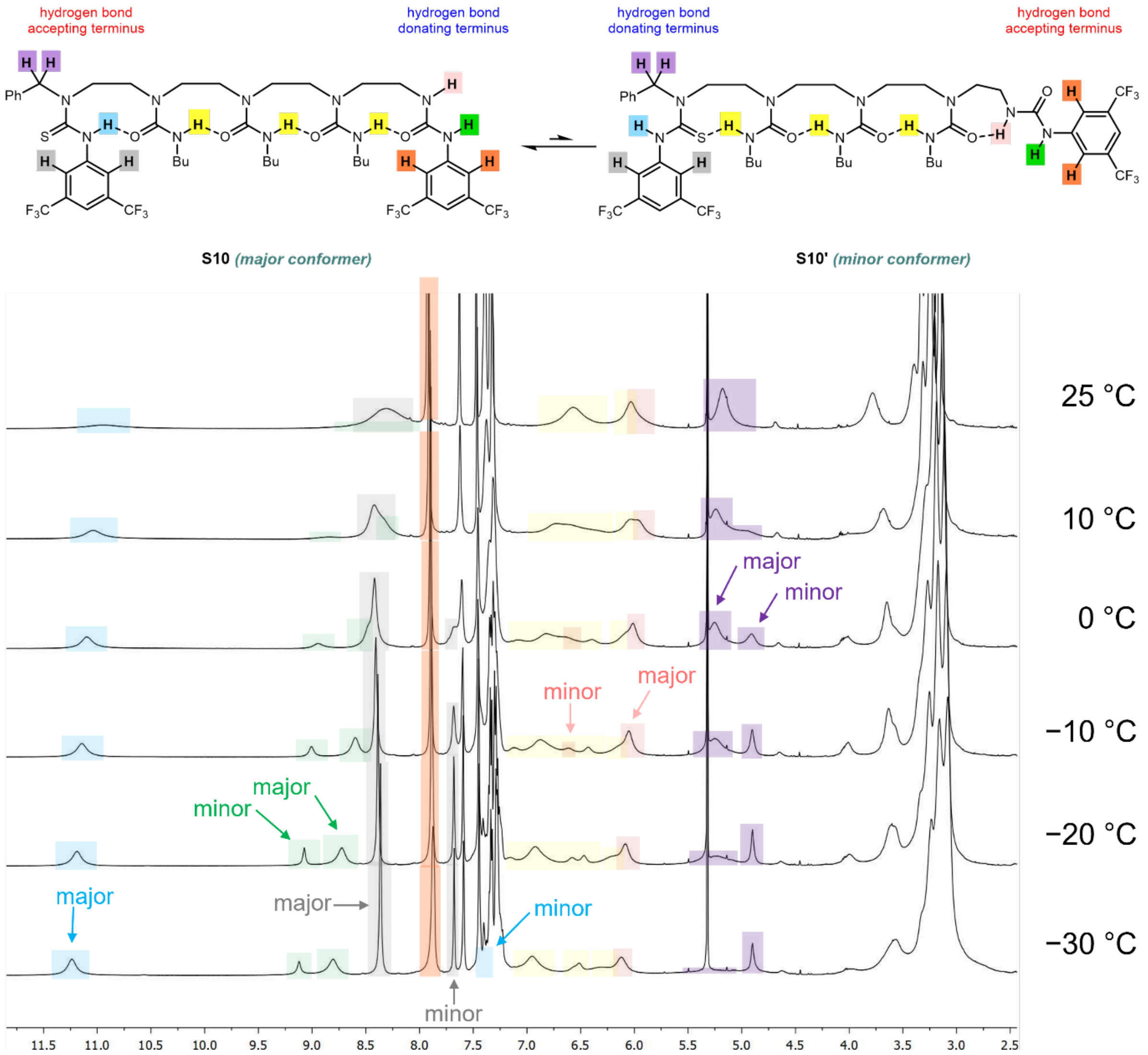

Figure S16 – Variable temperature ^{1}H NMR spectra of compound S10 (500 MHz, 25 mM, CD_2Cl_2). Like **1**, two conformers are populated for alkyl urea analogue **S10**, which differ in the directionality of the hydrogen bond chain. The major conformer **S10** (72% at −10 ºC) has the thiourea at the hydrogen bond-accepting terminus, while the minor conformer **S10'** (28% at −10 ºC) has the thiourea at the hydrogen bond-donating terminus. Selected pairs of rotationally exchanging protons are highlighted in different colours (except for the internal urea N–Hs, which are all coloured yellow), and where the protons in rotational exchange resonate at distinct chemical shifts, the relevant signals are labelled on the spectra as belonging to the major or minor conformer. These assignments were supported by NOESY (EXSY) experiments. The disappearance of the benzylic methylene signal at ~5.2 ppm (coloured purple) for the major conformer below −20 °C is attributed to the proximal sulfur atom slowing down rotation about the N−CH_2Ph bond at lower temperatures, causing signal broadening.

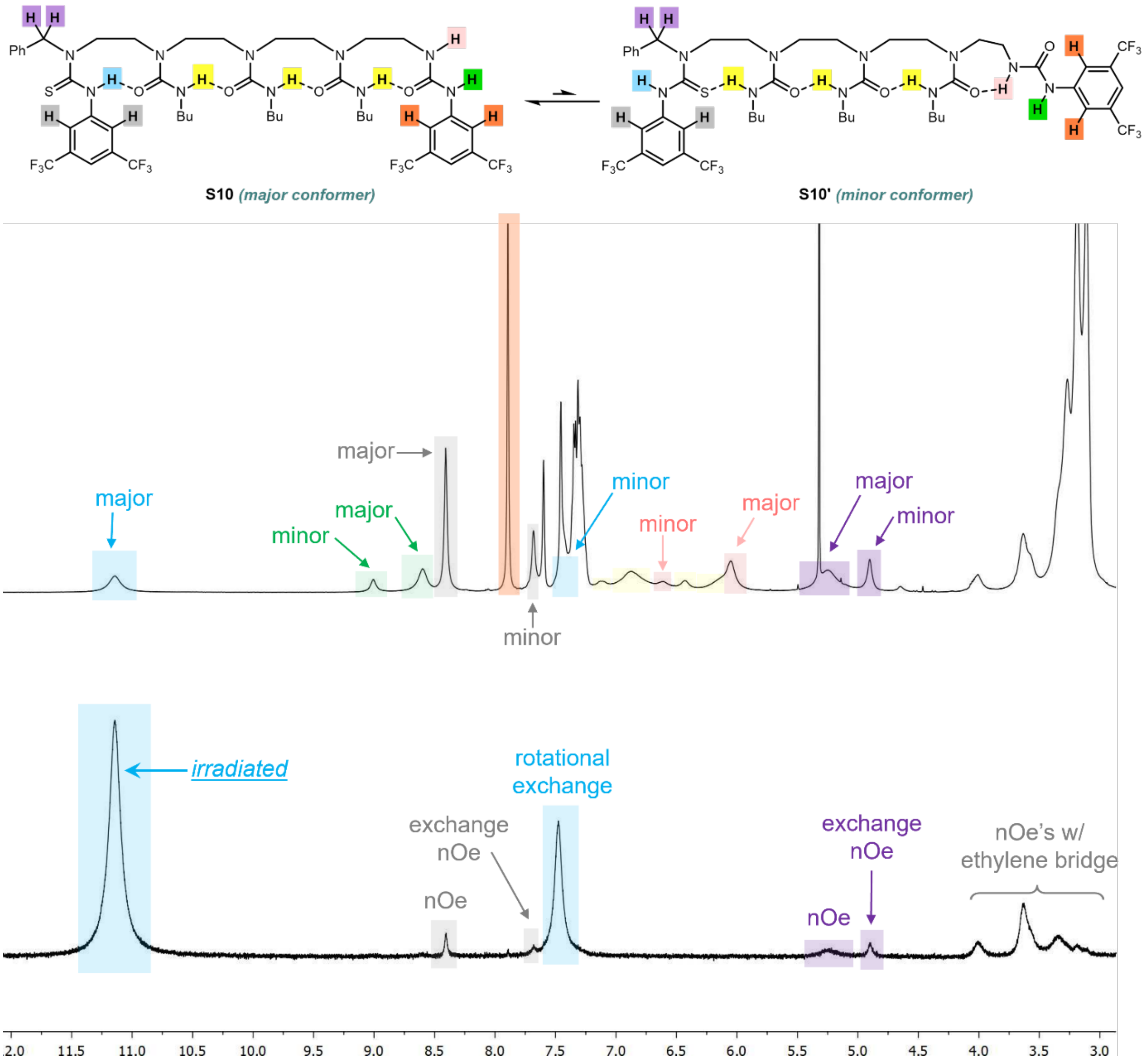

Figure S16a – Selective one-dimensional NOESY experiment for compound S10 at −10 °C (500 MHz, 25 mM, CD_2Cl_2). (Upper) ^{1}H NMR spectrum at −10 °C (for reference); (Lower) Irradiation of the thiourea proton of the major conformer (δ_H = 11.14 ppm, coloured blue). Signals arising from rotational exchange and through space nOes appear in the same phase, indicating that the nOes are negative. 'Exchange nOes' are also observed as a result of excitation transfer between the two conformers due to rotational exchange occurring on the timescale of the nOe build-up.

Table S1 – Summary of the conformational populations of thioureas 1, 7 and S10 under various conditions. All ratios were measured at −10 °C. The capture and release experiments shown in the manuscript essentially involve the 'major' conformer of **1** and **7** (i.e., conformer **X**, drawn on the left of the equilibrium below). As such, we investigated the effect of various parameters on the conformational population of **X**. The results show that the length of the oligomer (entry 1 *versus* entry 2), the identity of the internal ureas (entry 1 *versus* entry 3) and the concentration (entries 1, 4 and 8) have little effect on the conformer ratio, favouring **X** in all cases. Adding the ligand (Bu_3PO) at the same concentration as the capture and release experiments further shifts the equilibrium in favour of **X** (entry 4 *versus* entry 5), consistent with the proposed binding at the BTMP urea. Finally, the conformer ratio was found to be dependent on the solvent (entries 6−11): an almost equal population of the two conformers was observed in $CDCl_3$ (entry 7), while **X** was strongly favoured by more polar solvents and/or solvents with hydrogen bond-accepting heteroatoms (entries 9−11).

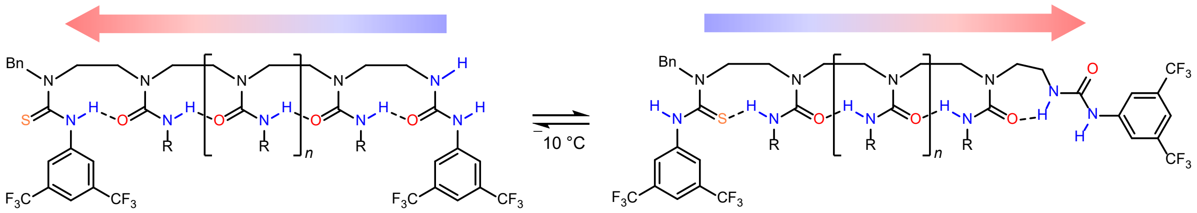

Entry	Compound	*n*	R	Conc.	Solvent	Ratio X:X'	Figure Reference
1	**1**	1	4-butoxyphenyl	25 mM	CD_2Cl_2	75:25	Fig. S1
2	**7**	3	4-butoxyphenyl	25 mM	CD_2Cl_2	74:26	Fig. S12
3	**S10**	1	butyl	25 mM	CD_2Cl_2	72:28	Fig. S16
4	**1**	1	4-butoxyphenyl	10 mM	CD_2Cl_2	73:27	Fig. S7b
5	**1**	1	4-butoxyphenyl	10 mM[a]	CD_2Cl_2	82:18	Fig. S7c
6	**1**	1	4-butoxyphenyl	2.5 mM	toluene-d8	nd[b]	Fig. S7d
7	**1**	1	4-butoxyphenyl	2.5 mM	$CDCl_3$	48:52	Fig. S7e
8	**1**	1	4-butoxyphenyl	2.5 mM	CD_2Cl_2	81:19	Fig. S7f
9	**1**	1	4-butoxyphenyl	2.5 mM	THF-d8	91:9	Fig. S7g
10	**1**	1	4-butoxyphenyl	2.5 mM	acetone-d6	>95:5	Fig. S7h
11	**1**	1	4-butoxyphenyl	2.5 mM	MeCN-d3[c]	>95:5	Fig. S7i

[a]Bu_3PO (2 mM) was also present. [b]Broad, unresolved signals were observed. [c]Partial precipitation was observed upon cooling in the NMR spectrometer.

Titrations, Binding Constants and Related Experiments

General Points

- ***^{31}P NMR parameters for titrations:*** proton decoupled [$^{31}P\{^{1}H\}$], CH_2Cl_2, 162 MHz, 128 scans, Bruker spectrometer; 'non-deuterated CH_2Cl_2' was selected as the solvent.

- A 150 mM solution of Ph_3PO in CH_2Cl_2 was used as external standard by placing a capillary tube containing this solution in the NMR tube; capillary tube dimensions (L x I.D. x O.D.) = 100 mm x 0.95 mm x 1.35 mm (pre-sealed at the bottom and, after addition of the Ph_3PO solution, sealed at the top using a Bunsen burner). Note that the height of the external standard solution in the capillary tube should be lower (by *ca* 20%) than the height of the sample solution in the NMR tube to avoid significant broadening of the external standard signal.

- The titrant solutions (containing the disubstituted urea ligand at 20 mM in CH_2Cl_2) were stored sealed in the fridge between additions, and a new line was marked at the solvent level after each aliquot was taken to check/ensure no solvent loss occurred between additions.

- ***Binding constants (K)*** for **1−3** with Bu_3PO in CH_2Cl_2 were determined using Bindfit (supramolecular.org) with a 1:1 binding model.[4]

***General procedure for ^{31}P NMR titrations**:* A solution of Bu_3PO (2.0 mM in CH_2Cl_2, 0.50 mL, 1.0 μmol, 1.0 equiv) was added to an NMR tube and a sealed capillary tube containing Ph_3PO (150 mM in CH_2Cl_2) was placed inside. The solvent level in the NMR tube (containing the capillary tube) was marked on a separate NMR tube for reference, then the $^{31}P\{^{1}H\}$ NMR spectrum was recorded to obtain the chemical shifts of 'free' Bu_3PO and the Ph_3PO external standard [δ_P Bu_3PO = 47.23 ppm;[5] δ_P Ph_3PO = 27.67 ppm; all subsequent spectra were referenced to the external standard at 27.67 ppm]. An aliquot of the appropriate ligand solution (**1**, **2**, **3**, (**3**+**4**)* or (**3**+**5**)**, 20 mM in CH_2Cl_2, 25 μL, 0.5 μmol, 0.5 equiv) was added via a Gilson pipette, followed by a small amount of CH_2Cl_2 (~0.1 mL) to rinse the upper interior of the NMR tube. The volume in the NMR tube was then re-adjusted to the original level by blowing N_2 into the tube using a 120 mm length, 21-gauge needle attached to a N_2-filled balloon. During this process, any moisture building up on the exterior of the NMR tube (due to endothermic evaporation) was removed with paper towel. The ^{31}P NMR spectrum was again recorded. Aliquots of the titrant were then added sequentially using the same procedure and the ^{31}P NMR spectrum was each time recorded. When the amount of ligand present reached a total of 5.0 equiv, the volume of the added titrant (containing 20 mM of the ligand in CH_2Cl_2) was increased to 50 μL so that a further 1.0 equiv of the ligand was introduced during each addition (up to a total of 9.0 equiv), again adjusting the solvent

volume as described to ensure a constant concentration of Bu_3PO (2.0 mM). [*Contained 20 mM of **3** and 20 mM of **4**; **Contained 20 mM of **3** and 20 mM of **5**.]

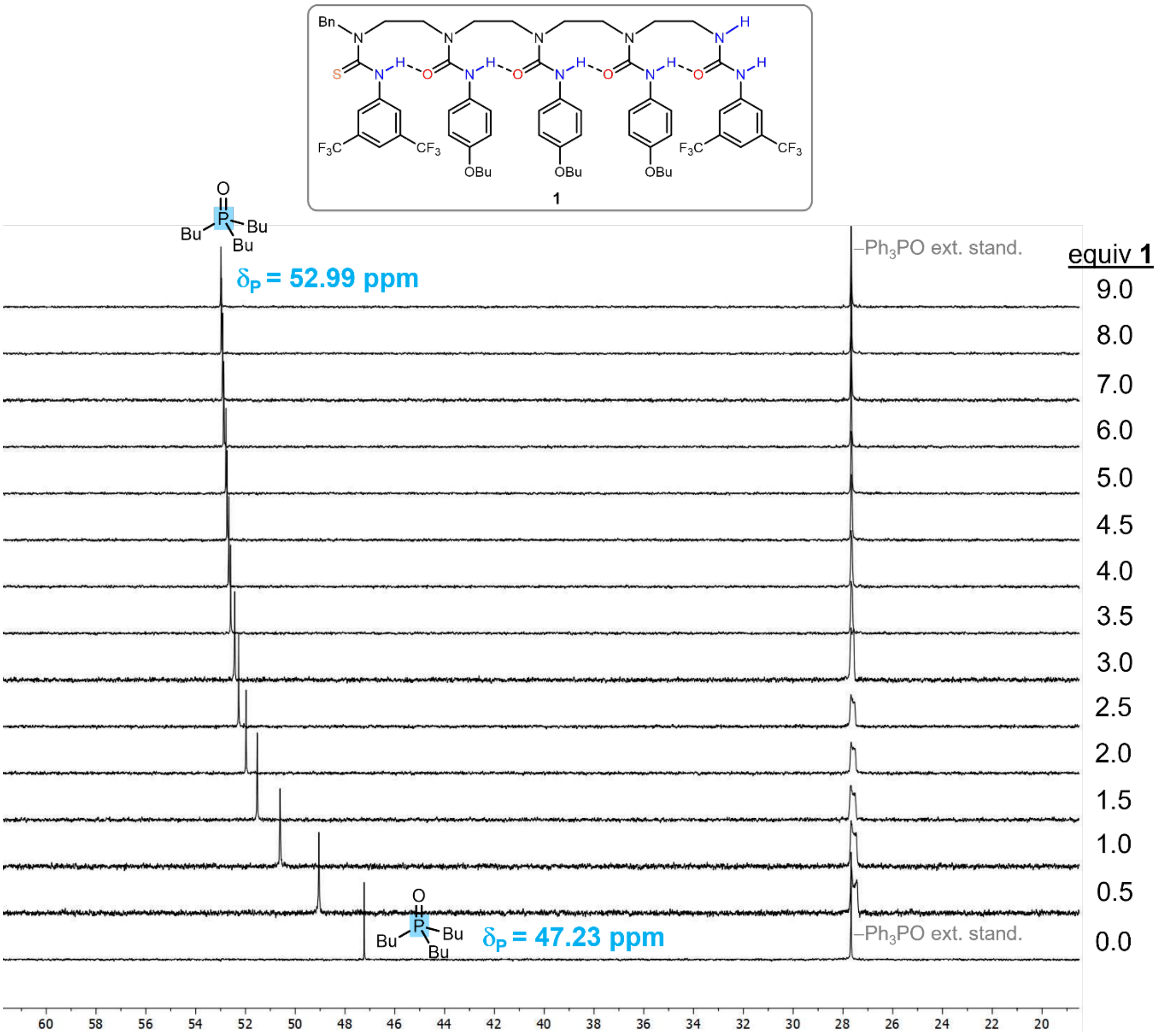

Figure S16 – Titration of Bu_3PO (2 mM) with 1 (0–9 equiv) as monitored by $^{31}P\{^1H\}$ NMR spectroscopy (162 MHz, CH_2Cl_2).

Table S2 – δ_P of Bu_3PO (2 mM, CH_2Cl_2) with increasing concentration of 1.

**concentration Bu_3PO** (molL^{-1})	**concentration 1** (molL^{-1})	δ_p (ppm)	$\Delta\delta_P$ (ppm)
0.002	0.000	47.23	0.00
0.002	0.001	49.05	1.82
0.002	0.002	50.61	3.38
0.002	0.003	51.53	4.30
0.002	0.004	51.98	4.75
0.002	0.005	52.27	5.04
0.002	0.006	52.43	5.20
0.002	0.007	52.59	5.36
0.002	0.008	52.67	5.44
0.002	0.009	52.74	5.51
0.002	0.010	52.78	5.55
0.002	0.012	52.87	5.64
0.002	0.014	52.91	5.68
0.002	0.016	52.96	5.73
0.002	0.018	52.99	5.76

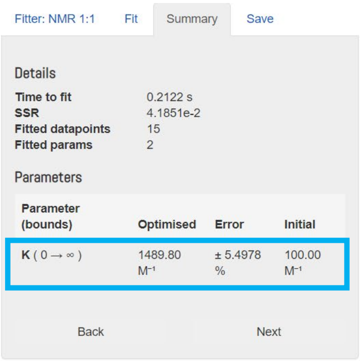

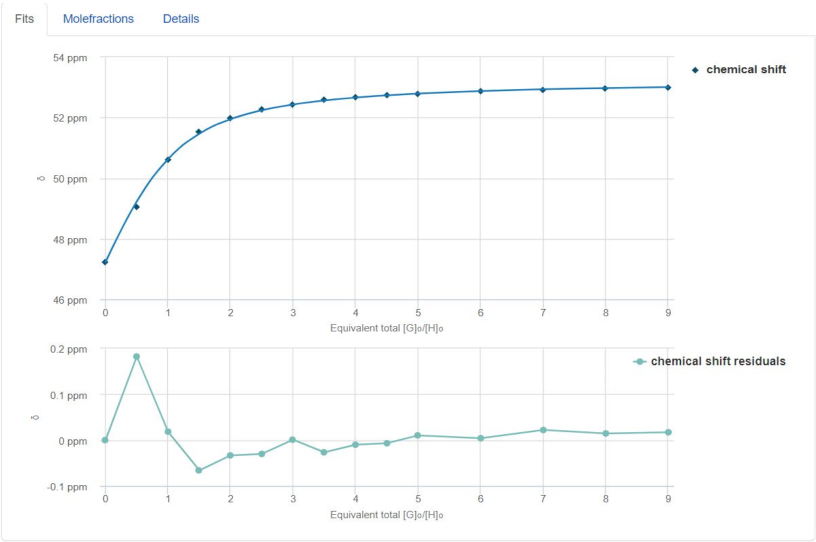

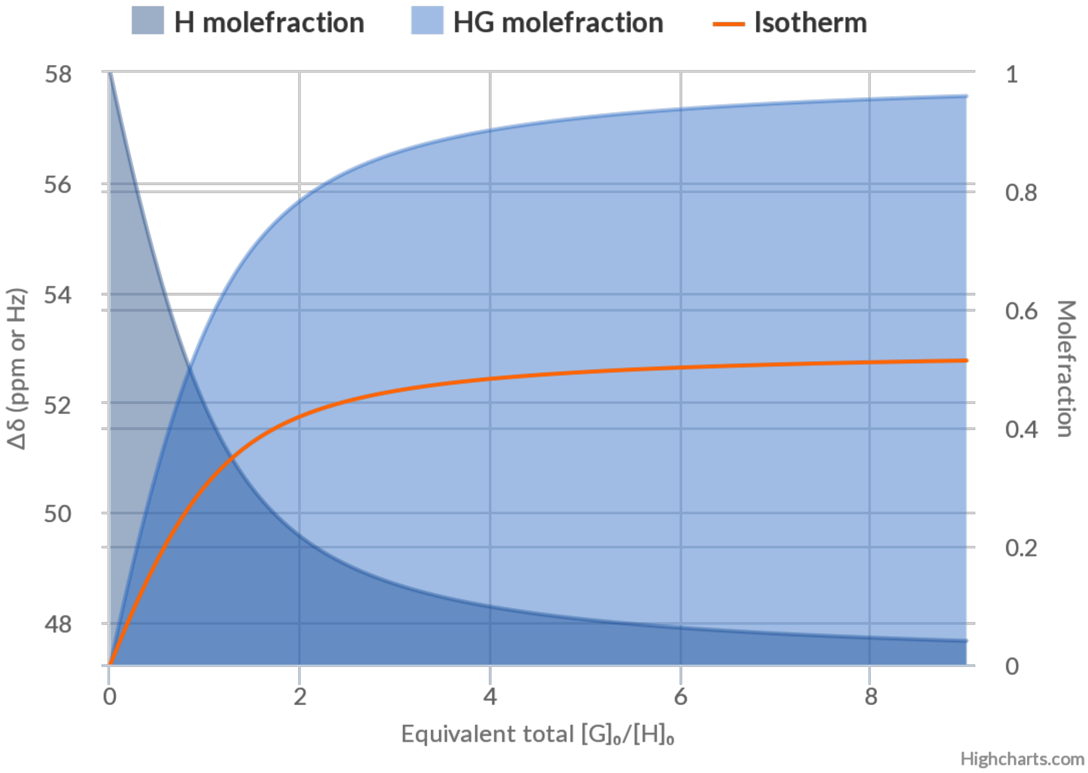

Figure S17 – Determination of the binding constant (*K*) of Bu_3PO with 1 in CH_2Cl_2. These results were obtained using Bindfit (supramolecular.org) with a 1:1 binding model. Fit details available at http://app.supramolecular.org/bindfit/view/17f96abe-2b42-4b99-adb9-6cfca405e124.

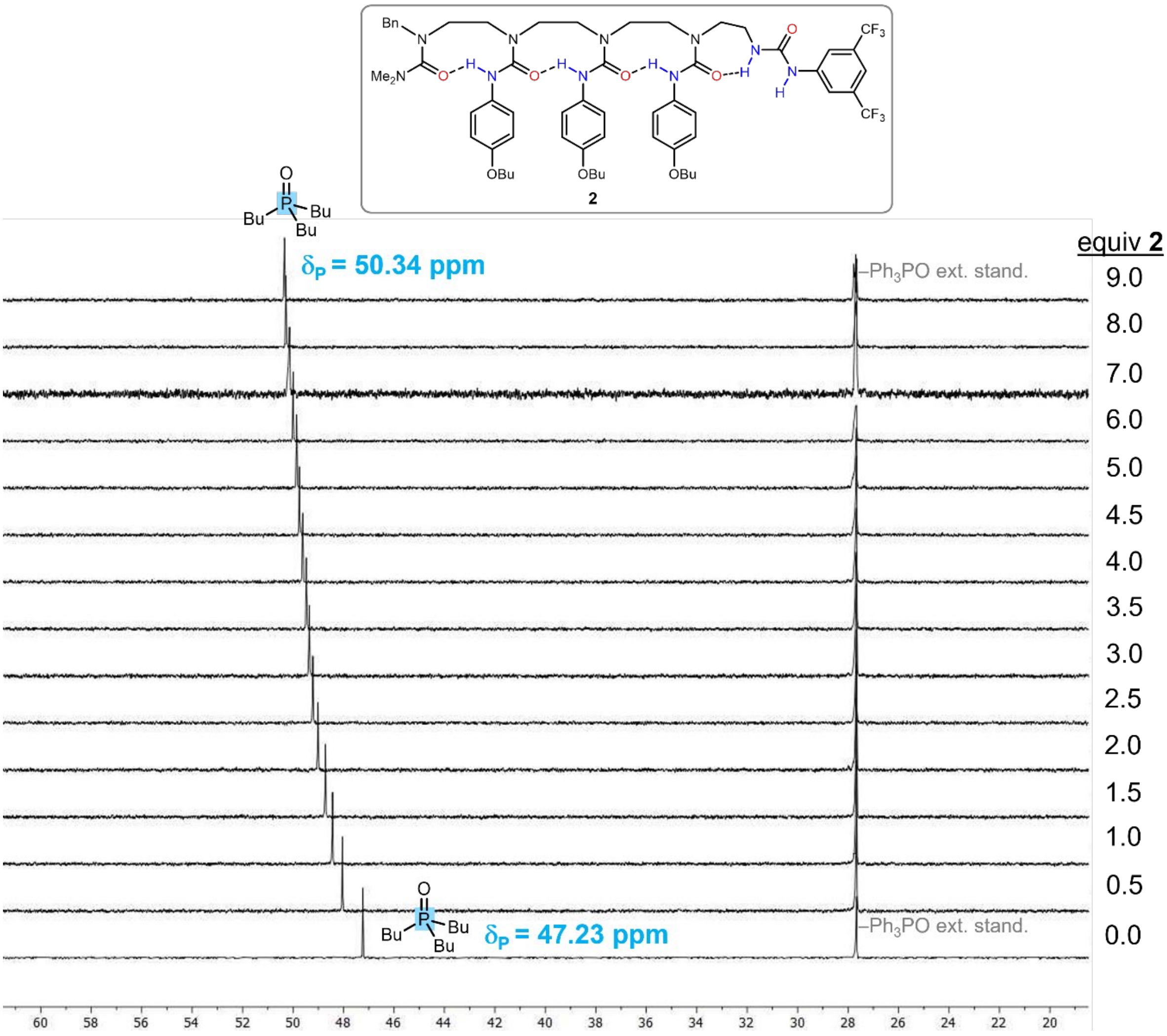

Figure S18 – Titration of Bu_3PO (2 mM) with 2 (0–9 equiv) as monitored by $^{31}P\{^1H\}$ NMR spectroscopy (162 MHz, CH_2Cl_2).

Table S3 – δ_P of Bu_3PO (2 mM, CH_2Cl_2) with increasing concentration of 2.

**concentration Bu_3PO** ($molL^{-1}$)	**concentration 2** ($molL^{-1}$)	δ_p (ppm)	$\Delta\delta_P$ (ppm)
0.002	0.000	47.23	0.00
0.002	0.001	48.04	0.81
0.002	0.002	48.44	1.21
0.002	0.003	48.72	1.49
0.002	0.004	49.01	1.78
0.002	0.005	49.22	1.99
0.002	0.006	49.36	2.13
0.002	0.007	49.47	2.24
0.002	0.008	49.62	2.39
0.002	0.009	49.75	2.52
0.002	0.010	49.86	2.63
0.002	0.012	50.00	2.77
0.002	0.014	50.14	2.91
0.002	0.016	50.28	3.05
0.002	0.018	50.34	3.11

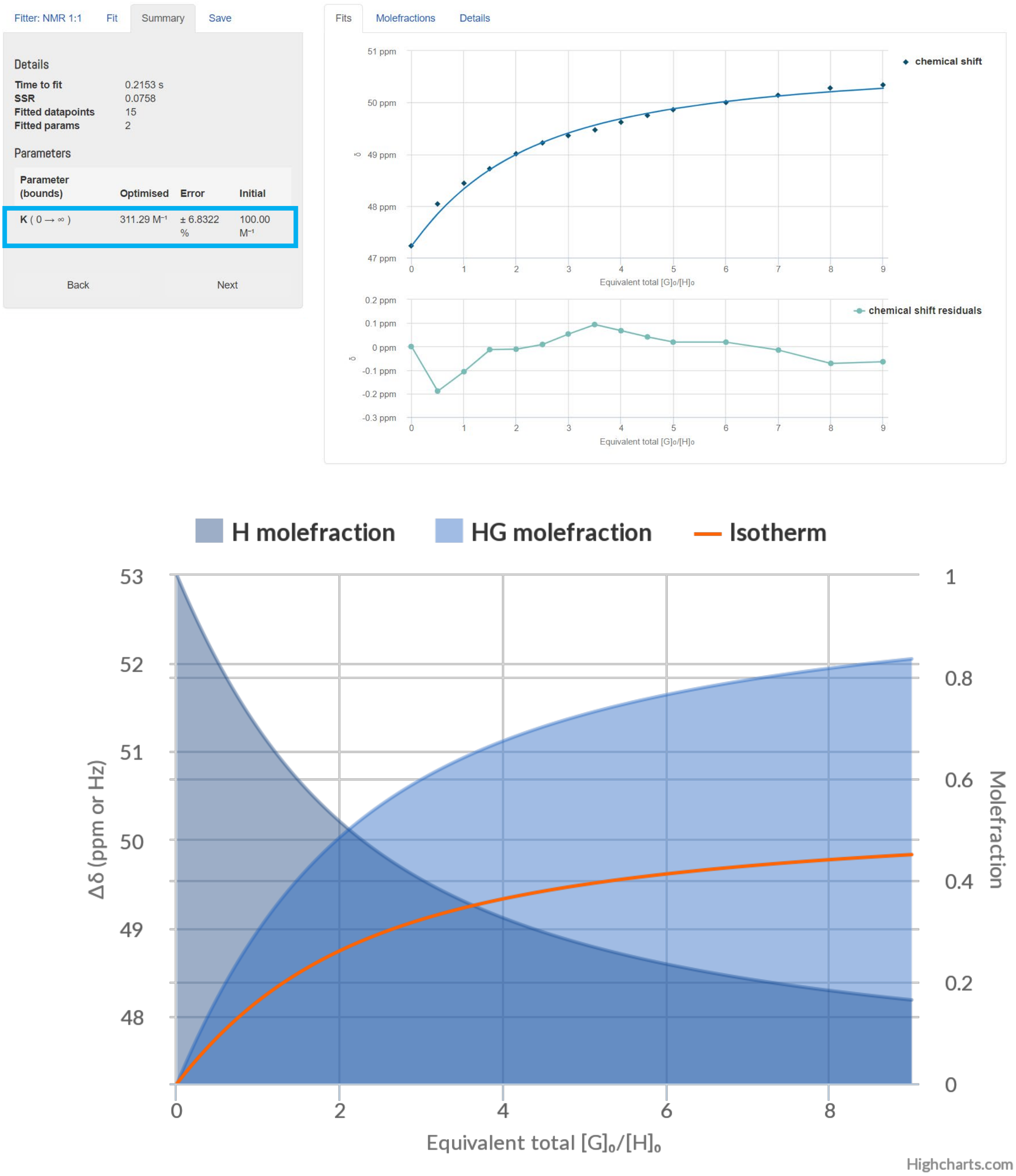

Figure S19 – Determination of the binding constant (*K*) of Bu_3PO with 2 in CH_2Cl_2. These results were obtained using Bindfit (supramolecular.org) with a 1:1 binding model. Fit details available at http://app.supramolecular.org/bindfit/view/60263b23-2d00-4e27-82da-9237c8ba1127.

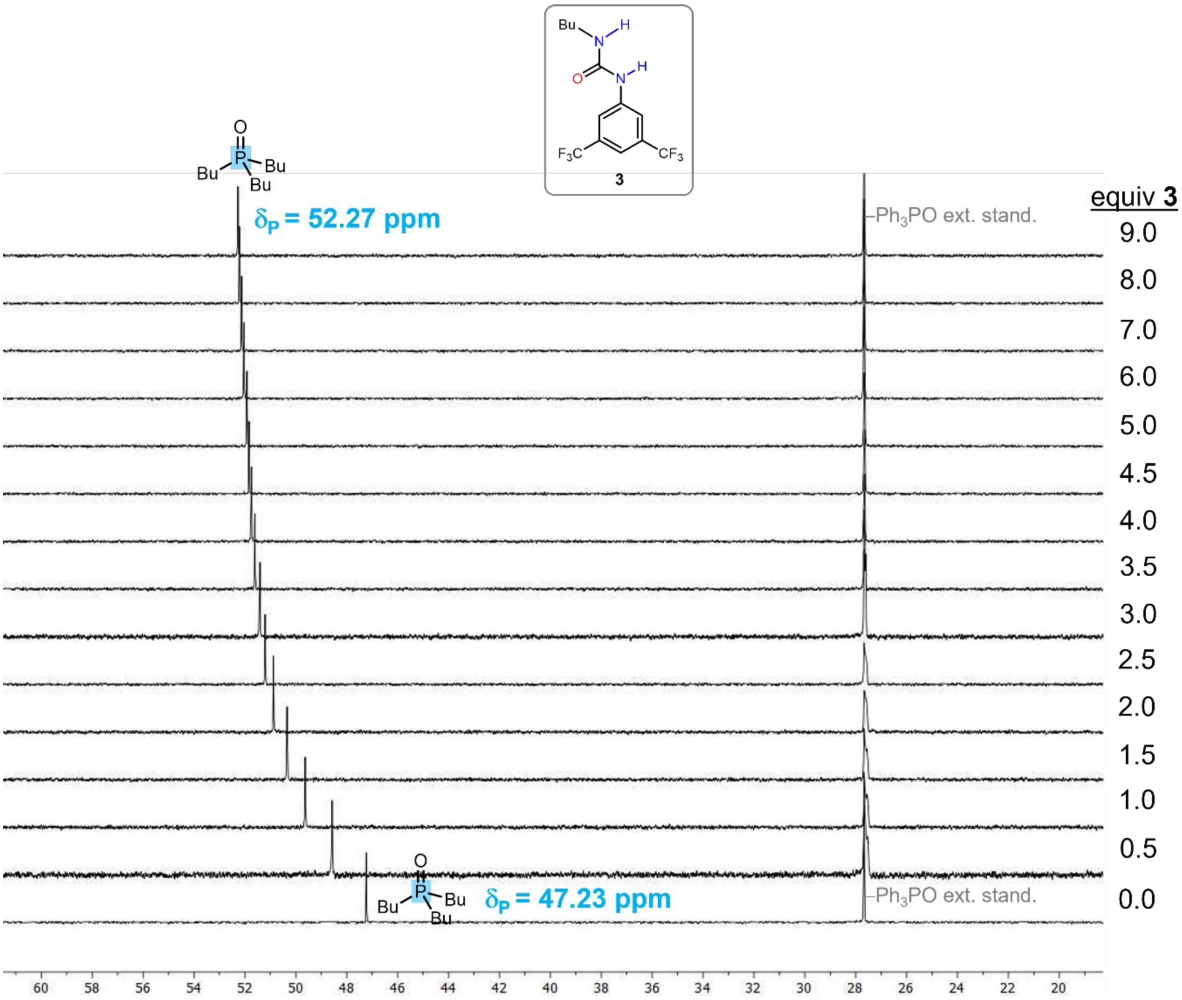

Figure S20 – Titration of Bu_3PO (2 mM) with 3 (0–9 equiv) as monitored by $^{31}P\{^1H\}$ NMR spectroscopy (162 MHz, CH_2Cl_2).

Table S4 – δ_P of Bu_3PO (2 mM, CH_2Cl_2) with increasing concentration of 3.

**concentration Bu_3PO** (molL^{-1})	**concentration 3** (molL^{-1})	δ_p (ppm)	$\Delta\delta_P$ (ppm)
0.002	0.000	47.23	0.00
0.002	0.001	48.57	1.34
0.002	0.002	49.63	2.40
0.002	0.003	50.35	3.12
0.002	0.004	50.88	3.65
0.002	0.005	51.20	3.97
0.002	0.006	51.41	4.18
0.002	0.007	51.61	4.38
0.002	0.008	51.75	4.52
0.002	0.009	51.84	4.61
0.002	0.010	51.93	4.70
0.002	0.012	52.04	4.81
0.002	0.014	52.13	4.90
0.002	0.016	52.22	4.99
0.002	0.018	52.27	5.04

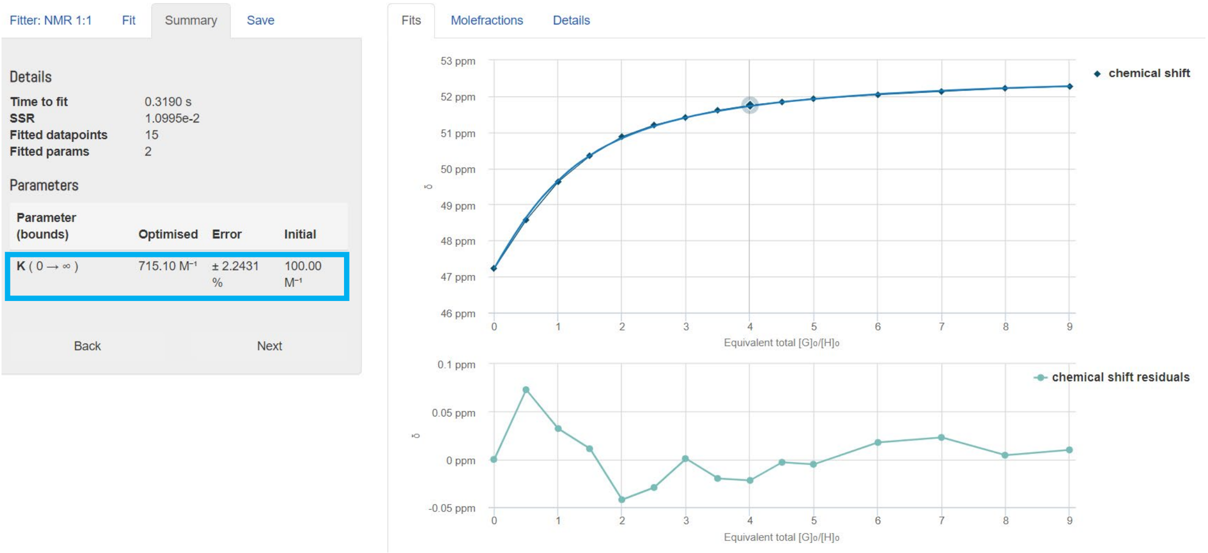

Figure S21 – Determination of the binding constant (*K*) of Bu_3PO with 3 in CH_2Cl_2. These results were obtained using Bindfit (supramolecular.org) with a 1:1 binding model. Fit details available at http://app.supramolecular.org/bindfit/view/dee1a1ec-c880-497a-8d71-15c68c0ef1bf.

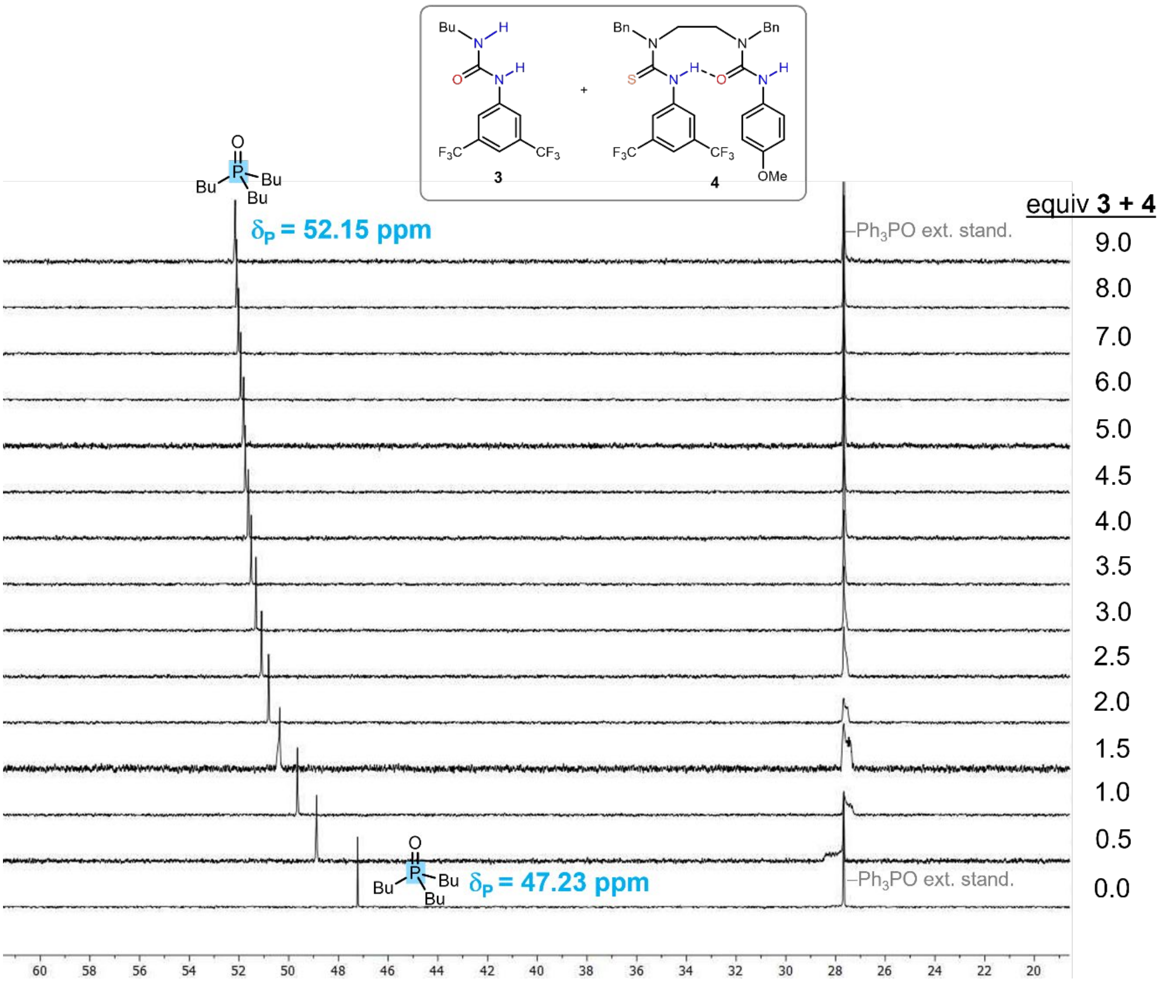

Figure S22 – Titration of Bu_3PO (2 mM) with 3+4 (0–9 equiv of each) as monitored by $^{31}P\{^1H\}$ NMR spectroscopy (162 MHz, CH_2Cl_2). The titrant consists of a mixture of **3** and **4** in a 1:1 molar ratio – the column to the right of the NMR stack shows the equivalents of both **3** and **4** present at each titration point.

Table S5 – δ_P of Bu_3PO (2 mM, CH_2Cl_2) with increasing concentrations of 3 and 4.

**concentration Bu_3PO** (molL^{-1})	**concentration 3 and 4** (molL^{-1})	δ_p (ppm)	$\Delta\delta_P$ (ppm)
0.002	0.000	47.23	0.00
0.002	0.001	48.88	1.65
0.002	0.002	49.65	2.42
0.002	0.003	50.36	3.13
0.002	0.004	50.81	3.58
0.002	0.005	51.09	3.86
0.002	0.006	51.32	4.09
0.002	0.007	51.51	4.28
0.002	0.008	51.63	4.40
0.002	0.009	51.75	4.52
0.002	0.010	51.82	4.59
0.002	0.012	51.94	4.71
0.002	0.014	52.03	4.80
0.002	0.016	52.10	4.87
0.002	0.018	52.15	4.92

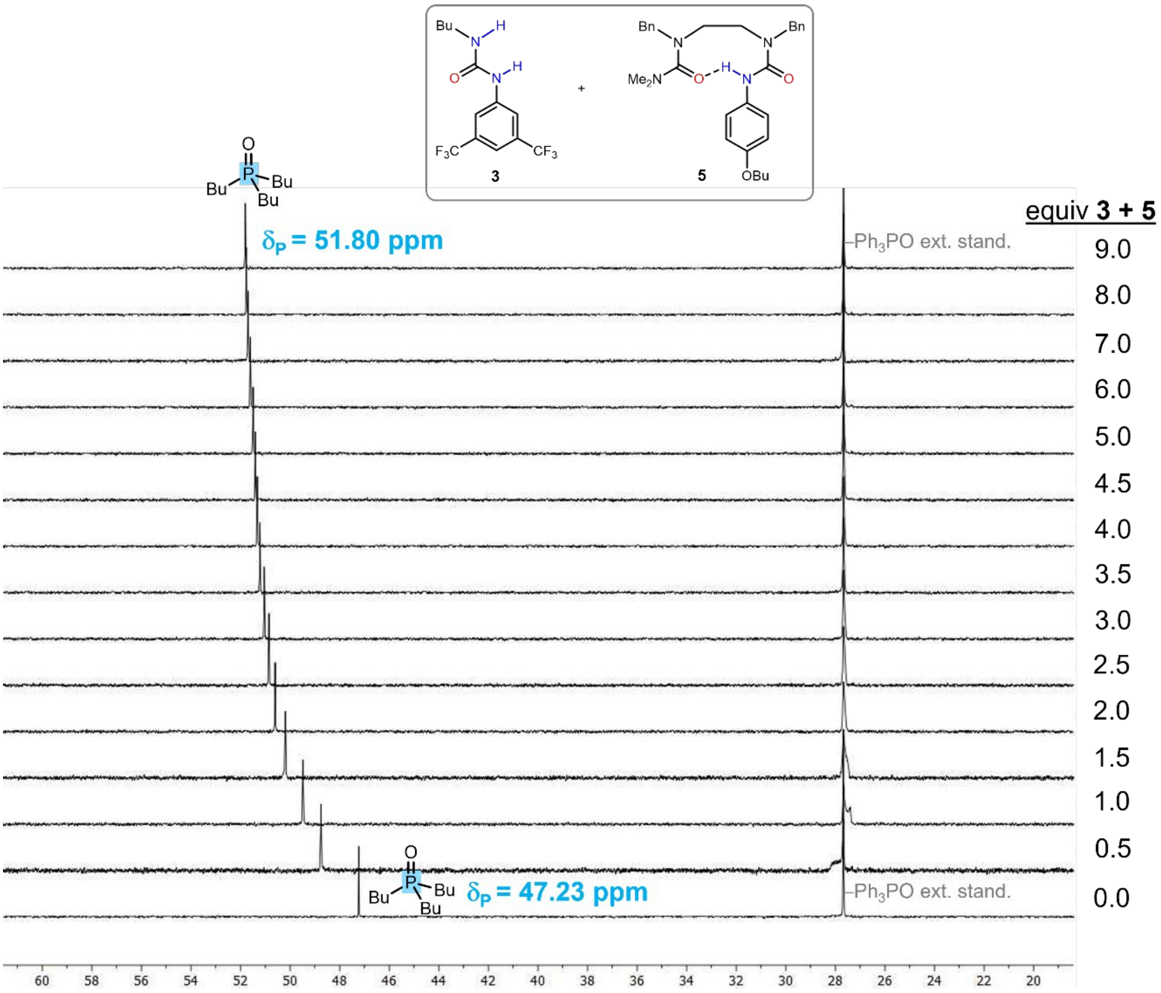

Figure S23 – Titration of Bu_3PO (2 mM) with 3+5 (0–9 equiv of each) as monitored by $^{31}P\{^1H\}$ NMR spectroscopy (162 MHz, CH_2Cl_2). The titrant consists of a mixture of **3** and **5** in a 1:1 molar ratio – the column to the right of the NMR stack shows the equivalents of both **3** and **5** present at each titration point.

Table S6 – δ_P of Bu_3PO (2 mM, CH_2Cl_2) with increasing concentrations of 3 and 5.

**concentration Bu_3PO** (molL^{-1})	**concentration 3 and 5** (molL^{-1})	**δ_p** (ppm)	**$\Delta\delta_P$** (ppm)
0.002	0.000	47.23	0.00
0.002	0.001	48.75	1.52
0.002	0.002	49.48	2.25
0.002	0.003	50.19	2.96
0.002	0.004	50.60	3.37
0.002	0.005	50.85	3.62
0.002	0.006	51.04	3.81
0.002	0.007	51.21	3.98
0.002	0.008	51.32	4.09
0.002	0.009	51.40	4.17
0.002	0.010	51.49	4.26
0.002	0.012	51.60	4.37
0.002	0.014	51.69	4.46
0.002	0.016	51.76	4.53
0.002	0.018	51.80	4.57

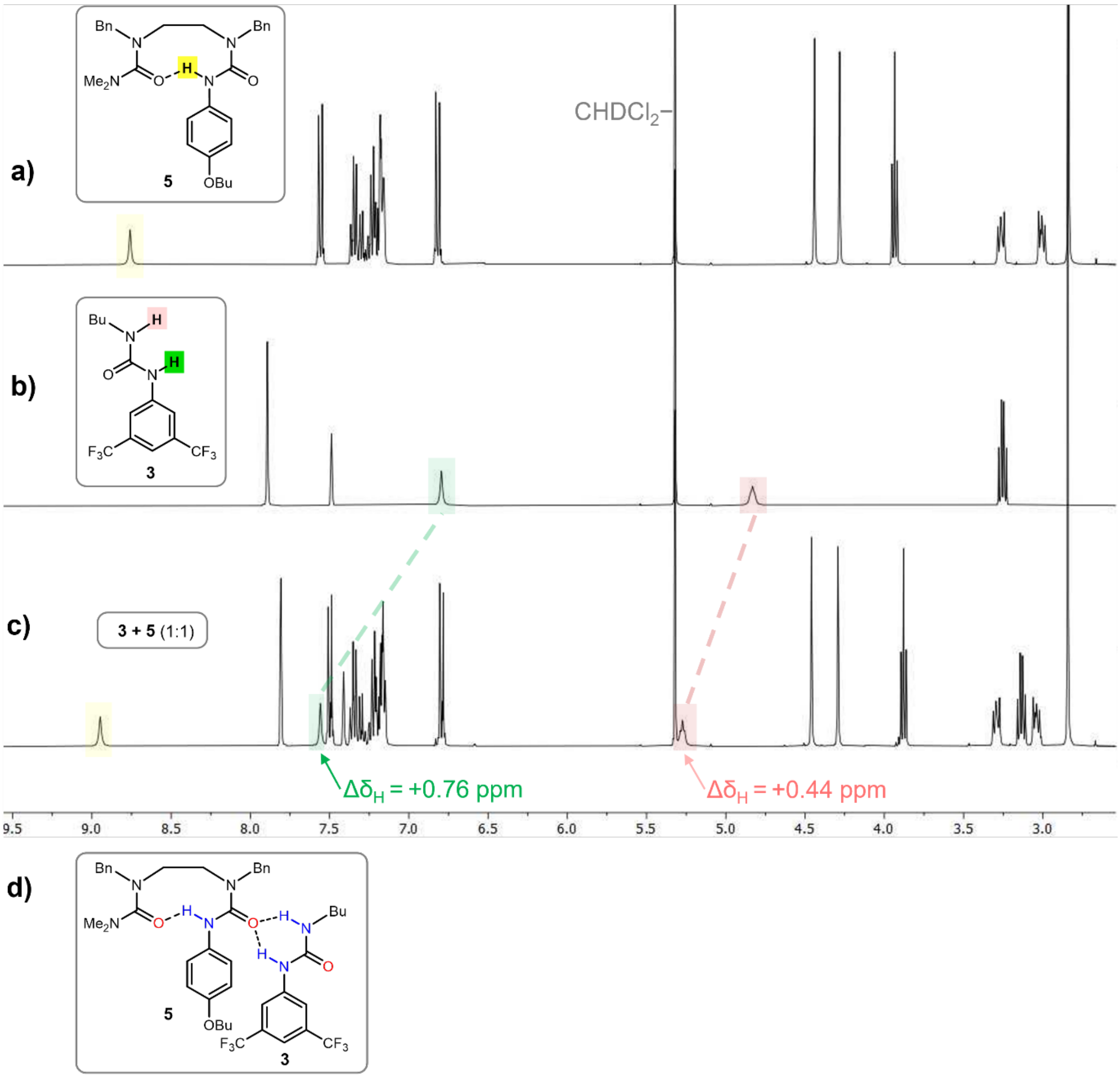

Figure S24 – Evidence of hydrogen bonding between 3 and 5. (a) ^{1}H NMR spectrum of compound 5 (400 MHz, 10 mM, CD_2Cl_2); (b) ^{1}H NMR spectrum of compound 3 (400 MHz, 10 mM, CD_2Cl_2); (c) ^{1}H NMR spectrum of 3 and 5 combined in a 1:1 molar ratio (400 MHz, both 10 mM, CD_2Cl_2); (d) Proposed intermolecular hydrogen bonding between the disubstituted urea in 3 and the available urea carbonyl group of 5.

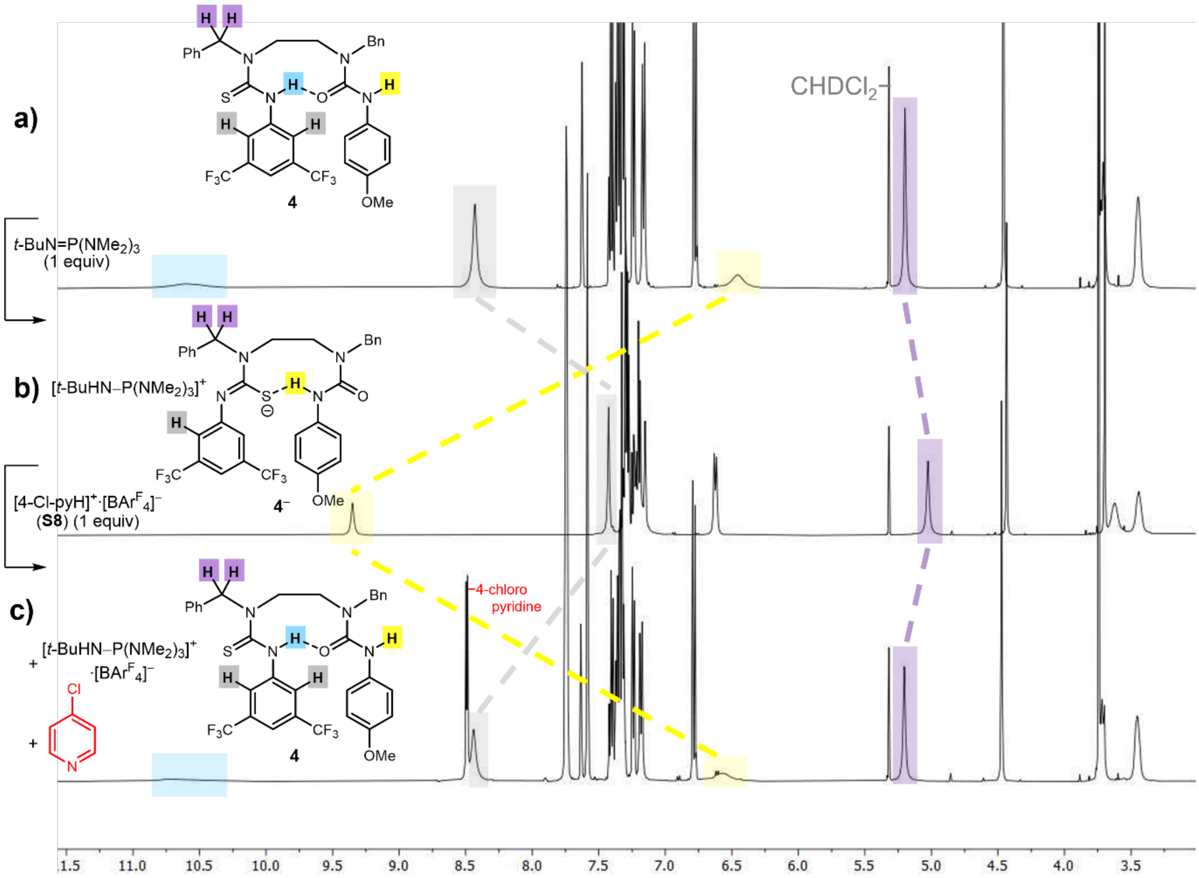

Figure S25 – Reversible deprotonation and polarity switching of thiourea transmitter 4. (a) ^{1}H NMR spectrum of 4 (500 MHz, 42 mM, CD_2Cl_2) and (b) with the addition of *t*-BuN=P(NMe$_2$)$_3$ (1 equiv) to the same sample to deprotonate the thiourea. (c) Re-protonation after addition of [4-Cl-pyH]$^{+}$·[BAr$^{F}_{4}$]$^{–}$ (1 equiv) to the same sample. Note that this Figure is an expansion of Figure 3 in the manuscript.

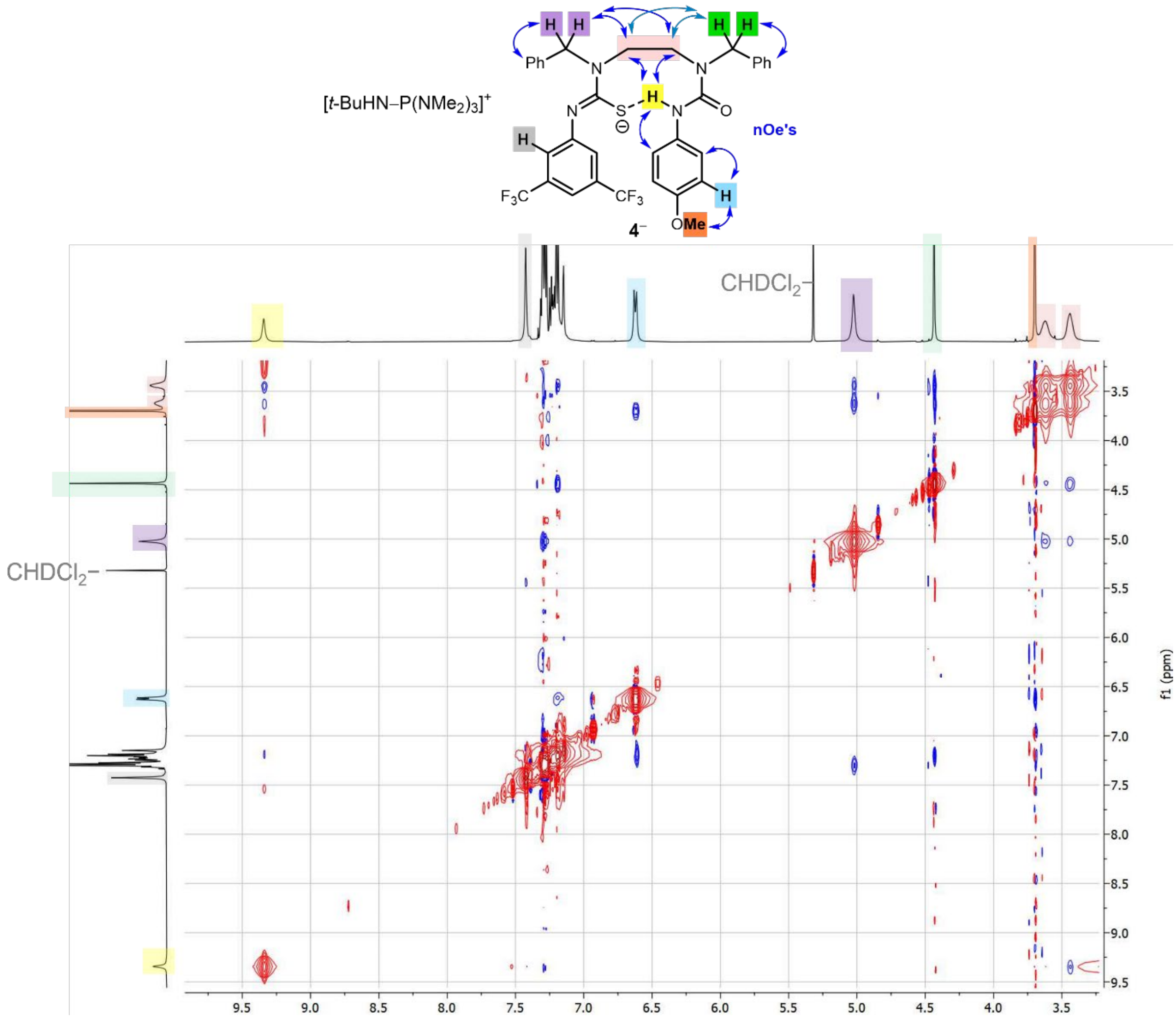

Figure S25d – NOESY spectrum of [*t*-BuHN−P(NMe$_2$)$_3$]$^+$·4$^-$ at 25 °C (500 MHz, 40 mM, CD$_2$Cl$_2$).

An alternative orientation of **4**$^-$ was also considered, where the N-aryl nitrogen of the thiourea points inwards and hydrogen bonds to the adjacent urea NH instead of the sulfur atom:

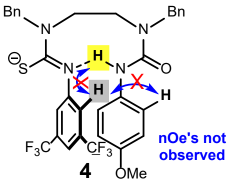

In this alternative orientation, an NOE would likely be expected between the urea NH (colored yellow) and the BTMP thiourea *ortho*-aryl protons (colored grey), as well as between the *ortho* protons on each of the two N-aryl rings; however, no appreciable NOE was observed in either case. These results tentatively suggest that it is the sulfur that points inwards and hydrogen bonds to the adjacent urea proton − a situation that would also relieve steric interactions between the N-aryl rings.

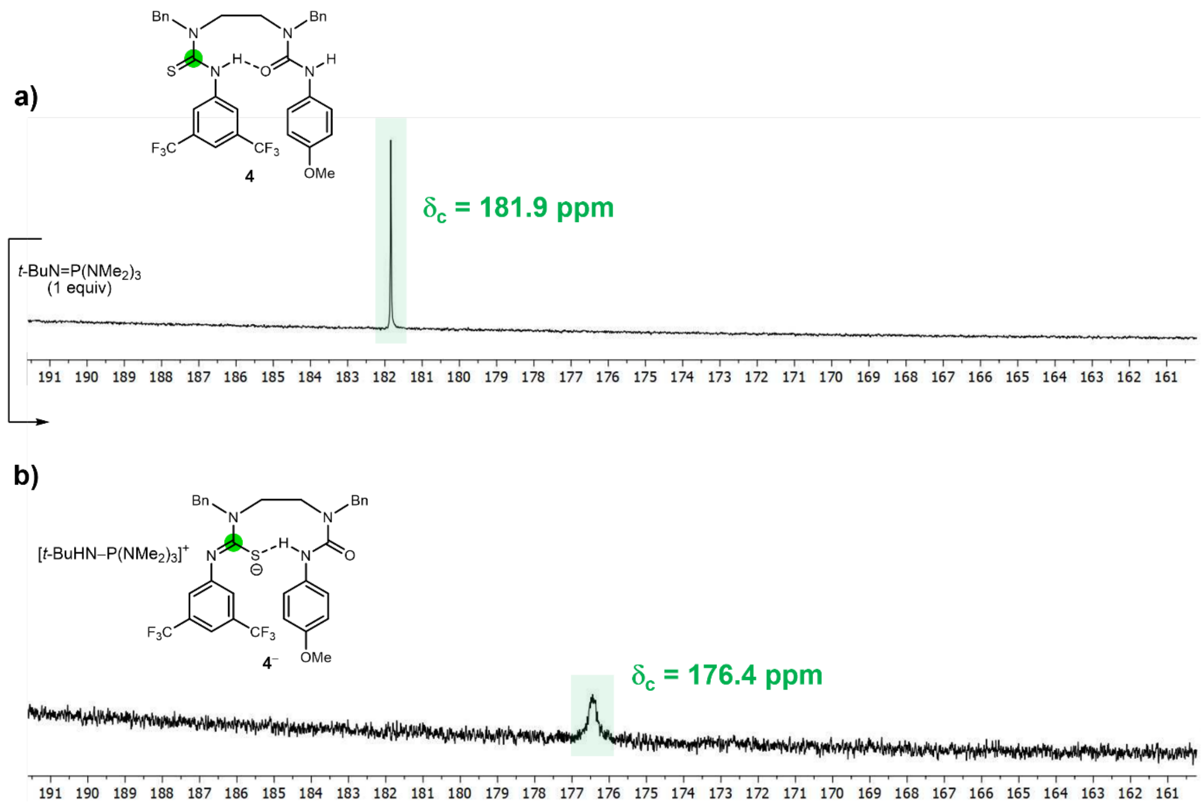

Figure S26 – Accompanying ^{13}C NMR evidence for the deprotonation of thiourea transmitter 4. (a) Portion of the ^{13}C NMR spectrum of 4 (126 MHz, 42 mM, CD_2Cl_2) showing the thiocarbonyl signal and (b) with the addition of *t*-BuN=P(NMe_2)$_3$ (1 equiv) to the same sample.

General Points for ^{31}P NMR Capture-and-Release Experiments

- All ^{31}P NMR experiments: proton decoupled [$^{31}P\{^1H\}$], CD_2Cl_2, 162 MHz, 192 scans, Bruker spectrometer.

- A 150 mM solution of Ph_3PO in CD_2Cl_2 was used as external standard by placing a capillary tube containing this solution in the NMR tube; capillary tube dimensions (L x I.D. x O.D.) = 100 mm x 0.95 mm x 1.35 mm (pre-sealed at the bottom and, after addition of the Ph_3PO solution, sealed at the top using a Bunsen burner). Note that the height of the external standard solution in the capillary tube should be lower (by *ca* 20%) than the height of the sample solution in the NMR tube to avoid significant broadening of the external standard signal.

- ^1H NMR spectra were also acquired for every experiment to confirm correct stoichiometry and, where applicable, chemoselective thiourea deprotonation; hence, why CD_2Cl_2 was used as the ^{31}P NMR solvent instead of CH_2Cl_2 (^1H NMR spectra were also acquired with the Ph_3PO external standard capillary in the NMR tube).

• The major indicators of deprotonation of the thiourea function in **1**, **4**, **6** and **7** with *t*-BuN=P(NMe$_2$)$_3$ were the loss of the thiourea NH signal at *ca* 11 ppm in the ^{1}H NMR spectrum, as well as a new signal in the ^{31}P NMR spectrum (in addition to Bu$_3$PO) corresponding to [*t*-BuHN−P(NMe$_2$)$_3$]$^+$·**X**$^-$ at δ_P = 32−35 ppm (**X** = **1**, **4**, **6** or **7**). For comparison, the conjugate base *t*-BuN=P(NMe$_2$)$_3$ was found to have δ_P = 7.05 ppm under the same conditions (Figure S28).

• The ^{31}P NMR chemical shift (δ_P) of the ion pairs [*t*-BuHN−P(NMe$_2$)$_3$]$^+$·**X**$^-$ (where **X** = **1**, **4**, **6** and **7**; δ_P = 32−35 ppm) matched closely with that of an in-house prepared tetraarylborate analogue [*t*-BuHN−P(NMe$_2$)$_3$]$^+$·[BAr$^F{}_4$]$^-$ (**S9**) characterized under the same conditions (δ_P = 34.71 ppm; Figure S29).

• Although no attempts have been made to isolate the [*t*-BuHN−P(NMe$_2$)$_3$]$^+$ salts of thiourea anions **1**$^-$, **4**$^-$, **6**$^-$ or **7**$^-$, we have seen no evidence of sensitivity to air or adventitious moisture during the timeframes of NMR analysis of their CD$_2$Cl$_2$ solutions. We note, however, that they do undergo slow alkylation at sulfur by the solvent itself (CD$_2$Cl$_2$). For this reason, all ^{1}H and ^{31}P NMR spectra of these anions (**1**$^-$, **4**$^-$, **6**$^-$ or **7**$^-$) were acquired within 30 min of adding the base (*t*-BuN=P(NMe$_2$)$_3$). Subsequent re-protonation, where relevant, was then carried out immediately after recording the NMR spectra. With these precautions taken (as described in the general procedure), at most, traces of thiourea alkylation are observed. The rate of alkylation was nonetheless investigated by ^{1}H NMR using **4**$^-$ as a representative example (42 mM, CD$_2$Cl$_2$, room temperature). After 4 h, the molar ratio of **4**$^-$/ alkylation product was 4.0:1.0. After 20 h, the ratio was 1.0:1.0. After 68 h, >99% consumption of **4**$^-$ was observed; the alkylation product was isolated in 73% yield (Scheme S7).

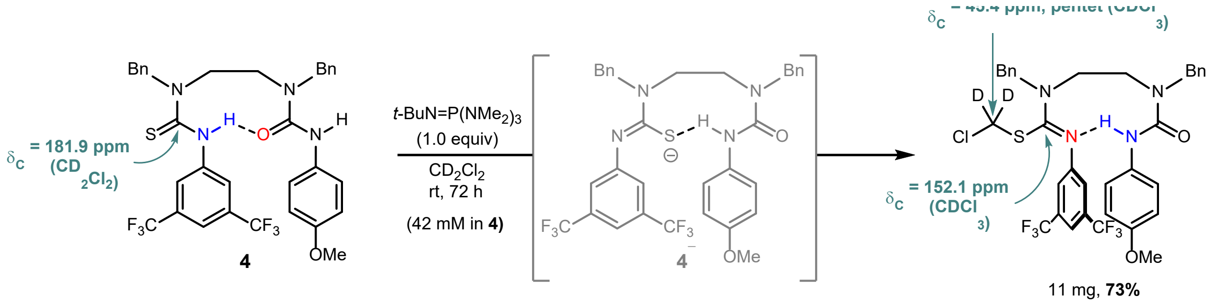

Scheme S7 – Thiourea anions are slowly alkylated at sulfur by CD$_2$Cl$_2$. The alkylation product of representative thiourea anion 4$^-$ was isolated in 73% yield after 72 h at room temperature.

***General procedure for ^{31}P NMR capture-and-release experiments and controls** (Figure 4 in manuscript):* First, the δ_P of the Ph$_3$PO external standard (relative to 'free' Bu$_3$PO; δ_P = 47.23 ppm)[5] was determined: an aliquot of Bu$_3$PO (2.0 mM in CD$_2$Cl$_2$, 0.50 mL, 1.0 µmol, 1.0 equiv) was added to an NMR tube and a sealed capillary tube containing Ph$_3$PO (150 mM in CD$_2$Cl$_2$) was placed inside. The ^{31}P{^{1}H} NMR spectrum was recorded [δ_P Bu$_3$PO = 47.23 ppm; δ_P Ph$_3$PO = 27.38 ppm; all subsequent spectra were referenced to the external standard at 27.38 ppm]. Then, for the capture-and-release studies

and controls, the appropriate ligand (**1**, **3**, (**3**+**4**)*, **4, 5**, or **6**; 5.0 µmol, 5.0 equiv) was weighed into a 1.7 mL vial. To this was added a solution of Bu_3PO (2.0 mM in CD_2Cl_2, 0.5 mL, 1.0 µmol, 1.0 equiv). The resulting solution was transferred quantitatively to an NMR tube and a sealed capillary tube containing Ph_3PO (150 mM in CD_2Cl_2) was placed inside. ^1H and $^{31}P\{^1H\}$ NMR spectra were acquired. Next, under air, a solution of *t*-BuN=P(NMe_2)$_3$ (200 mM in CD_2Cl_2, 25 µL, 5.0 µmol, 5.0 equiv) was added via a Gilson pipette (the concentration of Bu_3PO drops slightly from 2.0 mM to 1.9 mM after this step). ^1H and $^{31}P\{^1H\}$ NMR experiments were again acquired, within 30 min of adding the base. [*5.0 µmol of **3** and 5.0 µmol of **4** was used.]

Table S7 – Summary of the $^{31}P\{^{1}H\}$ NMR chemical shift (δ_P) of Bu_3PO (162 MHz, CD_2Cl_2) upon treatment with each of 1, 2, 3, (3+4), (3+5), 4, 5, 6 and 7 (5 equiv), followed by *t*-BuN=P(NMe$_2$)$_3$ (5 equiv) where applicable. Spectra of mixtures containing Bu_3PO + ligand were recorded at 2.0 mM of Bu_3PO; spectra of mixtures containing Bu_3PO + ligand + *t*-BuN=P(NMe$_2$)$_3$ were recorded at 1.9 mM in Bu_3PO.

**Ligand**	**$^{31}P\{^{1}H\}$ NMR: δ_P Bu_3PO (ppm)** *Note: δ_P free Bu_3PO = 47.23 ppm*	
	**+ Ligand** (5 equiv)	**+ Ligand** (5 equiv) **+ *t*-BuN=P(NMe$_2$)$_3$** (5 equiv)
no ligand (**Bu_3PO alone**)	**47.23**	**47.29**
[*t*-BuHN–P(NMe$_2$)$_3$]$^+$·[BAr$^F{}_4$]$^-$ (**S9**)	**47.51**	**-**
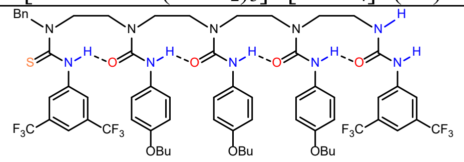 **1**	**52.66**	**48.31**
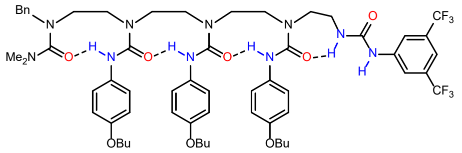 **2**	**49.86** (data obtained from titration in CH_2Cl_2)	**-**
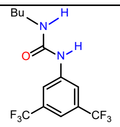 **3**	**51.99**	**51.25**
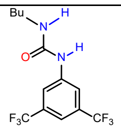 **3** + 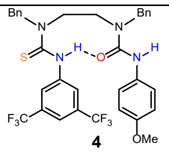 **4**	**51.81**	**50.10**
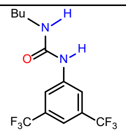 **3** + 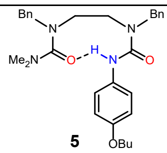 **5**	**51.49** (data obtained from titration in CH_2Cl_2)	**-**
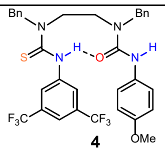 **4**	**47.60**	**47.16**
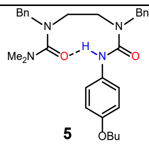 **5**	**47.51**	**-**
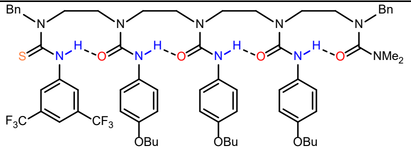 **6**	**47.27**	**47.17**
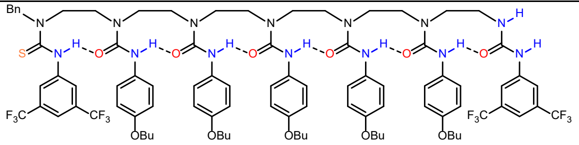 **7**	**52.51** (data from first cycle only)	**48.04** (data from first cycle only; obtained at 2.0 mM)

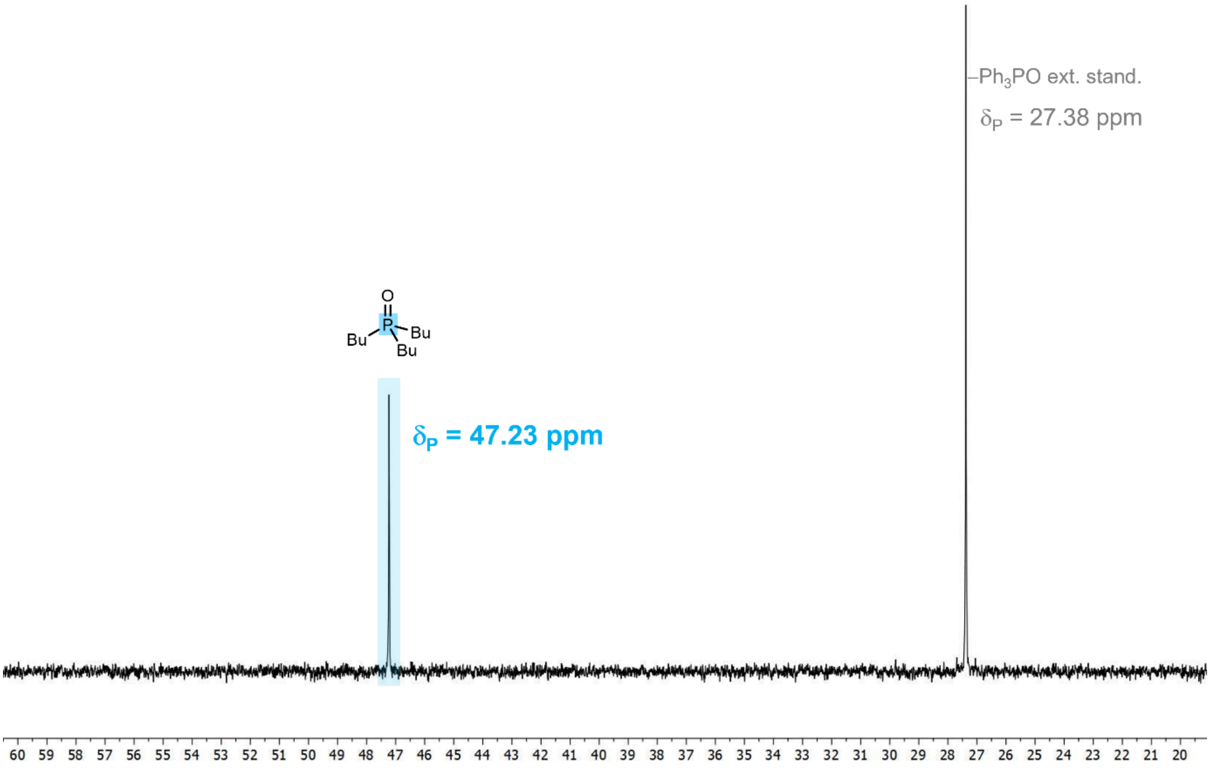

Figure S27 – $^{31}P\{^1H\}$ NMR spectrum of Bu_3PO (162 MHz, 2.0 mM, CD_2Cl_2).

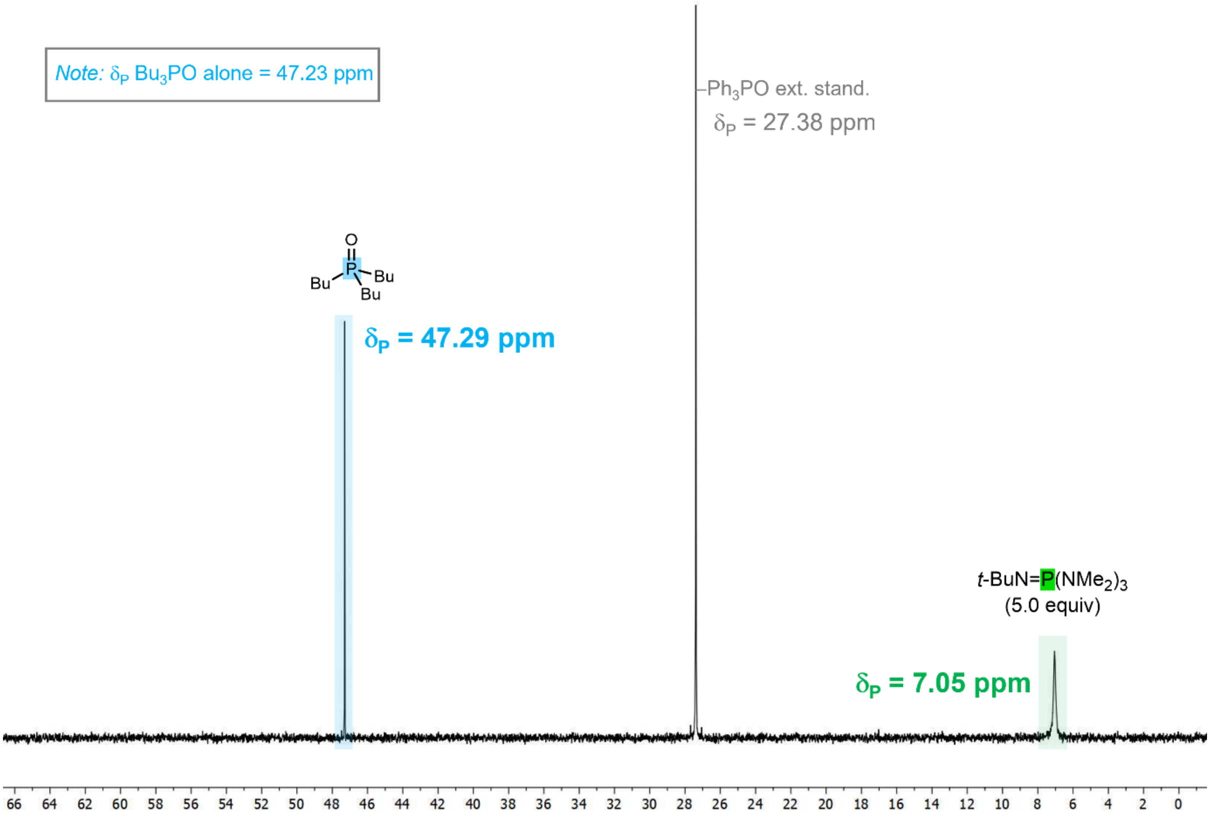

Figure S28 – $^{31}P\{^1H\}$ NMR spectrum of a mixture of Bu_3PO (1.9 mM) and *t*-BuN=P(NMe_2)$_3$ (5 equiv, 9.5 mM) (162 MHz, CD_2Cl_2).

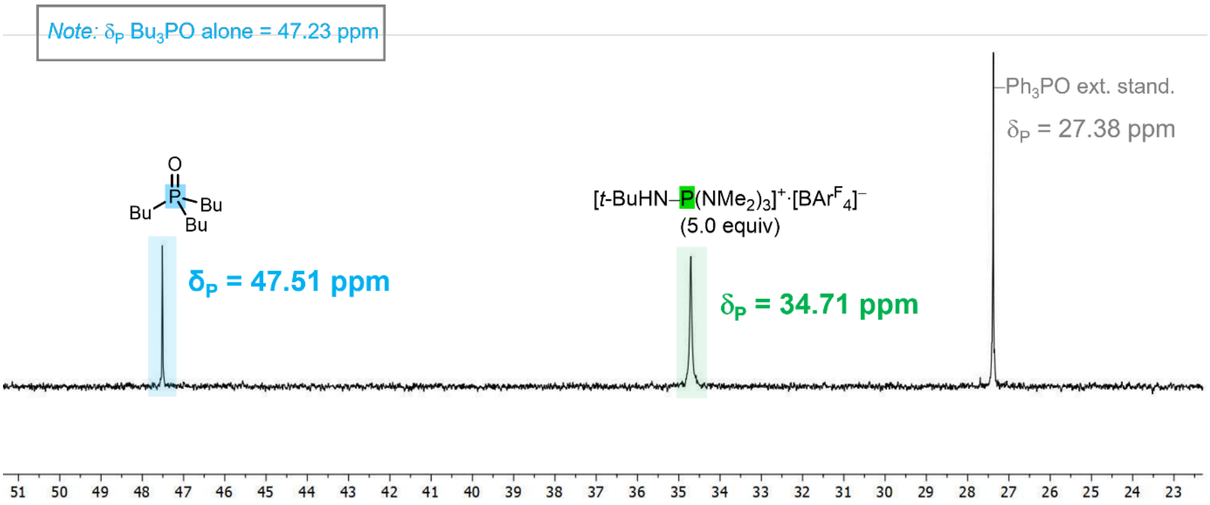

Figure S29 – $^{31}P\{^1H\}$ NMR spectrum of a mixture of Bu_3PO (2.0 mM) and $[t\text{-}BuHN{-}P(NMe_2)_3]^+{\cdot}[BAr^F{}_4]^-$ (5 equiv, 10 mM) (162 MHz, CD_2Cl_2). This experiment also provides further evidence that $[t\text{-}BuHN{-}P(NMe_2)_3]^+$ does not hydrogen bond to Bu_3PO to any significant extent – even when it is paired with the prototypical non-coordinating counterion $[BAr^F{}_4]^-$ – because the $\Delta\delta_P$ of Bu_3PO (relative to Bu_3PO alone) was minimal ($\Delta\delta_P$ $Bu_3PO = +0.28$ ppm).

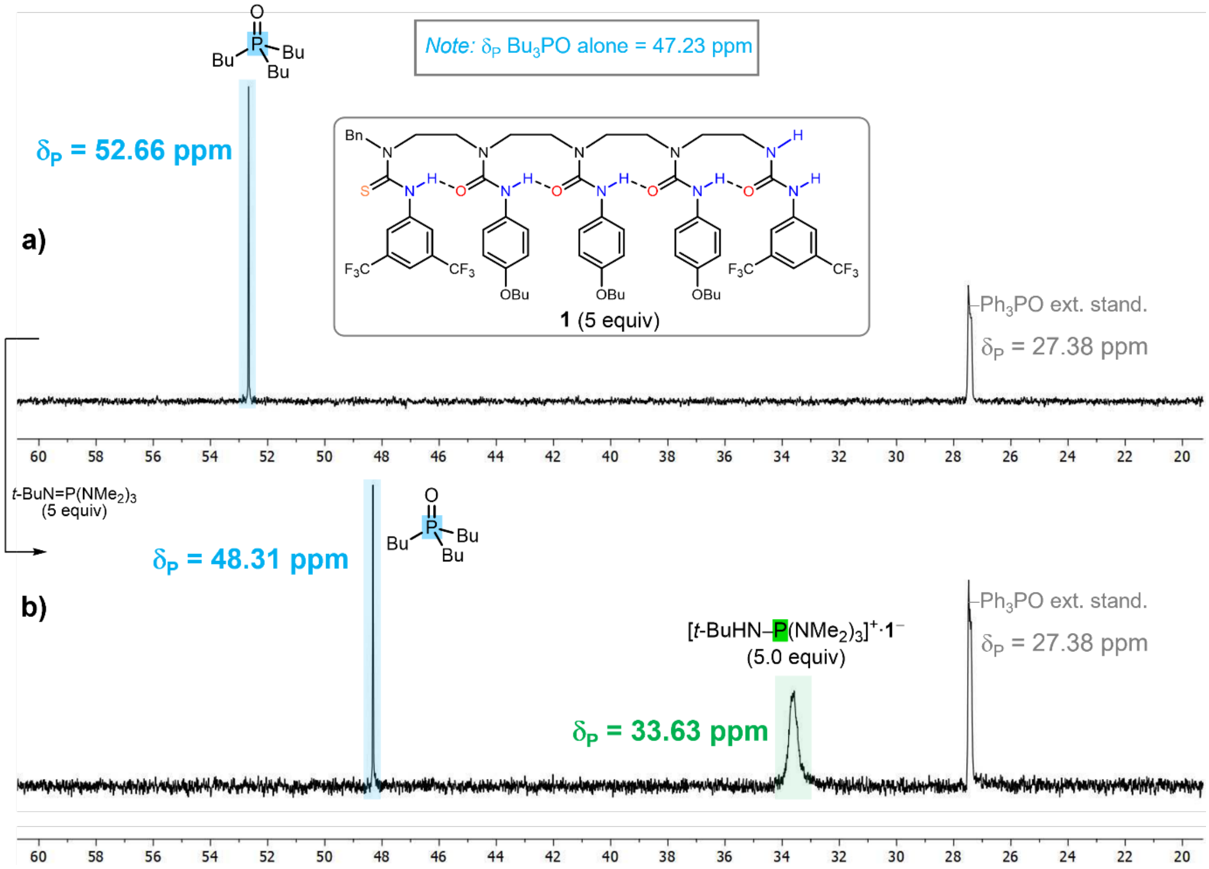

Figure S30 – (a) $^{31}P\{^1H\}$ NMR spectrum of a mixture of Bu_3PO (2.0 mM) and 1 (5 equiv, 10 mM) (162 MHz, CD_2Cl_2) and (b) after addition of t-BuN=P(NMe_2)$_3$ (5 equiv) to the same sample, generating a mixture of Bu_3PO (1.9 mM) and $[t\text{-}BuHN{-}P(NMe_2)_3]^+{\cdot}1^-$ (9.5 mM).

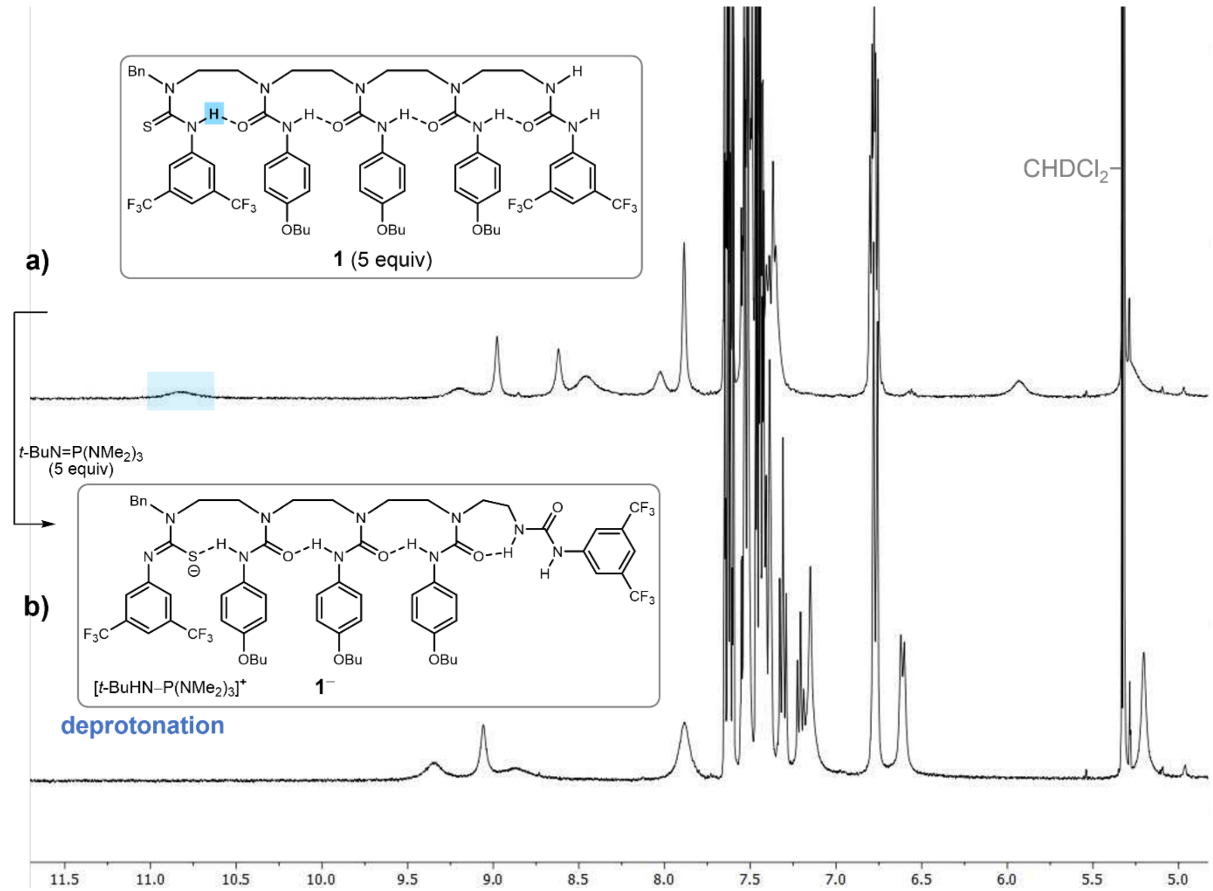

Figure S31 – (a) Accompanying ^{1}H NMR spectrum of a mixture of Bu_3PO (2.0 mM) and 1 (5 equiv, 10 mM) (400 MHz, CD_2Cl_2) and (b) after addition of *t*-BuN=P(NMe_2)$_3$ (5 equiv) to the same sample, generating a mixture of Bu_3PO (1.9 mM) and [*t*-BuHN−P(NMe_2)$_3$]$^+$·1$^-$ (9.5 mM). Deprotonation of **1** in (b) is indicated by the loss of the thiourea proton signal at δ_H = 10.82 ppm. (The spectra also contain signals in the aromatic region arising from the Ph_3PO external standard).

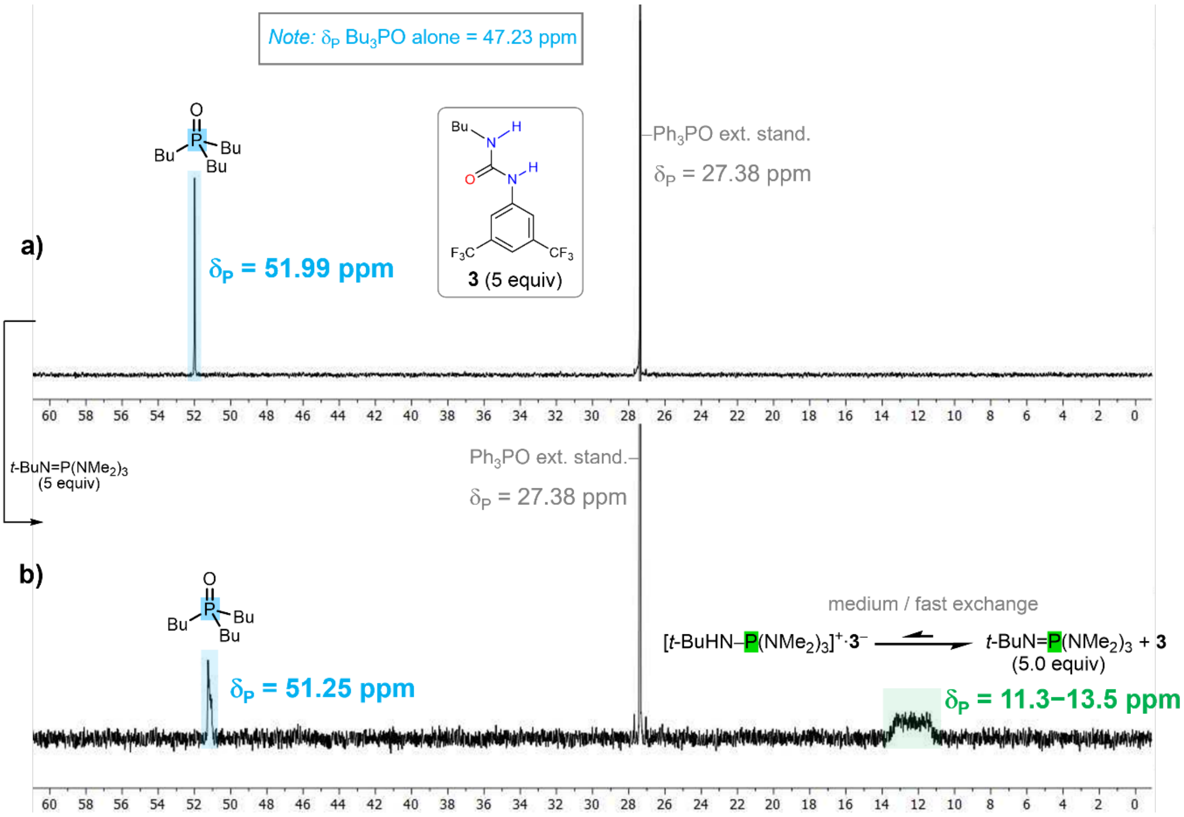

Figure S32 – (a) ^{31}P{^{1}H} NMR spectrum of a mixture of Bu_3PO (2.0 mM) and 3 (5 equiv, 10 mM) (162 MHz, CD_2Cl_2) and (b) after addition of *t*-BuN=P(NMe_2)$_3$ (5 equiv) to the same sample, generating a mixture of Bu_3PO (1.9 mM), 3 (9.5 mM) and *t*-BuN=P(NMe_2)$_3$ (9.5 mM). The broad signal at δ_P = 11.3−13.5 ppm is attributed to an equilibrium between *t*-BuN=P(NMe_2)$_3$ and [*t*-BuHN−P(NMe_2)$_3$]$^+$·**3**$^-$ arising from partial deprotonation of **3** (presumably at the aryl N–H). Presumably, this equilibrium strongly favours the freebase form because the observed δ_P is much closer to that of the freebase (*t*-BuN=P(NMe_2)$_3$ δ_P = 7.05 ppm) than the conjugate acid ([*t*-BuHN−P(NMe_2)$_3$]$^+$ = 32−35 ppm; counterion dependent).

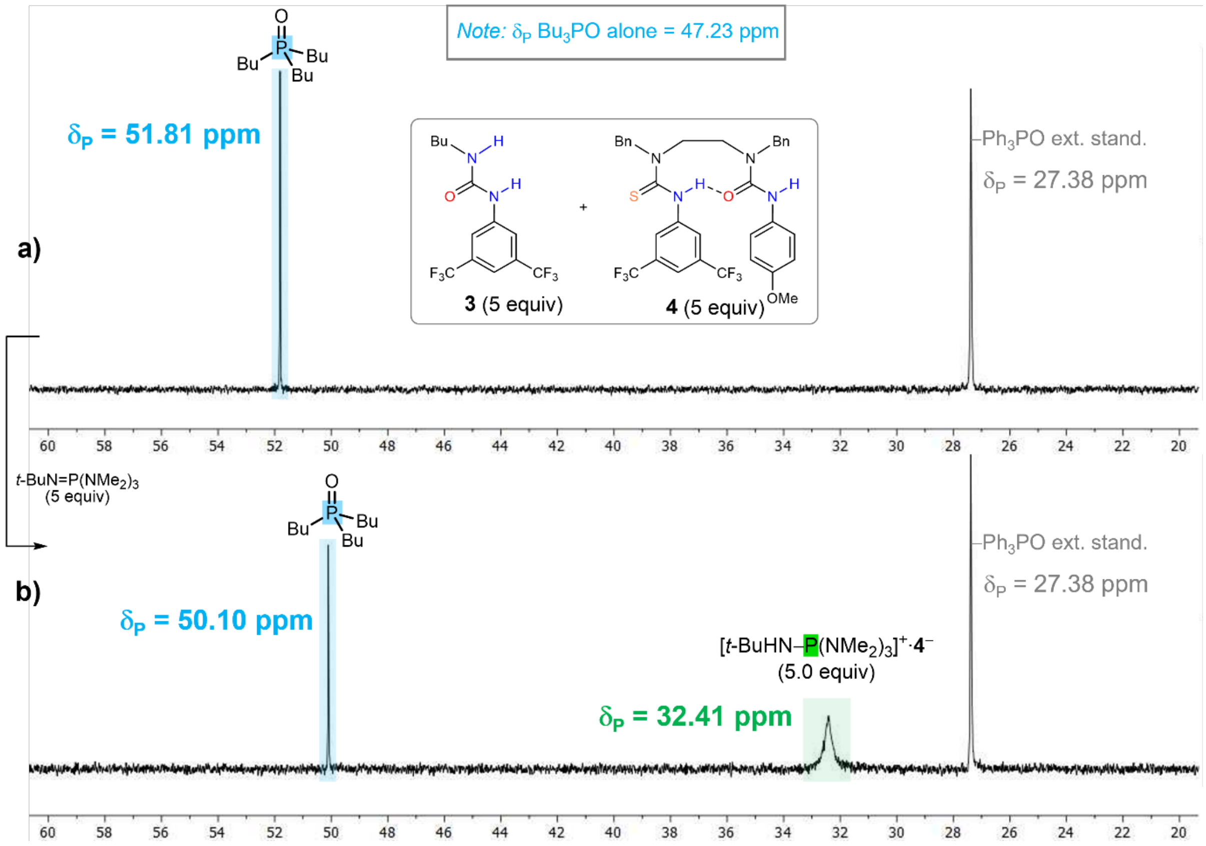

Figure S33 – (a) $^{31}P\{^1H\}$ NMR spectrum of a mixture of Bu_3PO (2.0 mM), 3 (5 equiv, 10 mM) and 4 (5 equiv, 10 mM) (162 MHz, CD_2Cl_2) and (b) after addition of *t*-BuN=P(NMe_2)$_3$ (5 equiv) to the same sample, generating a mixture of Bu_3PO (1.9 mM), 3 (9.5 mM) and [*t*-BuHN−P(NMe_2)$_3$]$^+$·4$^-$ (9.5 mM).

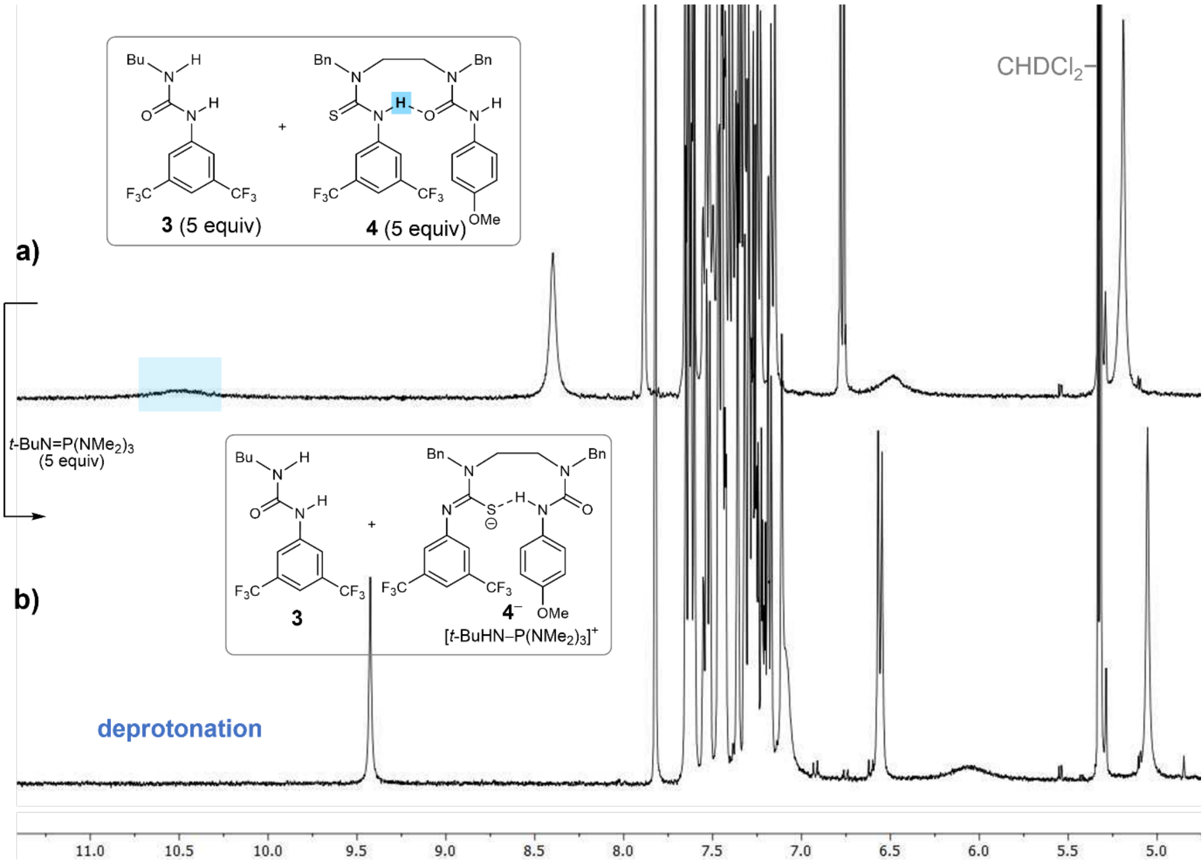

Figure S34 – (a) Accompanying ^{1}H NMR spectrum of a mixture of Bu_3PO (2.0 mM), 3 (5 equiv, 10 mM) and 4 (5 equiv, 10 mM) (400 MHz, CD_2Cl_2) and (b) after addition of *t*-BuN=P(NMe_2)$_3$ (5 equiv) to the same sample, generating a mixture of Bu_3PO (1.9 mM), 3 (9.5 mM) and [*t*-BuHN−P(NMe_2)$_3$]$^+$·4$^-$ (9.5 mM). Deprotonation of **4** in (b) is indicated by the loss of the thiourea proton signal at δ_H = 10.49 ppm. (The spectra also contain signals in the aromatic region arising from the Ph_3PO external standard.)

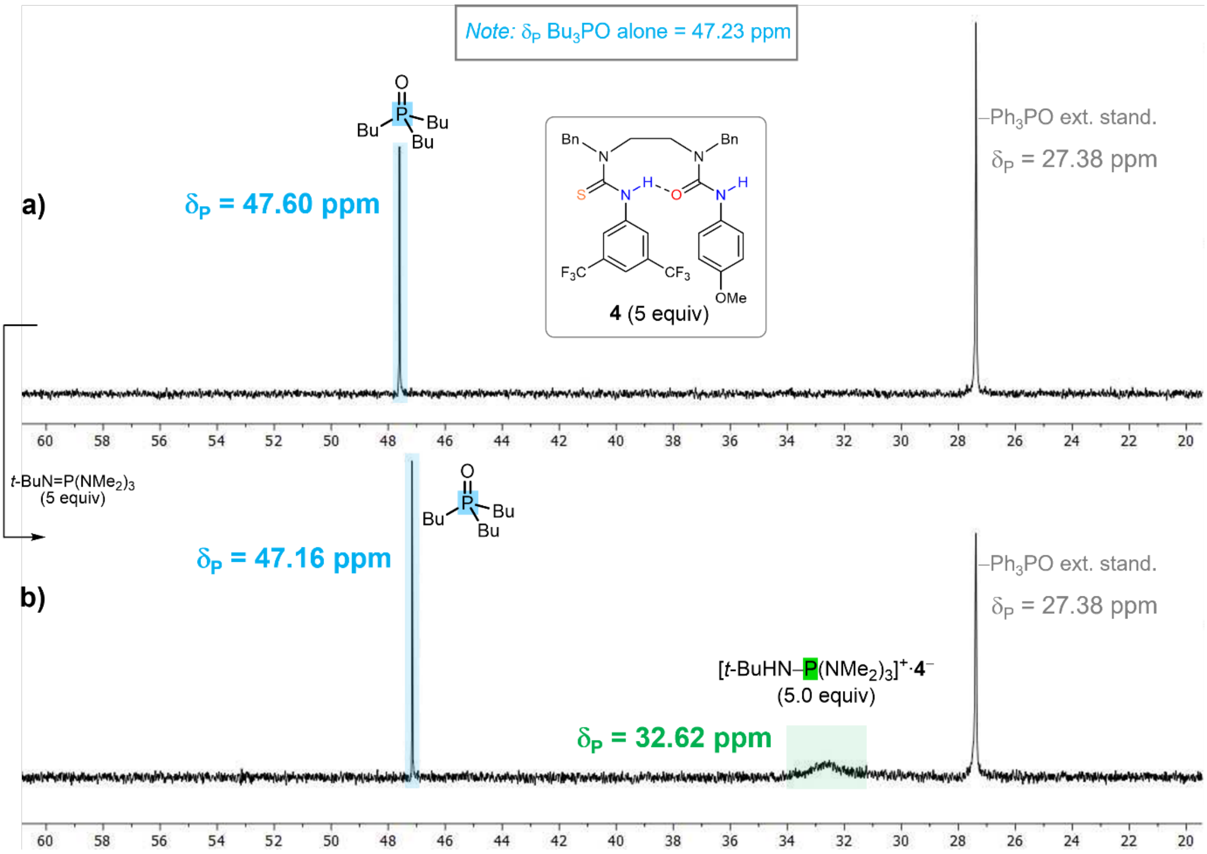

Figure S35 – (a) $^{31}P\{^{1}H\}$ NMR spectrum of a mixture of Bu_3PO (2.0 mM) and 4 (5 equiv, 10 mM) (162 MHz, CD_2Cl_2) and (b) after addition of *t*-BuN=P(NMe_2)$_3$ (5 equiv) to the same sample, generating a mixture of Bu_3PO (1.9 mM), and [*t*-BuHN–P(NMe_2)$_3$]$^{+}$·4^{-} (9.5 mM).

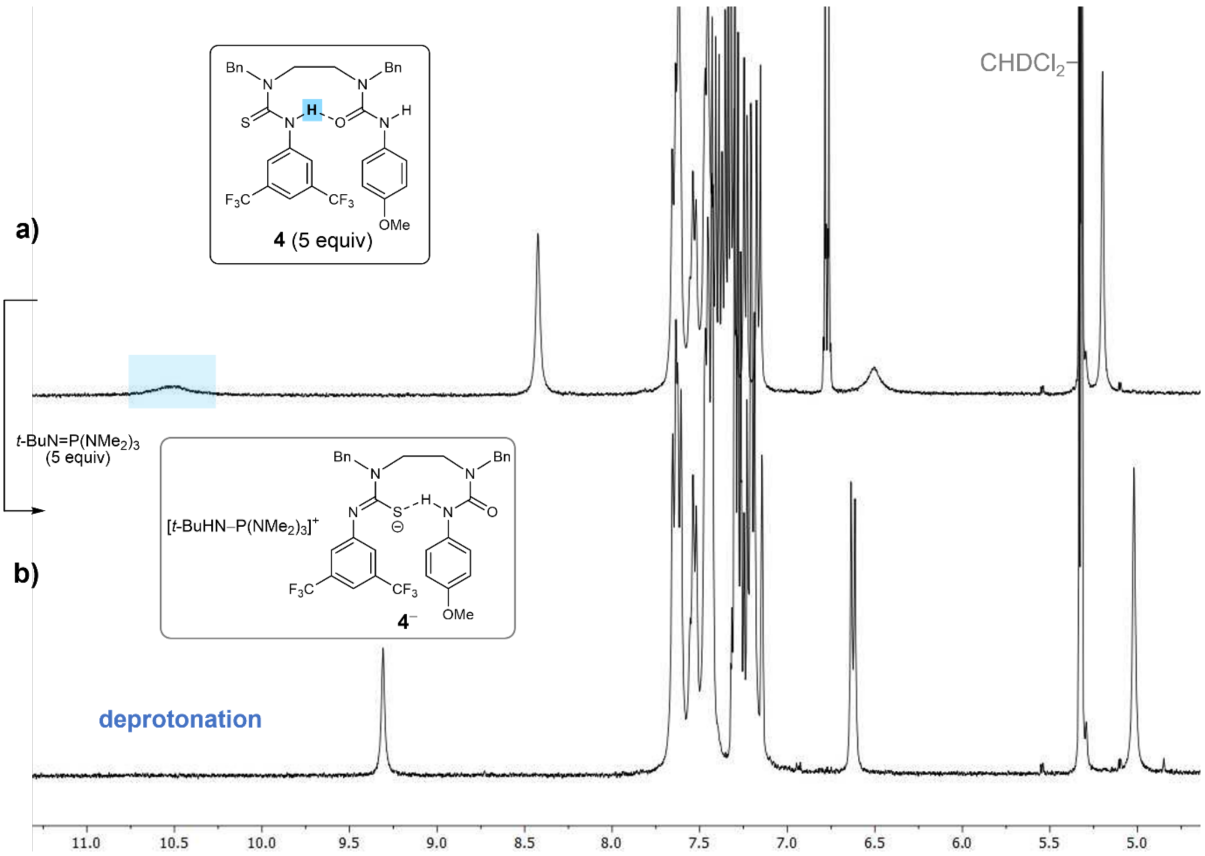

Figure S36 – (a) Accompanying ^{1}H NMR spectrum of a mixture of Bu_3PO (2.0 mM) and 4 (5 equiv, 10 mM) (400 MHz, CD_2Cl_2) and (b) after addition of *t*-BuN=P(NMe_2)$_3$ (5 equiv) to the same sample, generating a mixture of Bu_3PO (1.9 mM) and [*t*-BuHN−P(NMe_2)$_3$]$^{+}$·4^{-} (9.5 mM). Deprotonation of **4** in (b) is indicated by the loss of the thiourea proton signal at δ_H = 10.51 ppm. (The spectra also contain signals in the aromatic region arising from the Ph_3PO external standard).

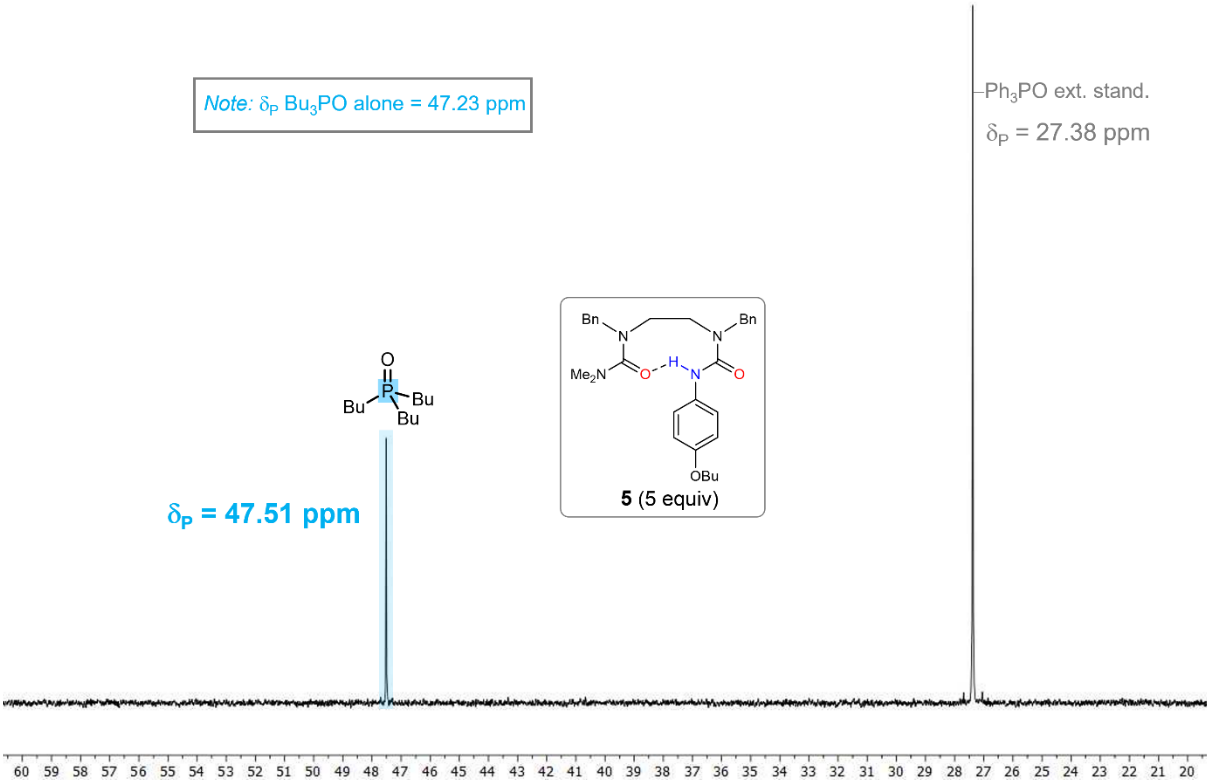

Figure S37 – $^{31}P\{^{1}H\}$ NMR spectrum of a mixture of Bu_3PO (2.0 mM) and 5 (5 equiv, 10 mM) (162 MHz, CD_2Cl_2).

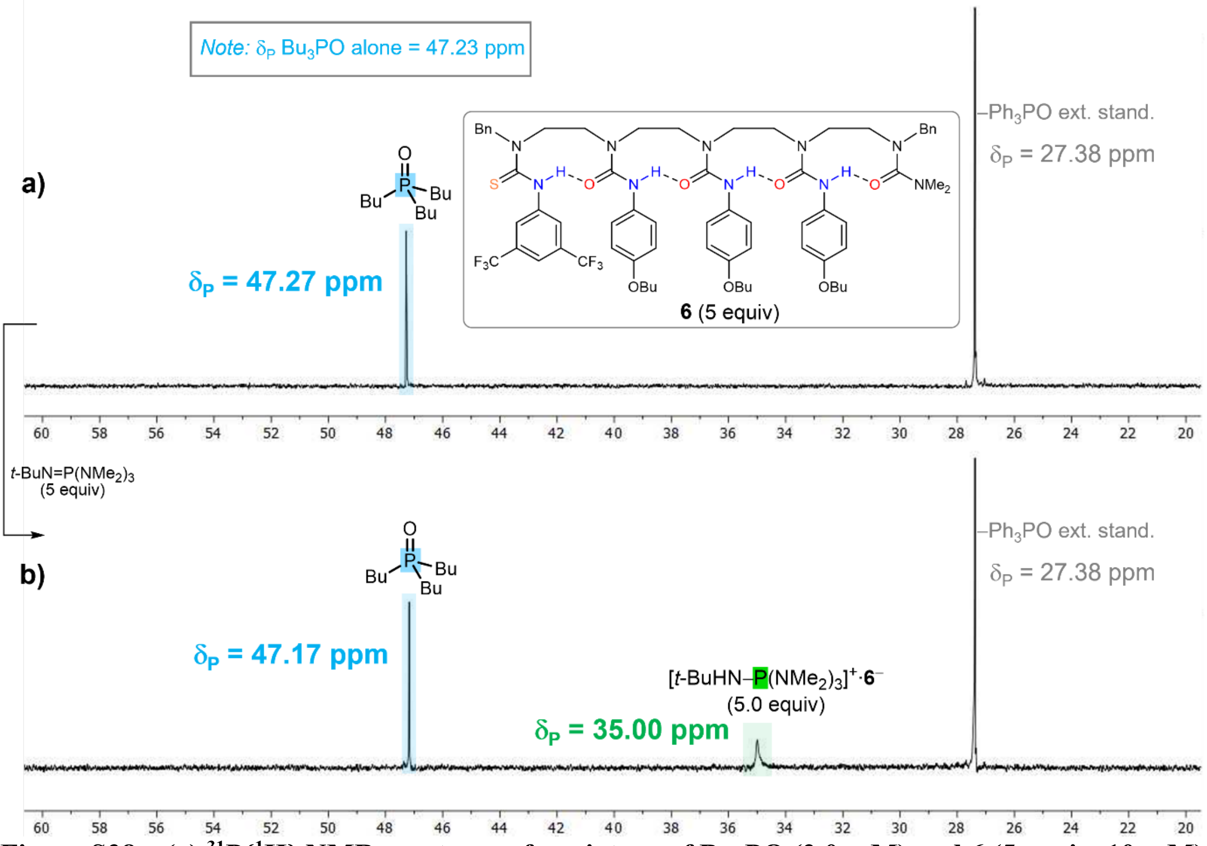

Figure S38 – (a) $^{31}P\{^{1}H\}$ NMR spectrum of a mixture of Bu_3PO (2.0 mM) and 6 (5 equiv, 10 mM) (162 MHz, CD_2Cl_2) and (b) after addition of *t*-BuN=P(NMe_2)$_3$ (5 equiv) to the same sample, generating a mixture of Bu_3PO (1.9 mM), and [*t*-BuHN−P(NMe_2)$_3$]$^+$·6$^-$ (9.5 mM).

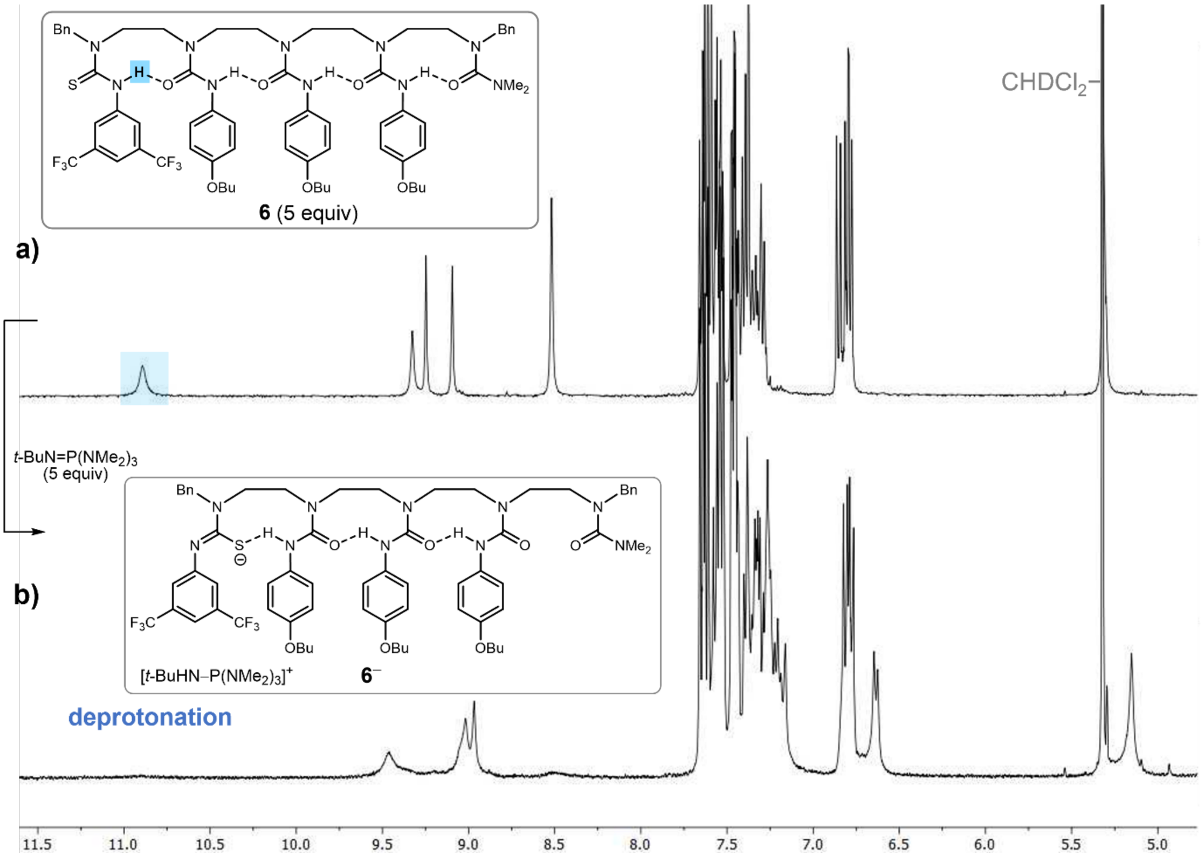

Figure S39 – (a) Accompanying ^{1}H NMR spectrum of a mixture of Bu_3PO (2.0 mM) and 6 (5 equiv, 10 mM) (400 MHz, CD_2Cl_2) and (b) after addition of *t*-BuN=P(NMe_2)$_3$ (5 equiv) to the same sample, generating a mixture of Bu_3PO (1.9 mM) and [*t*-BuHN−P(NMe_2)$_3$]$^{+}$·6^{-} (9.5 mM). Deprotonation of **6** in (b) is indicated by the loss of the thiourea proton signal at δ_H = 10.89 ppm. (The spectra also contain signals in the aromatic region arising from the Ph_3PO external standard.)

Additional Points for ^{31}P NMR Capture-and-Release Cycles with Compound 7

• The ^{31}P NMR spectrum of 'free' Bu_3PO with the Ph_3PO external standard was previously recorded as described in the *general procedure for ^{31}P NMR capture-and-release experiments and controls:* δ_P Bu_3PO = 47.23 ppm; δ_P Ph_3PO = 27.38 ppm; all spectra were referenced to the external standard at 27.38 ppm.

• To ensure the volume in the NMR tube was maintained at 0.50 mL throughout these experiments (and hence a constant 2.0 mM concentration of Bu_3PO), a 'reference NMR sample' was prepared containing 0.50 mL of CH_2Cl_2 and an empty capillary tube (the same size as used for the external standard); a line was marked on this tube at the solvent level as a reference.

• The total time between each deprotonation / re-protonation cycle − from adding *t*-BuN=P(NMe_2)$_3$ to form **7**$^{-}$, recording the ^{1}H and ^{31}P NMR spectra, and re-protonating with [4-Cl-pyH]$^{+}$·[BAr$^{F}_{4}$]$^{-}$ − was no

longer than 30−45 minutes each time; this prevents alkylation of **7**$^{-}$ by CD_2Cl_2 as discussed previously. After re-protonation to re-form **7**, the time before the next addition of *t*-BuN=P(NMe_2)$_3$ (usually 0.5−2 h) is less important, because **7** itself, Bu_3PO and the by-products [*t*-BuHN−P(NMe_2)$_3$]$^{+}$·[$BAr^F{}_4$]$^{-}$ and 4-Cl-py appear to be stable under these conditions, at least over the time course of these experiments (~8−12 h).

Procedure for ^{31}P NMR capture-and-release cycles with compound 7: A 1.7 mL vial was charged with **7** (9.24 mg, 5.0 μmol, 5.0 equiv) and a solution of Bu_3PO (2.0 mM in CD_2Cl_2, 0.50 mL, 1.0 μmol, 1.0 equiv) was added. The resulting solution was transferred via pipette to an NMR tube. Additional CD_2Cl_2 (~0.15 mL) was added to the vial and the solution was again transferred to the NMR tube to ensure quantitative transfer of **7** and Bu_3PO. A sealed capillary tube containing Ph_3PO (150 mM in CD_2Cl_2) was added to the NMR tube as an external standard. The volume in the NMR tube was then re-adjusted to the reference 0.50 mL mark (see second point above) by blowing N_2 into the tube using a 120 mm length, 21-gauge needle attached to a N_2-filled balloon. During this process, the moisture building up on the exterior of the NMR tube (due to endothermic evaporation) was removed with paper towel. ^{1}H and ^{31}P{^{1}H} NMR spectra were acquired. Next, under air, a solution of *t*-BuN=P(NMe_2)$_3$ (50 mM in CD_2Cl_2, 100 μL, 5.0 μmol, 5.0 equiv) was added via a Gilson pipette and additional CD_2Cl_2 (~0.05 mL) was added to rinse the upper interior of the NMR tube. The volume in the NMR tube was re-adjusted to the reference 0.50 mL mark as described. ^{1}H and ^{31}P{^{1}H} NMR spectra were then acquired. A solution of [4-Cl-pyH]$^{+}$·[$BAr^F{}_4$]$^{-}$ (4.89 mg, 5.0 μmol, 5.0 equiv) in CD_2Cl_2 (~0.5 mL) was then added from a 1.7 mL vial. Further CD_2Cl_2 (~0.15 mL) was added to the vial and this solution was again added to the NMR tube to ensure complete transfer of [4-Cl-pyH]$^{+}$·[$BAr^F{}_4$]$^{-}$. A small amount of additional CD_2Cl_2 (~0.05 mL) was then used to rinse the upper interior of the NMR tube. The volume in the NMR tube was again re-adjusted to the reference 0.50 mL mark as described, and the ^{1}H and ^{31}P{^{1}H} NMR spectra were acquired. The process of sequentially adding *t*-BuN=P(NMe_2)$_3$ and [4-Cl-pyH]$^{+}$·[$BAr^F{}_4$]$^{-}$ (and each time recording the ^{1}H and ^{31}P{^{1}H} NMR spectra) was repeated three more times as described.

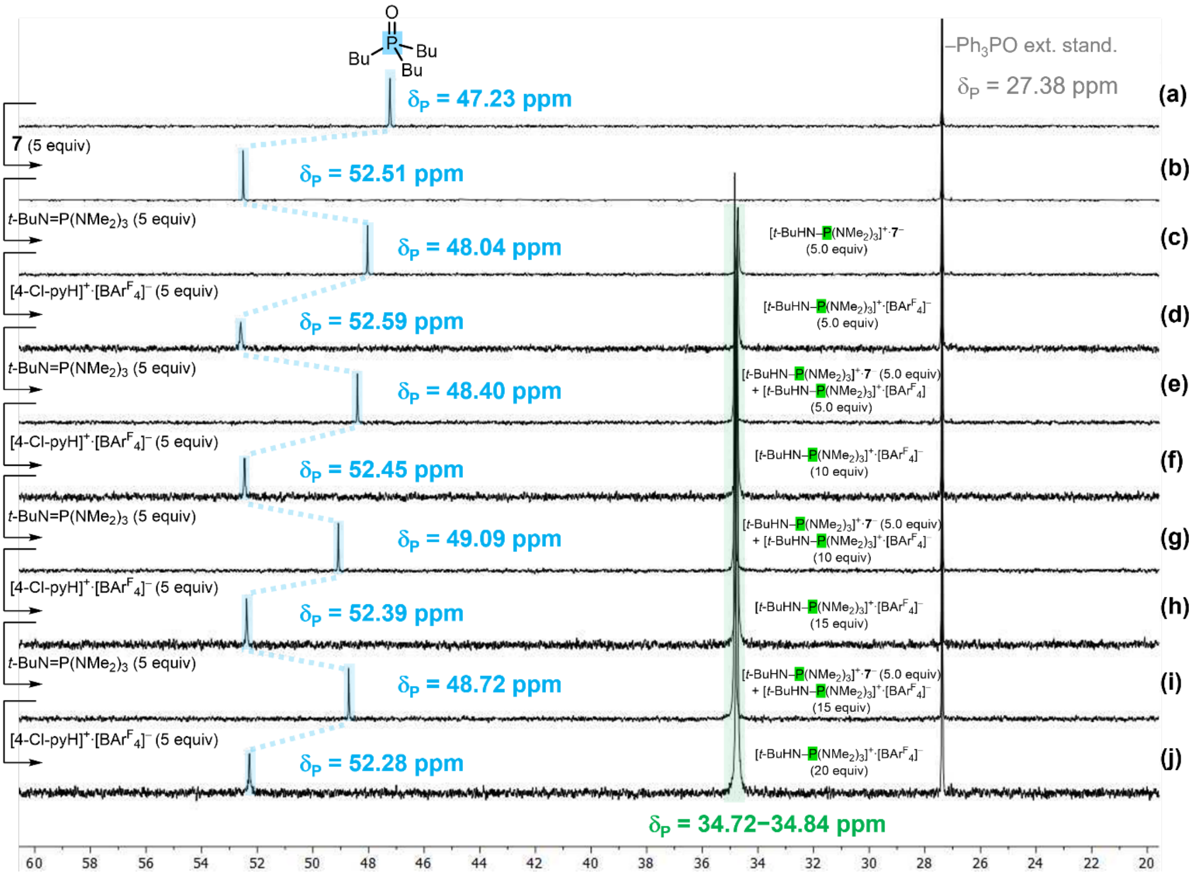

Figure S40 – (a) $^{31}P\{^1H\}$ NMR spectrum of 'free' Bu_3PO (162 MHz, 2.0 mM, CD_2Cl_2). (b) $^{31}P\{^1H\}$ NMR spectrum of a mixture of Bu_3PO (2.0 mM) and 7 (5 equiv, 10 mM) (162 MHz, CD_2Cl_2) and (c) after addition of *t*-BuN=P(NMe_2)$_3$ (5 equiv) to the same sample, generating a mixture of Bu_3PO (2.0 mM) and [*t*-BuHN−P(NMe_2)$_3$]$^+$·7$^-$ (10 mM) and (d) after addition of [4-Cl-pyH]$^+$·[BAr$^F{}_4$]$^-$ (5 equiv) to the same sample, generating a mixture of Bu_3PO (2.0 mM), 7 (10 mM), [*t*-BuHN−P(NMe_2)$_3$]$^+$·[BAr$^F{}_4$]$^-$ (10 mM) and 4-Cl-py (10 mM). (e)−(j) $^{31}P\{^1H\}$ NMR spectra from further cycles of deprotonation (of 7) with *t*-BuN=P(NMe_2)$_3$ and re-protonation (of 7$^-$) with [4-Cl-pyH]$^+$·[BAr$^F{}_4$]$^-$. Where both [*t*-BuHN−P(NMe_2)$_3$]$^+$·7$^-$ and [*t*-BuHN−P(NMe_2)$_3$]$^+$·[BAr$^F{}_4$]$^-$ are present in (e),(g),(i), only a single signal is observed for [*t*-BuHN−P(NMe_2)$_3$]$^+$. Note that this Figure is an expansion of Figure 5 in the manuscript.

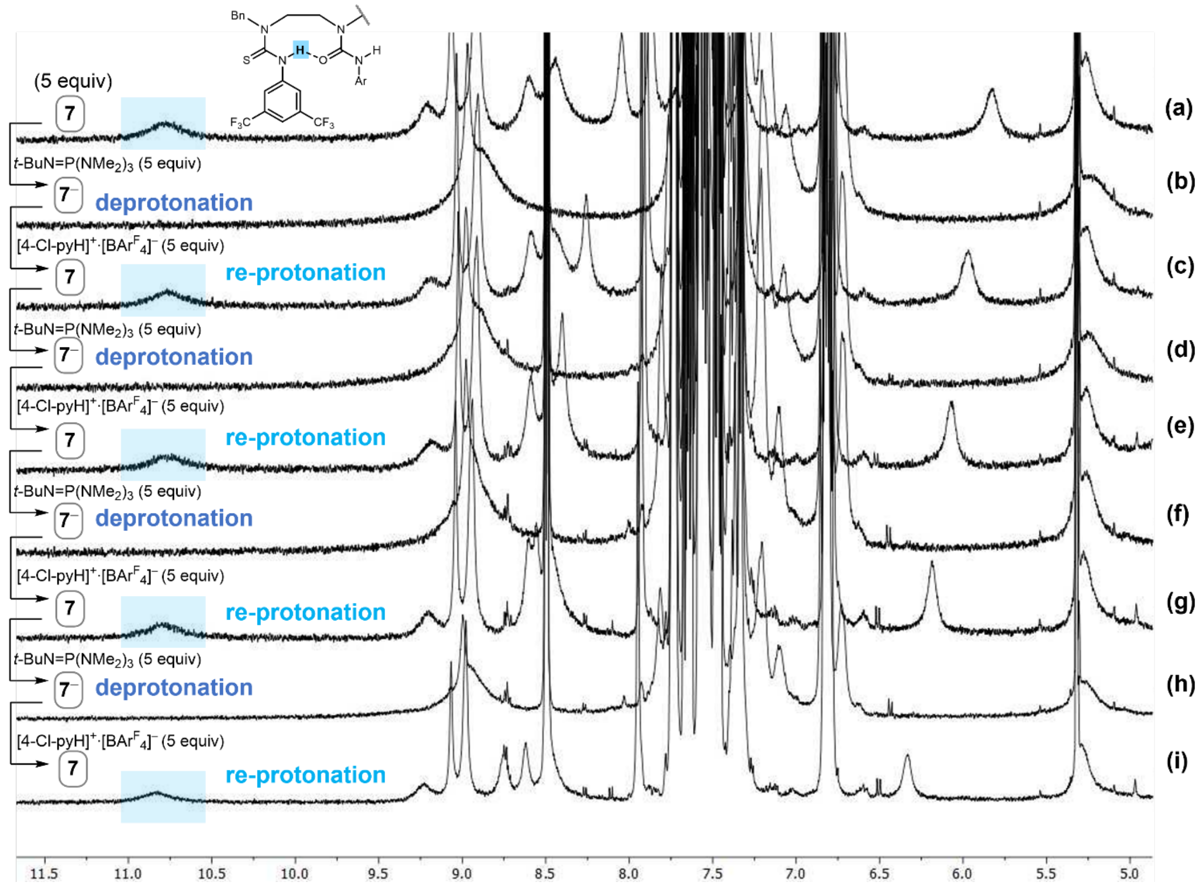

Figure S41 – (a) Accompanying ^{1}H NMR spectrum of a mixture of Bu$_3$PO (2.0 mM) and 7 (5 equiv, 10 mM) (400 MHz, CD$_2$Cl$_2$) and (b) after addition of *t*-BuN=P(NMe$_2$)$_3$ (5 equiv) to the same sample, generating a mixture of Bu$_3$PO (2.0 mM) and [*t*-BuHN−P(NMe$_2$)$_3$]$^+$·7$^−$ (10 mM) and (c) after addition of [4-Cl-pyH]$^+$·[BAr$^F{}_4$]$^−$ (5 equiv) to the same sample, generating a mixture of Bu$_3$PO (2.0 mM), 7 (10 mM), [*t*-BuHN−P(NMe$_2$)$_3$]$^+$·[BAr$^F{}_4$]$^−$ (10 mM) and 4-Cl-py (10 mM). (d)−(i) ^{1}H NMR spectra from further cycles of deprotonation (of 7) with *t*-BuN=P(NMe$_2$)$_3$ and re-protonation (of 7$^−$) with [4-Cl-pyH]$^+$·[BAr$^F{}_4$]$^−$. The formation of 7$^−$ in (b),(d),(f),(h) is indicated by the loss of the thiourea proton signal at δ_H = 10.77 ppm. The formation of **7** in (c),(e),(g),(i) is indicated by the reappearance of the thiourea proton signal. (All spectra also contain signals in the aromatic region arising from the Ph$_3$PO external standard.)

NMR Spectra of Novel Compounds

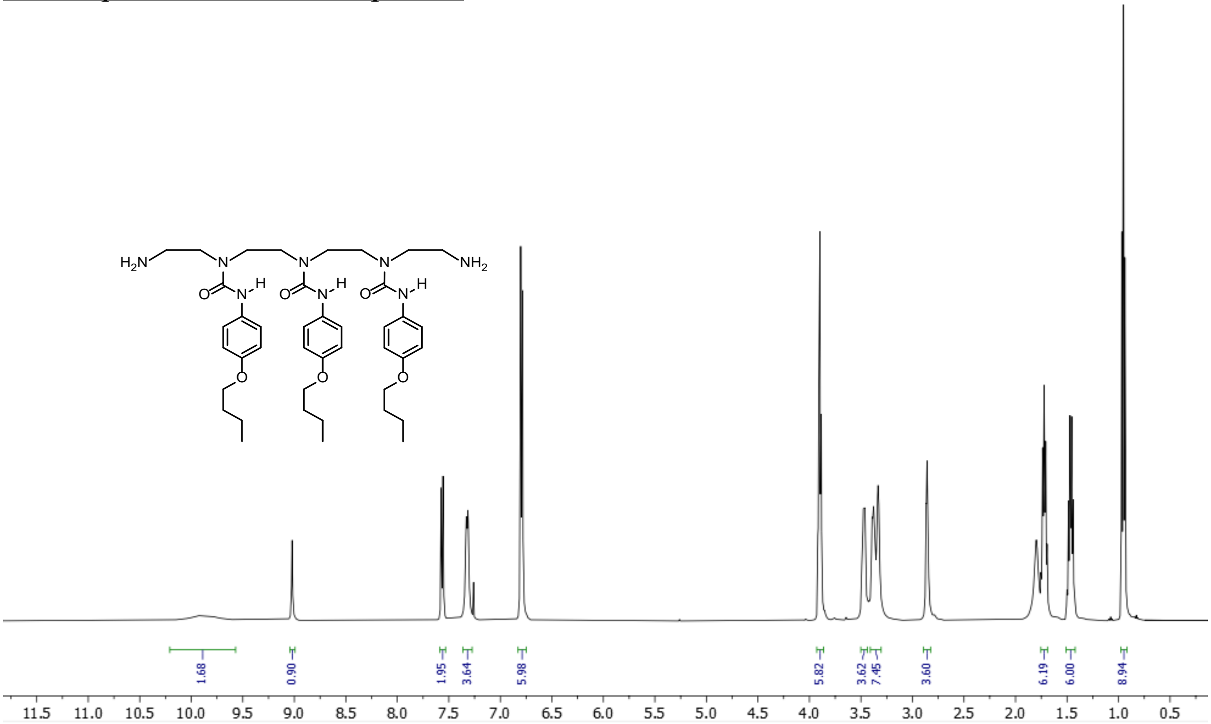

Figure S42 – ^{1}H NMR spectrum of 1-1 (500 MHz, $CDCl_3$).

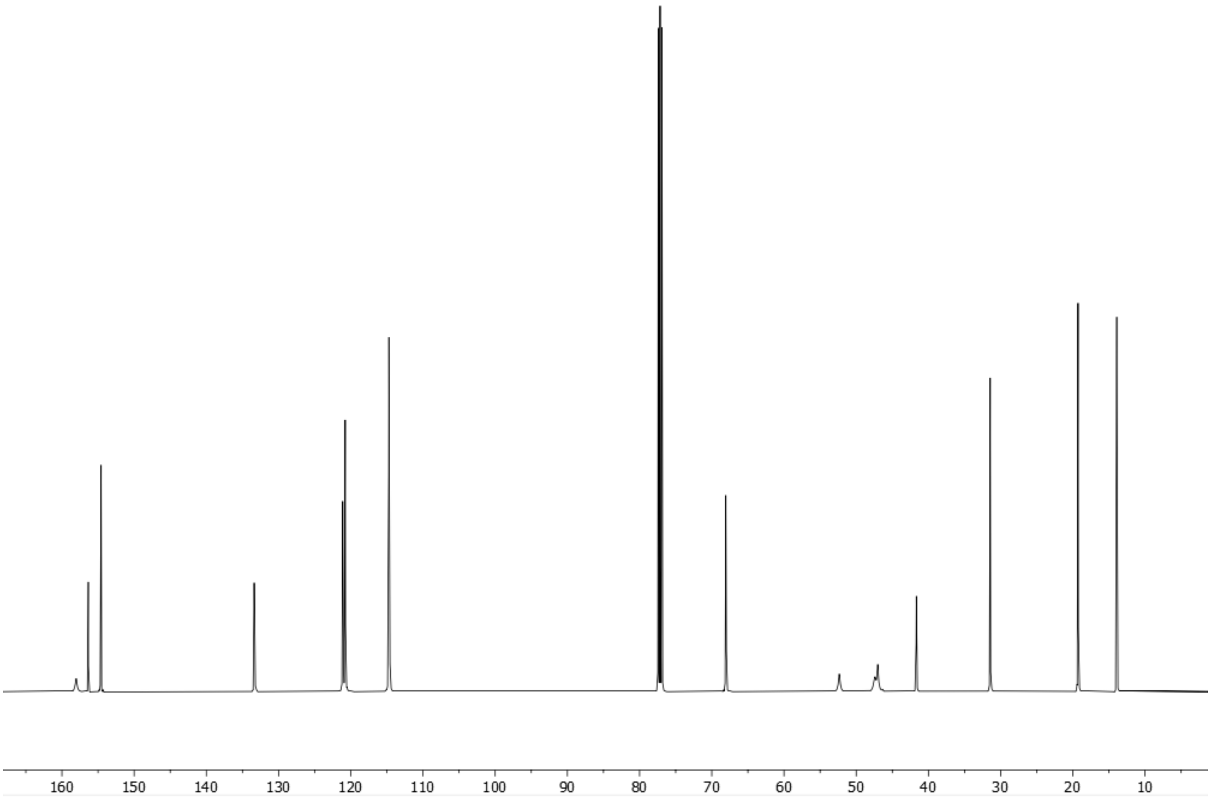

Figure S43 – ^{13}C NMR spectrum of 1-1 (126 MHz, $CDCl_3$).

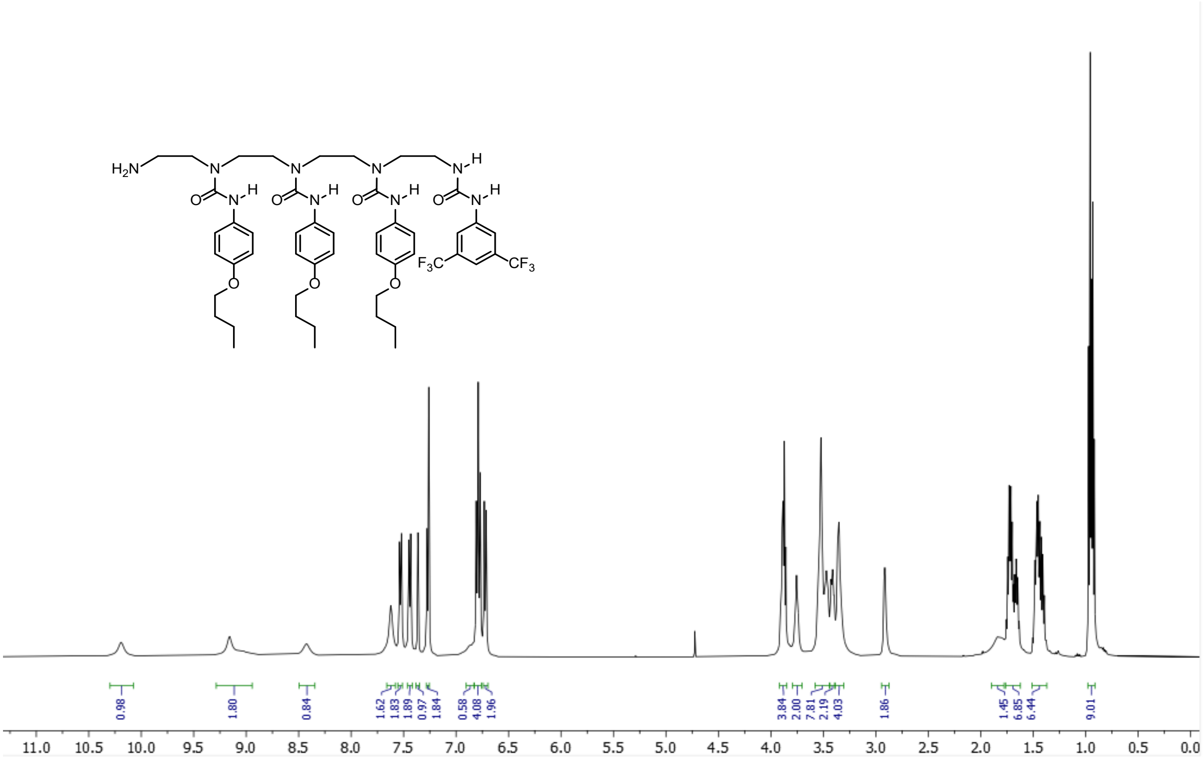

Figure S44 – ^{1}H NMR spectrum of 1-2 (500 MHz, $CDCl_3$).

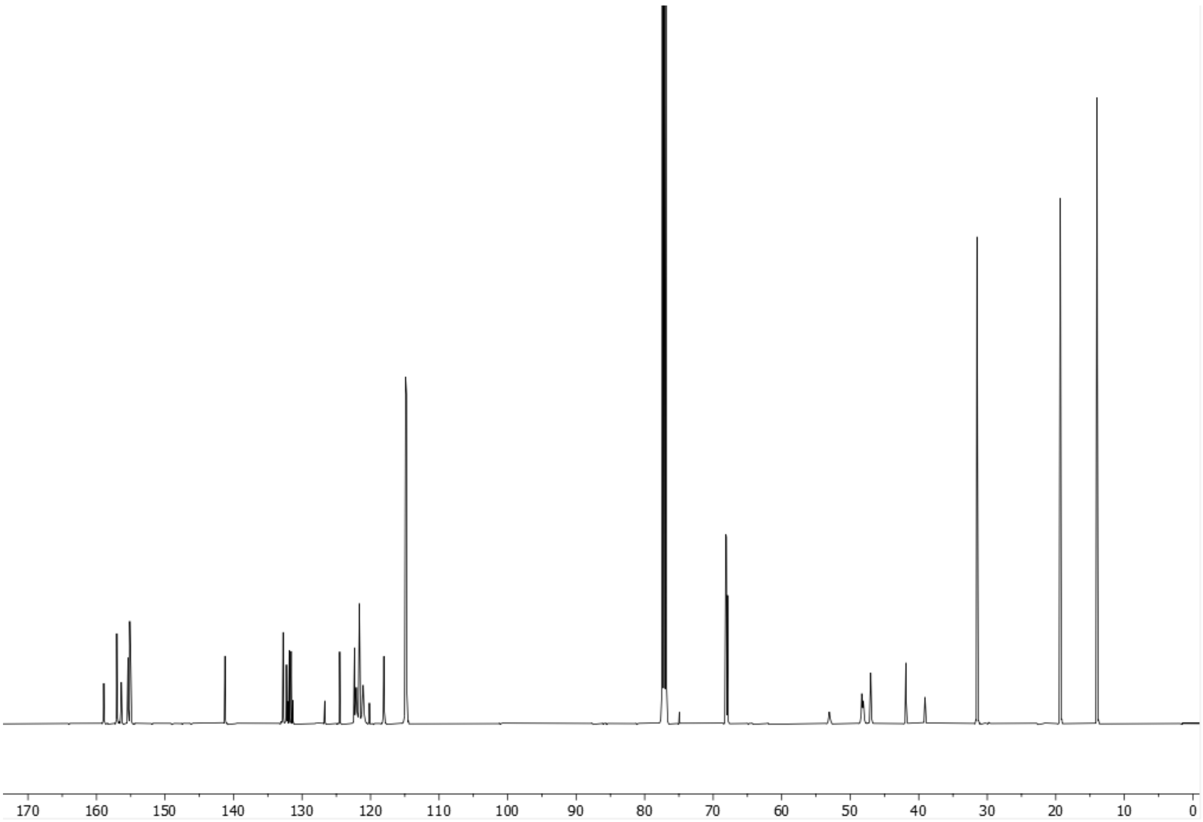

Figure S45 – ^{13}C NMR spectrum of 1-2 (126 MHz, $CDCl_3$).

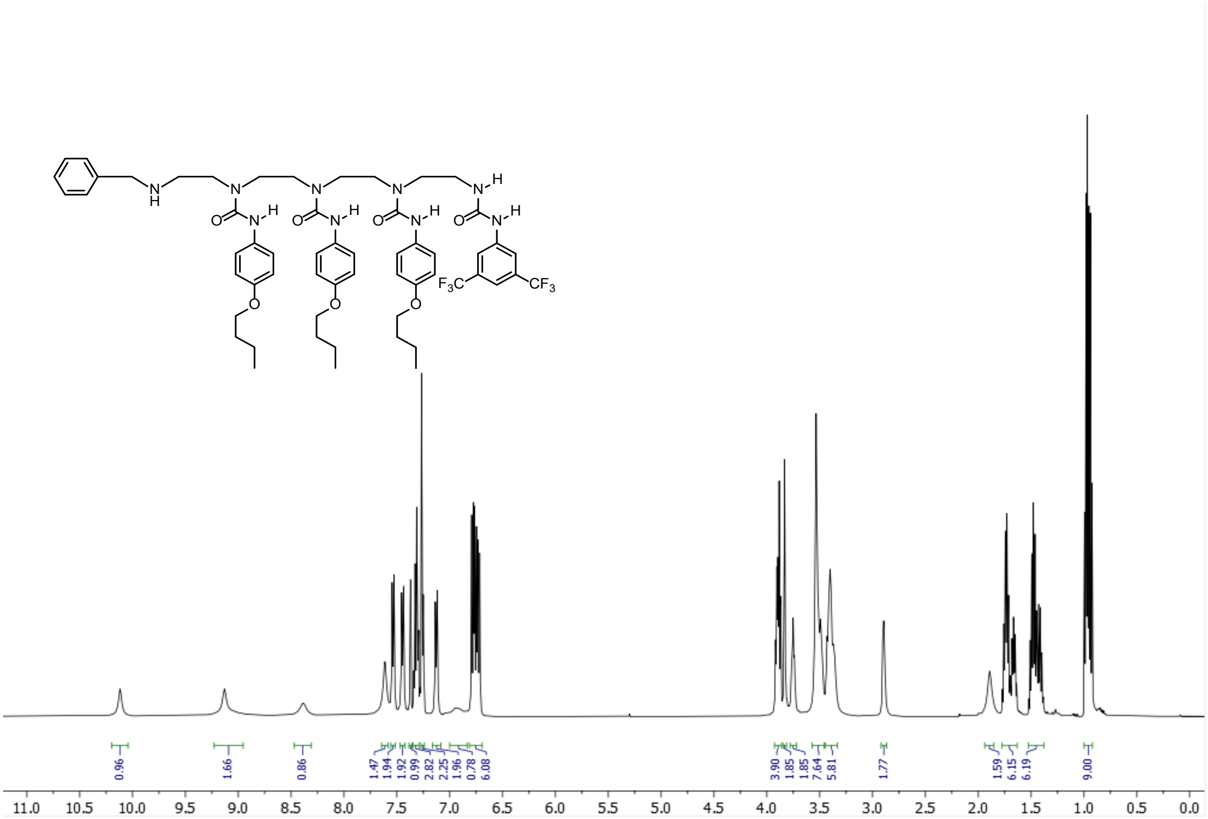

Figure S46 – ^{1}H NMR spectrum of 1-3 (500 MHz, $CDCl_3$).

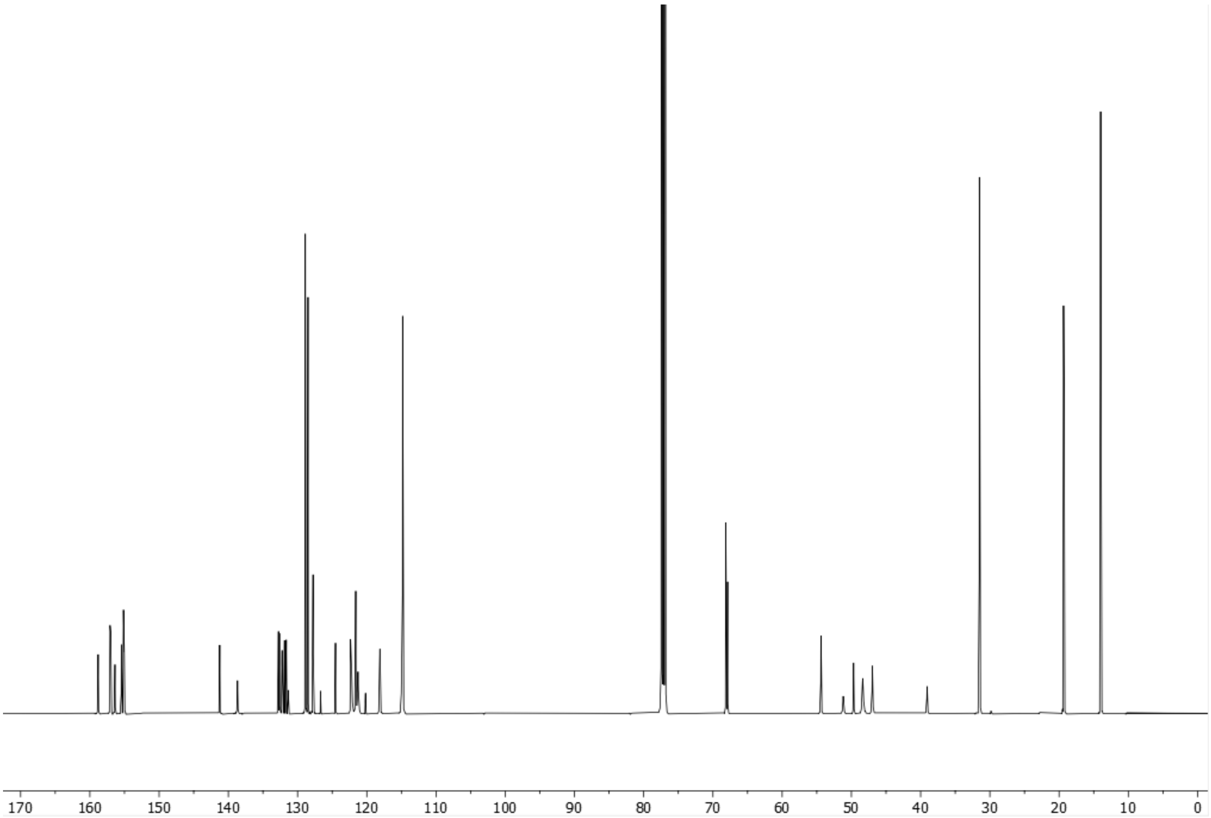

Figure S47 – ^{13}C NMR spectrum of 1-3 (126 MHz, $CDCl_3$).

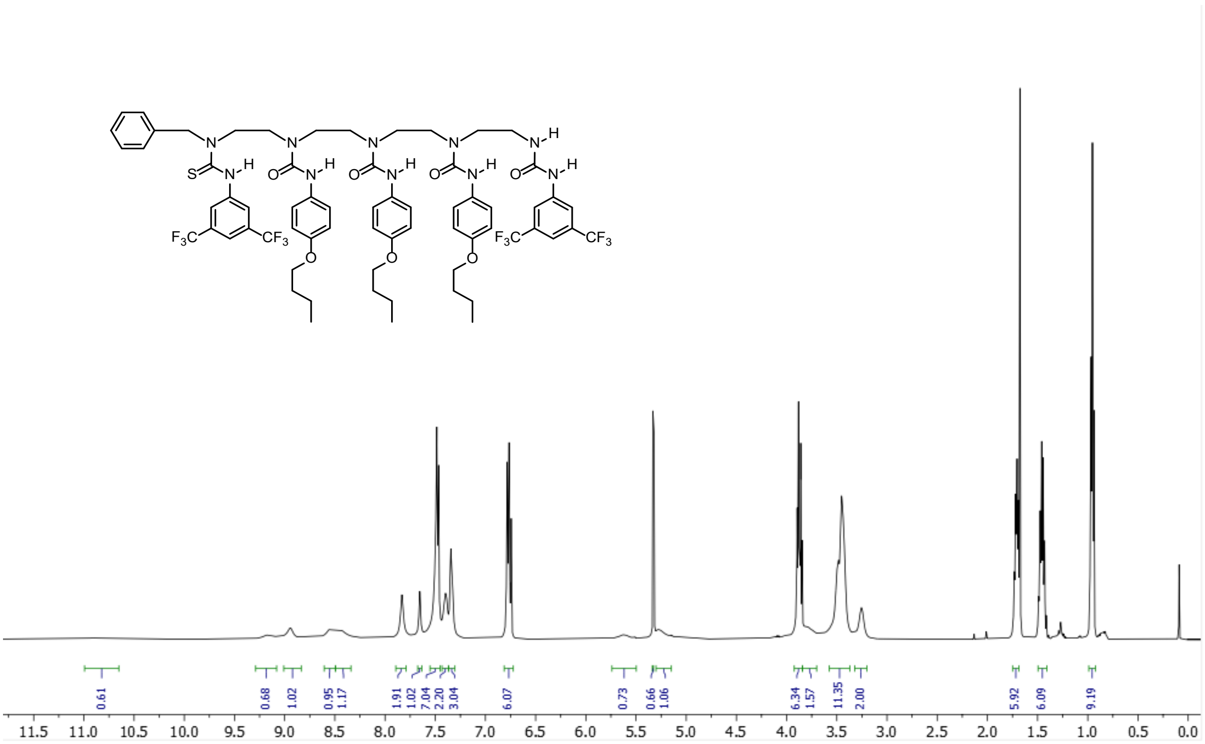

Figure S48 – ^{1}H NMR spectrum of 1 (500 MHz, CD_2Cl_2).

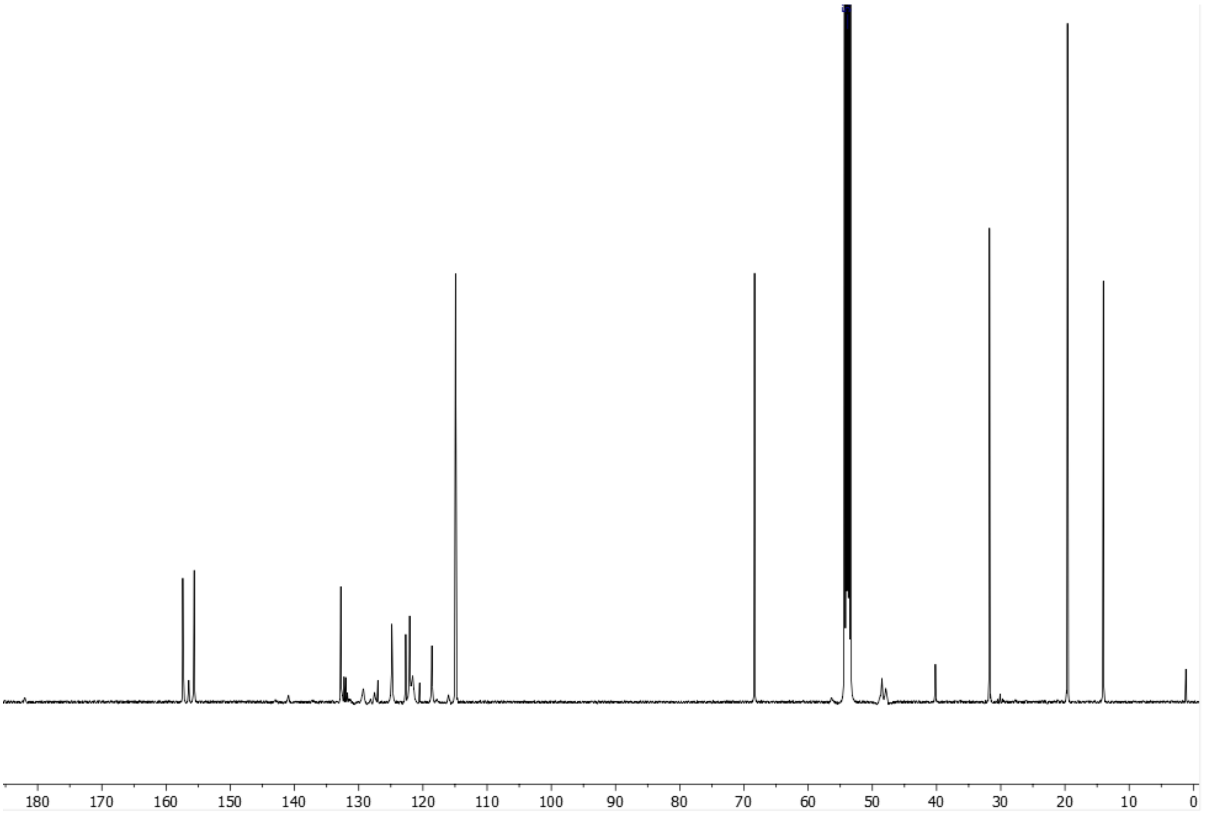

Figure S49 – ^{13}C NMR spectrum of 1 (126 MHz, CD_2Cl_2).

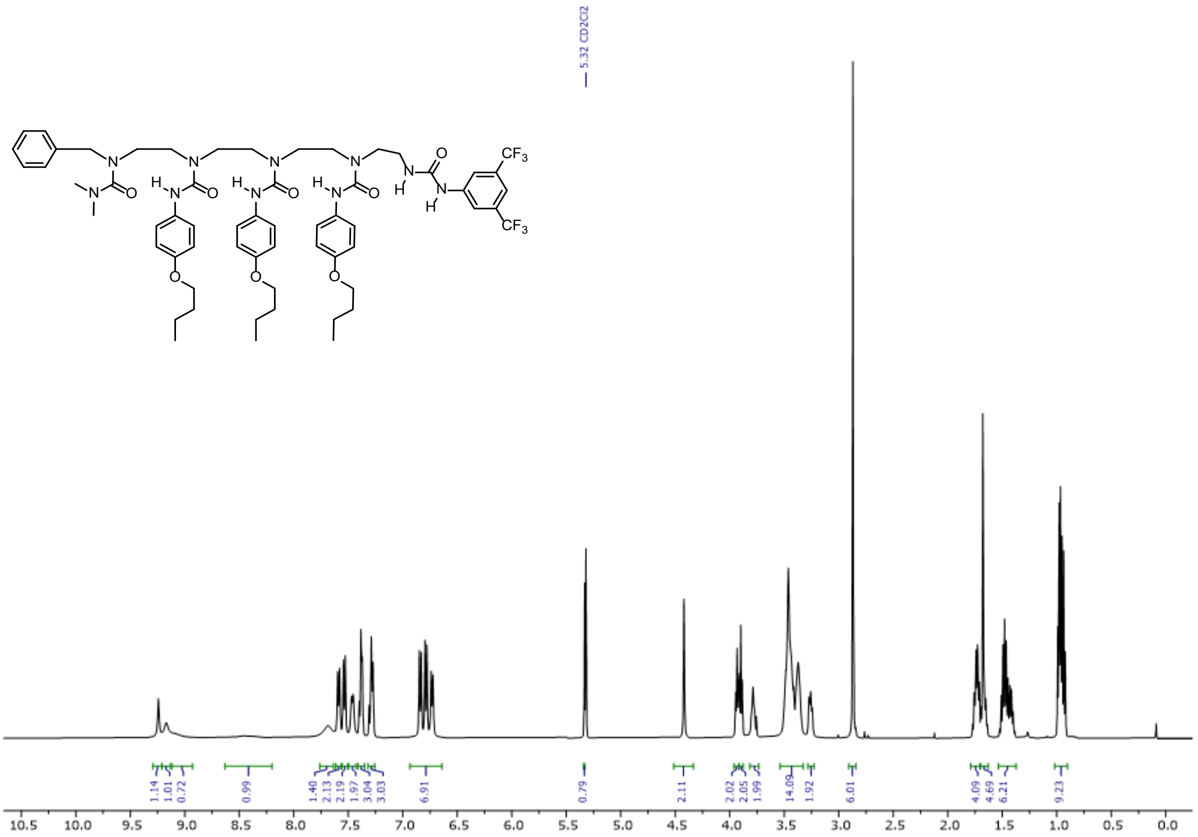

Figure S50 – ^{1}H NMR spectrum of 2 (500 MHz, CD_2Cl_2).

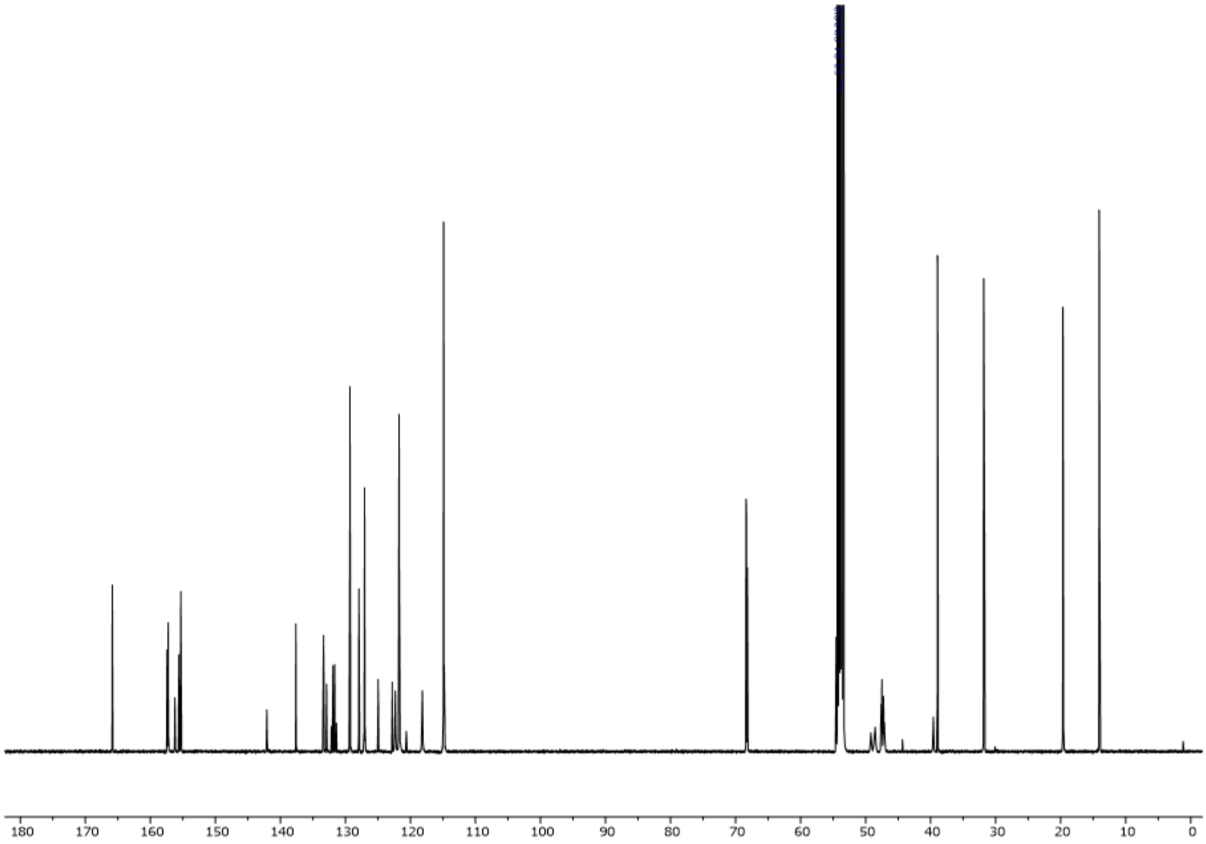

Figure S51 – ^{13}C NMR spectrum of 2 (126 MHz, CD_2Cl_2).

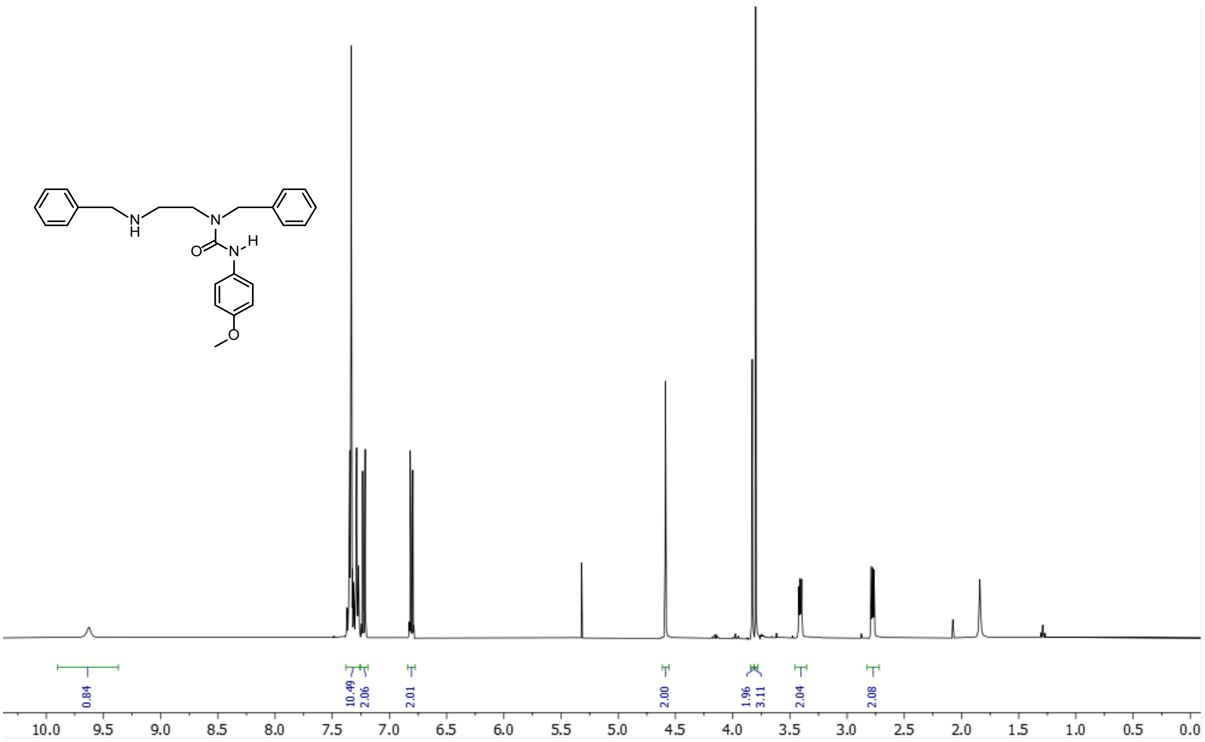

Figure S52 – ^{1}H NMR spectrum of 4-1 (400 MHz, $CDCl_3$).

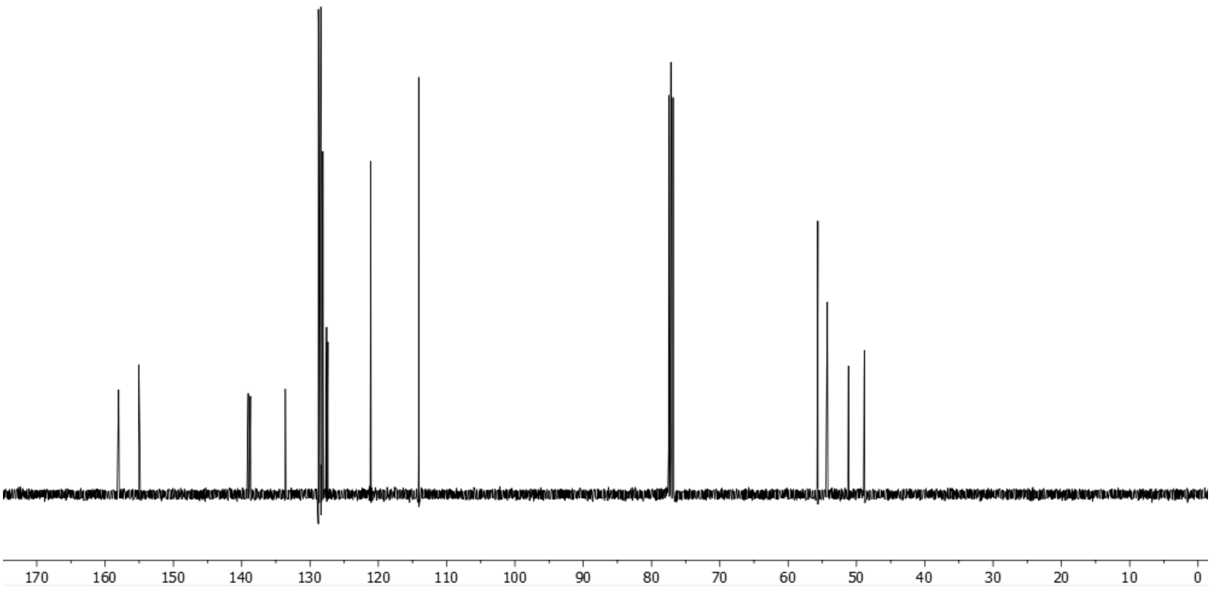

Figure S53 – ^{13}C NMR spectrum of 4-1 (101 MHz, $CDCl_3$).

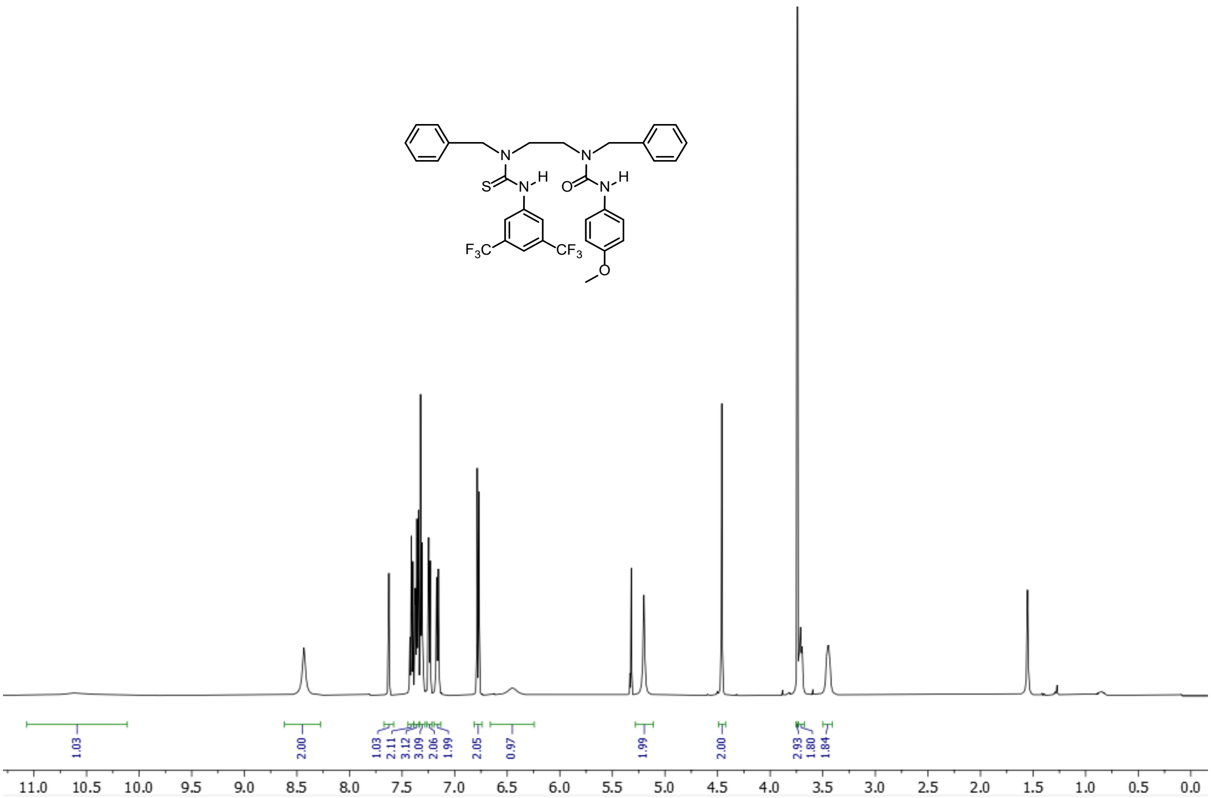

Figure S54 – ^{1}H NMR spectrum of 4 (500 MHz, CD_2Cl_2).

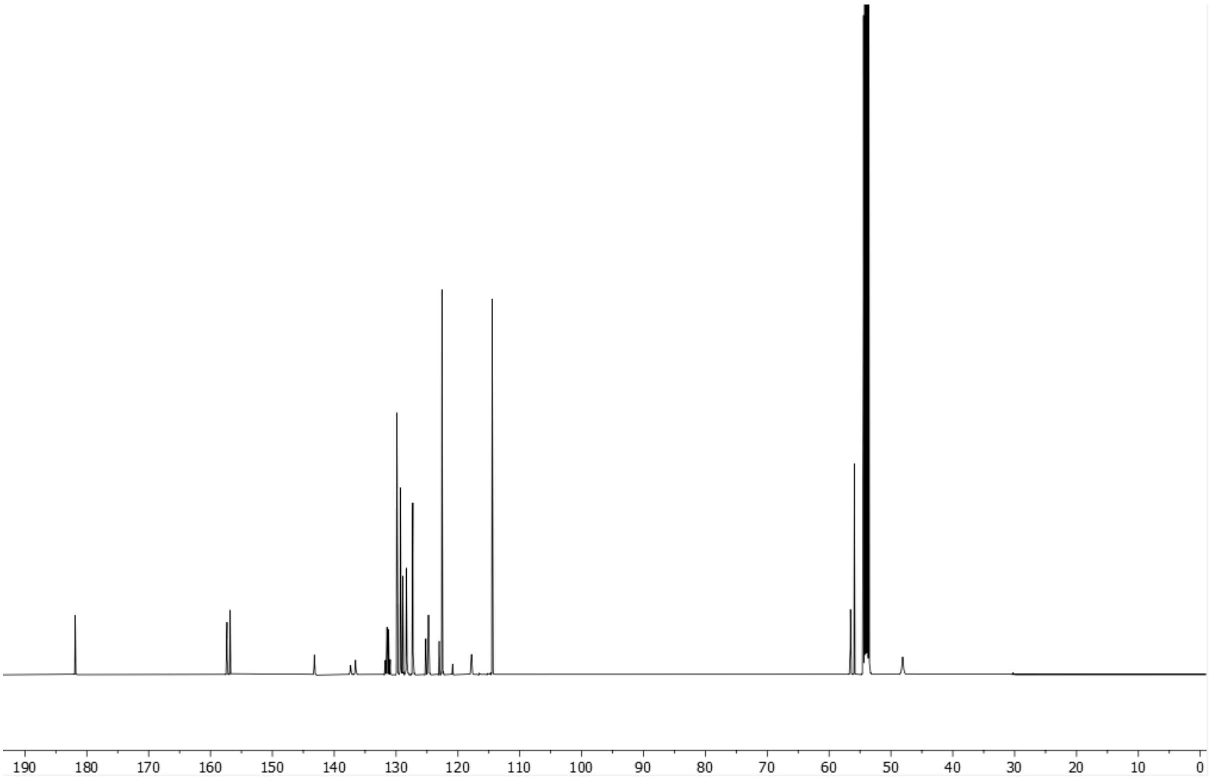

Figure S55 – ^{13}C NMR spectrum of 4 (126 MHz, CD_2Cl_2).

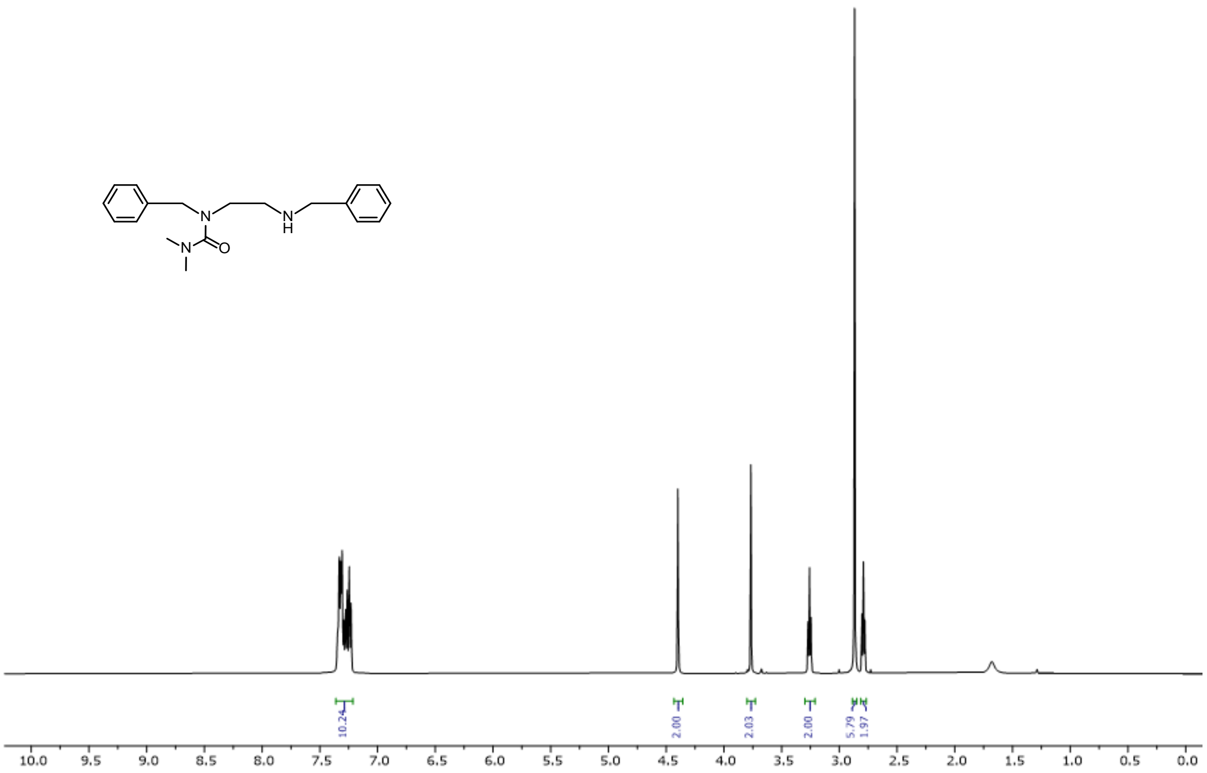

Figure S56 – ^{1}H NMR spectrum of 5-1 (500 MHz, $CDCl_3$).

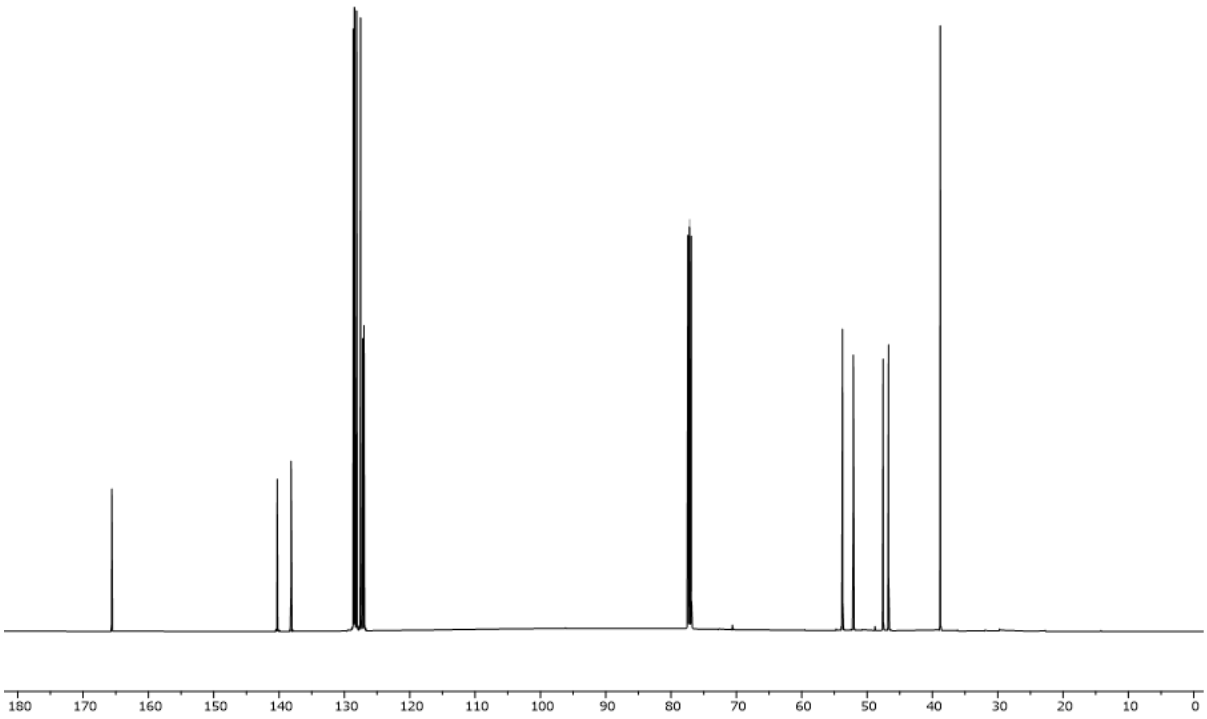

Figure S57 – ^{13}C NMR spectrum of 5-1 (126 MHz, $CDCl_3$).

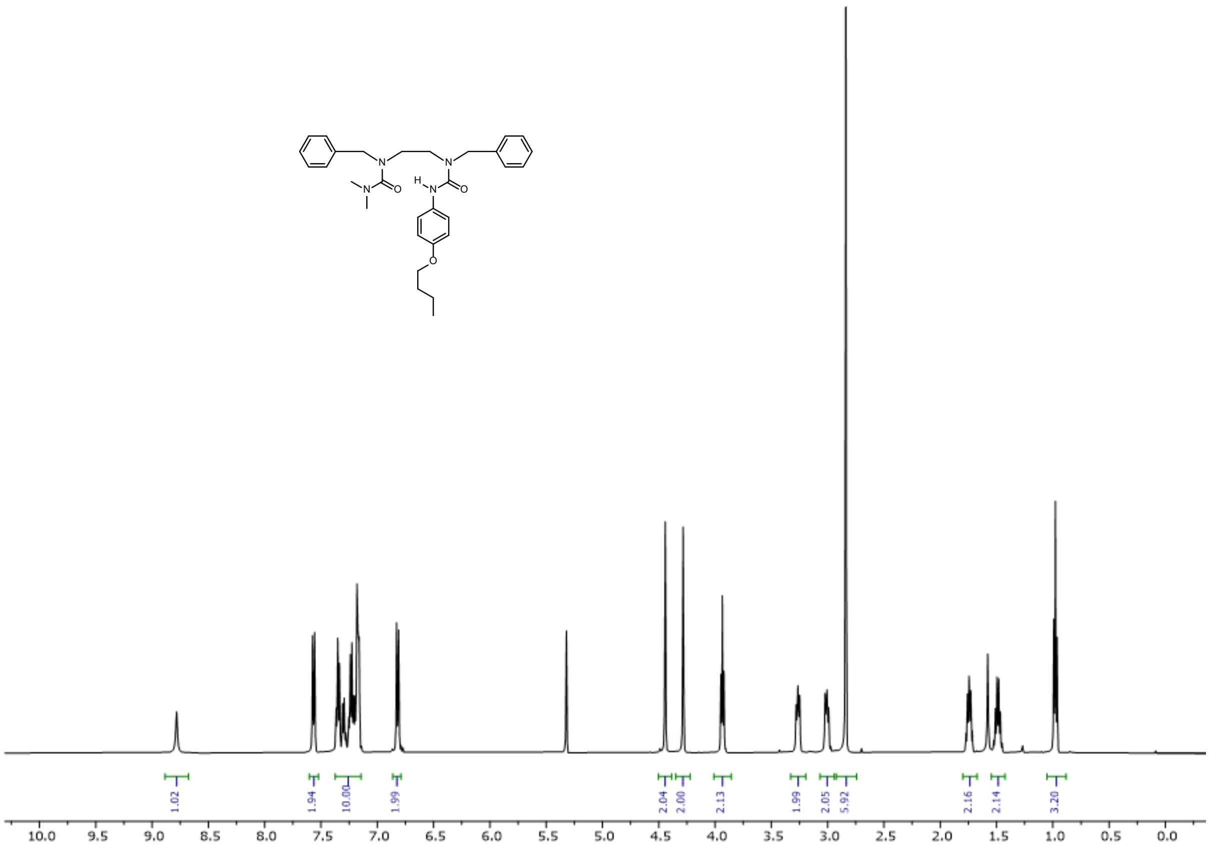

Figure S58 – ^{1}H NMR spectrum of 5 (500 MHz, CD_2Cl_2).

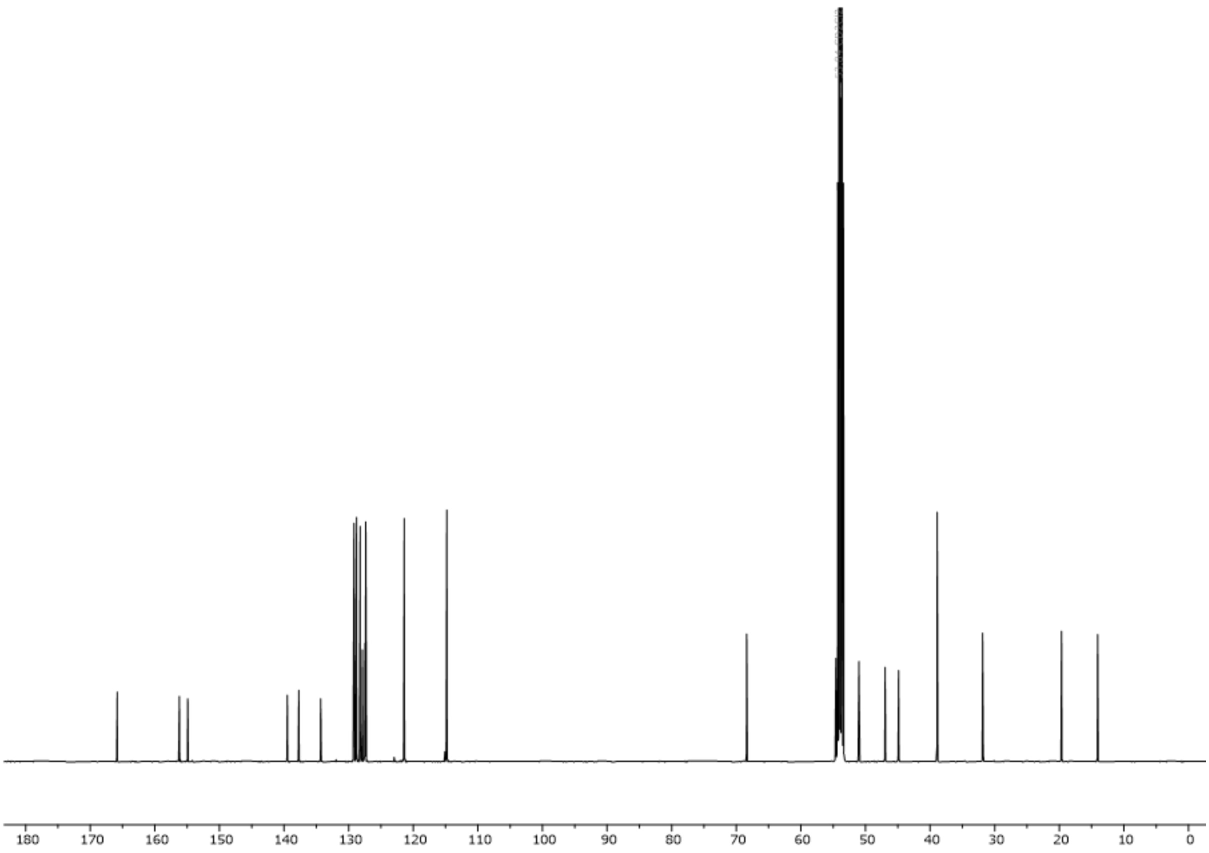

Figure S59 – ^{13}C NMR spectrum of 5 (126 MHz, CD_2Cl_2).

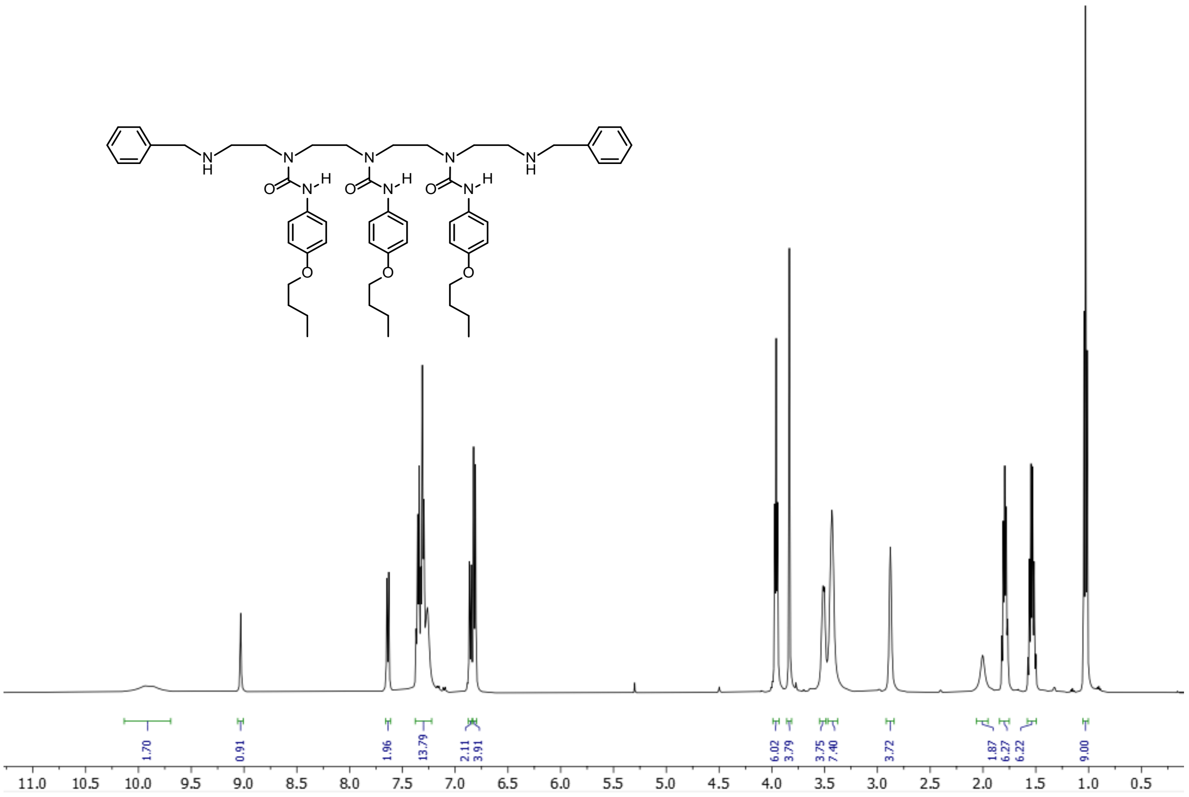

Figure S60 – ^{1}H NMR spectrum of 6-1 (500 MHz, $CDCl_3$).

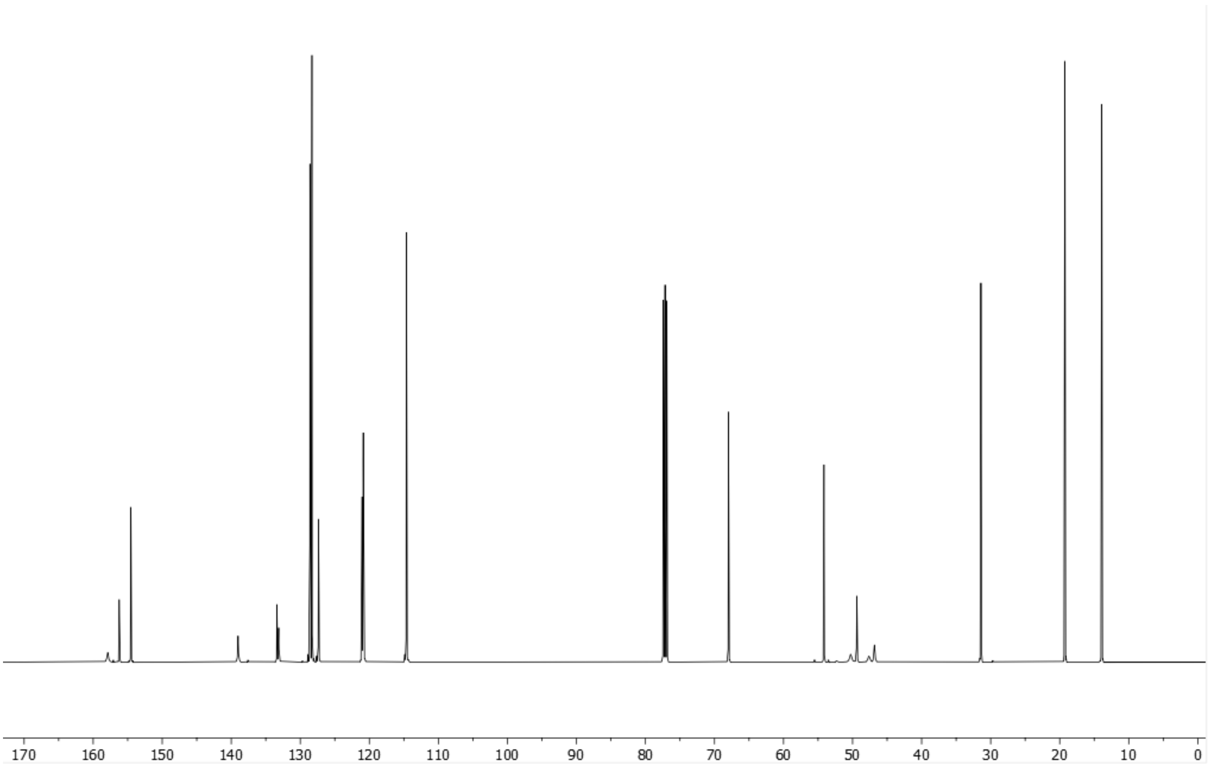

Figure S61 – ^{13}C NMR spectrum of 6-1 (126 MHz, $CDCl_3$).

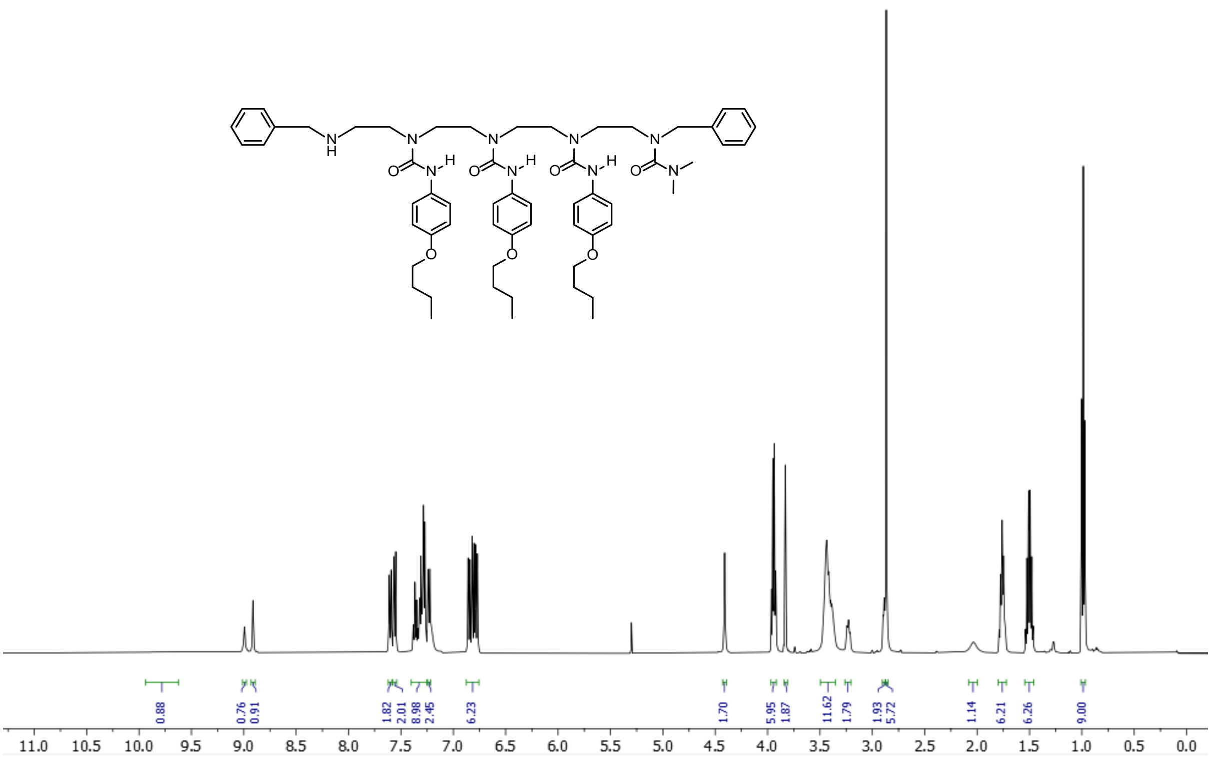

Figure S62 – ^{1}H NMR spectrum of 6-2 (500 MHz, $CDCl_3$).

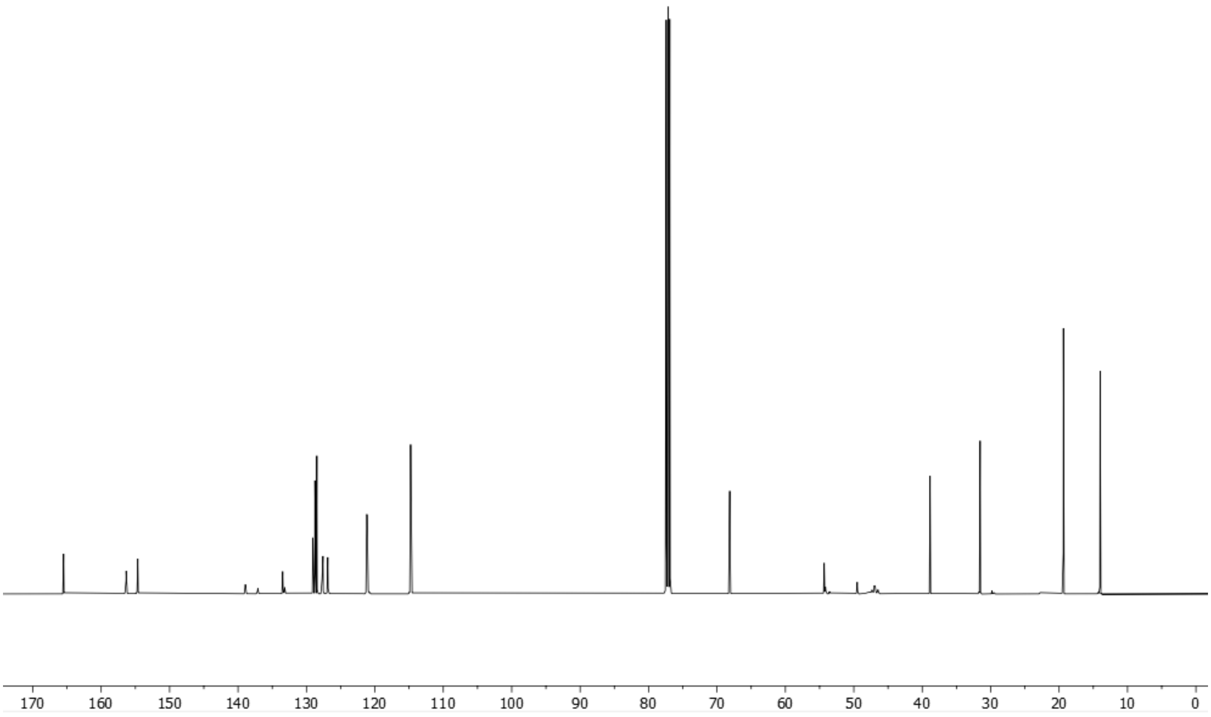

Figure S63 – ^{13}C NMR spectrum of 6-2 (126 MHz, $CDCl_3$).

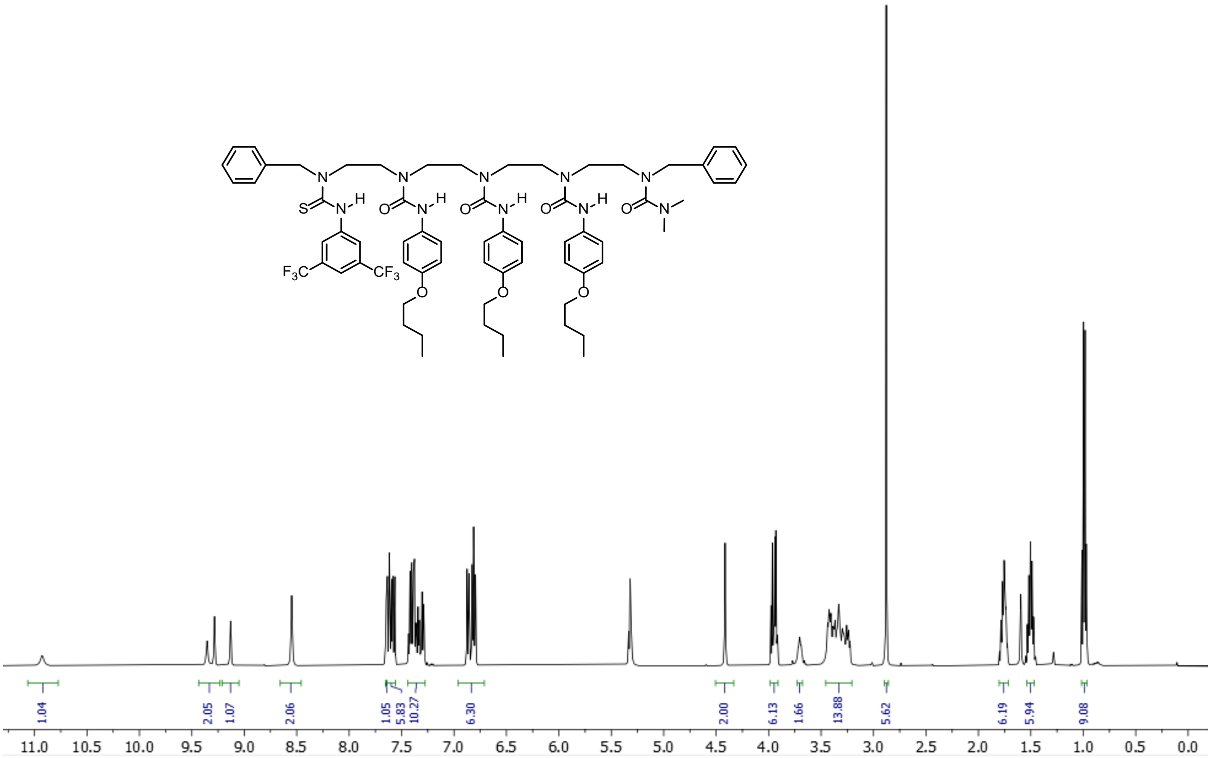

Figure S64 – ^{1}H NMR spectrum of 6 (500 MHz, CD_2Cl_2).

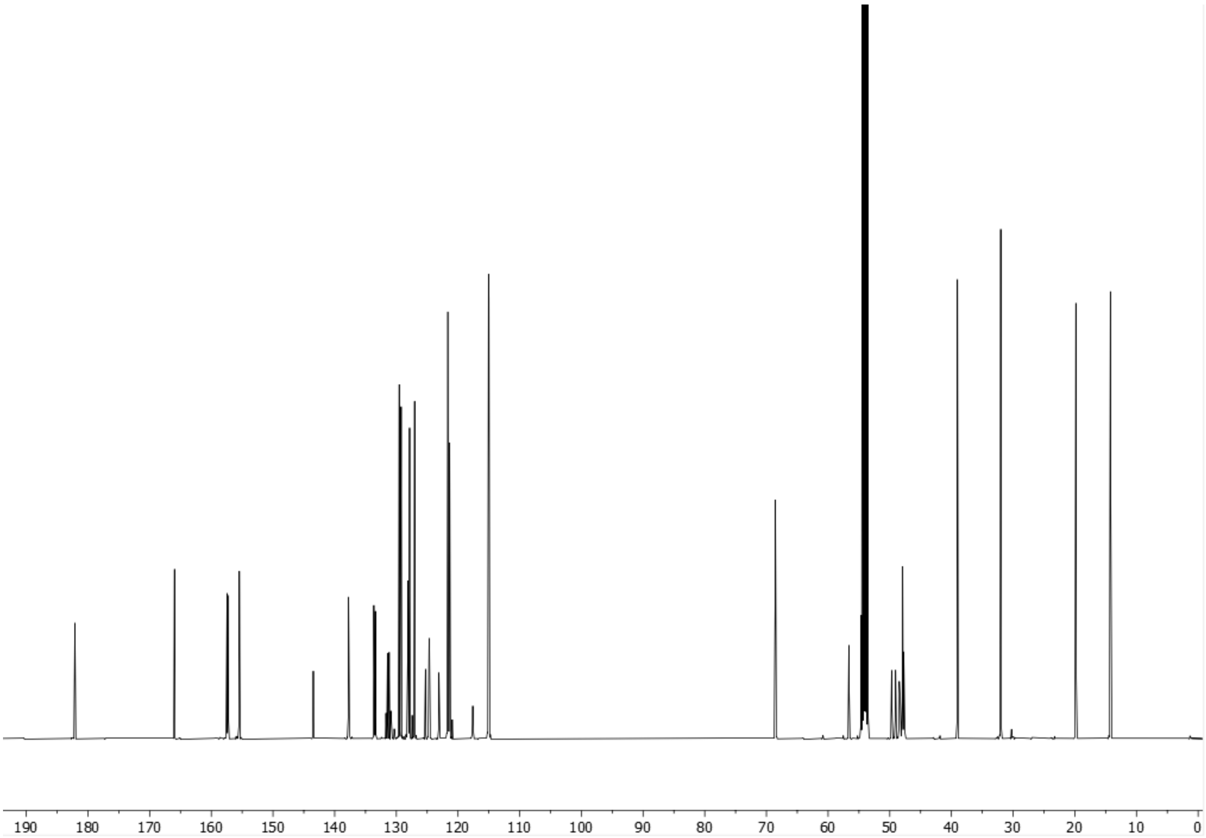

Figure S65 – ^{13}C NMR spectrum of 6 (126 MHz, CD_2Cl_2).

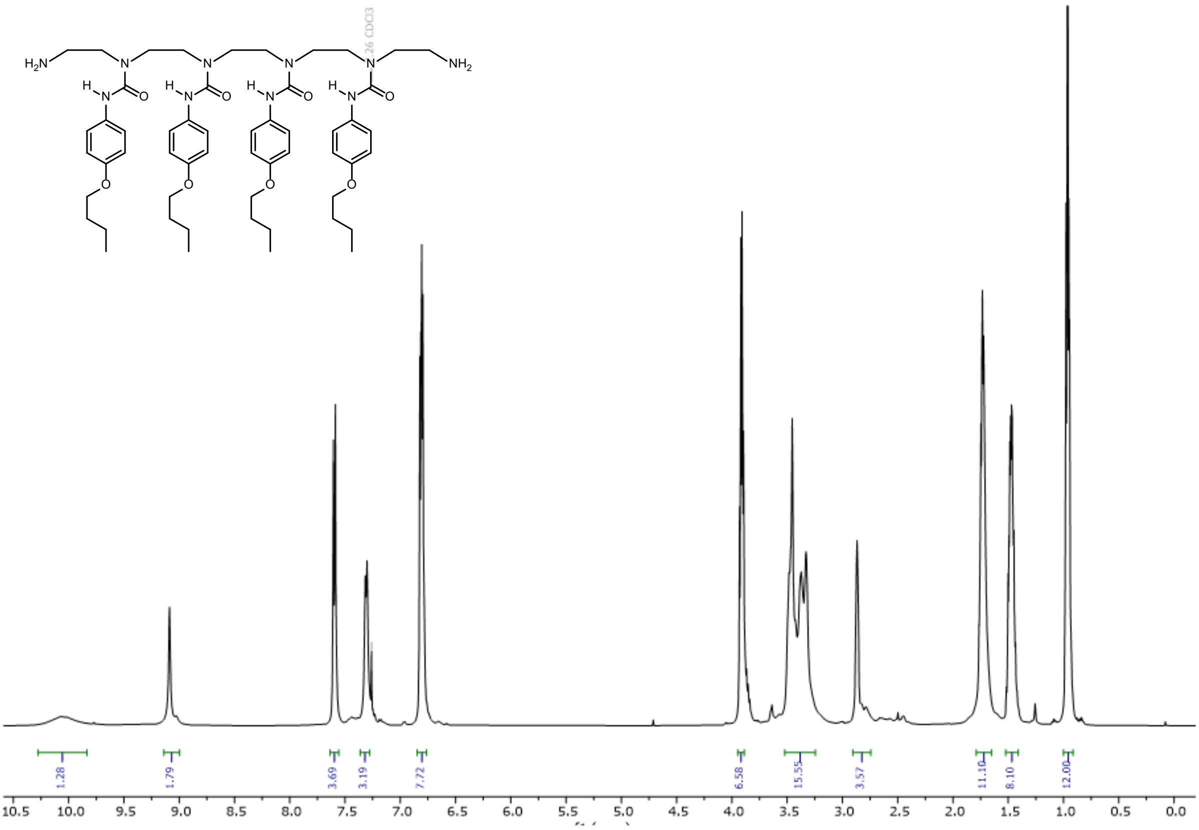

Figure S66 – ^{1}H NMR spectrum of 7-2 (500 MHz, $CDCl_3$).

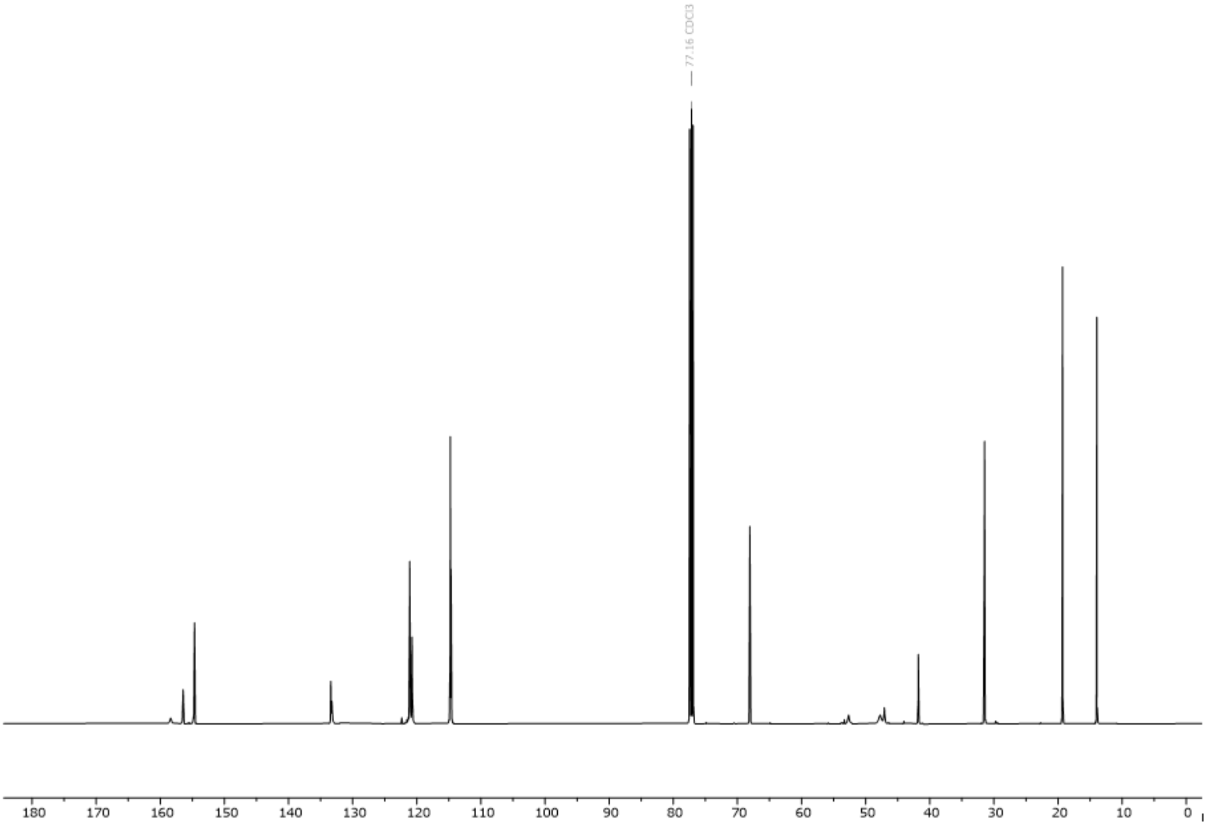

Figure S67 – ^{13}C NMR spectrum of 7-2 (126 MHz, $CDCl_3$).

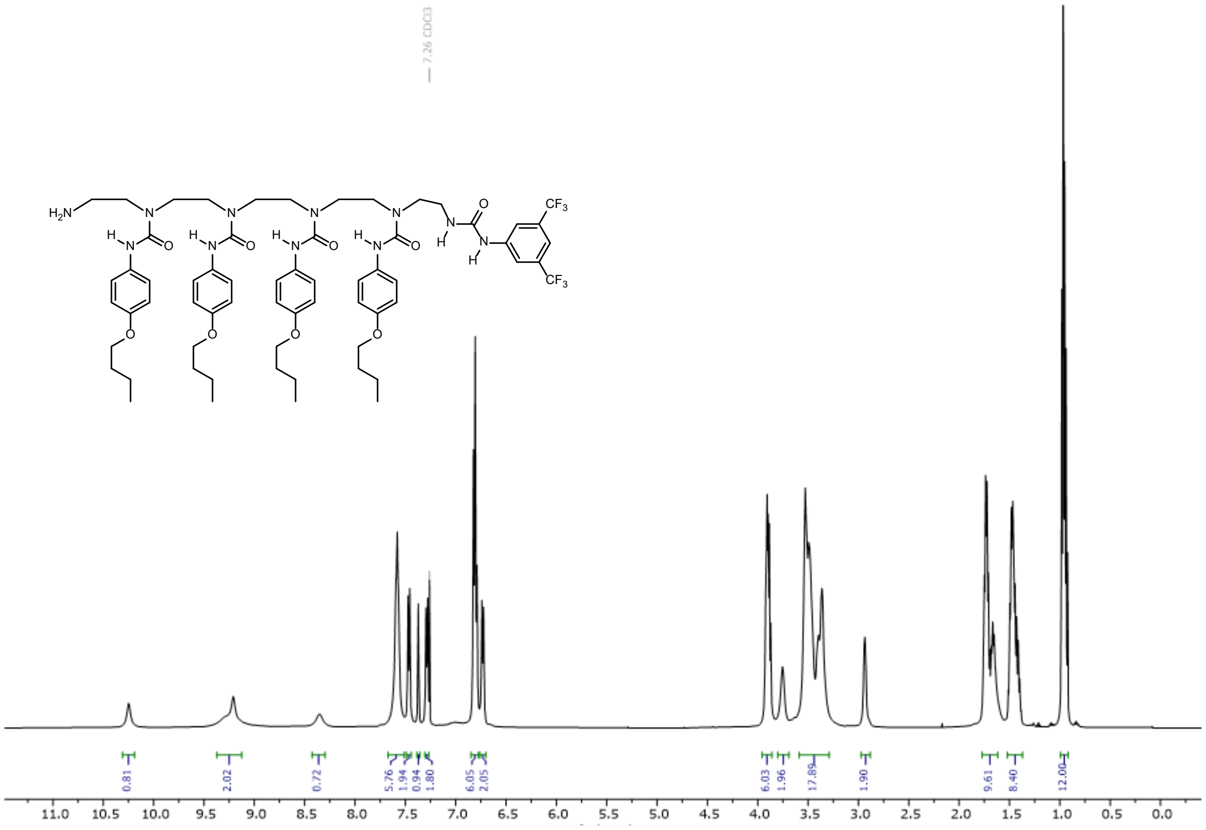

Figure S68 – ^{1}H NMR spectrum of 7-3 (500 MHz, $CDCl_3$).

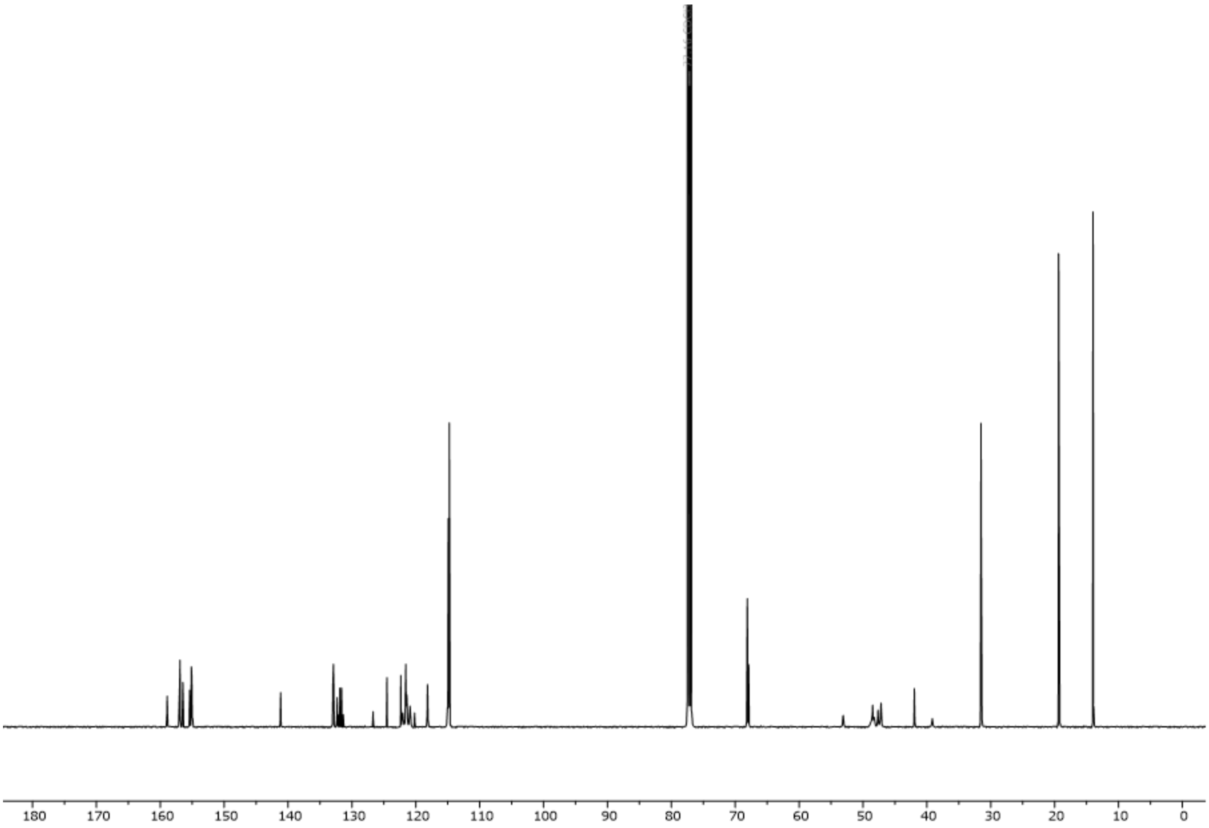

Figure S69 – ^{13}C NMR spectrum of 7-3 (126 MHz, $CDCl_3$).

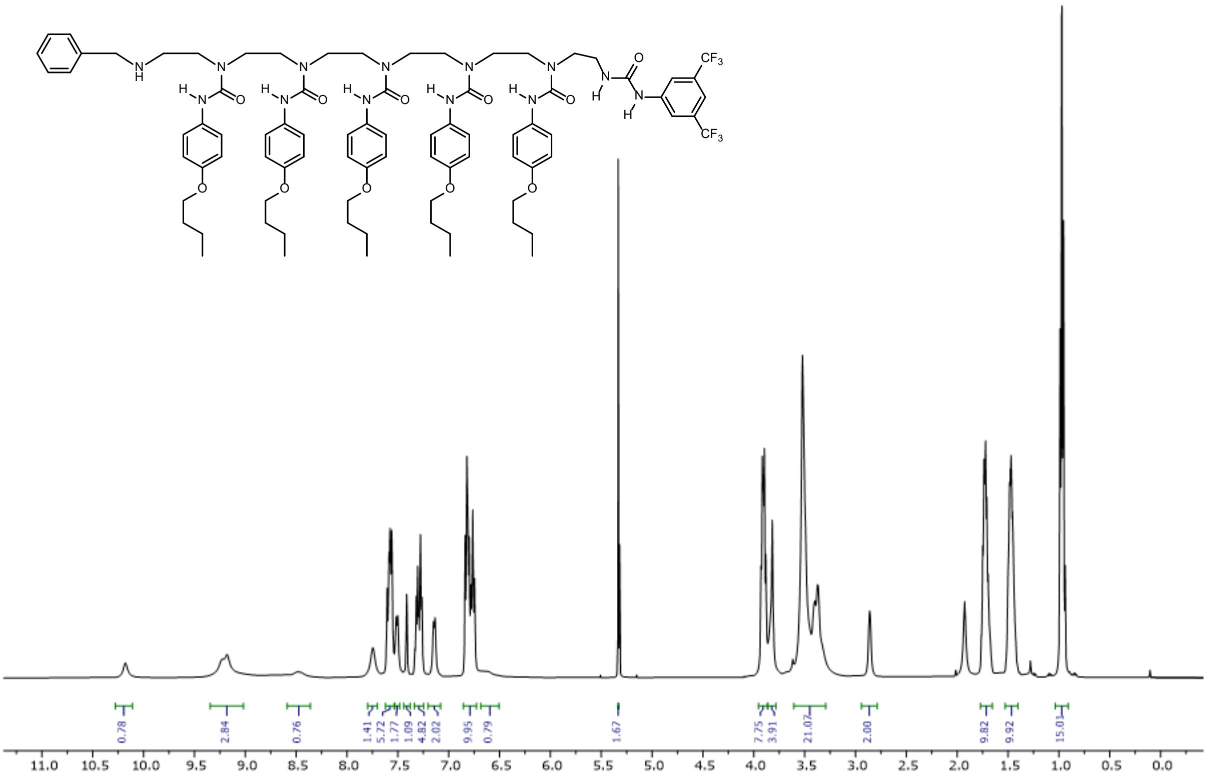

Figure S70 – ^{1}H NMR spectrum of 7-4 (500 MHz, CD_2Cl_2).

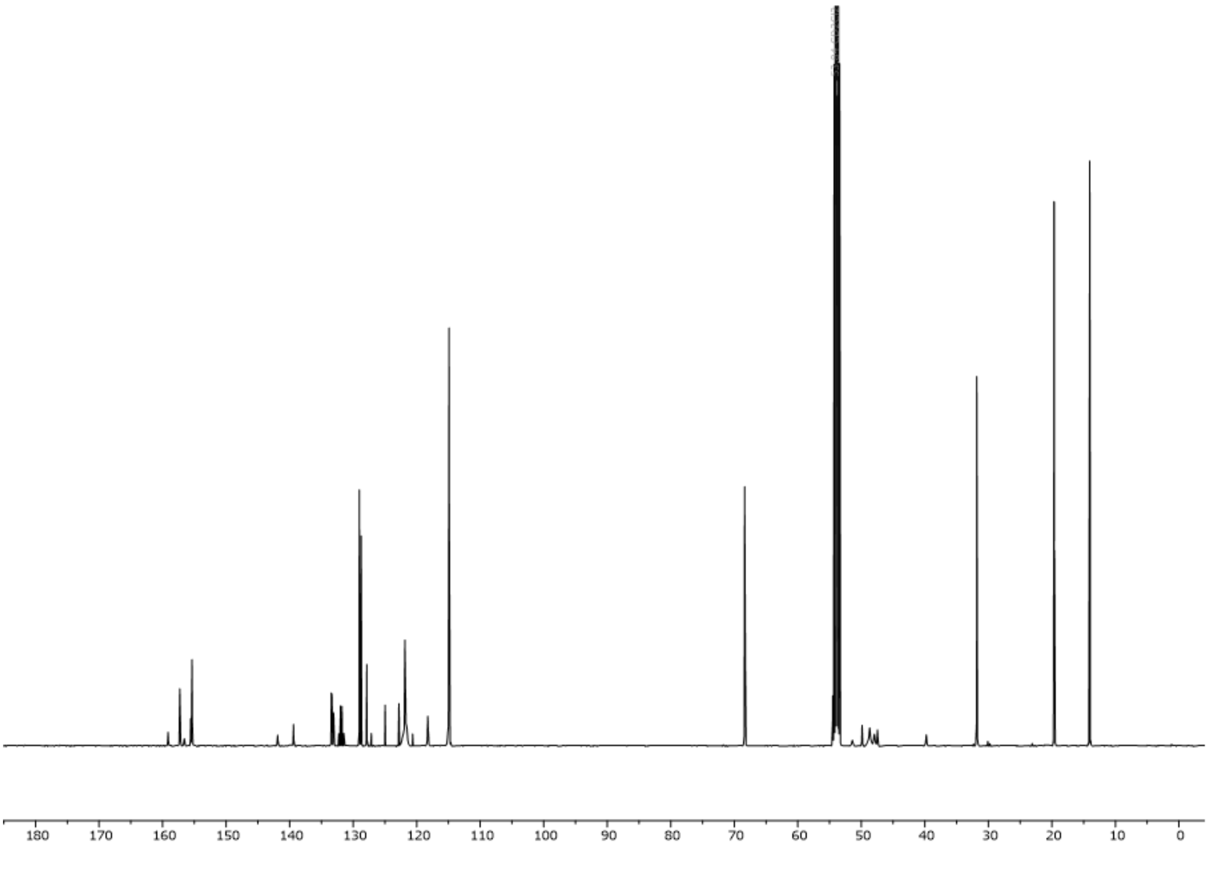

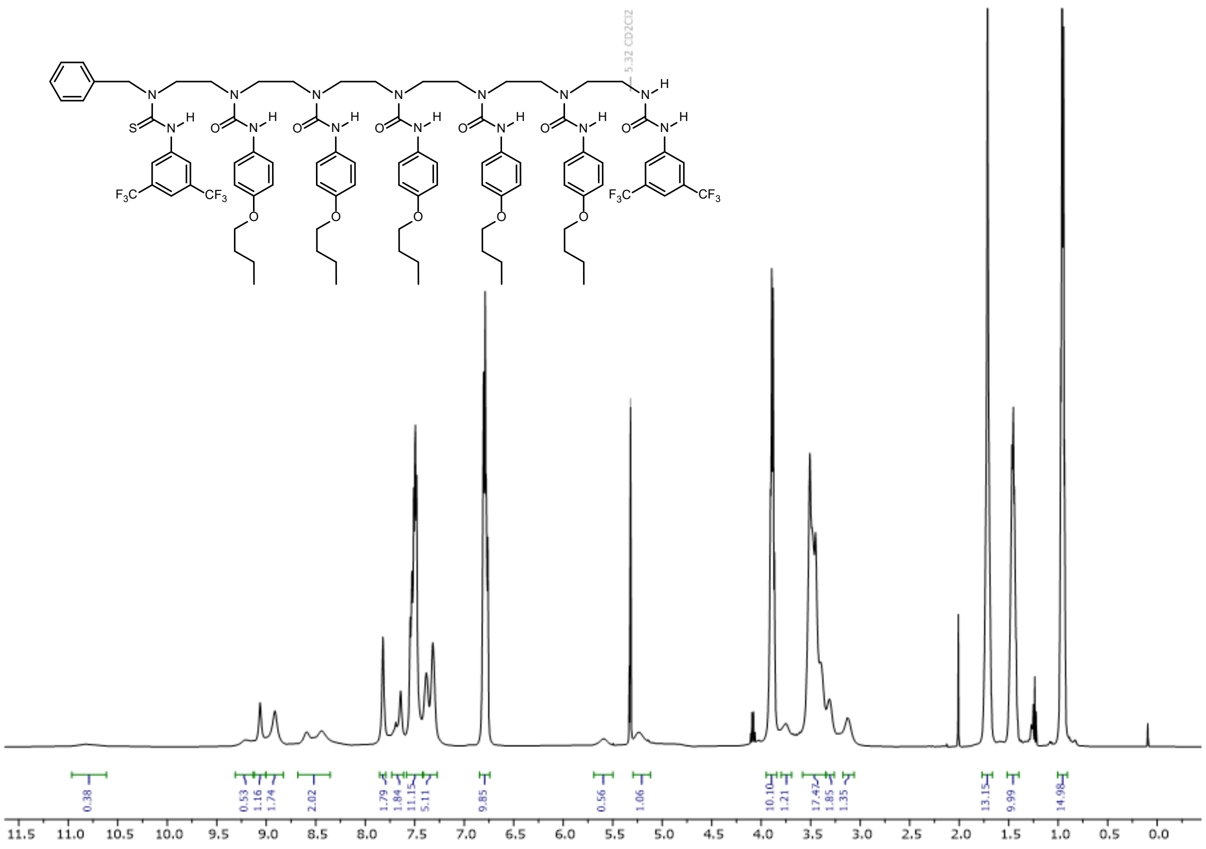

Figure S72 – ^{1}H NMR spectrum of 7 (500 MHz, CD_2Cl_2).

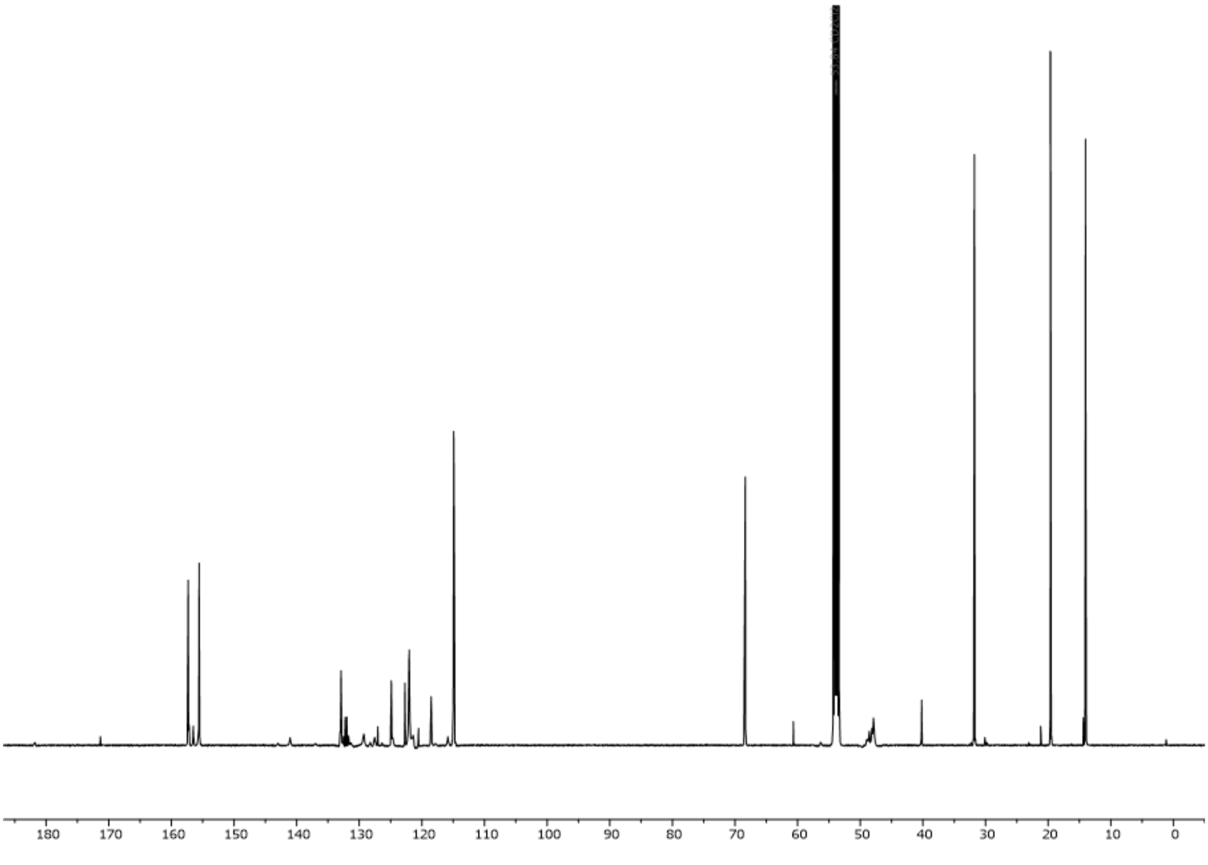

Figure S73 – ^{13}C NMR spectrum of 7 (126 MHz, CD_2Cl_2).

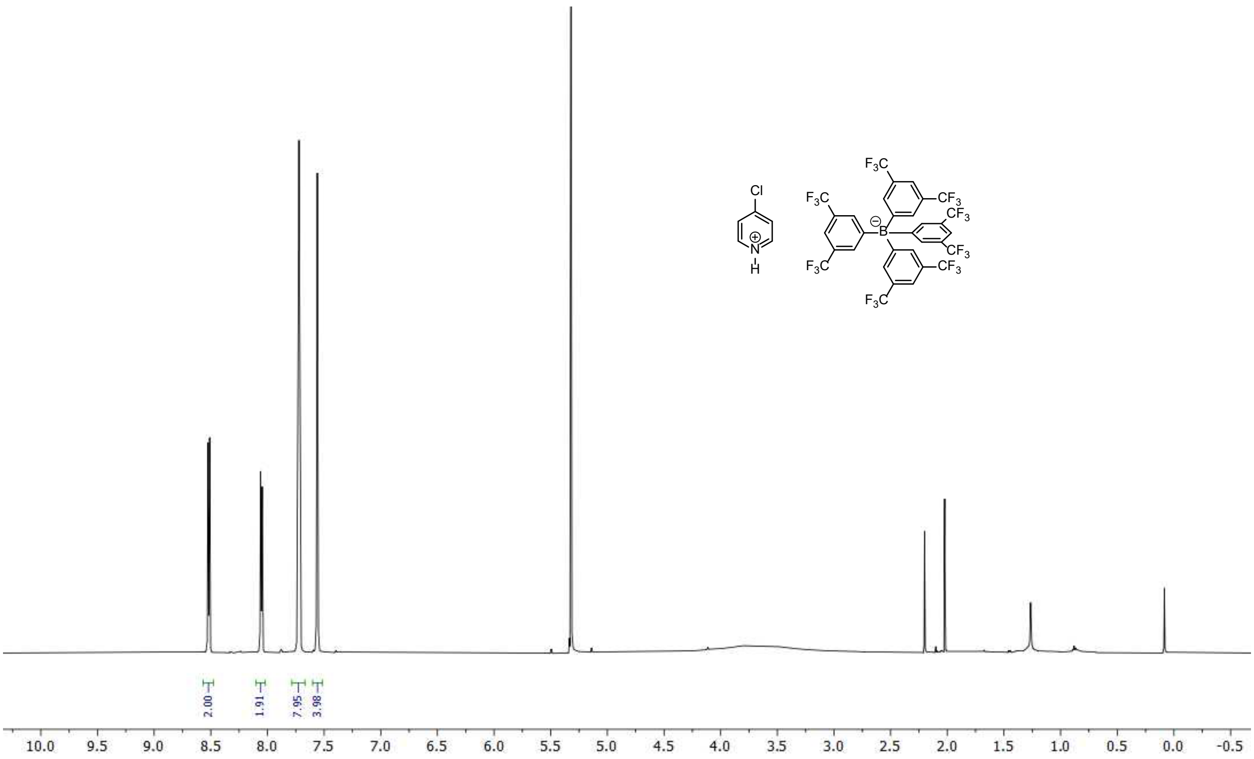

Figure S74 – ^{1}H NMR spectrum of S8 (500 MHz, CD_2Cl_2).

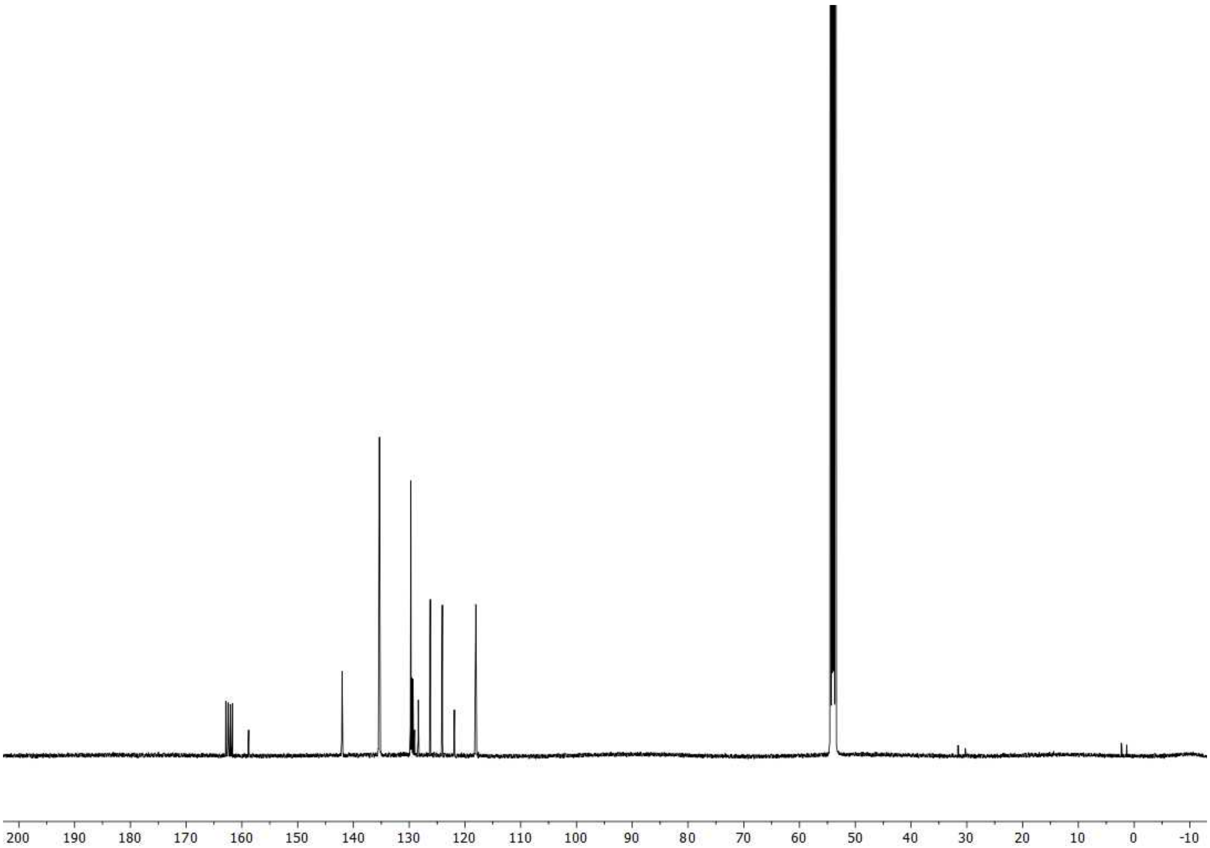

Figure S75 – ^{13}C NMR spectrum of S8 (126 MHz, CD_2Cl_2).

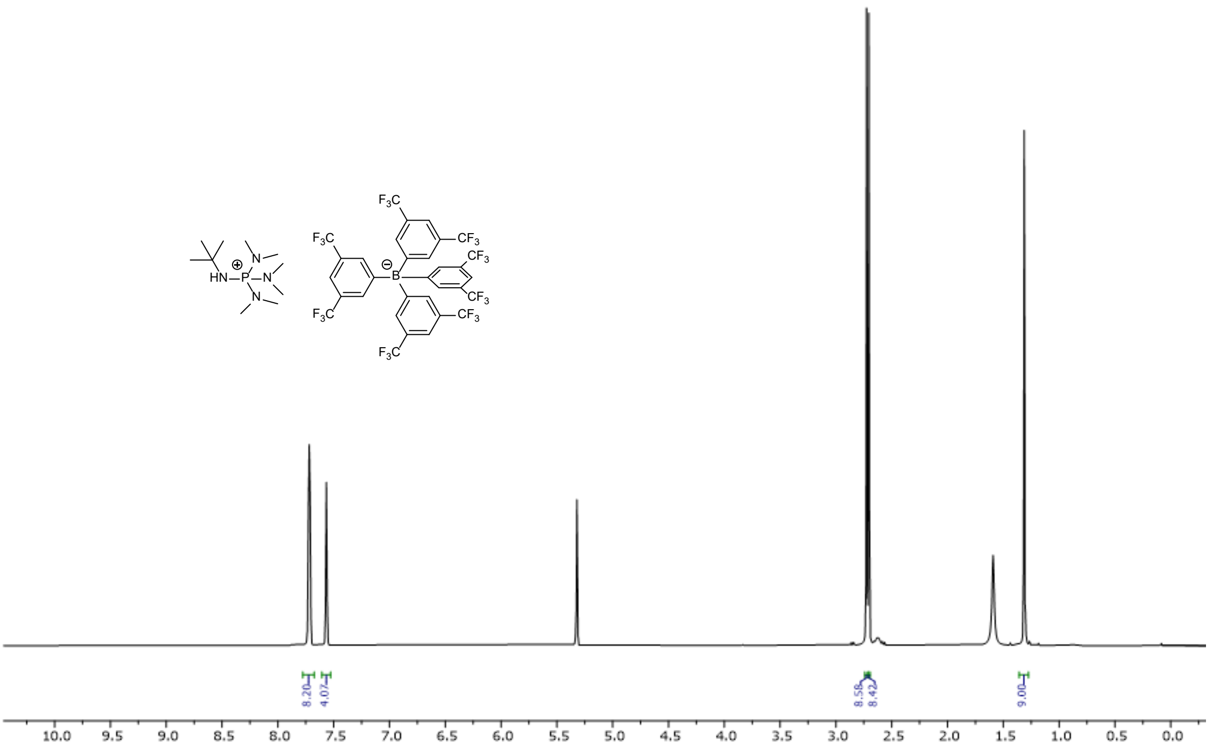

Figure S76 – ^{1}H NMR spectrum of S9 (500 MHz, CD_2Cl_2).

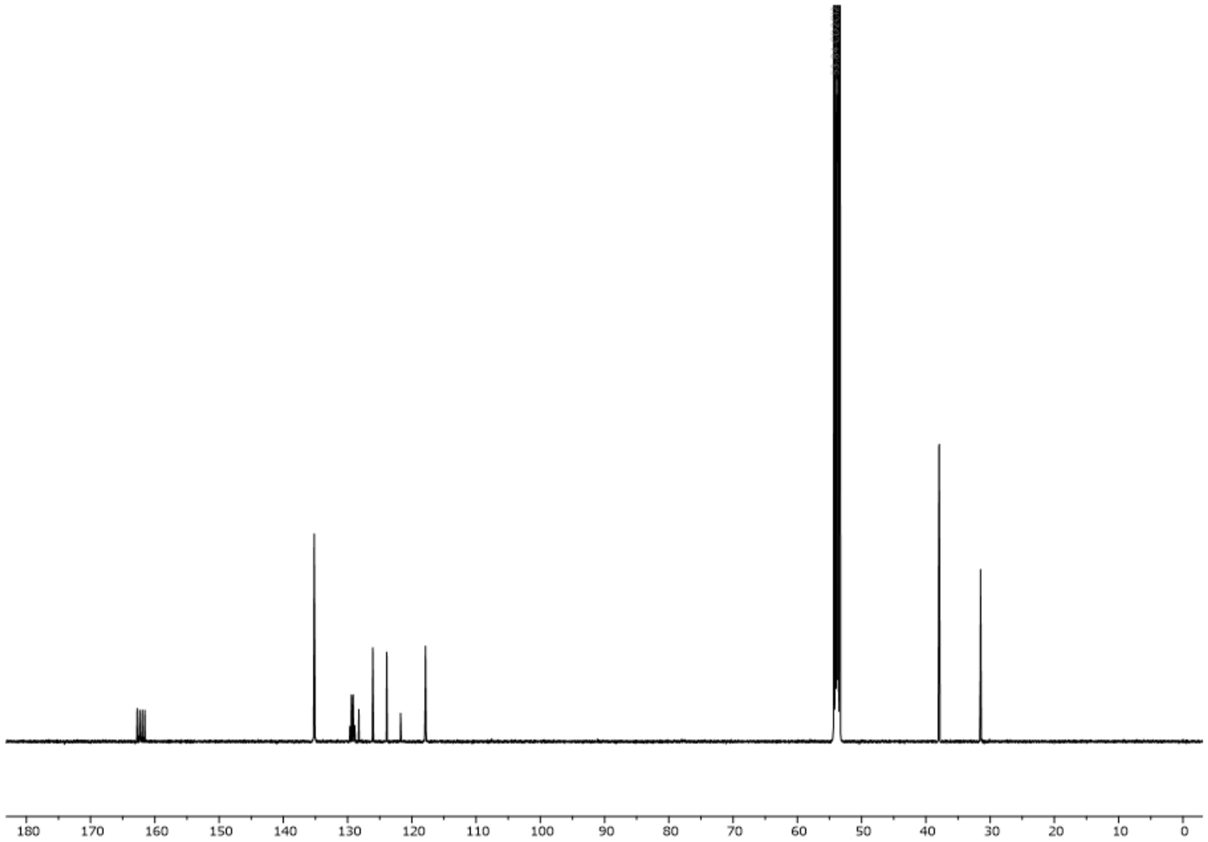

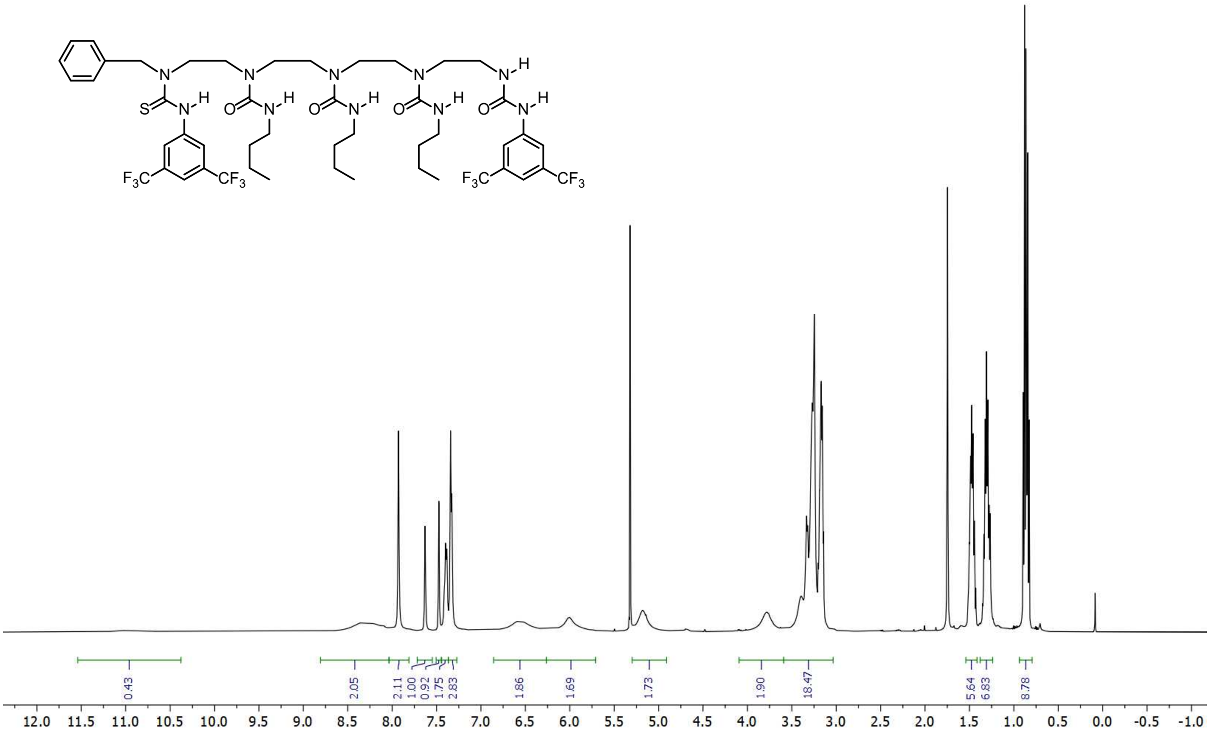

Figure S78 – ^{1}H NMR spectrum of S10 (500 MHz, CD_2Cl_2).

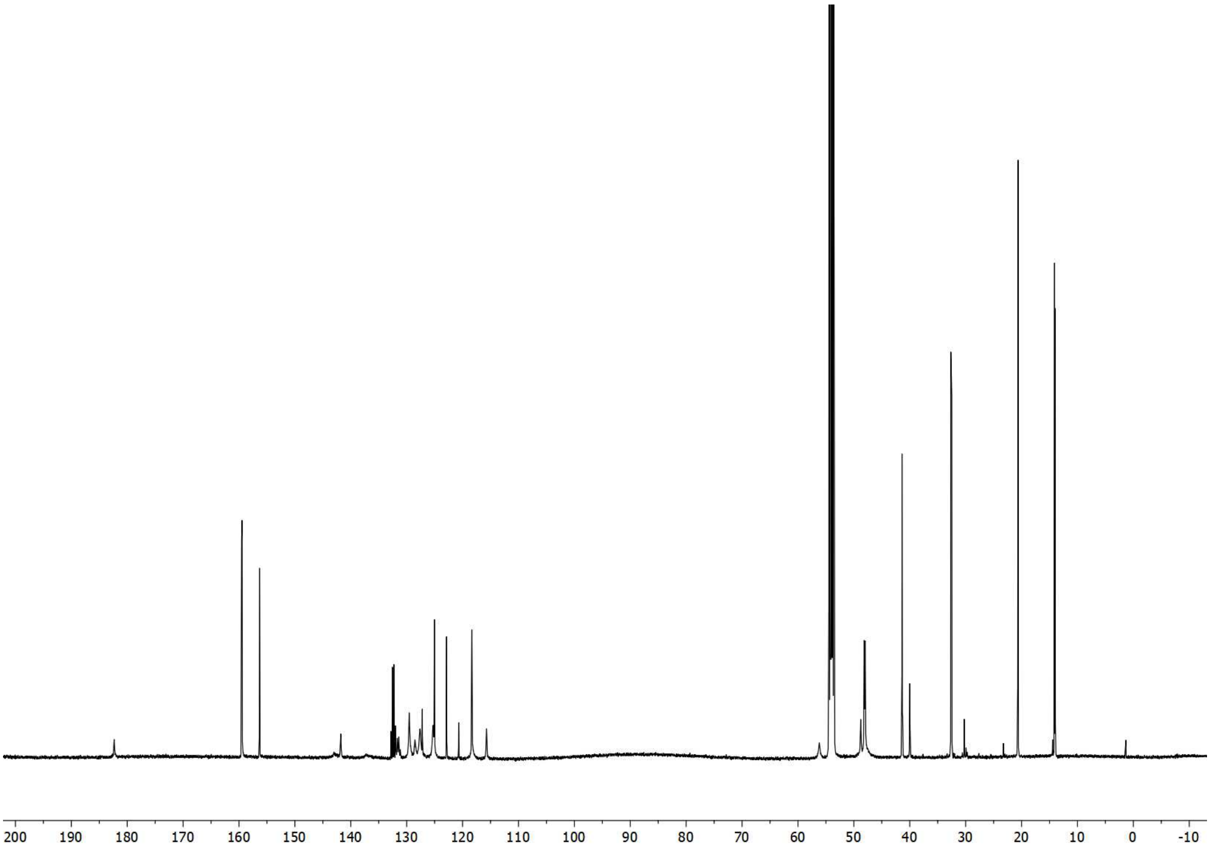

Figure S79 – ^{13}C NMR spectrum of S10 (126 MHz, CD_2Cl_2).

References

1. Morris, D. T. J.; Wales, S. M.; Tilly, D. P.; Farrar, E. H. E.; Grayson, M. N.; Ward, J. W.; Clayden, J. A molecular communication channel consisting of a single reversible chain of hydrogen bonds in a conformationally flexible oligomer. *Chem* **2021**, *7*, 2460–2472. DOI: 10.1016/j.chempr.2021.06.022
2. Chang, J.-C.; Tseng, S.-H.; Lai, C.-C.; Liu, Y.-H.; Peng, S.-M.; Chiu, S.-H., Mechanically interlocked daisy-chain-like structures as multidimensional molecular muscles. *Nat. Chem.* **2017**, *9*, 128–134. DOI: 10.1038/NCHEM.2608
3. Thota, S.; Jeon, S.; Wang, M.; Chiang, L. Y. Synthesis of Cationic Dumbbell-shaped Fullerene Nanostructures as Potential Photodynamic Sensitizers. *J. Mol. Sci.* **2010**, *47*, 1184–1190. DOI: 10.1080/10601325.2010.518838
4. (a) Thordarson, P. Determining Association Constants from Titration Experiments in Supramolecular Chemistry. *Chem. Soc. Rev.* **2011**, *40*, 1305. DOI: 10.1039/C0CS00062K (b) www.supramolecular.org.
5. Nödling, A. R.; Jakab, G.; Schreiner, P. R.; Hilt, G. ^{31}P NMR Spectroscopically Quantified Hydrogen-Bonding Strength of Thioureas and Their Catalytic Activity in Diels–Alder Reactions. *Eur. J. Org. Chem.* **2014**, *2014*, 6394–6398. DOI: 10.1002/ejoc.201402871
